# The pearl oyster *Pinctada fucata martensii* genome and multi-omic analyses provide insights into biomineralization

**DOI:** 10.1093/gigascience/gix059

**Published:** 2017-07-25

**Authors:** Xiaodong Du, Guangyi Fan, Yu Jiao, He Zhang, Ximing Guo, Ronglian Huang, Zhe Zheng, Chao Bian, Yuewen Deng, Qingheng Wang, Zhongduo Wang, Xinming Liang, Haiying Liang, Chengcheng Shi, Xiaoxia Zhao, Fengming Sun, Ruijuan Hao, Jie Bai, Jialiang Liu, Wenbin Chen, Jinlian Liang, Weiqing Liu, Zhe Xu, Qiong Shi, Xun Xu, Guofan Zhang, Xin Liu

**Affiliations:** 1 Fishery College, Guangdong Ocean University, Zhanjiang, 524025, China; 2 BGI-Qingdao, Qingdao 266555, China; 3 BGI-Shenzhen, Shenzhen, 518083 China; 4 Haskin Shellfish Research Laboratory, Department of Marine and Coastal Sciences, Rutgers University, Port Norris, NJ 08349, USA; 5 Atlantic Cape Community College, Mays Landing, NJ 08330, USA; 6 Key Laboratory of Experimental Marine Biology, Institute of Oceanology, Chinese Academy of Sciences, Qingdao 266071, China; 7 Laboratory for Marine Biology and Biotechnology, Qingdao National Laboratory for Marine Science and Technology, Qingdao, China; 8 National & Local Joint Engineering Laboratory of Ecological Mariculture, Qingdao 266071, China

**Keywords:** genome, biomineralization, nacre, VWA-containing protein, *Pinctada fucata martensii*

## Abstract

Nacre, the iridescent material found in pearls and shells of molluscs, is formed through an extraordinary process of matrix-assisted biomineralization. Despite recent advances, many aspects of the biomineralization process and its evolutionary origin remain unknown. The pearl oyster *Pinctada fucata martensii* is a well-known master of biomineralization, but the molecular mechanisms that underlie its production of shells and pearls are not fully understood. We sequenced the highly polymorphic genome of the pearl oyster and conducted multi-omic and biochemical studies to probe nacre formation. We identified a large set of novel proteins participating in matrix-framework formation, many in expanded families, including components similar to that found in vertebrate bones such as collagen-related VWA-containing proteins, chondroitin sulfotransferases, and regulatory elements. Considering that there are only collagen-based matrices in vertebrate bones and chitin-based matrices in most invertebrate skeletons, the presence of both chitin and elements of collagen-based matrices in nacre suggests that elements of chitin- and collagen-based matrices have deep roots and might be part of an ancient biomineralizing matrix. Our results expand the current shell matrix-framework model and provide new insights into the evolution of diverse biomineralization systems.

## Background

Biomineralization is an extraordinary process where minerals form not following rules of inorganic chemistry but through active biological facilitation and control. Biomineralization is widely distributed and essential to the lives of diverse organisms, ranging from algae to vertebrates that rely on mineralized materials for morphology, structure, protection, movement, and feeding. Three principal classes of skeletal biominerals exist on earth: calcium carbonate, calcium phosphate, and silica [1]. Whether these skeletal biominerals evolved independently or derived from a common origin is controversial, although current thinking favours independent evolution [2]. One of the remarkable characteristics of biomineralization is its precise control by organic matrices [3]. Organic matrices are complex and variable but can be classified into 2 basic and highly conserved types that use either chitin or collagen as the templating framework [3]. Despite great interest in harnessing the power of biomineralization for the production of novel materials, our understanding of biomineralization and associated matrices is limited in many taxa, including the well-known masters of biomineralization—shelled molluscs.

Nacre is the remarkable biomineral found in pearls and shells of molluscs that provides lustre and enhanced toughness. The formation of lustrous pearls and shells in molluscs such as the pearl oyster *Pinctada fucata martensii* has long fascinated humans. The biomineralization process of nacre formation is complex and involves sophisticated organic matrices as well as cells, many aspects of which remain unclear [4–8]. The origin and homology of nacre formation with other biomineralization processes such as crustacean shell and vertebrate bone formation are not understood [9]. Studies of biomineralization and other fundamental questions in biology and evolution can be greatly empowered by whole-genome analyses, which have been difficult in molluscs owing to challenges in assembling their highly polymorphic and complex genomes [6, 10]. To understand the biomineralization process of nacre, we sequenced and assembled the *P. f. martensii* genome and generated transcriptomes from 11 organs/tissues and 12 developmental stages, along with proteomes of shell organic matrices.

## Data Description

We used a pearl oyster from the third-generation breeding line, selected for fast growth for sequencing and assembly using a BAC-to-BAC strategy. In addition to BAC sequencing, we also constructed whole-genome shotgun (WGS) libraries, including 3 with short insert sizes and 4 with long insert sizes. We used a draft assembly from a previous study [10], Sanger-sequenced BACs, and transcripts generated by RNA-seq to assess the integrity of our assembly. Furthermore, to anchor scaffolds to chromosomes, we constructed a genetic map using restriction site–associated DNA sequencing (RAD-seq) using 148 F1 offspring obtained by crossing 2 genetically distant parents.

To determine the gene expression profiles in different organs or tissues, we performed transcriptome sequencing on 11 organs and tissues, including adductor muscle, mantle, mantle pallium, mantle edge, hepatopancreas, hemocyte, gonad, gill, foot, and pearl sac at 180 days after nucleus transplantation. Further, we sequenced transcriptomes of 12 developmental samples to determine the gene expression profile during development. Developmental samples included unfertilized eggs, 11 samples obtained at 30 minutes, 5 hours, 6 hours, 8 hours, 16 hours, 19 hours, 4 days, 14 days, 28 days, 40 days, and 90 days after fertilization. To understand gene regulation during nacre formation, we analyzed transcriptome data from mantle edge, mantle pallial, and 2 entire mantle tissues representing fast- and slow-growing pearl oysters with weighted-gene co-expression network analysis (WGCNA) and obtained co-expression network patterns. All sequencing and genome data were uploaded to NCBI under the accession number BioProject: PRJNA283019.

## Results

### Genome assembly and characterization

As our initial assembly of ∼130 Gb (134-fold coverage) of whole-genome shotgun (WGS) Illumina sequences (Additional file 1: Table S1) was too fragmented for annotation and analysis, probably due to high polymorphism and repetitive sequences (Additional file 2: Fig. S1a and b), we subsequently adopted a bacterial artificial chromosome (BAC)-to-BAC sequencing strategy [6, 11]. We sequenced 46 080 BACs (5-fold genome coverage) to a depth of ×100 using Illumina next-generation sequencing (NGS), assembled each BAC separately (Additional file 2: Fig. S1c), and then built supercontigs after merging and filtering redundant sequences. After constructing scaffolds and filling gaps with WGS reads, we obtained a final assembly of 990 658 107 bp with a contig N50 size of 21 kb and a scaffold N50 of 324 kb (Additional file 1: Table S2), which was a significant improvement compared with the contig N50 of 1.6 kb of the previous draft assembly [10].

**Figure 1:**
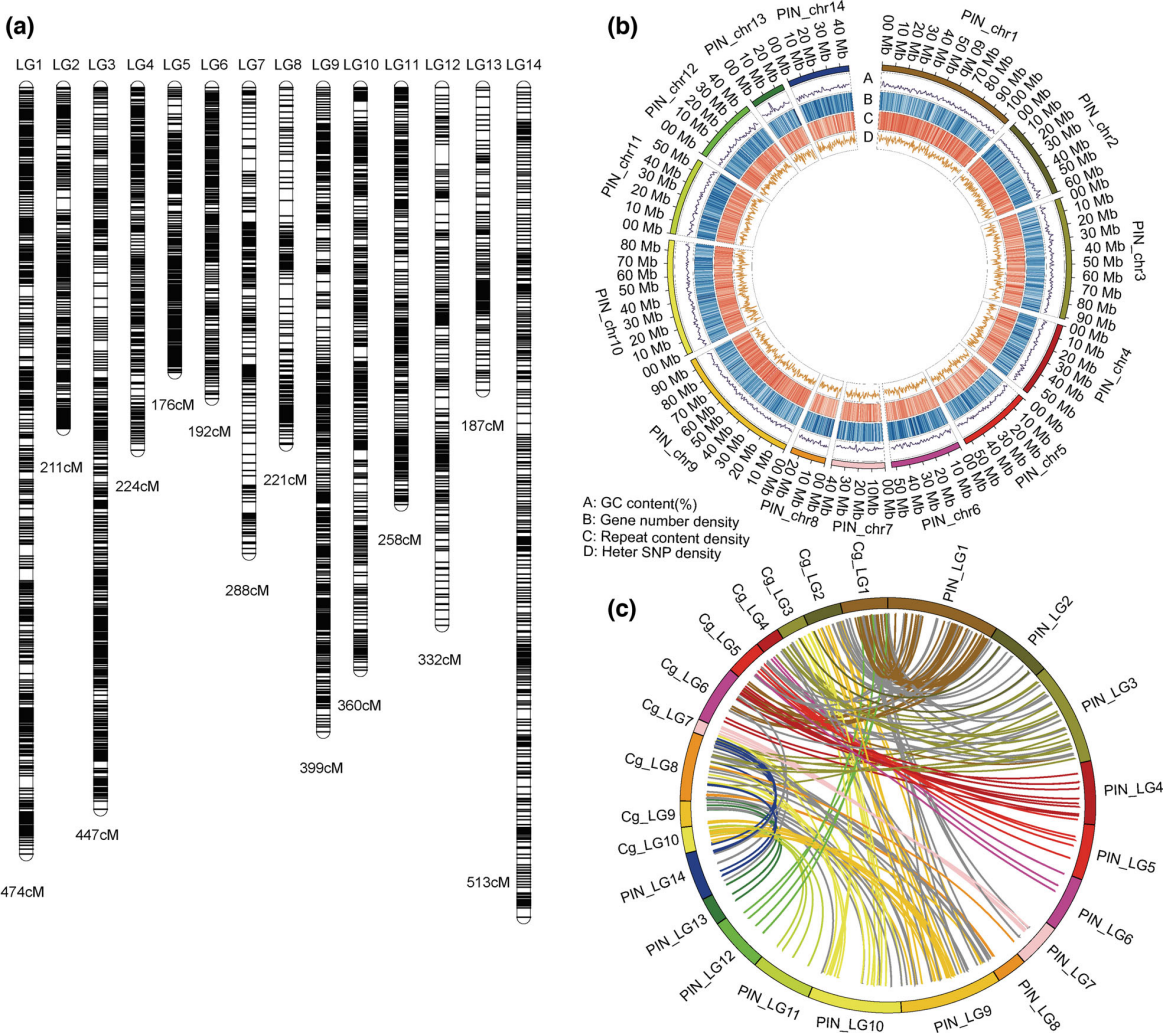
Genome organization of *P. f. martensii*. (**a**) Genetic map of *P. f. martensii* constructed with RAD SNPs. The lines on linkage groups represent SNP positions. (**b**) The distribution of GC, gene, repetitive elements, and SNPs on *P. f. martensii* pseudochromosomes. (**c**) Synteny blocks between *C. gigas* (Cg) and *P. f. martensii* (PIN).

The coverage of our assembly was demonstrated by the successful mapping of 90.5% of contigs, 95.5% (coverage ≥ 50%) of gene-model regions of the previous draft assembly [10], 99.8% of transcripts (coverage ≥ 50%), and all 4 BACs (coverage ≥ 93.2%) sequenced with Sanger technology (Additional file 1: Table S3 and S4; Additional file 3: Fig. S2). We constructed a high-density genetic map of 14 linkage groups in accordance with the haploid number, using RAD-seq of a full-sib family (Fig. 1). We were able to anchor 857.07 Mb (86.5%) of scaffolds to the genetic map with 4463 single nucleotide polymorphisms (SNPs) (Fig. 1a and b; Additional file 1: Table S5). Through alignment of our pseudochromosomes to that of *Crassostrea gigas*, we identified 2240 syntenic blocks and several possible chromosome rearrangements (Fig. 1c).

Combining *de novo* prediction and evidence-based annotation using published data and transcriptomes from 11 organs/tissues and 12 developmental stages (Additional file 1: Table S6), we identified 32 937 protein-coding gene models (Additional file 1: Table S7), which is comparable to the number of genes found in *Capitella teleta* (32 389) and *C. gigas* (28 027) but higher than that in *Drosophila melanogaster* (23 847), *Helobdella robusta* (23 400), and *Lottia gigantea* (23 800). Searches against public databases showed that 84.0% of the gene models matched known proteins (Additional file 1: Table S8). Further, BUSCO analysis shows that 82.8% of predicted genes are completed and 7.4% of them are fragmented, indicating that our assembly is adequate for further analysis. To assess the impact of selection, we determined codon usage, GC content of intron, exon, and inter-genic regions, and GC content at each codon position, which were similar in *P. f. martensii* and 8 other species (Additional file 4: Fig. S3).

Phylogenetic analysis of the sequenced genomes of *P. f. martensii, C. gigas*, and *L. gigantean*, along with selected model organisms, provided estimates of divergence times: 485 million years ago (mya) between *P. f. martensii* (Bivalvia) and *L. gigantea* (Gastropoda) and 316 mya between *P. f. martensii* (Pteriidae) and *C. gigas* (Ostreidae) (Additional file 5: Fig. S4). These estimates are in agreement with the most up-to-date phylogenetic analyses of molluscan evolution [12]. Compared to *Homo sapiens* and *Danio rerio*, molluscan genomes do not have transforming growth factor (TGF)–beta factors but only bone morphogenetic proteins (BMPs), and these 2 proteins share a common origin with TGF-beta being derived from BMPs (Additional file 1: Tables S10, S11, and S12; Additional file 6: Fig. S5). TGF-beta factors are crucial in regulating osteoblast proliferation, differentiation, and bone matrix maturation in vertebrates [13, 14]. This finding suggests that molluscs have maintained an ancient BMP regulatory system for shell formation [15], while TGF-beta emerged in vertebrates to regulate the bone matrix.

### Chitin is a basic component of the nacre matrix

Consistent with the matrix model of molluscan shell formation, we demonstrated the abundant presence of chitin in the shell matrix of *P. f. martensii* (in both prismatic and nacreous layers) and *C. gigas* (mostly prismatic) by Calcofluor white M2R staining (Additional file 7: Fig. S6a). Transcriptome analysis of different tissues indicated that some *chitin synthases* (*CHSs*) and *chitinase* were highly expressed in the mantle and pearl sac, the 2 main calcifying tissues responsible for shell and pearl formation (Additional file7: Fig. S6b and S6c). During larval development, some *CHSs* and *chitinases* were highly expressed at the trochophore and spat stages (Additional file 7: Fig. S6d), corresponding to prodissoconch and dissoconch/adult shell formation, respectively. Furthermore, the gene family of *CHS* was significantly expanded in *P. f. martensii* and other shelled molluscs, but not in molluscs without shells, such as *Octopus bimaculoides* (Additional file 1: Table S10). These results suggest that chitin is a key component of the shell matrix, and *CHS* genes in *P. f. martensii* and other shelled molluscs might have played crucial roles in the evolution of advanced shells in molluscs.

### The presence and involvement of VWA-containing proteins 

**Figure 2:**
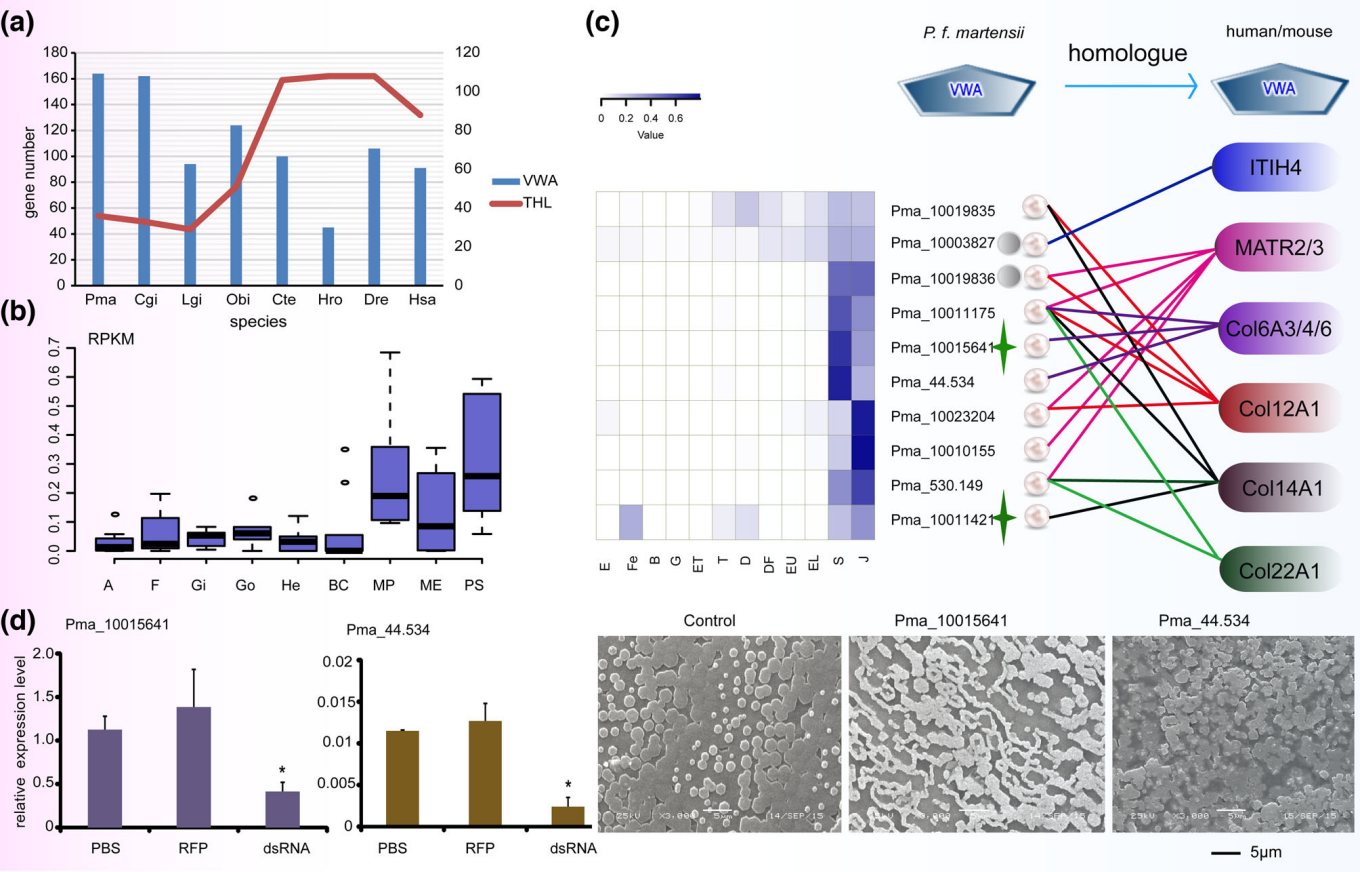
Expression and functional analysis of VWAPs in *P. f. martensii*. (**a**) Number of VWAPs and THR-containing proteins in different species. *P. f. martensii* (Pma), *C. gigas* (Cgi), *L. gigantea* (Lgi), *O. bimaculoides* (Obi), *C. teleta* (Cte), *H. robusta* (Hro), *D. rerio* (Dre), and *H. sapiens* (Hsa). (**b**) Expression of 10 genes encoding VWAPs from nacreous shell matrix showing higher expression in the MP and PS than in other organs. The y-axis is the normalized RPKM value. The x-axis lists 9 organs/tissues (MP: mantle pallium; ME: mantle edge; A: adductor muscle; He: hepatopancreas; BC: hemocyte; Go: gonad; Gi: gill; F: foot; PS: pearl sac at 180 days after nucleus transplantation). (**c**) Expression pattern of the 10 *VWAPs* during early development and the homology of their VWA domains to that from human and mouse proteins. E: egg; Fe: fertilized egg; B: blastula; G: gastrula; ET: early trochophore; T: trochophore; D: D-stage larvae; DF: D-stage larvae before feeding; EU: early umbo larvae; EL: eyed larvae; S: spat; J: juveniles. (**d**) Expression of *Pma_10015641* and *Pma_44.543* and nacre growth after RNA interference. Left: relative expression of *Pma_10015641* and *Pma_44.534* in mantle after RNAi; PBS: control; RPF: red fluorescent protein; dsRNA: RNAi. Right: Disordered microstructure of nacre observed after inhibition of the 2 *VWAP* genes (bar = 5 μm). Col: collagen; ITIH4: inter-alpha-trypsin inhibitor heavy chain H4; MATR: matrilin.

According to the current model, silk proteins are major components of the organic matrix in molluscan shells. We searched for silk proteins in the *P. f. martensii* genome and the proteome of the shell matrix but found none. Interestingly, a total of 10 VWA-containing proteins (VWAPs) were detected in the nacre proteome with 372 unique spectra, compared with 146 spectra in the prismatic layer proteome (Additional file 8: Datasets S1). Among the 10 VWAPs, 8 VWAPs specifically existed in the nacre and were not found in the prismatic layer proteome, and 8 VWAPs contained VWA domains that show highest-sequence homology with VWA domains of human or mouse collagens (Additional file 1: Table S13). Corresponding to the abundance of VWAPs in the nacre proteome, the *P. f. martensii* genome has an expanded family of 164 VWAPs, similar to the 162 found in *C. gigas* [6] but more than the 94 found in *L. gigantea* and 91 in humans (Fig. 2a). The 10 *VWAPs* were highly expressed in the mantle pallium and pearl sac, which are responsible for nacreous layer formation (Fig. 2b). All 10 *VWAPs* were up-regulated (at least ×5 of the level in egg) in spat with nacreous/aragonite shells, again suggesting their crucial role in nacreous construction. Meanwhile, 2 of the 10 *VWAPs* (Pma_10019835, Pma_10011421) were significantly up-regulated (40X of egg) at the trochophore stage, in correlation with aragonite shell formation (Fig. 2c). After inhibition of 6 *VWAPs* (Pma_44.534, Pma_530.149, Pma_10011175, Pma_10019835, Pma_10019836, Pma_10015641) by RNA interference, the microstructure of the nacre showed disordered growth, as observed by scanning electron microscopy (SEM) (Fig. 2d; Additional file 9: Fig. S7). These results suggest that VWAPs are a major component of the nacreous organic matrix and play a key role in nacreous shell formation in *P. f. martensii*. Structural analysis indicated that 2 of the 10 VWAPs (Pma_10015641 and Pma_10011421) have a chitin-binding domain, supporting their possible interaction with the chitin framework. Surprisingly, there are no collagens containing both VWA and triple helix repeat (THR) in genomes of the 3 molluscs analyzed (*P. f. martensii, C. gigas*, and *L. gigantea*). Collagens with VWA and THR are found in some invertebrates (*C. teleta, H. robusta* and *Mytilus coruscus* [16]) (Additional file 10: Fig. S8), suggesting that THRs may be lost in some lineages. THR-containing genes showed contraction in mollusc genomes compared with annelids and vertebrates (Fig. 2a).

### Acidic glycosaminoglycans constitute a gel-like substance

**Figure 3:**
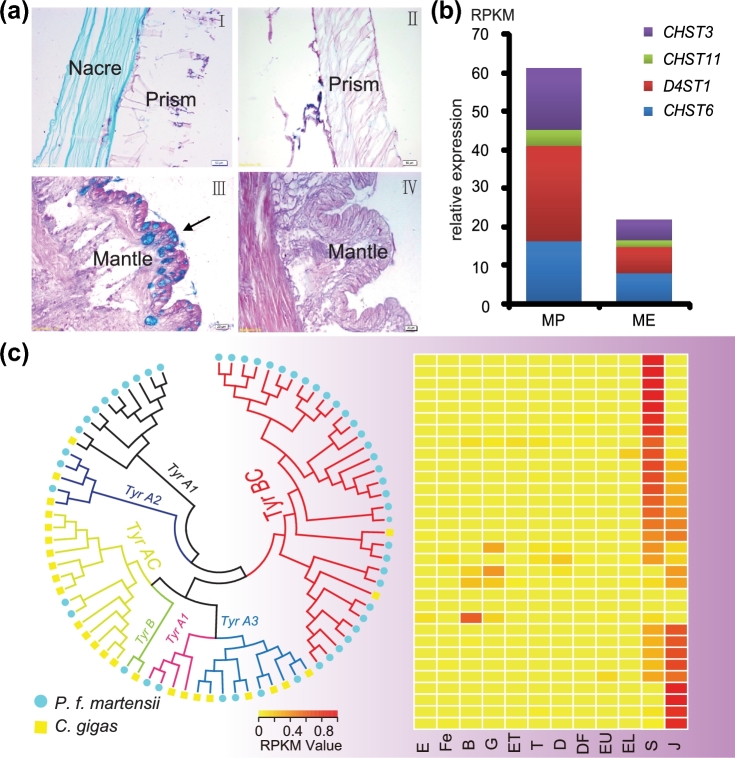
GAGs and tyrosinase genes in *P. f. martensii*. (**a**) The shell matrix extracted from the nacre of *P. f. martensii* contains abundant acid glycosaminoglycans stained blue (I), whereas matrices extracted from the prismatic layer of *P. f. martensii* (I) and *C. gigas* (II) contain neutral GAGs stained red. Secretory cells (arrow) in the mantle pallium of *P. f. martensii* are filled with acid GAGs stained blue (III), whereas cells in the mantle pallium of *C. gigas* contain neutral GAGs stained red (IV). (**b**) Expression (y-axis) of *CHST3, CHST11, CHST6*, and *D4ST1* in the mantle pallium and the mantle edge. (**c**) Phylogenetic tree of tyrosinase proteins from *P. f. martensii* and *C. gigas*. Tyrosinase genes specifically expanded in *P. f. martensii* are shaded in purple, and their expression patterns during early development are presented in the heat map. E: egg; Fe: fertilized egg; B: blastula; G: gastrula; ET: early trochophore; T: trochophore; D: D-stage larvae; DF: D-stage larvae before feeding; EU: early umbo larvae; EL: eyed larvae; S: spat; J: juveniles.

According to the matrix model, the shell matrix contains a gel-like substance where acidic proteins induce the nucleation of calcium carbonate crystals [17]. Consistent with the model and previous reports, we identified a list of acid proteins that might be involved in shell formation (Additional file 8: Datasets S2). In addition to the acidic proteins that are unique to molluscan shells, we also found acidic glycosaminoglycans (GAGs), fibronectin-like proteins, and chondroitin sulfotransferases that are characteristic components of vertebrate bone matrices. By Alcian blue-periodic acid Schiff staining (AB-PAS), we found that the organic matrix extracted from nacreous shells contained large amounts of acidic GAGs compared with mainly neutral GAGs in prismatic layers of *P. f. martensii* and *C. gigas* shells. In addition, we detected acid GAGs in secretory cells of the mantle pallium of *P. f. martensii*, but mainly neutral GAGs in the mantle of *C. gigas* (Fig. 3a). Further, our data show that the *P. f. martensii* genome has an expanded set of 5 types of sulfotransferases (Additional file 1: Table S10), including chondroitin 4-sulfotransferase 11 (CHST11), chondroitin 6-sulfotransferase 3 (CHST3), carbohydrate 6-sulfotransferase 6 (CHST6), carbohydrate 4-sulfotransferase 9 (CHST9), and dermatan 4-sulfotransferase 1 (D4ST1). Corresponding to large amounts of acidic GAGs in the mantle pallium, some of the sulfotransferases (*CHST3, CHST11, CHST6*, and *D4ST1*) exhibited higher expression levels in the mantle pallium (Fig. 3b). *CHST11* and *D4ST1* expressed at the spat stage, whereas *CHST6* and *CHST3* were mostly up-regulated at the trochophore stage (Additional file 11: Fig. S9a).

### Tyrosinase may participate in nacre matrix cross-linking

*C. gigas* has an expanded set of 26 *tyrosinases* (*Tyrs*) [6], and we observed an even larger expansion of *Tyrs* in *P. f. martensii* to 53 genes compared with 3 genes in *L. gigantea*, 1 in humans, and 4 in stony coral (Additional file 1: Table S10). Phylogenetic analysis of these *Tyrs* from *P. f. martensii* and *C. gigas* revealed unbalanced and lineage-specific expansion in both species (Fig. 3c; Additional file 1: Table S14). Their expression profiles in calcifying tissues and at shelled larval stages indicate that 29 of the expanded *P. f. martensii Tyrs* may be involved in shell formation, among which 23 were highly expressed after the spat/juvenile stage, pointing to possible functions in adult shell formation (Fig. 3c). Seven *Tyrs* highly expressed in the mantle pallium (MP) (Additional file 11: Fig. S9b) and 9 in the pearl sac (Additional file 11: Fig. S9c), compared with 13 in the mantle edge (ME). Twelve Tyrs were identified from shell proteome: 2 specific to the nacreous layer and highly expressed in MP, 4 specific to the prismatic layer and highly expressed in ME, and 6 found in both nacreous and prismatic layers. A greater abundance of quinoproteins was observed in the nacreous than in the prismatic layers (Additional file 11: Fig. S9d). These results indicate that dopaquinone production catalysed by Tyr may be essential for the assembly and maturation of both nacreous and prismatic shell matrices.

### Regulation network of the nacre matrix proteins

**Figure 4:**
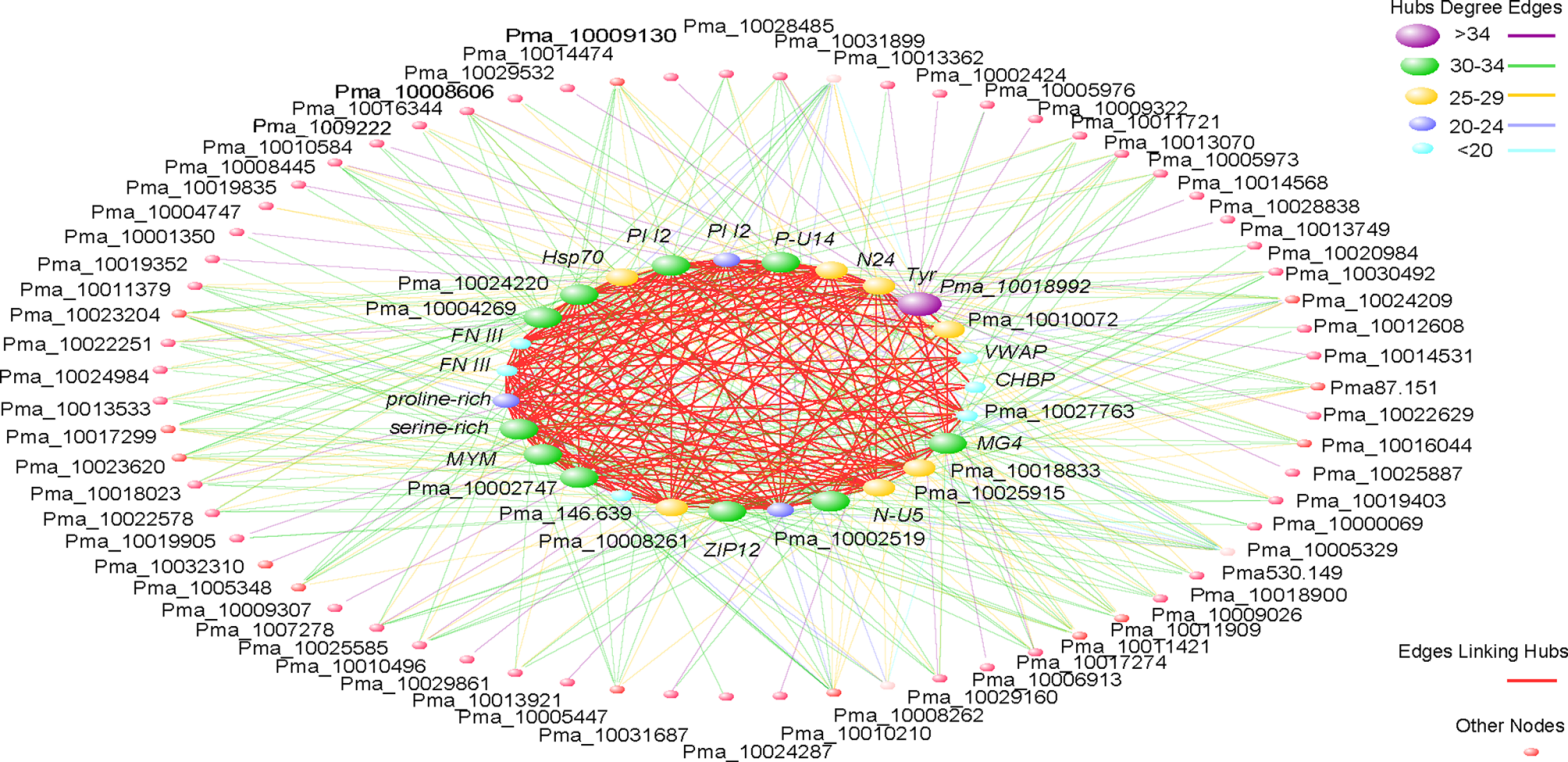
Co-expression network of nacre formation–related genes of *P. f. martensii*. Hub genes are illustrated in the internal circle, where connections among them are coloured red. The number of visible links for each hub gene is represented by the size of the node. Links and their corresponding hub genes are in the same colour.

WGCNA of the 234 nacre matrix protein genes revealed 27 genes at hubs of the network (Fig. 4; Additional file 8: Datasets S3 and S4), including well-known as well as novel genes for shell formation, such as *fibronectin III, VWAP*, and *Tyr*. In addition, heat shock protein 70 (Hsp70), proteinase inhibitor I2–containing proteins, and chitin-binding domain–containing proteins were also among the hub genes, indicating their possible roles in nacre formation. Furthermore, after filtering by the adjacent coefficient (no less than 0.5), we obtained 3245 crucial genes co-expressed with nacre matrix proteins. These co-expressed genes were significantly enriched (*P* < 0.05) in the ErbB, Jak-STAT, Wnt, osteoclast differentiation, vascular endothelial growth factor (VEGF) signalling pathways, and ECM-receptor interactions that are involved in bone formation (Additional file 1: Table S15). Genes related to the metabolism of a polysaccharide such as glycosaminoglycan, N-glycan, and O-glycan were also implicated. Analysis by gene ontology indicated that genes involved in transmembrane transporter activity were significantly enriched, which is consistent with the enrichment of ABC transporters in KEGG analysis (Additional file 1: Table S15, S16).

## Discussion

The assembly of highly polymorphic genomes and gene prediction in non-model organisms remain challenging. Software based on the *de Bruijn* graph, such as SOAP*denov*o [18], is inadequate in producing satisfactory results due to the increased complexity of the *de Bruijn* graph structure. Overlap-Layout-Consensus assemblers, such as Celera Assembler [19], that use data from fosmid or BAC hierarchical sequencing and third-generation long reads (such as PacBio) are employed to overcome such problems. However, the best choice for assembling complex genomes is to sample haploid or homozygous sequences. For *ab initio* gene prediction software, such AUGUSTUS [20], the aim is to find potential coding sequences with sufficiently long open reading frames, but the translated regions may be too short to determine the real absence of stop codons. Similarity-based approaches including homologous protein sequences, EST sequences, and transcripts assembled from RNA-seq reads can produce biologically relevant predictions, but they may not cover all coding exons. Considering their strengths and weaknesses, synthesis software, such as GLEAN [21] and MAKER [22], were used to synthesize the evidence obtained from *ab initio* gene predictions and similarity-based approaches into the final gene annotation. BUSCO [23] analysis indicates that our assembly is sufficiently complete.

Nacre's molecular composition and mechanism of formation are the target of many studies and modelling. According to the matrix model of molluscan shell formation, the mineralization of calcium carbonate is directed by a mantle-secreted organic matrix [24, 25], which is not fully understood but may contain chitin [26–28] and silk fibroin [29–31] for the structural framework and soluble acidic proteins for crystal nucleation [32–34]. Alternatively, the cellular hypothesis argues that biomineralization may be directed by hemocytes [7, 35] although there is no dispute about the involvement of organic matrices, which are the focus of our study. Chitin is an ancient macromolecule and the primary framework component of organic matrices in cell walls of fungi and diatoms, sponge skeletons, and arthropod shells [3]. It is possible that the chitin components of lophotrochozoan and ecdysozoan shells and of sponge skeletons constitute a shared feature and have the same ancient origin. Our results provide strong evidence that chitin is the basic component of *P. f. martensii* shell matrices.

While silk proteins, which are also considered the major components of the organic matrix in molluscan shells, were not found in the *P. f. martensii* genome or the proteome of the nacreous shell matrix, abundant signatures of expanded VWAPs were detected in the nacre proteome. VWAPs were also found in *C. gigas, Mytilus edulis*, and *Pecten maximus* [36]. VWA domains are a family of 200-amino-acid residues and function as interaction modules in many proteins, such as copines, integrins, von Willebrand factor, complement factors B and C2, matrilins, and collagens [37]. Collagens are a large family of extracellular matrix proteins with typical THRs. Eight of the 28 known collagens (collagen VI, VII, XII, XIV, XX, XXI, XXII, and XXVIII) contain VWA domains in addition to THRs. The finding that VWAs of VWAPs from the shell matrix show the highest homology with VWAs of vertebrate collagens suggests that these VWAPs and vertebrate collagens may have a common origin. It is possible that collagens with VWAs have evolved from VWAPs through the addition of THRs and VWAPs of *P. f. martensii* represent an ancient form that never acquired THRs. It is also possible and that some of the VWAPs were collagens that lost THRs. The existence of collagens with both VWAs and THRs in some invertebrates, such as *C. teleta, H. robusta*, and *Mytilus coruscus* [16] but not *P. f. martensii, C. gigas*, and *L. gigantea*, argues for the loss of THRs in some molluscan lineages.

THRs are crucial for the self-assembly of collagen subunits into triple-helix protomers and the formation of fibrillar collagens [38, 39]. The absence of THRs in VWAPs indicates that VWAPs may function differently in the nacreous shells of *P. f. martensii* from collagens in bone; they may not self-assemble into fibrous structures but cross-link with each other or with other matrix proteins to form a network structure [40–42]. The finding of VWAPs with chitin-binding domains further highlights their function in interacting with the chitin framework during matrix formation. In addition, VWA domains bind to positive ions that attract water, and they may cooperate with GAGs or other proteins and provide initial hydrogel properties for biomineralization [37].

Mammalian cartilage and bone matrices consist of collagen fibrils and a gel-like ground substance that is rich in chondroitin-containing proteoglycans, fibronectins, and link proteins [43]. Our results confirm the presence of fibronectin-like proteins in the shells of *P. f. martensii* and *C. gigas* [6]. Proteoglycans or GAGs, which have strong water-binding capabilities and have been detected in shells [29], may function as the gel-like substance [44]. In the nacre and the secretory cells of the mantle pallium of *P. f. martensii*, we found large amounts of acidic GAGs, which have also been detected in coral [45] and bone [46]. This finding argues that the acidic GAGs may also play a key role in crystal nucleation during nacre formation. Combining this finding with the finding of collagen-related VWAPs and other elements shared by bone formation, our results indicate that the nacreous matrix, while having a chitin-based framework, also possesses key elements of collagen-based matrices, such as fibronectins, proteoglycans, and chondroitin sulfotransferases. Chitin- and collagen-based matrices are considered 2 basic types of biomineralizing framework with independent origins. The finding of elements of both types in nacreous shells suggests that chitin- and collagen-based matrices may have a common origin or might have co-existed as parts of an ancient/ancestral matrix with dual elements, despite subsequent divergence in different taxa into chitin- or collagen-based organic matrices.

The shell matrix, rather than being a simple self-assembling structure, may instead be a complex and dynamic matrix that requires active construction, regulation, and remodelling. Tyrs, which can catalyse the formation of dopa and dopaquinone, are highly abundant in bivalve shells and may function in mediating intermolecular cross-links [6, 47] or as a structural component of the shells. Tyrs belong to the “type-3 copper” family and have a conserved active site of 6 histidine residues that facilitate the binding of copper ion as a cofactor [48]. Metal ions such as Cu^2+^, Zn^2+^, and Mg^2+^ are important factors for stabilizing the crystalline form of calcium carbonate [49–51]. Therefore, the deposition of Tyrs and associated metal ions in the matrix may regulate metal ion concentration in the extrapallial fluid and help to stabilize calcium carbonate crystals. Interestingly, we found that the histidine residues were retained in the 4 prism-specific Tyrs but mostly lost in the 2 nacre-specific Tyrs (Pma_10005159 and Pma_10016044), suggesting possible divergence in metal ion binding capability between nacre-specific and prism-specific Tyrs. It should be noted that many of the expanded *Tyrs* may be unrelated to shell formation as shell-less *Octopus bimaculoides* also shows some expansion (Table S10), and instead they may function in their well-established roles in melanin pigment production, wound healing, and immune responses in *P. f. martensii* also [52].

The abundance of chitin and GAGs in the nacreous layer is consistent with the results of co-expression network analysis, that genes related to polysaccharide metabolism are co-expressed with nacre proteins. Interestingly, ABC-transporters, also known as ATP-dependent transport proteins, are also co-expressed with nacre proteins. These ABC-transporters may mediate the secretion of matrix proteins without signal peptides [53], which are not uncommon among nacre proteins and may be also secreted through other mechanisms such as exosomes [6]. Some nacre proteins without signal peptides may be due to assembly and annotation errors. More importantly, signal pathways related to bone formation, such as the Wnt signalling pathway and osteoclast differentiation signalling pathway, are also implicated. Together, these results suggest that molluscan shell formation is an elaborate and dynamic process that shares certain basic elements with mammalian bone formation, and with added complexity. Although molluscan shells have a chitin-dominated framework, the identification of key elements shared by collagen-based matrices supports a single origin for the 2 types of matrices or a common set of tools that may have been lost, modified, and reorganized during evolution to produce diverse biomineralized structures in adaptating to changing environments or in assuming new functions.

**Figure 5:**
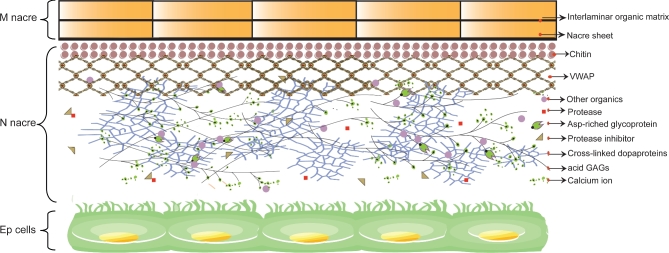
A model of nacre formation in *P. f. martensii*. In this model, new nacre (N) is formed in an organic matrix secreted by haemocytes or epithelial (Ep) cells beneath the mature nacre (M). Chitin provides the core of the polymer framework of the organic matrix. VWAP with chitin-binding domains binds to chitin and interacts with fibronectins and other VWAPs, forming the matrix networks. Asp-rich acid glycoproteins and acid GAGs function as the hydrogel substances. *Tyrs* catalyse the oxidation of tyrosine and dopamine and function in cross-linking and shell matrix maturation. Protease inhibitors, proteases, and other enzymes regulate the biosynthesis or degradation of the organic matrix.

In conclusion, we sequenced and assembled the highly polymorphic genome of *P. f. martensii* using NGS and the BAC-to-BAC strategy. Genomic, transcriptomic, and proteomic analyses plus experimental studies allowed the identification of a large number of genes related to shell formation and the reconstruction of the model for the nacre matrix (Fig. 5). The identification of collagen-related VWAPs and other elements of collagen-based matrices in the chitin-rich nacre matrix supports the homology and single evolutionary origin of the common biomineralization toolkit. The hypothesis of a single evolutionary origin challenges the prevailing idea of independent evolution [2] and may stimulate homology-based studies towards a better understanding of the diverse forms of biomineralization.

**Figure:**
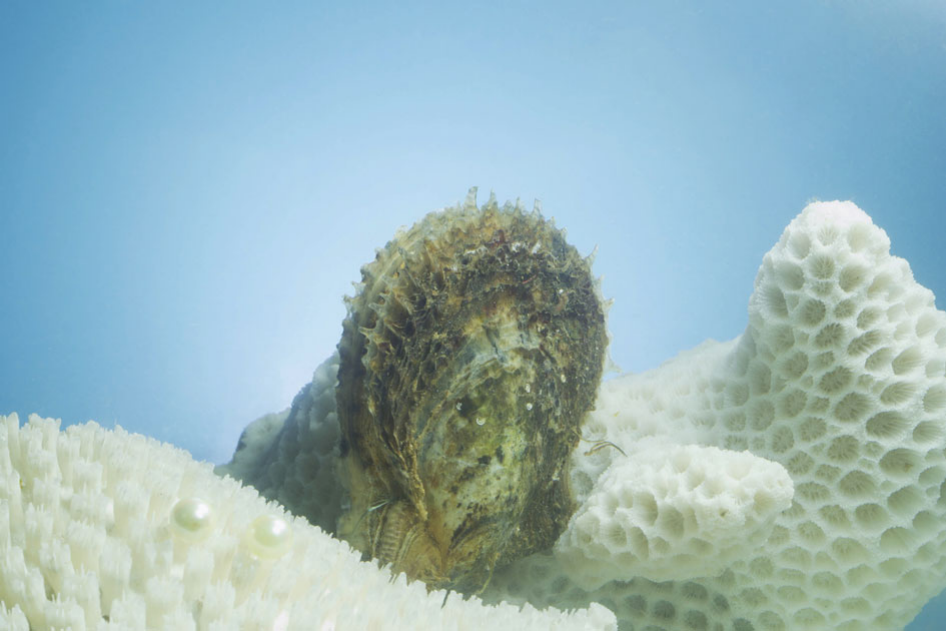


## Methods

The SI Appendix has additional information relating to the methodologies described below.

### Library construction and sequencing

We constructed all sequencing libraries according to protocols from Illumina and sequenced these libraries on a HiSeq 2000 sequencing system.

### Hierarchical BAC-to-BAC assembly strategy

We used a hierarchical BAC-to-BAC assembly approach, as used for the moth genome [11]. Before the hierarchical assembly of BACs, we used SOAPdenovo (SOAPdenovo, RRID:SCR_010752) to assemble the reads of each BAC with odd numbered K-mers from 27 to 63 and selected the best results with the longest scaffold N50 and total length as primary scaffolds. Then, we used paired-end reads of the BACs and locally assembled the reads in the gap regions to fill in the gaps within primary BAC scaffolds. Our custom assembly software (Rabbit) [11] was used to assemble scaffolds of BACs with large overlaps. After finding the relationship among sequences, merging overlapping sequences, and removing redundant sequences, we obtained longer segments as secondary scaffolds. Finally, SSPACE (SSPACE, RRID:SCR_005056) was used to join the secondary scaffolds to form final scaffolds, and SOAP-Gapcloser (GapCloser, RRID:SCR_015026) was used to fill in the gaps in the final scaffolds using all WGS reads with short insert sizes.

### Linkage group construction

We constructed a genetic map using RAD-seq of 148 F1 progeny from a family obtained by crossing 2 genetically distant parents. We used SOAP2 (SOAP2, RRID:SCR_005503) [54] to map the reads to the reference genome sequences of *P. f. martensii* (scaffolds) and performed SNP calling using SOAPsnp (SOAPsnp, RRID:SCR_010602) [55]. After SNP calling, we extracted genotypes by combining all SNPs among the 148 progeny and the 2 parents and constructed a linkage map using JoinMap 4.1 (JOINMAP, RRID:SCR_009248) [56].

### Phylogenetic tree construction and divergence time estimation

We used Treefam (Tree families database, RRID:SCR_013401) to obtain gene families and one-to-one orthologs and used MrBayes to construct the phylogenetic tree.

### Transcriptome analysis

We extracted total RNA from each sample and isolated mRNA using oligo (dT) magnetic beads. Then, the mRNA was fragmented into short fragments (200∼500 bp) for construction of RNA-seq libraries that were sequenced on Illumina HiSeq2000. Using SOAP2, all clean reads were mapped to the genome assembly with less than 5 mismatches. We used the reads per kilobase transcript per million mapped reads (RPKM) method to calculate the gene expression levels. We also tested transcripts per million (TPM) [57] for quantifying gene expression and found excellent correspondence between RPKM and TRM for our samples.

### Identification of the matrix proteins

We used Mascot software v. 2.3.02 (Mascot, RRID:SCR_014322) to query the MS/MS spectra data of matrix proteins in the database. We applied the trypsin cleavage rule with 1 missed cleavage site. Carbamidomethylation of cysteines was considered a fixed modification while Gln→pyro-Glu (N-term Q), oxidation (M), and deamidated (NQ) were considered variable modifications. Peptide mass tolerance was set to 0.05Da, and fragment mass tolerance was set to 0.01Da. We used a target-decoy search strategy [58] to identify matrix proteins with a false discovery rate (PDR) of ≤1%.

### Extraction of matrix proteins from the nacre and prismatic layer

Shells of freshly collected oysters were thoroughly cleaned by hand and treated with sodium hypochlorite solution (6–14% active chlorine) to remove organic surface contaminants [59]. The prismatic layer was separated from the edges of pearl oyster shells without nacre. The nacre was directly scraped from the internal shell surfaces dominated by aragonite. These samples were thoroughly ground and soaked in acetic acid solution (5%, v/v) for at least 12 hours to dissolve calcium carbonate, before being centrifuged at 14 000 g and 4°C for 1 hour. Acid-soluble proteins were in the supernatant, and acid-insoluble proteins were in the residue.

Samples were electrophoresed on 12% polyacrylamide gels and stained with Coomassie blue R-250. The extracted peptides were dried and stored at –80°C until liquid chromatography/tandem mass spectrometry (LC-MS/MS) analysis.

### Chitin identification in shell matrix

We decalcified the shells in 1 M acetic acid at 4°C for 1 week, and the acid-insoluble material was collected. This insoluble material was washed with distilled water and embedded in paraffin for sectioning. The sections were placed on slides and stained for 5 minutes with 0.1% Calcofluor White M2R (Flupstain I; Sigma-Aldrich). Excess dye was rinsed off with distilled water. The stained specimens were observed under a confocal laser microscope using filters with 492 nm excitation and 520 nm emission [60].

### RNAi experiment

The primers used for generating double-strand RNA (dsRNA) of *VWAPs* are shown in Additional file 1: Table S17. DsRNAs were synthesized following the method of Suzuki et al. [61] and injected into the adductor muscle every 4 days at 100 μg per 100 μl per pearl oyster each time. The effects of RNAi of the 6 *VWAPs* on nacre formation were detected by SEM.

### Identification of GAGs in shells and pearls

Shells and pearls were decalcified in 1 M acetic acid at 4°C for 1 week and then in 10% EDTA-2Na solution at room temperature for 10 days. The fixed materials were embedded in paraffin and stained with Alcian blue/periodic acid-Schiff and observed under an Olympus BX51 optical microscope.

### Nitrobluetetrazolium/glycinate assay for dopa and dopaquione protein

Sections of decalcified shells were stained with 100 μL of solution containing 0.24 mM nitrobluetetrazolium (NBT) and 2 M potassium glycinate (pH10) for nearly 5 minutes in darkness until violet positive signals appeared [62]. The sections were rinsed with double-distilled water to stop the reaction and then mounted for microscopic examination.

### Co-expression network analysis

We used Weighted Gene Co-expression Network Analysis (WGCNA, RRID:SCR_003302) to reconstruct the co-expression network for biomineralization [63]. A weighted correlation network was constructed between all pairs of genes across 4 mantle tissue samples. The adjacency matrix was calculated through a so-called “soft” thresholding framework (power β = 9) that converted the co-expression measure to a connection weight. Based on the adjacency matrix, we implemented a topological overlap dissimilarity measure to reflect relative inter-connectedness, which may represent a meaningful biological network. Hub genes (highly connected genes), by definition, tend to have high connectivity in the constructed network.

## Availability of supporting data

Data from the pearl oyster (*P. f. martensii*) genome projects are available from NCBI BioProject: PRJNA283019. The *P. f. martensii* shell matrix protein LC-MSMS project has been submitted to the PRIDE database (accession PXD006786). Data supporting the manuscript, including sequence assembly and annotation data, BUSCO results, phylogeny, and SEM and LC-MSMS data, are also available via the *Giga*DB database [64].

## Additional files

Additional file 1: Methods and related tables.

Additional file 2: Figure S1. Sequencing date and k-mer analysis. (a) The distribution of 17-mer depth derived from WGS sequence reads. The x-axis is the K-mer depth, and the y-axis is the percentage of each K-mer depth. The first peak is created by sequence polymorphism, and its relative height provides a measure of heterozygosity in the diploid genome. (b) The heterozygous ratio of the oyster genome estimated by k-mer analysis (left). The sequencing depth obtained by WGS reads mapped against assembly and GC content of our genome (right). (c) The assembled length of the BACs of 4 pooling libraries. Four libraries were randomly selected, and the total length of each assembly was calculated.

Additional file 3: Figure S2. Assembly coverage of BACs. Sequencing depth of BACs was calculated by mapped sequence reads. The annotated transposable elements (TEs) are shown in black or red, and the remaining unclosed gaps on the scaffolds are marked as white blocks.

Additional file 4: Figure S3. Codon usage and GC content analyses. (a) The distribution of codon usage among 9 species. ACAL: *Aplysia californica*; CGIG: *C. gigas*; CTEL: *C. teleta*; DRER: *D. rerio*; HROB: *H. robusta*; HSAP: *H. sapiens*; LGIG: *L. gigantea*; OBIM: *O. bimaculoides*; PMAR: *P. f. martensii*. (b) The GC content for each codon position. (c) The GC content of exon, intron, and inter-genetic regions.

Additional file 5: Figure S4. Phylogenetic analysis and gene clustering. (a) Species tree of *P. f. martensii* and 6 selected species. The numbers are the divergence time of the clades, with ranges in parentheses. (b) Unique and shared gene families between *P. f. martensii* (*P. mar*) and other 3 species, including *Crassostrea gigas* (*C. gig*), *Lottia gigantea* (*L. gig*), and *Homo sapiens* (*H. sap*).

Additional file 6: Figure S5. Phylogenetic analysis of TGF-β1/2/3 and bone morphogenetic proteins (BMP) from different species. Proteins and accession numbers are listed in *SI Appendix*, Table S12.

Additional file 7: Figure S6. CHS and chitinase genes in *P. f. martensii*. (a) Chitin in the shell matrix of *P. f. martensii* and *C. gigas* stained green with Calcofluor White M2R. (b) Expression of *CHS* in different organs. One *CHS* (Pma_10008435) is highly expressed in both the mantle pallium and the pearl sac. (c) Expression of *chitinase* in mantle pallium (MP), mantle edge (ME), and pearl sac (PS), compared with non-calcifying tissues (A: adductor muscle; He: hepatopancreas; BC: hemocyte; Go: gonad; Gi: gill; F: foot). (d) Expression of *Chitinases* and *CHS* at different developmental stages of *P. f. martensii*. Most of the *chitinases* are highly expressed at T and S stages. The expression of 1 *CHS* (*Pma_10008435*), which is highly expressed both in mantle pallium and pearl sac, is also induced at T and S stages. E: egg; Fe: fertilization; B: blastula; G: gastrula; ET: early trochophore; T: trochophore; D: D-stage larvae; DF: D-stage larvae before feeding; EU: early umbo larvae; EL: eyed larvae; S: spat; J: juveniles.

Additional file 9: Figure S7. RNAi analysis of 4 *VWAPs* in *P. f. martensii.* Suppression of 4 *VWAPs* with RNAi. Expression profiles of 4 *VWAP* genes in the mantle, Pma_530.149, Pma_10019835, Pma_10019836, and Pma_1011175, were determined using real-time quantitative polymerase chain reaction, with GAPDH as the internal reference gene. *VWAPs* were significantly inhibited in the treatment group (*P* < 0.05). SEM images of the surface of the nacre from *P. f. martensii* injected with PBS and 100 μg red fluorescent protein dsRNA demonstrated a normal growth of nacre, whereas *P. f. martensii* in the treatment group injected with Pma_530.149, Pma_10019835, Pma_10019836, and Pma_10011175 dsRNA showed disruptions in crystal growth.

Additional file 10: Figure S8. Domain structure of collagens containing both VWA and THR in *C. teleta* (Cte), *H. robusta* (Hro), and *M. coruscus* (Mco). Accession numbers: (a) ELU11155.1; (b) ELT92434.1; (c) WP_021368082.1; (d) XP_009018142.1; (e) XP_009024759.1; (f) ALA16011.1.

Additional file 11: Figure S9. Tyrosinases and sulfotransferases in *P. f. martensii.* (a) Expression of *sulfotransferase* in early development. *CHST11* (Pma_133.4) and *D4ST1* (Pma_10006752) showed expression at the S stage, whereas *CHST6* (Pma_279.110) and *CHST3* (Pma_10022575) were mostly up-regulated at the T stage. E: egg; Fe: fertilized egg; B: blastula; G: gastrula; ET: early trochophore; T: trochophore; D: D-stage larvae; DF: D-stage larvae before feeding; EU: early umbo larvae; EL: eyed larvae; S: spat; J: juveniles. (b, c) *Tyr* expression in the mantle and pearl sac, respectively, compared with other non-calcifying tissues (including A, adductor muscle; He: hepatopancreas; BC: hemocyte; Go: gonad; Gi: gill; F: foot). *Tyrs* that were highly expressed in mantle pallium or mantle edge are shown in (b); the different cycles represent different *Tyrs* (inside-out: Pma_10005159, Pma_10013533, Pma_10015392, Pma_10016044, Pma_10021421, Pma_10021422, Pma_10022578, Pma_10001525, Pma_10004452, Pma_10005803, Pma_10013532, Pma_10014430, Pma_10015306, Pma_10018719, Pma_10018775, Pma_10021425, Pma_10024726, Pma_10028201, Pma_10028307, Pma_10028311). Expression of *Tyrs* in pearl sac compared with other non-calcifying tissues is presented in (c), with 9 *Tyrs* highly expressed in PS marked with a red frame. (d) Abundance of quinoproteins (stained purple) in the nacre matrix revealed by an NBT/glycinate assay. Triangles designate the prismatic layer, and arrows designate the nacreous layer.

## Abbreviations

BMPs: bone morphogenetic proteins; CHS: chitin synthases; CHST11: chondroitin 4-sulfotransferase 11; CHST3: chondroitin 6-sulfotransferase 3; CHST6: carbohydrate 6-sulfotransferase 6; CHST9: carbohydrate 4-sulfotransferase 9; Col12A1: collagen alpha-1(XII) chain; Col14A1: collagen alpha-1(XIV) chain; Col22A1: collagen alpha-1(XXII) chain; Col6A3/4/6: collagen alpha-3/4/6(VI) chain; D4ST1: dermatan 4-sulfotransferase 1; ITIH4: inter alpha-trypsin inhibitor, heavy chain 4; MATN2/3: matrilin-2/3; Tyr: tyrosinase; VWA: von Willebrand factor A; VWAP: VWA domain containing protein; WGCNA: weighted-gene co-expression network analysis.

## Competing interests

The authors declare that they have no competing interests.

## Funding

This research is partly supported the Guangdong Ocean University Nature Science Foundation (University program: Genome Studies of Pearl Oyster), the National Nature Science Foundation of China (31 272 635, 31 372 526, 31 672 626), Modern Agro-industry Technology Research System (CARS-48), USDA/NJAES Project 1004475/NJ32920, and the “Taishan Oversea Scholar” program.

## Author contributions

X.D. conceived the study. X.D., X.G., G.Z., and X.L. designed scientific objectives. X.D. and X.G. directed final data analysis and interpretation. G.F. and H.Z. designed sequencing strategies and directed and contributed the most to genome sequencing and assembly. Y.J. (designer and leader), J.L., and Z.W. conducted studies on chitin and VWAPs and performed data analysis. R.H. (designer and leader), C.B., and H.L. conducted studies on tyrosinases and performed data analysis. Z.Z. (designer and leader), Q.W., and H.Z. conducted studies on GAGs and performed data and WGCNA analyses. F.S., X.L., C.S., W.L., and X.X. participated in genome assembly, gene annotation, and evolution analyses. Y.D., J.B., Q.S., W.C., and X.Z. constructed the genetic map. J.L. and R.H. conducted the RNAi experiments. Y.J., X.G., and Z.X. directed critical revisions of intellectual content. X.D., X.G., X.L., and G.Z. supervised all aspects of the work to ensure the accuracy or integrity of the research and data. All authors contributed to writing and revision and approved the submission.

## Supplementary Material

GIGA-D-16-00075_Original-Submission.pdf

GIGA-D-16-00075_Revision-1.pdf

GIGA-D-16-00075_Revision-2.pdf

GIGA-D-16-00075_Revision-3.pdf

GIGA-D-16-00075_Revision-4.pdf

Response-to-Reviewer-Comments_Original-Submission.pdf

Response-to-Reviewer-Comments_Revision-1.pdf

Response-to-Reviewer-Comments_Revision-2.pdf

Response-to-Reviewer-Comments_Revision-3.pdf

Reviewer-1-Report-(Original-Submission).pdf

Reviewer-1-Report-(Revision-1).pdf

Reviewer-2-Report-(Original-Submission).pdf

Reviewer-2-Report-(Revision-1).pdf

Reviewer-2-Report-(Revision-2).pdf

Reviewer-3-Report-(Original-Submission).pdf

Additional Files
