# Supplementary material for: The pearl oyster Pinctada fucata martensii genome and multi-omic analyses provide insights into biomineralization
Source: Gigascience. 2017 Jul 25;6(8):1–12. doi: 10.1093/gigascience/gix059 (PMC5597905; doi:10.1093/gigascience/gix059)
Supplement: GIGA-D-16-00075_Original-Submission.pdf [file gix059_GIGA-D-16-00075_Original-Submission.pdf]

Abstract

Background: Nacre, the iridescent material found in pearls and shells of molluscs, is formed through an extraordinary process of matrix-assisted biomineralization. The pearl oyster *Pinctada fucata martensii* is a well-known master of biomineralization, but the process it uses to produce remarkable nacre remain elusive and the origin and homology of nacre formation with other biomineralization processes is not fully understood.

Results: We sequenced the highly polymorphic genome of the pearl oyster *P. fucata martensii* and conducted multi-omic and biochemical studies to probe nacre formation. We identified a large set of novel genes participating in matrix-framework formation, including many in expanded families. Our analysis reveals that chitin, collagen VI, fibronectin and chondroitin sulfotransferases are key elements in nacre matrix-framework.

Conclusions: Considering that there are only collagen-based matrixes in vertebrate bones and chitin-based matrixes in most invertebrate skeletons, the presence of both chitin and collagen VI in nacre matrixes suggests that elements of chitin- and collagen-based matrixes are deeply rooted and might be part of an ancient biomineralizing matrix. Our results expand the current shell matrix-framework model of biomineralization and provide new insights into the evolution of diverse biomineralization systems.

Keywords: genome, biomineralization, nacre, collagen, *Pinctada fucata martensii*.

Background

Biomineralization is an extraordinary process where minerals form not following rules of inorganic chemistry but through active biological facilitation and control. Biomineralization is widely distributed and essential to the lives of diverse organisms, ranging from algae to vertebrates that rely on mineralized hard structures for morphology, structure, protection, movement and feeding. Three principal classes of skeletal biominerals exist on earth: calcium carbonate, calcium phosphate and silica [1]. Whether these skeletal biominerals evolved independently or derived from a

common origins is controversial, although current thinking favours independent evolution [2]. One of the remarkable characteristics of biominerals is their precise control by organic matrixes [3]. Organic matrixes are complex and variable but can be classified into two highly conserved types that use either chitin or collagen as the templating framework [3]. Despite great interest in harnessing the power of biomineralization for the production of novel materials, our understanding of biomineralization and associated matrixes is limited in many taxa, including the well-known masters of biomineralization - shelled molluscs.

Nacre is the remarkable biomineral found in the pearls and shells of molluscs that provides lustre and enhanced toughness. The formation of lustrous pearls and shells in molluscs such as the pearl oyster *Pinctada fucata martensii* has long fascinated humans. The biomineralization process of nacre formation is complex and involves sophisticated organic matrixes as well as cells, many aspects of which remain elusive [4-8]. The origin and homology of nacre formation with other biomineralization processes such as crustacean shell and vertebrate bone formation is not understood [9]. Studies of biomineralization and other fundamental questions in biology and evolution can be greatly empowered by whole genome analyses, which have been difficult in molluscs owing to challenges in assembling their highly polymorphic and complex genomes [6, 10]. To understand the biomineralization process of nacre, we sequenced and assembled the *P. fucata martensii* genome and generated transcriptomes from 11 organs/tissues and 12 developmental stages, along with the proteomes of shell organic matrixes.

Data description

We generated pearl oyster tissues from the third generation line selected for fast growth and decided to obtain a fine assembly using BAC-to-BAC assembly strategy. Along with the libraries construction and sequencing of BAC clones, we also constructed the whole genome shotgun (WGS) libraries including 3 short insert-size and 4 long insert-size libraries to support the assembly. To assess our assembly integrity, we adopted three level examinations including the draft assembly of previous study, Sanger-sequenced BACs and the transcripts generated by RNA-seq data. Furthermore, we constructed a genetic map using restriction association site DNA technology (RAD-seq) for *P. fucata martensii* to anchor our scaffolds to chromosomes. Total 148 F1 offspring were obtained by crossing two relatively distant

parents. Raw sequencing data for each individual could be separated by identifying the index sequences of pooling technology.

To illustrate the differentiation of gene expression profile in different organic tissues, we performed transcriptome sequencing on nine tissue samples, including adductor muscle, mantle pallium, mantle edge, hepatopancreas, hemocyte, gonad, gill, foot, along with pearl sac at days 180 after nucleus transplantation. Meanwhile, we performed transcriptome sequencing on 12 developmental samples to study the mechanism underlying *P. fucata martensii* development. Developmental samples included an unfertilized egg sample, 11 samples obtained at 30 min, 5 h, 6 h, 8 h, 16 h, 19 h, 4 d, 14 d, 28 d, 40 d, and 90 d after fertilization, respectively. To further reveal the hub genes in nacre formation, we analyzed the transcriptome data of four mantle tissue samples including mantle edge, mantle pallial and two entire mantle tissues representing the higher and lower growth of pearl oysters by WGCNA analysis, and obtained the co-expression pattern. All genome data were uploaded to GigaDB under the accession number XX.

Results

Genome assembly and characterization

As our initial assembly of ~130 Gb (134-fold coverage) of whole-genome shotgun (WGS) Illumina sequences (Additional file 1: Table S1) was too fragmented for annotation and analysis, probably due to high polymorphism and repetitive sequences (Additional file 2: Figure S1a and b), we subsequently adopted a BAC-to-BAC (bacterial artificial chromosome) sequencing strategy [6, 11]. We sequenced 46,080 BACs (5×fold genome coverage) to a depth of 100X using Illumina next-generation sequencing (NGS) and assembled each BAC separately (Additional file 2: Figure S1c), then built the supercontigs after merging and filtering redundant sequences. After constructing scaffolds and filling gaps with WGS reads, we obtained a final assembly of 990,658,107 bp with a contig N50 size of 21 kb and a scaffold N50 of 324 kb (Additional file 1: Table S2), which was a significant improvement compared with the contig N50 of 1.6 kb of the previous draft assembly [10].

The coverage of our assembly was demonstrated by the successful mapping of 90.5% of contigs, 95.5% (coverage ≥50%) of gene-model regions of the previous draft assembly [10], 99.8% of transcripts (coverage ≥50%), and all four BACs (coverage ≥93.2%) sequenced with Sanger sequencing (Additional file 1: Table S3 and S4;

Additional file 3: Figure S2). We constructed a high-density genetic map of 14 linkage groups in accordance with the haploid number, using restriction-site associated DNA sequencing (RAD-seq) of a full-sib family (Additional file 4: Figure S3a). We were able to anchor 857.07 Mb (86.5%) scaffolds to the genetic map with 4,463 single-nucleotide polymorphisms (SNPs) (Additional file 1: Table S5; Additional file 4: Figure S3b,). Through alignment of our pseudochromosome to *Crassostrea gigas*, we identified 2,240 syntenic blocks and several possible pseudochromosome rearrangements (Additional file 4: Figure S3c).

Combining *de novo* prediction and evidence-based annotation using published data and transcriptomes from 11 tissues and 12 developmental stages (Additional file 1: Table S6), we identified 32,937 protein-coding gene models (Additional file 1: Table S7), which was comparable to the gene numbers found in *Capitella teleta* (32,389) and *C. gigas* (28,027) but higher than those in *Drosophila melanogaster* (23,847), *Helobdella robusta* (23,400) and *Lottia gigantea* (23,800). Searches against public databases showed that 84.0% of the gene models matched known proteins (Additional file 1: Table S8). To further validate the predicted gene models, we performed eukaryotic orthologous group (KOG) analysis using the core eukaryotic gene mapping approach (CEGMA). A total of 452 of the 458 (98.7%) KOGs could be found in the *P. fucata martensii* gene-set. Repetitive sequences accounted for 48.5% of the genome (Additional file 1: Table S9), which was considerably higher than that in *C. teleta* (31.1%), *H. robusta* (33.4%), *L. gigantea* (20.8%) and *C. gigas* (36.0%). Most of the repetitive sequences were novel and potentially important in genome biology and divergence.

Phylogenetic analysis of the sequenced genomes of *P. fucata martensii*, *C. gigas* and *L. gigantea* along with selected model organisms provided estimates of divergence times: 485 million years ago (mya) between *P. fucata martensii* (Bivalvia) and *L. gigantea* (Gastropoda) and 316 mya between *P. fucata martensii* (Pteriidae) and *C. gigas* (Ostreidae) (Additional file 5: Figure S4). Compared to *Homo sapiens* and *Danio rerio*, molluscan genomes do not have transforming growth factor (TGF)-beta factors but only bone morphogenetic proteins (BMPs), but these two proteins share a common origin with TGF-beta being derived from BMPs (Additional file 1: Table S10, S11 and S12; Additional file 6: Figure S5,). TGF-beta factors are crucial in regulating osteoblast proliferation, differentiation and bone matrix maturation in vertebrates [12, 13]. This finding suggests that molluscs have

maintained an ancient BMP-regulatory system for shell formation [14], while TGF-beta emerged in vertebrates to regulate bone matrix.

Chitin is a basic component of the nacre matrix

Consistent with the matrix model of molluscan shell formation, we demonstrated the abundant presence of chitin in the shell matrix of *P. fucata martensii* (with both prismatic and nacreous layers) and *C. gigas* (mostly prismatic) by Calcofluor white M2R staining (Additional file 7: Figure S6a). Transcriptome analysis of different tissues indicated that some *chitin synthases* (*CHSs*) and *chitinases* were highly expressed in the mantle and pearl sac, the two main calcifying tissues responsible for shell and pearl formation (Additional file 7: Figure S6b and S6c). During larval development, some *CHSs* and *chitinases* were highly expressed at the trochophore and post-veliger stages (Additional file 7: Figure S6d), corresponding to prodissoconch and dissoconch/adult shell formation, respectively. Furthermore, the gene families of *chitinase* and *CHS* were significantly expanded in *P. fucata martensii* and other shelled molluscs (Additional file 1: Table S10). These results suggest that chitin is a key component of the organic matrix, and the expansion of *chitinase* and *CHS* genes in *P. fucata martensii* and other shelled molluscs might have played crucial roles in the evolution of advanced shells in molluscs.

The presence and involvement of collagen

According to the current model, silk proteins are major components of the organic matrix in molluscan shells. We searched for silk proteins in the *P. fucata martensii* genome and the proteome of the nacreous shell matrix but found none. Interestingly, a total of 355 unique spectra of 6 collagen VIs (COL6) were detected in the nacre proteome, ranking 6th among the known biomineralization-related proteins, compared with only 23 COL6 spectra in the prismatic layer proteome (Additional file 8: Datasets S1). Corresponding to the abundance of COL6s in the nacre proteome, the *P. fucata martensii* genome has an expanded family of 46 *COL6*s, similar to the 47 found in *C. gigas* [6] but more than the 22 found in *L. gigantea* and the 5 in humans (Additional file 1: Table S10). Together, the transcriptome and proteome data identified six COL6 proteins that were likely to be important components of the nacre matrix. The six *COL6*s were highly expressed in the mantle pallium and pearl sac, which are responsible for nacreous layer production (Fig. 1a). All six *COL6*s were up-regulated (at least 96×of the level in egg) in post-veliger larvae with nacreous/aragonite shells, again suggesting their crucial role in nacreous matrix

formation. Meanwhile, one of the six *COL6*s (Pma_10019835) was significantly up-regulated (40×of egg) at the trochophore stage, in correlation with aragonite shell formation (Fig. 1b). After inhibition of the six *COL6*s by RNA interference, the microstructure of the nacre showed disordered growth, as observed by scanning electron microscopy (SEM) (Fig. 1c and Additional file 9: Figure S7). These results suggest that COL6 is a major component of the nacreous organic matrix and plays a key role in nacreous shell formation in *P. fucata martensii*.

The typical COL6 structure in vertebrates consists of a short triple-helix region (THR) and globular structures made up of von Willebrand factor A (VWA) domains [15] (Fig. 1d). The COL6s of *P. fucata martensii,* other molluscs and Porifera have only VWA and some unique domains but no THRs (Fig. 1d, Additional file 1: Table S11). Furthermore, one COL6 (Pma_10015641) in the shell matrix has a chitin-binding domain, supporting its possible function in interacting with the chitin framework during matrix formation. Phylogenetic analysis suggests that this COL6 is derived early from an ancestor and might represent an ancient form of COL6 in molluscs (Fig. 1e). Collagens detected in the proteome of the skeletal organic matrix of the stony coral *Stylophora pistillata* (Spicol-A/B) also belong to the COL6 family (Fig. 1e). Some COL6 without THRs still exist in the human and zebrafish genomes (Fig. 1e). Furthermore, our analysis shows that fibrillar COL1/2/4 are derived from collagens without THRs (Fig. 1e).

Acidic glycosaminoglycans (GAGs) constitute a gel-like substance

According to the matrix model, the shell matrix contains a gel-like substance where acidic proteins induce the nucleation of calcium carbonate crystals [16]. Consistent with the model and previous reports, we identified a list of acid proteins that might be involved in shell formation (Additional file 8: Datasets S2). In addition to the acidic proteins that are unique to molluscan shells, we also found acidic GAGs, fibronectin-like proteins and chondroitin sulfotransferases that are characteristic components of vertebrate bone matrixes. By Alcian blue-periodic acid Schiff staining (AB-PAS), we found that the organic matrix extracted from nacreous shells contained large amounts of acidic GAGs compared with mainly neutral GAGs in prismatic layers of *P. fucata martensii* and *C. gigas* shells. In addition, we detected acid GAGs in secretory cells of the mantle pallium of *P. fucata martensii*, but mainly neutral GAGs in the mantle of *C. gigas* (Fig. 2a). Further, our data show that the *P. fucata martensii* genome has an expanded set of five types of sulfotransferase (Additional

file 1: Table S10), including chondroitin 4-sulfotransferase 11 (CHST11), chondroitin 6-sulfotransferase 3 (CHST3), carbohydrate 6-sulfotransferase 6 (CHST6), carbohydrate 4-sulfotransferase 9 (CHST9) and dermatan 4-sulfotransferase 1 (D4ST1). Corresponding to large amounts of acidic GAGs in the mantle pallium, some of the sulfotransferases (*CHST3*, *CHST11*, *CHST6* and *D4ST1*) exhibited higher expression levels in the mantle pallium than in the mantle edge (Fig. 2b). *CHST11* and *D4ST1* expressed at the post-veliger stage, whereas *CHST6* and *CHST3* were mostly up-regulated at the trochophore stage (Additional file 10: Figure S8a).

Tyrosinase may participate in the nacre matrix cross-linking

C. gigas has an expanded set of 26 *Tyrosinases* (*Tyrs*) [6], and we observed an even larger expansion of *Tyrs* in *P. fucata martensii* to 53 genes compared with 3 genes in *L. gigantea*, 1 in humans and 4 in coral (Additional file 1: Table S10). Phylogenetic analysis of these Tyrs from *P. fucata martensii* and *C. gigas* revealed unbalanced and lineage-specific expansion in both species (Fig. 2c, Additional file 1: Table S13). Their expression profiles in calcifying tissues and at shelled larval stages indicate that 29 of the expanded *P. fucata martensii Tyr*s may be involved in shell formation, among which 23 *Tyr*s were highly expressed after the post-veliger/spat stage, pointing to possible functions in adult shell formation (Fig. 2c). Seven *Tyr*s showed high expression levels in the mantle pallium (Additional file 10: Figure S8b), along with 9 *Tyrs* highly expressed in the pearl sac (Additional file 10: Figure S8c), corresponding to the greater abundance of quinoproteins in the nacreous than in the prismatic layers (Additional file 10: Fig. S8d). These results indicate that dopaquinone catalysed by Tyr may be essential for the assembly and maturation of the nacreous shell matrix.

Regulation network of the nacre matrix proteins

Weighted-gene co-expression network analysis (WGCNA) of the 234 nacre matrix protein genes revealed 27 hub genes at the centre of the network (Fig. 3, Additional file 8: Datasets S3 and S4), including well-known as well as novel genes for shell formation, such as *COL6*, *fibronectin III* and *Tyr*s, which reinforced our findings and demonstrated the usefulness of WGCNA. In addition, heat shock protein 70 (Hsp70), proteinase inhibitor I2-containing proteins and proteins with chitin-binding domains were also included in the hub genes, indicating their possible roles in nacre formation. Furthermore, we filtered the adjacent coefficients (no less than 0.5) and obtained 3245 crucial genes co-expressed with the nacre matrix proteins. These co-expressed crucial genes were significantly enriched ($P<0.05$) in the ErbB signalling pathway, the

Jak-STAT signalling pathway, the Wnt signalling pathway, osteoclast differentiation signalling pathways, ECM-receptor interactions and the vascular endothelial growth factor (VEGF) signalling pathway, which are all involved in bone formation (Additional file 1: Table S14). Lysosome, N-glycan and O-glycan biosynthesis and degradation were also enriched.

Discussion

All molluscan shells consist of calcium carbonate crystals embedded in organic matrixes and arranged in diverse microstructures that are classified into two basic types: the prismatic and nacreous forms. The aragonite nacre that gives pearls and certain shells their lustre and enhanced toughness is the target of many studies and modelling. According to the matrix model of molluscan shell formation, the mineralization of calcium carbonate is directed by a mantle-secreted organic matrix [17, 18], which is not fully understood but may contain chitin [19-21] and silk fibroin [22-24] for the structural framework and soluble acidic proteins for crystal nucleation [25-27]. Alternatively, the cellular hypothesis argues that biomineralization may be directed by hemocytes [7, 28] although there is no dispute about the involvement of organic matrixes which are the focus of our study. Our results provide strong evidence that chitin is the basic component of shell matrixes. Chitin is an ancient macromolecule and the primary framework component of organic matrixes in the cell walls of fungi and diatoms, sponge skeletons and arthropod shells [3]. It is possible that the chitin components of lophotrochozoan and ecdysozoan shells and of sponge skeletons constitute a shared feature and have the same ancient origin.

While silk proteins, which are also considered as the major components of the organic matrix in the molluscan shell, were not found in the *P. fucata martensii* genome and the proteome of the nacreous shell matrix, abundant presence of expanded COL6s were detected in the nacre proteome. In mammals, COL6 is a ubiquitously expressed extracellular matrix (ECM) protein and functions in linking cells and matrix macromolecules [15]. Compared with the typical COL6 structure in vertebrates, COL6 of *P. fucata martensii*, other molluscs and Porifera lack THRs, which is the crucial region that allows collagen subunits to assemble into triple-helixprotomers and form fibrillar collagens [29, 30]. The absence of THRs indicates that COL6 in the nacreous shells of *P. fucata martensii* might not self-assemble into fibrous structures but instead may cross-link with each other or other proteins to form a network

structure [15, 31, 32]. In addition, VWA domains bind to positive ions that attract water, and may provide initial hydrogel properties for biomineralization [33]. The finding of a chitin-binding domain in one COL6 supported its function in interacting with the chitin framework during matrix formation.

Collagens without THRs were also detected in the proteome of the skeletal organic matrix from the stony coral *S. pistillata*, and some COL6s without THRs still exist in the human and zebrafish genomes, and phylogenetic analysis indicates that COL6 without THR region is derived early during evolution. Together, these results indicated that COL6 without THRs might be one common and ancient protein in animals that gave rise to fibrillar COL1/2/4. In vertebrates, fibrillar COL1 and COL2 are the major collagens in phosphate bone and cartilage tissues, respectively [34]. In *Lingula*, fibrillar COL4 (lan col4) is the main component of the phosphate shell [35], and THRs are essential for calcium phosphate skeletons [34, 36]. The later origin of THRs suggests that the emergence of phosphate skeletons might be later than carbonate skeletons, supporting the view that phosphate skeletons in most early Cambrian fossils originated from calcitic, aragonitic or organic skeletons based on detailed analysis of the preserved microstructure [1, 37]. These findings suggest that the evolution of collagens is crucial in the divergence of carbonate and phosphate skeletons.

Mammalian cartilage and bone matrixes consist of collagen fibrils and a gel-like ground substance that is rich in chondroitin-containing proteoglycans, fibronectins and link proteins [38]. Our results confirm the presence of fibronectin-like proteinsin shells of *P. fucata martensii* and *C. gigas* [6]. Proteoglycans or GAGs, which have strong water-binding capabilities and have been detected in the shell [22], may function as the gel-like substance. In the nacre and the secretory cells of the mantle pallium of *P. fucata martensii*, we found large amounts of acidic GAGs, which have also been detected in coral [39] and bone [40], and this finding argues that the acidic GAGs might also play key roles in crystal nucleation during nacre formation. Combing this finding and the findings regarding collagens, our results suggest that the nacreous shell matrix, while having a chitin-based framework, also possesses key elements of collagen-based matrixes, such as collagens, fibronectins, proteoglycans and chondroitin sulfotransferases. Chitin- and collagen-based matrixes are considered as two basic types of biomineralizing matrixes, and our results suggest that they may have a common origin or might have co-existed as parts of an ancient/ancestral matrix

with dual-elements, despite subsequent divergence in different taxa into chitin- or collagen-based organic matrixes.

The shell organic matrix, rather than being a simple self-assembling structure, might instead be a complex and dynamic matrix that requires active construction, regulation and remodelling. Tyr, which can catalyse the formation of dopa and dopaquinone, may function in mediating intermolecular cross-links [6, 41]. The lineage-specific expansion and high expression levels of *Tyrs* in the mantle pallium and pearl sac, along with the great abundance of quinoproteins in the nacre indicated that dopaquinone catalysed by Tyr may be essential for the assembly and maturation of the nacreous shell matrix.

The complexity of the organic matrix and the biomineralization process is further demonstrated by co-expression network analysis. Together, these results suggest that molluscan shell formation is an elaborate and dynamic process that shares certain basic elements with mammalian bone formation, but with added complexity. Although molluscan shells have a chitin-dominated framework, the identification of elements of collagen-based matrixes supports a single origin of the two types of matrixes and a common biomineralization toolkit that may have been lost, modified and reorganized during evolution to produce diverse forms of biomineralized structures in the adaptation to new environments and in assuming new functions.

In conclusion, we sequenced and assembled the highly polymorphic genome of *P. fucata martensii* using NGS and the BAC-to-BAC strategy. Based on genomic, transcriptomic, and proteomic analyses and experimental studies, we identified a large number of genes related to shell nacre formation, which helped us to re-construct the shell matrix model (Fig. 4). The identification of COL6 without THRs and other elements of collagen-based matrixes in the chitin-rich nacre matrix not only supports the homology and single evolutionary origin of the common biomineralization toolkit, but also provides evidence that changes in collagen may underlie the divergence of the two principal classes of skeletal biominerals: calcium carbonate and phosphate. The hypothesis of a single evolutionary origin challenges the prevailing idea of independent evolution [2] and may stimulate homology-based studies towards a better understanding of the diverse forms of biomineralization.

Methods

Additional file 1 has additional information relating to the methodologies

described below.

Library construction and sequencing

We constructed all sequencing libraries according to protocols from Illumina and sequenced these libraries on a HiSeq 2000 sequencing system.

Hierarchical (BAC-to-BAC) assembly strategy

We used the BAC-to-BAC assembly approach as used for the moth genome [11]. Before the hierarchical assembly of BACs, we used SOAPdenovo to assemble the reads of each BAC with odd numbered K-mers from 27 to 63 and selected the best results with the longest scaffold N50 and total length, as primary scaffolds. Then, we used the paired-end reads information of the BACs and locally assembled the reads in the gap regions to fill in the gaps within the primary BAC scaffolds. Our custom assembly software (Rabbit) [11] was used to assemble scaffolds of BACs with large overlaps. After finding relationship among sequences, merging overlapping sequences and removing redundant sequences, we obtained longer segments as secondary scaffolds. Finally, SSPACE was used to join the secondary scaffolds to form final scaffolds, and SOAP-Gapcloser was used to fill in the gaps in the final scaffolds using all WGS reads with short insert sizes.

Linkage group construction

We constructed a genetic map using RAD-seq. A total of 148 F1 offsprings were sampled in a family obtained by crossing two relatively distant parents. We used SOAP2 [42] to map the reads to the reference genome sequences of *P. fucata martensii* (scaffolds) and performed SNP calling using SOAPsnp [43]. After SNP calling, we extracted genotypes by combining all SNPs among the 148 progeny and the 2 parents and constructed linkage map using Joinmap (version 4.1) [44].

Phylogenetic tree construction and divergence time estimation

We used Treefam to obtain gene families and one-to-one orthologs. To determine the phylogenetic position of *P. fucata martensii*, we used MrBayes to construct the phylogenetic tree.

Transcriptome analysis

We extracted total RNA from each sample and isolated mRNA using oligo (dT) magnetic beads. Then, the mRNA was fragmented into short fragments (200~500 bp) for construction of RNA-seq libraries that were sequenced on an Illumina HiSeq2000. Using SOAP2, all clean reads were mapped to the genome assembly with less than 5 mismatches. We used the *RPKM* method (Reads per kilobase transcript per million

mapped reads) to calculate the gene expression levels.

Extraction of matrix proteins from the nacre and prismatic layer

The prismatic layer was separated from the edges of pearl oyster shells without nacre. The nacre was directly scraped from the internal shell surfaces dominated by aragonite. These samples were thoroughly ground and soaked in acetic acid solution (5%, v/v) for at least 12 h to dissolve calcium carbonate, before being centrifuged at 14,000 g and 4 °C for 1 h. Acid-soluble proteins were in the supernatant, and acid-insoluble proteins were in the residue.

Samples were electrophoresed on 12% polyacrylamide gels and stained with Coomassie blue R-250. The extracted peptides were dried and stored at -80 °C until liquid chromatography/tandem mass spectrometry (LC-MS/MS) analysis.

Chitin identification in shell matrix

We decalcified the shells in 1 M acetic acid at 4 °C for one week, and the acid-insoluble material was collected. This insoluble material was washed with distilled water and embedded in paraffin for sectioning. The sections were placed on slides and stained for 5 min with 0.1% Calcofluor White M2R (Flupstain Ⅰ) (Sigma-Aldrich). Excess dye was rinsed off with distilled water. The stained specimens were observed under a confocal laser microscope using filters with 492 nm excitation and 520 nm emission [45].

RNAi experiment

The primers used for generating the *COL6* double-strand RNA (dsRNA) are shown in Additional file 1: Table S15. DsRNAs were synthesized following the method of Michio Suzuki et al. [46], and injected into the adductor muscle every 4 days at 100 μg per 100 μl per pearl oyster each time. The effects of RNAi of the six *COL6* genes on nacre formation were detected by SEM.

Identification of GAGs in shell and pearl

Shells were decalcified in 1 M acetic acid at 4 °C for 1 week and then in 10% EDTA-2NA solution at room temperature for 10 days. The fixed materials were embedded in paraffin and stained with AB/PAS (Alcian blue/periodic acid-Schiff) and observed using a OlympusBX51 optical microscope.

Nitrobluetetrazolium (NBT)/glycinate assay for dopa and dopaquione protein

Sections of decalcified shells were stained with 100 μL of solution containing 0.24 mM NBT and 2 M potassium glycinate (pH10) for nearly 5 min in darkness until

violet positive signals appeared [47]. The sections were rinsed with double-distilled water to stop the reaction and then mounted for microscopic examination.

Co-expression network analysis

We used WGCNA to reconstruct the co-expression network for biomineralization [48]. A weighted correlation network was constructed between all pairs of genes across four mantle tissue samples [49]. The adjacency matrix was calculated through a so-called 'soft' thresholding framework (power β=9) that converted the co-expression measure to a connection weight. Based on the adjacency matrix, we implemented a topological overlap dissimilarity measure to reflect relative inter-connectedness, which may represent a meaningful biological network. Hub genes (highly connected genes), by definition, tend to have high connectivity in the constructed network.

Availability of supporting data

The pearl oyster (*P. fucata martensii*) whole genome shotgun projects have been deposited at DDBJ/EMBL/GenBank under the accession numbers PRJNA283019.

List of abbreviations

CHS: chitin synthases; COL6: collagen VI; BMPs: bone morphogenetic proteins; VWA: von Willebrand factor A; CHST11: chondroitin 4-sulfotransferase 11; CHST3: chondroitin 6-sulfotransferase 3; CHST6: carbohydrate 6-sulfotransferase 6; CHST9: carbohydrate 4-sulfotransferase 9; D4ST1: dermatan 4-sulfotransferase 1; Tyr: Tyrosinase; WGCNA: Weighted-gene co-expression network analysis;

Competing interests

The authors declare that they have no competing interests.

Funding

This research supports from the Guangdong Ocean University Nature Science Foundation (University program: Genome studies of pearl oyster), the National Nature Science Foundation of China (31272635, 31372526, 41206141), Modern Agro-industry Technology Research System (CARS-48), USDA/NJAES Project 1004475/NJ32920 and "Taishan Oversea Scholar" program.

Authors' contributions

X.D., G.Z, and X.G. designed the scientific objectives. Y.J, R.H. W.C and G.F. managed the progect. C.B., F.S., X.L., C.S., W.L. and Z.W. performed the genome assembly, gene annotation and evolution analysis. Y.D. and Q.W. cultured P. martensii and provided materials. Y.D., Q.W., F.S., J.B and Z.W constructed the genetic map. Q.W, Z.Z., and R.H. performed the acid GAG analysis. Y.J., R.H., Z.W., Z.Z, J.L.

performed the chitin and COL6 related analysis. R.H. and C.B. performed the tyrosinase related analysis. Z.Z and H.Z. performed the WGCNA analysis. X.D., G.F. and X.G. directed final data analyses. X.L., Q.S, X.D., G.F, Y.J., H.Z., X.G., R.H., C.B, Y.D, Q.W., Z.Z. did most of the writing with contributions from all authors.

Acknowledgments

We acknowledge Z. He, W. Liu, Z. Wu, C. Liu, J. Jian, B. Tan for their supports of the pearl oyster genome project. We thank Y. Guo, X. Chen, M. Xue and Xuwen Pearl Oyster Farm for assistance with DNA, RNA and protein extraction, data analysis and oyster culture. We thank L. Goodman for helping to edit the manuscript. We thank other faculty and staff at Guangdong Ocean University, BGI-Shenzhen and Rutgers who contributed to the genome project. We acknowledge grant supports from the Guangdong Ocean University Nature Science Foundation (University program: Genome studies of pearl oyster), the National Nature Science Foundation of China (31272635, 31372526, 41206141), Modern Agro-industry Technology Research System (CARS-48). X.G. acknowledges support from USDA/NJAES Project 1004475/NJ32920 and “Taishan Oversea Scholar” program.

Author Information

Correspondence and requests for materials should be addressed to X.L. (liuxin@genomics.cn), G.Z. (gzhang@qdio.ac.cn), X.G. (xguo@hsrl.rutgers.edu) and X.D. (duxd@gdou.edu.c).

References

1. Knoll AH. Biomineralization and evolutionary history. Rev Mineral Geochem. 2003;54:329-356.
2. Drake JL, Mass T, Falkowski PG. The evolution and future of carbonate precipitation in marine invertebrates: Witnessing extinction or documenting resilience in the Anthropocene? Elementa: Science of the Anthropocene. 2014;2:000026.
3. Ehrlich H. Chitin and collagen as universal and alternative templates in biomineralization. Int Geol Rev. 2010;52:661-699.
4. Furuhashi T, Schwarzinger C, Miksik I, Smrz M, Beran A. Molluscan shell evolution with review of shell calcification hypothesis. Comp Biochem Phys B. 2009;154:351-371.
5. Addadi L, Joester D, Nudelman F, Weiner S. Mollusk shell formation: a source of new concepts for understanding biomineralization processes. Chem-Eur J. 2006;12:980-987.
6. Zhang G, Fang X, Guo X, Li L, Luo R, Xu F, et al. The oyster genome reveals stress adaptation and complexity of shell formation. Nature. 2012;490:49-54.
7. Mount AS, Wheeler A, Paradkar RP, Snider D. Hemocyte-mediated shell mineralization in the eastern oyster. Science. 2004;304:297-300.
8. Marin F, Luquet G, Marie B, Medakovic D. Molluscan shell proteins: primary structure, origin, and evolution. Curr Top Dev Biol. 2007;80:209-276.
9. Murdock DJ, Donoghue PC. Evolutionary origins of animal skeletal biomineralization. Cells Tissues Organs. 2011;194:98-102.
10. Takeuchi T, Kawashima T, Koyanagi R, Gyoja F, Tanaka M, Ikuta T, et al. Draft genome of the pearl oyster *Pinctada fucata*: a platform for understanding bivalve biology. DNA Res. 2012;19:117-130.
11. You M, Yue Z, He W, Yang X, Yang G, Xie M, et al. A heterozygous moth genome provides insights into herbivory and detoxification. Nat Genet. 2013;45:220-225.
12. Zhang H, Ahmad M, Gronowicz G. Effects of transforming growth factor-beta 1 (TGF-β1) on in vitro mineralization of human osteoblasts on implant materials. Biomaterials. 2003;24:2013-2020.
13. Miron RJ, Saulacic N, Buser D, Iizuka T, Sculean A. Osteoblast proliferation and differentiation on a barrier membrane in combination with BMP2 and TGFβ1. Clin Oral Invest. 2013;17:981-988.
14. Yan F, Luo S, Jiao Y, Deng Y, Du X, Huang R, et al. Molecular characterization of the BMP7 gene and its potential role in shell formation in *Pinctada martensii*. Int J Mol Sci. 2014;15:21215-21228.
15. Becker A-KA, Mikolajek H, Paulsson M, Wagener R, Werner JM. A structure of a collagen VI VWA domain displays N and C termini at opposite sides of the protein. Structure. 2014;22:199-208.
16. Nudelman F, Shimoni E, Klein E, Rousseau M, Bourrat X, Lopez E, et al. Forming nacreous layer of the shells of the bivalves *Atrina rigida* and *Pinctada margaritifera*: an environmental-and cryo-scanning electron

microscopy study. J Struct Biol. 2008;162:290-300.
17. Addadi L, Weiner S. Biomineralization: A pavement of pearl. Nature. 1997;389:912-915.
18. Nassif N, Pinna N, Gehrke N, Antonietti M, Jäger C, Cölfen H. Amorphous layer around aragonite platelets in nacre. P natl Acad Sci USA. 2005;102:12653-12655.
19. Levi-Kalisman Y, Falini G, Addadi L, Weiner S. Structure of the nacreous organic matrix of a bivalve mollusk shell examined in the hydrated state using cryo-TEM. J Struct Biol. 2001;135:8-17.
20. Weiss IM, Schönitzer V. The distribution of chitin in larval shells of the bivalve mollusk *Mytilus galloprovincialis*. J Struct Biol. 2006;153:264-277.
21. Furuhashi T, Beran A, Blazso M, Czegeny Z, Schwarzinger C, Steiner G. Pyrolysis GC/MS and IR spectroscopy in chitin analysis of molluscan shells. Biosci Biotech Bioch(BBB). 2009;73:93-103.
22. Pereira‐ Mouriès L, Almeida MJ, Ribeiro C, Peduzzi J, Barthélémy M, Milet C, et al. Soluble silk‐ like organic matrix in the nacreous layer of the bivalve *Pinctada maxima*. Eur J Biochem. 2002;269:4994-5003.
23. Sudo S, Fujikawa T, Nagakura T, Ohkubo T, Sakaguchi K, Tanaka M, et al. Structures of mollusc shell framework proteins. Nature. 1997;387:563-564.
24. Nudelman F, Chen HH, Goldberg HA, Weiner S, Addadi L. Spiers Memorial Lecture Lessons from biomineralization: comparing the growth strategies of mollusc shell prismatic and nacreous layers in *Atrina rigida*. Faraday Discuss. 2007;136:9-25.
25. Weiner S, Hood L. Soluble protein of the organic matrix of mollusk shells: a potential template for shell formation. Science. 1975;190:987-989.
26. Fu G, Valiyaveettil S, Wopenka B, Morse DE. CaCO3 biomineralization: acidic 8-kDa proteins isolated from aragonitic abalone shell nacre can specifically modify calcite crystal morphology. Biomacromolecules. 2005;6:1289-1298.
27. Evans JS. “Tuning in” to Mollusk Shell Nacre-and Prismatic-Associated Protein Terminal Sequences. Implications for Biomineralization and the Construction of High Performance Inorganic− Organic Composites. Chem Rev. 2008;108:4455-4462.
28. Li S, Liu Y, Liu C, Huang J, Zheng G, Xie L, et al. Hemocytes participate in calcium carbonate crystal formation, transportation and shell regeneration in the pearl oyster *Pinctada fucata*. Fish Shellfish Immun. 2016;51:263-270.
29. Kadler KE, Baldock C, Bella J, Boot-Handford RP. Collagens at a glance. J Cell Sci. 2007;120:1955-1958.
30. Van der Rest M, Garrone R. Collagen family of proteins. Faseb J. 1991;5:2814-2823.
31. Fitzgerald J, Mörgelin M, Selan C, Wiberg C, Keene DR, Lamandé SR, et al. The N-terminal N5 subdomain of the α3 (VI) chain is important for collagen VI microfibril formation. J Biol Chem. 2001;276:187-193.
32. Suhre MH, Gertz M, Steegborn C, Scheibel T. Structural and functional

features of a collagen-binding matrix protein from the mussel byssus. Nat Commun. 2014;5:3392-3392.
33. Whittaker CA, Hynes RO. Distribution and evolution of von Willebrand/integrin A domains: widely dispersed domains with roles in cell adhesion and elsewhere. Mol Biol Cell. 2002;13:3369-3387.
34. Boot- Handford RP, Tuckwell DS. Fibrillar collagen: the key to vertebrate evolution? A tale of molecular incest. Bioessays. 2003;25:142-151.
35. Aouacheria A, Geourjon C, Aghajari N, Navratil V, Deléage G, Lethias C, et al. Insights into early extracellular matrix evolution: spongin short chain collagen-related proteins are homologous to basement membrane type IV collagens and form a novel family widely distributed in invertebrates. Mol Biol Evol. 2006;23:2288-2302.
36. Brodsky B, Persikov AV. Molecular structure of the collagen triple helix. Adv Protein Chem. 2005;70:301-339.
37. Kouchinsky A. Shell microstructures in Early Cambrian molluscs. Acta Palaeontol. Pol. 2000;45.
38. Heinegård D, Oldberg A. Structure and biology of cartilage and bone matrix noncollagenous macromolecules. Faseb J. 1989;3:2042-2051.
39. Goldberg WM. Acid polysaccharides in the skeletal matrix and calicoblastic epithelium of the stony coral Mycetophyllia reesi. Tissue Cell. 2001;33:376-387.
40. Vejlens L. Glycosaminoglycans of human bone tissue. Calcified tissue research. 1971;7:175-190.
41. Aguilera F, McDougall C, Degnan BM. Evolution of the tyrosinase gene family in bivalve molluscs: Independent expansion of the mantle gene repertoire. Acta Biomater. 2014;10:3855-3865.
42. Hecker A, Mikulski Z, Lips KS, Pfeil U, Zakrzewicz A, Wilker S, et al. Pivotal Advance: Up-regulation of acetylcholine synthesis and paracrine cholinergic signaling in intravascular transplant leukocytes during rejection of rat renal allografts. J Leukocyte Biol. 2009;86:13-22.
43. Li R, Li Y, Fang X, Yang H, Wang J, Kristiansen K, et al. SNP detection for massively parallel whole-genome resequencing. Genome Res. 2009;19:1124-1132.
44. Van Ooijen J. Multipoint maximum likelihood mapping in a full-sib family of an outbreeding species. Genet Res. 2011;93:343-349.
45. Su X, Matthay MA, Malik AB. Requisite role of the cholinergic α7 nicotinic acetylcholine receptor pathway in suppressing gram-negative sepsis-induced acute lung inflammatory injury. J Immunol. 2010;184:401-410.
46. Suzuki M, Saruwatari K, Kogure T, Yamamoto Y, Nishimura T, Kato T, et al. An acidic matrix protein, Pif, is a key macromolecule for nacre formation. Science. 2009;325:1388-1390.
47. Paz M, Flückiger R, Boak A, Kagan H, Gallop PM. Specific detection of quinoproteins by redox-cycling staining. J Biol Chem. 1991;266:689-692.
48. Langfelder P, Horvath S. WGCNA: an R package for weighted correlation

network analysis. BMC bioinformatics. 2008;9:559.
49. Zhang B, Horvath S. A general framework for weighted gene co-expression network analysis. Stat Appl Genet Mol. 2005;4:1128.

Figure legend

Figure 1. Expression and structure analysis of COL6 genes in *P. fucata martensii*.

a. Expression of six COL6 genes implicated in nacreous shell formation showing higher expression in the mantle pallium (MP) and pearl sac (PS) than in other organs. Y-axis represents the relative expression level. X-axis represents nine organs (MP, mantle pallium; ME, mantle edge; A, adductor muscle; He, hepatopancreas; BC, hemocyte; Go, gonad; Gi, gill; F, foot; PS, pearl sac at 180 days after nucleus transplantation). **b.** Expression of six COL6 genes during early development with *COL6*-Pma_10019835 up-regulated at the trochophore (T) stage and all highly expressed at and after the post-veliger (PV) stage, corresponding to active adult shell formation. E, egg; Fe, fertilization; B, blastula; G, gastrula; ET, early trochophore; T, trochophore; D, D-larvae; DF, D-shaped larvae before feeding; EU, early umbo larvae; U, eye-larvae; PV, post-veliger; J, juveniles. **c.** Disordered microstructure of nacre observed after inhibition of two *COL6* genes (Pma_10015641 and Pma_44.543) with RNA interference (bar = 5 um). **d.** Structural analysis of COL6 genes from *P. fucata martensii* (Pma), *C. gigas* (Cgi), *L. gigantea* (Lgi), *A. queenslandica* (Aqu), *A. digitifera* (Adi), *H. sapiens* (Hsa) and *D. rerio* (Dre). P, G, L, Aq and Ad are various unique domains found in different taxa. **e.** Phylogenetic analysis of collagen proteins. Genes in green are the COL6 genes found in the shell matrix proteome of *P. fucata martensii*. Genes shaded in pink are collagens without triple-helix regions (THRs), and genes shaded in blue are COL1/2/4 with THRs.

Figure 2. GAGs and tyrosinase genes in *P. fucata martensii*.

a. The shell matrix extracted from the nacre of *P. fucata martensii* contains abundant acid glycosaminoglycans (GAGs) stained blue (I), whereas matrixes extracted from the prismatic layer of *P. fucata martensii* (I) and *C. gigas* (II) contain neutral GAGs stained red. Secretory cells (arrow) in the mantle pallium of *P. fucata martensii* are filled with acid GAGs stained blue (III), whereas cells in the mantle pallium of *C. gigas* contains neutral GAGs stained in red (IV). **b.** Expression (y-axis) of *CHST3*, *CHST11*, *CHST6* and *D4ST1* genes in the mantle pallium (MP) and the mantle edge (ME). **c.** Phylogenetic tree of the tyrosinase proteins from *P. fucata martensii* and *C. gigas*. Tyrosinase genes specifically expanded in *P. fucata martensii* are shaded in

purple, and their expression patterns during early development are presented in the heat map. E, egg; Fe, fertilization; B, blastula; G, gastrula; ET, early trochophore; T, trochophore; D, D-shaped larvae; DF, D-larvae before feeding; EU, early umbo larvae; U, eye-larvae; PV, post-veliger; J, juveniles.

Figure 3. Co-expression network of nacre formation-related genes of *P. fucata martensii*.

Hub genes are illustrated in the internal circle, where connections among them are coloured red. The number of visible links for each hub gene is represented by the size of the node. Links and their corresponding hub genes are in the same colour.

Figure 4. A model of nacre formation in *P. fucata martensii*.

In this model, new nacre (N) is formed in an organic matrix secreted by haemocytes or epithelial (Ep) cells beneath the mature nacre (M). Chitin provides the core of the polymer framework of the organic matrix. COL6 with chitin-binding domains binds to chitin and interacts with fibronectins and other COL6 proteins, forming the matrix networks. Asp-rich acid glycoproteins and acid GAGs function as the hydrogel substances. Tyrs catalyse the oxidation of tyrosine and dopamine and function in cross-linking and shell matrix maturation. Protease inhibitors, proteases and other enzymes regulate the biosynthesis or degradation of the organic matrix.

Additional file 2: Figure S1. Sequencing date and k-mer analysis.

a. The distribution of 17-mer depth derived from the WGS sequence reads. X-axis is the K-mer depth and the Y-axis is the percentage of each K-mer depth. The first peak is created by sequence polymorphism and its relative height provides a measure of heterozygosity in the diploid genome. **b.** The sequencing depth distribution of WGS reads mapped against the assembly. **c.** The assembled length of the BACs of four pooling libraries. Four libraries were randomly selected and the total length of each BAC's assembly was calculated.

Additional file 3: Figure S2. Assembly coverage of BACs.

Sequencing depth on the BACs was calculated by mapped sequence reads. The annotated transposable elements (TEs) are shown in black or red, and the remaining unclosed gaps on the scaffolds are marked as white blocks.

Additional file 4: Figure S3. Genome organization of *P. fucata martensii*.

a. Genetic map of *P. fucata martensii* constructed with RAD polymorphisms. The lines represent SNP positions on the linkage groups. **b.** The distribution of GC, gene,

repeat content and SNPs on *P. fucata martensii* pseudochromosomes. **c.** The synteny blocks between *C. gigas* (Cg) and *P. fucata martensii* (PIN).

Additional file 5: Figure S4. Phylogenetic analysis and gene clustering.

a. Species tree of *P. fucata martensii* and 6 other selected species. The number is the divergence time of the clades with ranges in parenthesis. **b.** Unique and shared gene families between *P. fucata martensii* (*P. mar*) and other three species including *C. gigas* (*C. gig*), *L. gigantea* (*L. gig*) and *Homo sapiens* (*H. sap*).

Additional file 6: Figure S5. Phylogenetic analysis of TGF-β1/2/3 and bone morphogenetic protein (BMP) from different species.

Proteins and accession numbers are listed in *SI Appendix*, Table S12.

Additional file 7: Figure S6. CHS and chitinase genes in *P. fucata martensii*.

a. Chitin in the shell matrix of *P. fucata martensii* and *C. gigas* stained green with Calcofluor White M2R. **b.** Expression analysis of *CHSs* in different organs. One *CHS* (Pma_10008435) is highly expressed in both the mantle pallium and the pearl sac. **c.** Expression analysis of *chitinases* in mantle pallium (MP), mantle edge (ME) and in pearl sac (PS), compared with non-calcifying tissues (including A, adductor muscle; He, hepatopancreas; BC, hemocyte; Go, gonad; Gi, gill; F, foot). **d.** Expression analysis of *Chitinases* and *CHSs* at different developmental stages of *P. fucata martensii*. Most of the *chitinases* are highly expressed at the T and PV stages. The expression of one *CHS* (Pma_10008435) gene, which is highly expressed both in mantle pallium and pearl sac, is also induced at the T and PV stages. E, egg; Fe, fertilization; B, blastula; G, gastrula; ET, early trochophore; T, trochophore; D, D-shaped larvae; DF, D-larvae before feeding; EU, early umbo larvae; U, eye-larvae; PV, post-veliger; J, juveniles.

Additional file 9: Figure S7. COL6 RNAi analysis in *P. fucata martensii.*

a. The expression profiles of Pma_10015641 and Pma_44.534 in the mantle under RNAi. **b.** Knockdown of other four *COL6s* performed using RNAi. The expression profiles of four *COL6* genes in the mantle, Pma_530.149, Pma_10019835, Pma_10019836 and Pma_10011175, were determined using real-time quantitative PCR, with GAPDH as the internal reference gene. *COL6s* were significantly inhibited in the treatment group ($P < 0.05$). SEM images of the surface of the nacre from *P. fucata martensii* injected with PBS and 100 μg RFP (red fluorescent protein) dsRNA demonstrated a normal growth status of nacre formation, whereas *P. fucata martensii* in the treatment group injected with Pma_530.149, Pma_10019835, Pma_10019836

and Pma_10011175 genes showed disruptions in crystal growth during nacre formation.

Additional file 10: Figure S8. Tyrosinases and sulfotransferases in *P. fucata martensii*.

a. Expression analysis of *sulfotransferase* genes in early development. *CHST11* (Pma_133.4) and *D4ST1* (Pma_10006752) showed expression at the PV stage, whereas *CHST6* (Pma_279.110) and *CHST3* (Pma_10022575) were mostly up-regulated at the T stage. E, egg; Fe, fertilization; B, blastula; G, gastrula; ET, early trochophore; T, trochophore; D, D-shaped larvae; DF, D-larvae before feeding; EU, early umbo larvae; U, eye-larvae; PV, post-veliger; J, juveniles. **b,c.** *Tyr* expression in the mantle and pearl sac, respectively, compared with other non-calcifying tissues (including A, adductor muscle; He, hepatopancreas; BC, hemocyte; Go, gonad; Gi, gill; F, foot). Seven *Tyrs* that highly expressed in mantle pallium (MP) were shown in b, the different cycles represent different *Tyrs* (inside-out: Pma_10005159, Pma_10013533, Pma_10015392, Pma_10016044, Pma_10021421, Pma_10021422, Pma_10022578). The expression patterns of *Tyrs* in pearl sac (PS) compared with other non-calcifying tissues were exhibited in c, with nine *Tyrs* highly expressed in PS that were marked with red frame. **d.** Abundance of quinoproteins (stained purple) in the nacre matrix revealed by a NBT/glycinate assay. Triangle represented prismatic layer and arrows represented nacreous layer.

Figure 1 Click here to download Figure Fig.1.tif

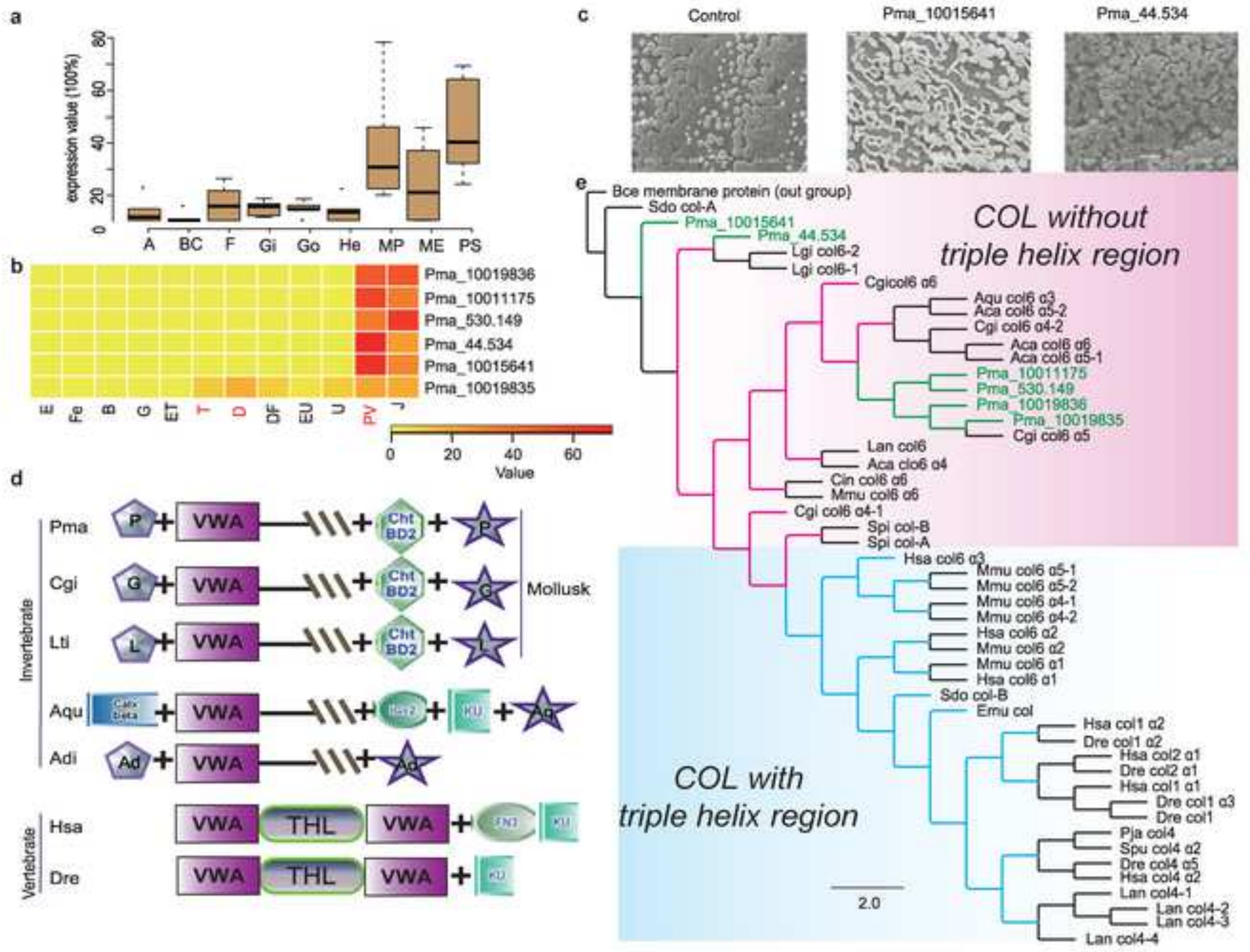

Figure 2

Click here to download Figure Fig.2.tif

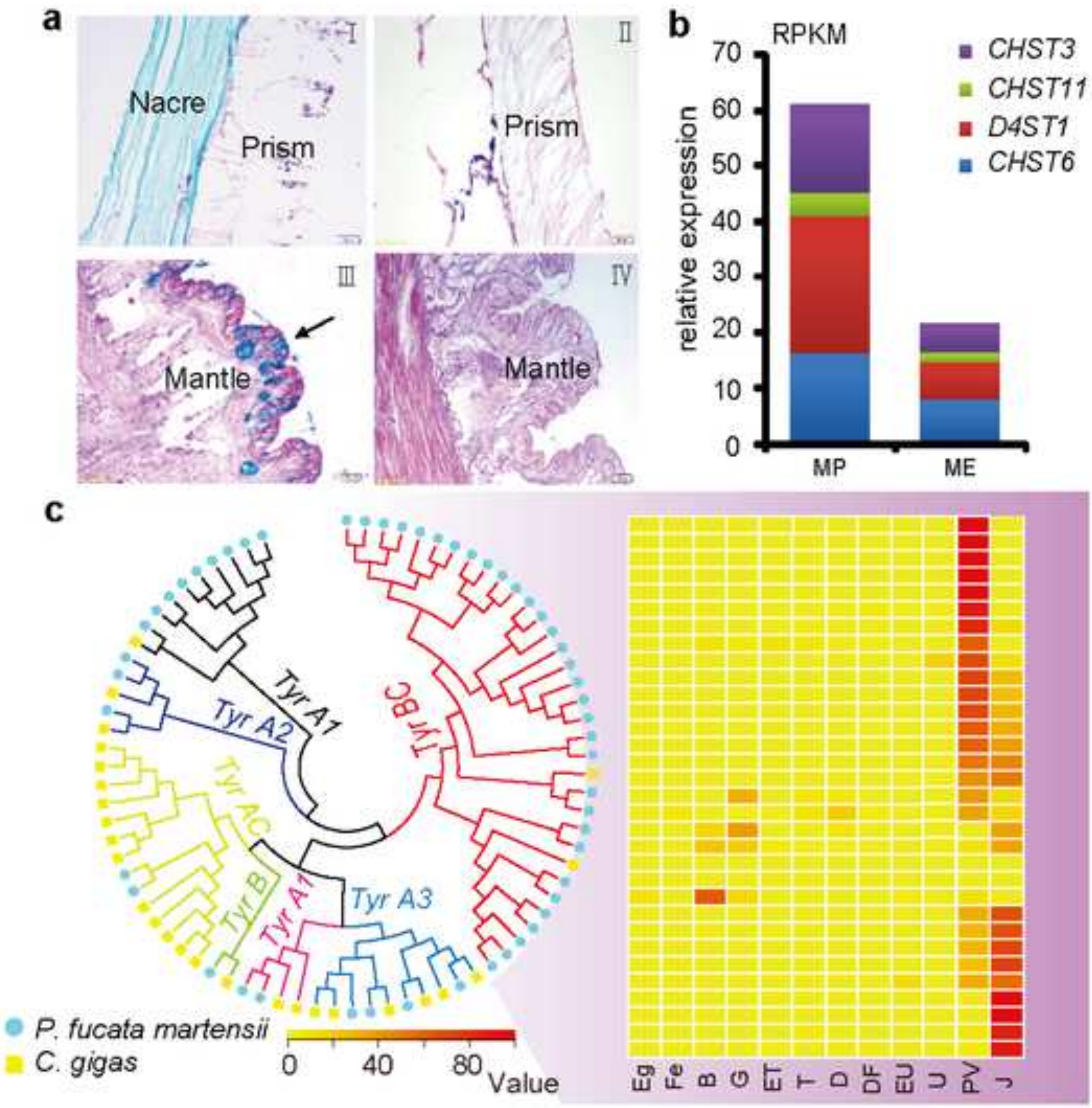

Figure 3 Click here to download Figure Fig.3.tif

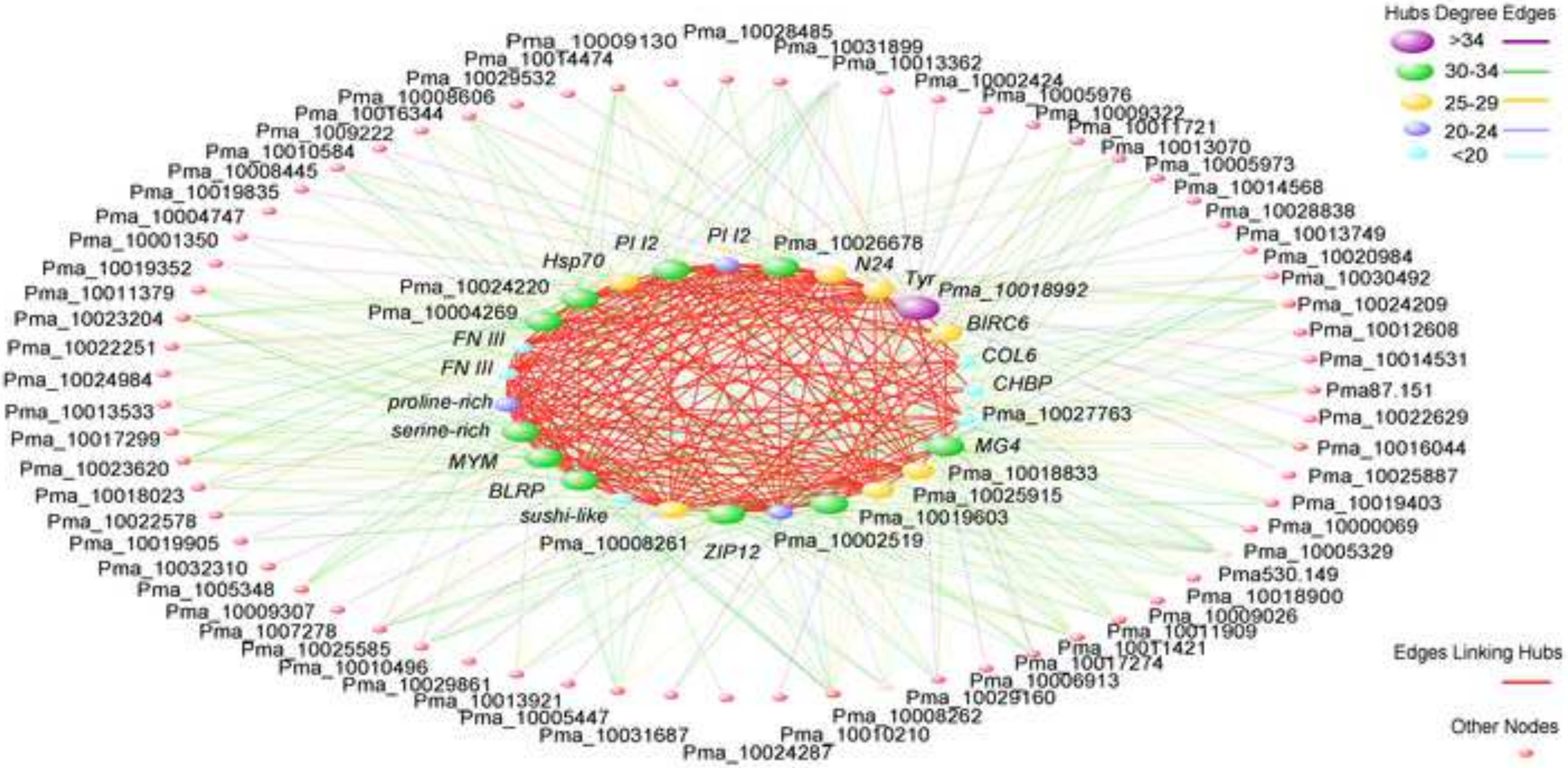

Figure 4

Click here to download Figure Fig.4.tif

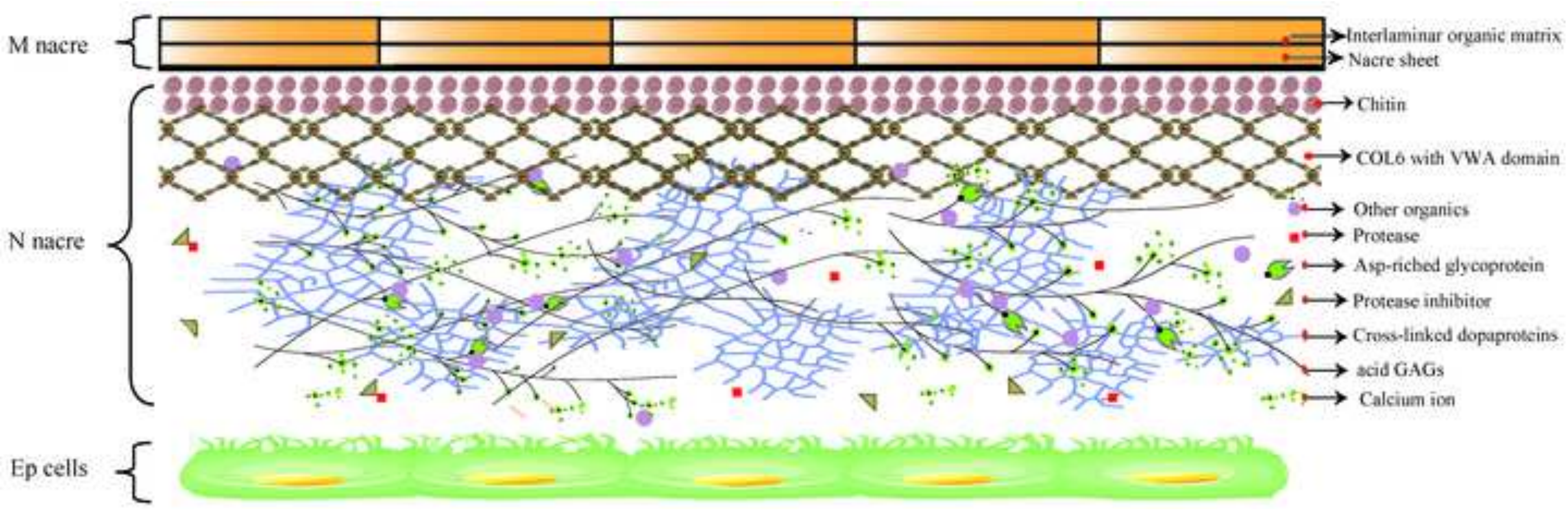

Figure S1

Click here to access/download
Supplementary Material
Additional file 2.figure S1.tif

Figure S2

Click here to access/download
Supplementary Material
Additional file 3.figure S2.tif

Figure S3

Click here to access/download
Supplementary Material
Additional file 4. figure S3.tif

Figure S4

Click here to access/download
Supplementary Material
Additional file 5.figure S4.tif

Figure S5

Click here to access/download
Supplementary Material
Additional file 6.figure S5.tif

Figure S6

Click here to access/download
Supplementary Material
Additional file 7.figure 6.tif

Figure S7

Click here to access/download
Supplementary Material
Additional file 9.figure S7.tif

Figure S8

Click here to access/download
Supplementary Material
Additional file 10.figure 8.tif

supplementary information and table

Click here to access/download

Supplementary Material

Additional file 1.docx

supplementary table

Click here to access/download
Supplementary Material
Additional file 8.xlsx

cover letter
Click here to download Personal Cover cover letter GigaScience.docx

Dear editor,

Please find enclosed our manuscript entitled "The genome of *Pinctada fucata martensii* and multi-omic analyses provide insights into biomineralization", which we would like to submit for publication as an original article in GigaScience.

Nacre, the iridescent material found in pearls and shells of molluscs, is formed through an extraordinary process of matrix-assisted biomineralization that is not yet well understood. We sequenced the highly polymorphic genome of the pearl oyster *Pinctada fucata martensii* and conducted multi-omic and biochemical studies to probe nacre formation. We identified a large set of novel genes participating in matrix-framework formation, including many in expanded families. Our analysis reveals that chitin, collagen VI, fibronectin and chondroitin sulfotransferases are key elements in nacre matrix-framework. Considering that there are only collagen-based matrixes in vertebrate bones and chitin-based matrixes in most invertebrate skeletons, the presence of both chitin and collagen VI in nacre matrixes suggests that elements of chitin- and collagen-based matrixes are deeply rooted and might be part of an ancient biomineralizing matrix. Our results expand the current shell matrix-framework model of biomineralization and provide new insights into the evolution of diverse biomineralization systems.

The pearl oyster (*P. fucata martensii*) whole genome shotgun projects have been deposited at DDBJ/EMBL/GenBank under the accession numbers PRJNA283019.

We confirm that this manuscript is original research, has not been published elsewhere and is not under consideration by another journal. All authors have approved the manuscript and agreed with submission to GigaScience. The authors have no conflicts of interest to declare.

We look forward to hearing from you at your earliest convenience.

Yours sincerely,

Xiaodong Du, PhD
