# Supplementary material for: The pearl oyster Pinctada fucata martensii genome and multi-omic analyses provide insights into biomineralization
Source: Gigascience. 2017 Jul 25;6(8):1–12. doi: 10.1093/gigascience/gix059 (PMC5597905; doi:10.1093/gigascience/gix059)
Supplement: GIGA-D-16-00075_Revision-1.pdf [file gix059_GIGA-D-16-00075_Revision-1.pdf]

Manuscript
Click here to download Manuscript manuscript-06.docx

The pearl oyster genome and multi-omic analyses provide insights into biomineralization

Xiaodong Du[a,*,#], Guangyi Fan[b,g*], Yu Jiao[a,*], He Zhang[b,*], Ximing Guo[c,*,#], Ronglian Huang[a,*], Chao Bian[b,*], Yuewen Deng[a,*], Qingheng Wang[a,*], Zhe Zheng[a,*], Zhongduo Wang[a*], Xinming Liang[b], Haiying Liang[a], Chengcheng Shi[b], Xiaoxia Zhao[a], Fengming Sun[b], Ruijuan Hao[a], Jie Bai[b], Jialiang Liu[a], Wenbin Chen[b], Jinlian Liang[a], Weiqing Liu[b], Zhe Xu[e], Qiong Shi[b], Xun Xu[b], Guofan Zhang[d,f,#], Xin Liu[b,#]

*These authors contributed equally.

#Corresponding authors: X.D. (zjduxd@126.com), X.G. (xguo@hsrl.rutgers.edu), G.Z. (gzhang@qdio.ac.cn) and X.L. (liuxin@genomics.cn).

[a]Fishery College, Guangdong Ocean University, Zhanjiang, 524025, China;

[b]BGI-Shenzhen, Shenzhen, 518083 China;

[c]Haskin Shellfish Research Laboratory, Department of Marine and Coastal Sciences, Rutgers University, Port Norris, New Jersey 08349, USA;

[d]Institute of Oceanology, Chinese Academy of Science, Qingdao 266071, China;

[e]Atlantic Cape Community College, Mays Landing, New Jersey 08330, USA;

[f]Laboratory for Marine Biology and Biotechnology, Qingdao National Laboratory for Marine Science and Technology, Qingdao, China

[g]BGI-Qingdao, Qingdao 266555, China

Abstract

Background: Nacre, the iridescent material found in pearls and shells of molluscs, is formed through an extraordinary process of matrix-assisted biomineralization. Despite recent advances, many parts of the biomineralization process and its evolutionary origin remain a mystery. The pearl oyster *Pinctada fucata martensii* is a well-known master of biomineralization, but the process it uses to produce remarkable shells and pearls is not fully understood.

Results: We sequenced the highly polymorphic genome of the pearl oyster and conducted multi-omic and biochemical studies to probe nacre formation. We identified a large set of novel genes participating in matrix-framework formation, including many in expanded families. Our analysis reveals that chitin, collagen VI-like (COL6L) protein, fibronectin and chondroitin sulfotransferases are key elements in nacre matrix-framework.

Conclusions: Considering that there are only collagen-based matrices in vertebrate bones and chitin-based matrices in most invertebrate skeletons, the presence of both chitin and COL6L proteins in nacre matrices suggests that elements of chitin- and collagen-based matrices are deeply rooted and might be part of an ancient biomineralizing matrix. Our results expand the current shell matrix-framework model and provide new insights into the evolution of diverse biomineralization systems.

Keywords: genome, biomineralization, nacre, collagen, *Pinctada fucata martensii*

Background

Biomineralization is an extraordinary process where minerals form not following rules of inorganic chemistry but through active biological facilitation and control. Biomineralization is widely distributed and essential to the lives of diverse organisms, ranging from algae to vertebrates that rely on mineralized materials for morphology, structure, protection, movement and feeding. Three principal classes of skeletal biominerals exist on earth: calcium carbonate, calcium phosphate and silica [1]. Whether these skeletal biominerals evolved independently or derived from a common origin is controversial, although current thinking favours independent evolution [2]. One of the remarkable characteristics of biomineralization is its precise control by organic matrices [3]. Organic matrices are complex and variable but can be classified into two basic and highly conserved types that use either chitin or collagen as the

templating framework [3]. Despite great interest in harnessing the power of biomineralization for the production of novel materials, our understanding of biomineralization and associated matrices is limited in many taxa, including the well-known masters of biomineralization - shelled molluscs.

Nacre is the remarkable biomineral found in pearls and shells of molluscs that provides lustre and enhanced toughness. The formation of lustrous pearls and shells in molluscs such as the pearl oyster *Pinctada fucata martensii* has long fascinated humans. The biomineralization process of nacre formation is complex and involves sophisticated organic matrices as well as cells, many aspects of which remain elusive [4-8]. The origin and homology of nacre formation with other biomineralization processes such as crustacean shell and vertebrate bone formation is not understood [9]. Studies of biomineralization and other fundamental questions in biology and evolution can be greatly empowered by whole genome analyses, which have been difficult in molluscs owing to challenges in assembling their highly polymorphic and complex genomes [6, 10]. To understand the biomineralization process of nacre, we sequenced and assembled the *P. f. martensii* genome and generated transcriptomes from 11 organs/tissues and 12 developmental stages, along with the proteomes of shell organic matrices.

Data description

We used a pearl oyster from a line selected for fast growth for 3 generations for sequencing and assembly using BAC-to-BAC strategy. In addition to BAC sequencing, we also constructed whole genome shotgun (WGS) libraries including 3 with short insert-sizes and 4 with long insert-sizes. We used a draft assembly from a previous study [10], Sanger-sequenced BACs and transcripts generated by RNA-seq to assess the integrity of our assembly. Furthermore, to anchor scaffolds to chromosomes, we constructed a genetic map using restriction site associated DNA sequencing (RAD-seq) using 148 F1 offspring obtained by crossing two genetically distant parents.

To determine gene expression profile in different organs or tissues, we performed transcriptome sequencing on nine organs and tissues, including adductor muscle, mantle pallium, mantle edge, hepatopancreas, hemocyte, gonad, gill, foot, and pearl sac at 180 days (d) after nucleus transplantation. Further, we performed transcriptome sequencing on 12 developmental samples to determine gene expression profile during development. Developmental samples included unfertilized eggs, 11 samples obtained

at 30 min, 5 h, 6 h, 8 h, 16 h, 19 h, 4 d, 14 d, 28 d, 40 d, and 90 d after fertilization. To understand gene regulation during nacre formation, we analyzed transcriptome data from mantle edge, mantle pallial and two entire mantle tissues representing fast and slow growing pearl oysters with WGCNA (weighted-gene co-expression network analysis), and obtained co-expression network patterns. All sequencing and genome data were uploaded to GigaDB under the accession number XXX.

Results

Genome assembly and characterization. As our initial assembly of ~130 Gb (134-fold coverage) of whole-genome shotgun (WGS) Illumina sequences (Additional file 1: Table S1) was too fragmented for annotation and analysis, probably due to high polymorphism and repetitive sequences (Additional file 2: Figure S1a and b), we subsequently adopted a BAC-to-BAC (bacterial artificial chromosome) sequencing strategy [6, 11]. We sequenced 46,080 BACs (5-fold genome coverage) to a depth of 100X using Illumina next-generation sequencing (NGS), assembled each BAC separately (Additional file 2: Figure S1c), and then built supercontigs after merging and filtering redundant sequences. After constructing scaffolds and filling gaps with WGS reads, we obtained a final assembly of 990,658,107 bp with a contig N50 size of 21 kb and a scaffold N50 of 324 kb (Additional file 1: Table S2), which was a significant improvement compared with the contig N50 of 1.6 kb of the previous draft assembly [10].

The coverage of our assembly was demonstrated by the successful mapping of 90.5% of contigs, 95.5% (coverage ≥50%) of gene-model regions of the previous draft assembly [10], 99.8% of transcripts (coverage ≥50%), and all four BACs (coverage ≥93.2%) sequenced with Sanger technology (Additional file 1: Table S3 and S4; Additional file 3: Figure S2). We constructed a high-density genetic map of 14 linkage groups in accordance with the haploid number, using RAD-seq of a full-sib family (Fig. 1). We were able to anchor 857.07 Mb (86.5%) scaffolds to the genetic map with 4,463 single-nucleotide polymorphisms (SNPs) (Additional file 1: Table S5; Fig. 1b). Through alignment of our pseudochromosomes to that of *Crassostrea gigas*, we identified 2,240 syntenic blocks and several possible chromosome rearrangements (Fig. 1c).

Combining *de novo* prediction and evidence-based annotation using published data and transcriptomes from 11 organs/tissues and 12 developmental stages (Additional file 1: Table S6), we identified 32,937 protein-coding gene models

(Additional file 1: Table S7), which is comparable to the gene numbers found in *Capitella teleta* (32,389) and *C. gigas* (28,027) but higher than those in *Drosophila melanogaster* (23,847), *Helobdella robusta* (23,400) and *Lottia gigantea* (23,800). Searches against public databases showed that 84.0% of the gene models matched known proteins (Additional file 1: Table S8). Further, BUSCO analysis shows that 82.8% of predicted genes are completed and 7.4% of them are fragmented, indicating our assembly is adequate for further analysis. To assess the impact of selection, we determined codon usage, GC content of intron, exon and inter-genic regions, and GC content at each codon position, which were similar in *P. f. martensii* and other 8 species (Additional file 4: Figure S3).

Phylogenetic analysis of the sequenced genomes of *P. f. martensii*, *C. gigas* and *L. gigantea* along with selected model organisms provided estimates of divergence times: 485 million years ago (mya) between *P. f. martensii* (Bivalvia) and *L. gigantea* (Gastropoda) and 316 mya between *P. f. martensii* (Pteriidae) and *C. gigas* (Ostreidae) (Additional file 5: Figure S4). These estimates are in agreement with the most up-to-date phylogenetic analyses of molluscan evolution [12]. Compared to *Homo sapiens* and *Danio rerio*, molluscan genomes do not have transforming growth factor (TGF)-beta factors but only bone morphogenetic proteins (BMPs), but these two proteins share a common origin with TGF-beta being derived from BMPs (Additional file 1: Table S10, S11 and S12; Additional file 6: Figure S5,). TGF-beta factors are crucial in regulating osteoblast proliferation, differentiation and bone matrix maturation in vertebrates [13, 14]. This finding suggests that molluscs have maintained an ancient BMP-regulatory system for shell formation [15], while TGF-beta emerged in vertebrates to regulate bone matrix.

Chitin is a basic component of the nacre matrix. Consistent with the matrix model of molluscan shell formation, we demonstrated the abundant presence of chitin in the shell matrix of *P. f. martensii* (in both prismatic and nacreous layers) and *C. gigas* (mostly prismatic) by Calcofluor white M2R staining (Additional file 7: Figure S6a). Transcriptome analysis of different tissues indicated that some *chitin synthases* (*CHSs*) and *chitinase* were highly expressed in the mantle and pearl sac, the two main calcifying tissues responsible for shell and pearl formation (Additional file7: Figure S6b and S6c). During larval development, some *CHSs* and *chitinases* were highly expressed at the trochophore and post-veliger stages (Additional file 7: Figure S6d), corresponding to prodissoconch and dissoconch/adult shell formation, respectively.

Furthermore, the gene family of *CHS* was significantly expanded in *P. f. martensii* and other shelled molluscs, but not in molluscs without shells, *Octopus bimaculoides* (Additional file 1: Table S10). These results suggest that chitin is a key component of the shell matrix, and *CHS* genes in *P. f. martensii* and other shelled molluscs might have played crucial roles in the evolution of advanced shells in molluscs.

The presence and involvement of collagen-like proteins. According to the current model, silk proteins are major components of the organic matrix in molluscan shells. We searched for silk proteins in the *P. f. martensii* genome and the proteome of the nacreous shell matrix but found none. Interestingly, 355 unique spectra of 6 collagen VI-like proteins (COL6Ls) were detected in the nacre proteome, ranking 7th among the known biomineralization-related proteins, compared with only 23 COL6L spectra in the prismatic layer proteome (Additional file 8: Datasets S1). Corresponding to the abundance of COL6Ls in the nacre proteome, the *P. f. martensii* genome has an expanded family of 46 *COL6L*s, similar to the 47 found in *C. gigas* [6] but more than the 22 found in *L. gigantea* and the 10 in humans (Additional file 1: Table S10). Together, the transcriptome and proteome data identified six COL6L proteins that were likely to be important components of the nacre matrix. The six *COL6Ls* were highly expressed in the mantle pallium and pearl sac, which are responsible for nacreous layer production (Fig. 2a). All six *COL6Ls* were up-regulated (at least 96X of the level in egg) in post-veliger larvae with nacreous/aragonite shells, again suggesting their crucial role in nacreous matrix formation. Meanwhile, one of the six *COL6Ls* (Pma_10019835) was significantly up-regulated (40X of egg) at the trochophore stage, in correlation with aragonite shell formation (Fig. 2b). After inhibition of the six *COL6L*s by RNA interference, the microstructure of the nacre showed disordered growth, as observed by scanning electron microscopy (SEM) (Fig. 2c and Additional file 9: Figure S7). These results suggest that COL6Ls are a major component of the nacreous organic matrix and play a key role in nacreous shell formation in *P. f. martensii*.

The typical COL6 structure in vertebrates consists of a short triple-helix region (THR) and globular structures made up of von Willebrand factor A (VWA) domains [16] (Fig. 2d). COL6Ls of *P. f. martensii*, other molluscs and Porifera have only VWA and some unique domains but no THRs (Fig.2d, Additional file 1: Table S11). Furthermore, one COL6L (Pma_10015641) in the shell matrix has a chitin-binding domain, supporting its possible function in interacting with the chitin framework

during matrix formation. Phylogenetic analysis suggests that this COL6L is derived early from an ancestor and might represent an ancient form of COL6L in molluscs (Fig. 2e). Collagens detected in the proteome of the skeletal organic matrix of the stony coral *Stylophora pistillata* (Spicol-A/B) also belong to the COL6 family (Fig. 2e). Some COL6 without THRs still exist in the human and zebrafish genomes (Fig. 2e). Furthermore, our analysis shows that fibrillar COL1/2/4 that are associated with vertebrate bones are derived from collagens without THRs (Fig. 2e).

Acidic glycosaminoglycans (GAGs) constitute a gel-like substance. According to the matrix model, the shell matrix contains a gel-like substance where acidic proteins induce the nucleation of calcium carbonate crystals [17]. Consistent with the model and previous reports, we identified a list of acid proteins that might be involved in shell formation (Additional file 8: Datasets S2). In addition to the acidic proteins that are unique to molluscan shells, we also found acidic GAGs, fibronectin-like proteins and chondroitin sulfotransferases that are characteristic components of vertebrate bone matrices. By Alcian blue-periodic acid Schiff staining (AB-PAS), we found that the organic matrix extracted from nacreous shells contained large amounts of acidic GAGs compared with mainly neutral GAGs in prismatic layers of *P. f. martensii* and *C. gigas* shells. In addition, we detected acid GAGs in secretory cells of the mantle pallium of *P. f. martensii*, but mainly neutral GAGs in the mantle of *C. gigas* (Fig. 3a). Further, our data show that the *P. f. martensii* genome has an expanded set of five types of sulfotransferase (Additional file 1: Table S10), including chondroitin 4-sulfotransferase 11 (CHST11), chondroitin 6-sulfotransferase 3 (CHST3), carbohydrate 6-sulfotransferase 6 (CHST6), carbohydrate 4-sulfotransferase 9 (CHST9) and dermatan 4-sulfotransferase 1 (D4ST1). Corresponding to large amounts of acidic GAGs in the mantle pallium, some of the sulfotransferases (*CHST3*, *CHST11*, *CHST6* and *D4ST1*) exhibited higher expression levels in the mantle pallium than in the mantle edge (Fig. 3b). *CHST11* and *D4ST1* expressed at the post-veliger stage, whereas *CHST6* and *CHST3* were mostly up-regulated at the trochophore stage (Additional file 10: Figure S8a).

Tyrosinase may participate in the nacre matrix cross-linking. *C. gigas* has an expanded set of 26 *tyrosinases* (*Tyrs*) [6], and we observed an even larger expansion of *Tyrs* in *P. f. martensii* to 53 genes compared with 3 genes in *L. gigantea*, 1 in humans and 4 in coral (Additional file 1: Table S10). Phylogenetic analysis of these Tyrs from *P. f. martensii* and *C. gigas* revealed unbalanced and lineage-specific

expansion in both species (Fig. 3c, Additional file 1: Table S13). Their expression profiles in calcifying tissues and at shelled larval stages indicate that 29 of the expanded *P. f. martensii Tyr*s may be involved in shell formation, among which 23 were highly expressed after the post-veliger/spat stage, pointing to possible functions in adult shell formation (Fig. 3c). Seven *Tyr*s showed high expression levels in the mantle pallium (MP, Additional file10: Figure S8b) and 9 *Tyrs* highly expressed in the pearl sac (Additional file 10: Figure S8c), compared with 13 *Tyrs* highly expressed in mantle edge (ME). Twelve Tyrs were identified from shell proteome: 2 specific to nacreous layer and highly expressed in MP, 4 specific to prismatic layer and highly expressed in ME and 6 found in both nacreous and prismatic layers. Greater abundance of quinoproteins was observed in the nacreous than in the prismatic layers (Additional file 10: Figure S8d). These results indicate that dopaquinone catalysed by Tyr may be essential for the assembly and maturation of both nacreous and prismatic shell matrices.

Regulation network of the nacre matrix proteins. WGCNA of the 234 nacre matrix protein genes revealed 27 hub genes at the centre of the network (Fig. 4, Additional file 8: Datasets S3 and S4), including well-known as well as novel genes for shell formation, such as *COL6L*, *fibronectin III* and *Tyr*s, which reinforced our findings and demonstrated the usefulness of WGCNA. In addition, heat shock protein 70 (Hsp70), proteinase inhibitor I2-containing proteins and proteins with chitin-binding domains were also included in the hub genes, indicating their possible roles in nacre formation. Furthermore, we filtered the adjacent coefficients (no less than 0.5) and obtained 3245 crucial genes co-expressed with the nacre matrix proteins. These co-expressed crucial genes were significantly enriched ($P<0.05$) in the ErbB signalling pathway, the Jak-STAT signalling pathway, the Wnt signalling pathway, osteoclast differentiation signalling pathways, ECM-receptor interactions and the vascular endothelial growth factor (VEGF) signalling pathway, which are all involved in bone formation (Additional file 1: Table S14). Meanwhile, metabolism of polysaccharide such as glycosaminoglycan, N-glycan and O-glycan were also implicated. Analysis by Gene Ontology indicated that genes related to transmembrane transporter activity were significantly enriched, which is consistent with the enrichment of ABC transporters in KEGG analysis (Additional file 1: Table S14, S15).

Discussion

The assembly of highly polymorphic genomes and gene prediction in non-model organisms remain major challenges. Software based on *de Bruijn* Graph, such as SOAP*denovo* [18], is inadequate in producing satisfactory results due to the increased complexity of *de Bruijn* graph structure. Overlap-Layout-Consensus assembler, such as Celera Assembler [19], based on the data of fosmids or BACs hierarchical sequencing and third-generation long reads (such as PacBio long reads) are employed with overcoming such problems. However, the best choice for assembling complex genomes is to sample haploid or homozygous sequences. For the *ab initio* gene prediction software, such AUGUSTUS [20], the aim is to find potential coding sequences with sufficiently long open reading frames, but the translated regions may be too short making the absence of stop codons meaningless. The similarity-based approaches including homologous protein sequences, EST sequences and transcripts assembled from RNA-seq reads can produce biologically relevant predictions, but they may not cover entire coding exons. Considering their strengths and weaknesses, synthesis software, such as GLEAN [21] and MAKER [22], were used to synthesize these evidences obtained from *ab initio* gene predictions and similarity-based approaches into the final gene annotation. BUSCO analysis [23] indicates our assembly is complete enough for further analysis.

The aragonite nacre that gives pearls and certain shells their lustre and enhanced toughness is the target of many studies and modelling. According to the matrix model of molluscan shell formation, the mineralization of calcium carbonate is directed by a mantle-secreted organic matrix [24, 25], which is not fully understood but may contain chitin [26-28] and silk fibroin [29-31] for the structural framework and soluble acidic proteins for crystal nucleation [32-34]. Alternatively, the cellular hypothesis argues that biomineralization may be directed by hemocytes [7, 35] although there is no dispute about the involvement of organic matrices which are the focus of our study. Chitin is an ancient macromolecule and the primary framework component of organic matrices in cell walls of fungi and diatoms, sponge skeletons and arthropod shells [3]. It is possible that the chitin components of lophotrochozoan and ecdysozoan shells and of sponge skeletons constitute a shared feature and have the same ancient origin. Our results provide strong evidence that chitin is the basic component of *P. f. martensii* shell matrices.

The connection or interaction between chitin and other matrix proteins is a crucial part of matrix assembly. One COL6L with chitin-binding domain identified from

nacreous shell matrix supports its function in interacting with the chitin framework during matrix formation. While silk proteins, which are also considered as the major components of the organic matrix in the molluscan shell, were not found in the *P. f. martensii* genome and the proteome of the nacreous shell matrix, abundant presence of expanded COL6s were detected in the nacre proteome. Liu et al. also found two VWA domain proteins that could be identified as COL6L in the shell matrix proteome of *P. fucata* [36]. In mammals, COL6 is a ubiquitously expressed extracellular matrix (ECM) protein and functions in linking cells and matrix macromolecules [16]. Compared with the typical COL6 structure in vertebrates, COL6L of *P. f. martensii*, other molluscs and Porifera lack THRs, which is the crucial region that allows collagen subunits to assemble into triple-helix protomers and form fibrillar collagens [37, 38]. The absence of THRs indicates that COL6L in the nacreous shells of *P. f. martensii* might not self-assemble into fibrous structures but instead may cross-link with each other, chitin and other proteins to form a network structure [16, 39, 40]. In addition, VWA domains bind to positive ions that attract water, and may cooperate with GAGs or other proteins and provide initial hydrogel properties for biomineralization [41].

Collagens without THRs were also detected in the proteome of the skeletal organic matrix from the stony coral *S. pistillata*, and some COL6s without THRs still exist in the human and zebrafish genomes, and phylogenetic analysis indicates that COL6Ls without THR region are derived early during evolution. Together, these results indicated that COL6L or similar proteins without THRs might be the ancient proteins in early animals that gave rise to fibrillar COL1/2/4 of vertebrates. In vertebrates, fibrillar COL1 and COL2 are the major collagens in phosphate bone and cartilage tissues, respectively [42]. In *Lingula*, fibrillar COL4 (lan col4) is the main component of the phosphate shell [43], and THRs are essential for calcium phosphate skeletons [42, 44]. The later origin of THRs suggests that the emergence of phosphate skeletons might be later than carbonate skeletons, supporting the view that phosphate skeletons in most early Cambrian fossils originated from calcitic, aragonitic or organic skeletons based on detailed analysis of the preserved microstructure [1, 45]. These findings suggest that the evolution of collagens is crucial in the divergence of carbonate and phosphate skeletons and associated matrices.

Mammalian cartilage and bone matrices consist of collagen fibrils and a gel-like ground substance that is rich in chondroitin-containing proteoglycans, fibronectins

and link proteins [46]. Our results confirms the presence of fibronectin-like proteins in shells of *P. f. martensii* and *C. gigas* [6]. Proteoglycans or GAGs, which have strong water-binding capabilities and have been detected in the shell [29], may function as the gel-like substance [47]. In the nacre and the secretory cells of the mantle pallium of *P. f. martensii*, we found large amounts of acidic GAGs, which have also been detected in coral [48] and bone [49], and this finding argues that the acidic GAGs might also play key roles in crystal nucleation during nacre formation. Combining this finding with the findings on collagens, our results suggest that the nacreous shell matrix, while having a chitin-based framework, also possesses key elements of collagen-based matrices, such as collagen-like proteins, fibronectins, proteoglycans and chondroitin sulfotransferases. Chitin- and collagen-based matrices are considered as two basic types of biomineralizing framework, and our results suggest that they may have a common origin or might have co-existed as parts of an ancient/ancestral matrix with dual-elements, despite subsequent divergence in different taxa into chitin- or collagen-based organic matrices.

The shell organic matrix, rather than being a simple self-assembling structure, might instead be a complex and dynamic matrix that requires active construction, regulation and remodelling. Tyrs, which can catalyse the formation of dopa and dopaquinone, were highly abundant in the shell of bivalves, and may function in mediating intermolecular cross-links [6, 50], or as a structural component of the shell. Tyrs belong to the "type-3 copper" family and have a conserved active site of six histidine residues mediating the binding of copper ion as cofactor [51]. Metal ions such as Cu^{2+}, Zn^{2+} and Mg^{2+} are important factors for stabilizing the crystalline form of calcium carbonate [52-54]. Therefore, the deposition of Tyrs and associated metal ions in the matrix may regulate metal ion concentration in the extrapallial fluid and help to stabilize the crystalline form. Interestingly, we found that the histidine residues were retained in the 4 prism-specific Tyrs but mostly lost in the two nacre-specific Tyrs (Pm10005159 and 10016044), suggesting possible divergence in metal ion binding capability between nacre-specific and prism-specific Tyrs. It should be noted that many of expanded Tyrs may be unrelated to shell formation as shell-less *Octopus bimaculoides* also shows some expansion (Table S10), and instead they may function in their well-established roles in melanin pigment production, wound healing and immune responses in *P. f. martensii* also [55].

The complexity of the shell matrix and biomineralization processes is further demonstrated by co-expression network analysis, which indicates that genes related to polysaccharide metabolism are significantly co-expressed with nacre proteins. This result is consistent with the abundance of acid GAGs in the nacreous layer. Interestingly, nacre proteins were also co-expressed with ABC-transporters known as ATP-dependent transport proteins. ABC-transporters may mediate the secretion of proteins without signal peptide [56], which are not uncommon among nacre proteins and may be also secreted through other mechanisms such as exosomes [6]. Some nacre proteins without signal peptide may be due to assembly and annotation errors. More importantly, signal pathway related to bone formation, such as Wnt signalling pathway and osteoclast differentiation signalling pathway, were also implicated. Together, these results suggest that molluscan shell formation is an elaborate and dynamic process that shares certain basic elements with mammalian bone formation, but with added complexity. Although molluscan shells have a chitin-dominated framework, the identification of key elements of collagen-based matrices supports a single origin for the two types of matrices or a common set of tools that may have been lost, modified and reorganized during evolution to produce diverse forms of biomineralized structures in adaptation to new environments and in assuming new functions.

In conclusion, we sequenced and assembled the highly polymorphic genome of *P. f. martensii* using NGS and the BAC-to-BAC strategy. Based on genomic, transcriptomic, and proteomic analyses and experimental studies, we identified a large number of genes related to shell nacre formation, which helped us to re-construct the shell matrix model (Fig. 5). The identification of COL6Ls without THRs and other elements of collagen-based matrices in the chitin-rich nacre matrix not only supports the homology and single evolutionary origin of the common biomineralization toolkit, but also provides evidence that changes in collagen may underlie the divergence of the two principal classes of skeletal biominerals: calcium carbonate and calcium phosphate. The hypothesis of a single evolutionary origin challenges the prevailing idea of independent evolution [2] and may stimulate homology-based studies towards a better understanding of the diverse forms of biomineralization.

Methods

SI Appendix has additional information relating to the methodologies described

below.

Library construction and sequencing. We constructed all sequencing libraries according to protocols from Illumina and sequenced these libraries on a HiSeq 2000 sequencing system.

Hierarchical BAC-to-BAC assembly strategy. We used a hierarchical BAC-to-BAC assembly approach as used for the moth genome [11]. Before the hierarchical assembly of BACs, we used SOAPdenovo to assemble the reads of each BAC with odd numbered K-mers from 27 to 63 and selected the best results with the longest scaffold N50 and total length, as primary scaffolds. Then, we used the paired-end reads information of the BACs and locally assembled the reads in the gap regions to fill in the gaps within the primary BAC scaffolds. Our custom assembly software (Rabbit) [11] was used to assemble scaffolds of BACs with large overlaps. After finding relationship among sequences, merging overlapping sequences and removing redundant sequences, we obtained longer segments as secondary scaffolds. Finally, SSPACE was used to join the secondary scaffolds to form final scaffolds, and SOAP-Gapcloser was used to fill in the gaps in the final scaffolds using all WGS reads with short insert sizes.

Linkage group construction. We constructed a genetic map using RAD-seq of 148 F1 progeny from a family obtained by crossing two genetically distant parents. We used SOAP2 [57] to map the reads to the reference genome sequences of *P. f. martensii* (scaffolds) and performed SNP calling using SOAPsnp [58]. After SNP calling, we extracted genotypes by combining all SNPs among the 148 progeny and the 2 parents and constructed linkage map using JoinMap 4.1 [59].

Phylogenetic tree construction and divergence time estimation. We used Treefam to obtain gene families and one-to-one orthologs, and used MrBayes to construct the phylogenetic tree.

Transcriptome analysis. We extracted total RNA from each sample and isolated mRNA using oligo (dT) magnetic beads. Then, the mRNA was fragmented into short fragments (200~500 bp) for construction of RNA-seq libraries that were sequenced on an Illumina HiSeq2000. Using SOAP2, all clean reads were mapped to the genome assembly with less than 5 mismatches. We used the *RPKM* method (Reads per kilobase transcript per million mapped reads) to calculate the gene expression levels. We also tested TPM (Transcripts Per Million) [60] for quantifying gene expression and found excellent correspondence between RPKM and TRM for our samples.

Identification of the matrix proteins. We used the Mascot software (v 2.3.02) to query the MS/MS spectra data of matrix proteins in the database. We applied the trypsin cleavage rule with one missed cleavage site. Carbamidomethylation of cysteines was considered as the fixed modifications while Gln->pyro-Glu (N-term Q), Oxidation (M) and Deamidated (NQ) were considered as the variable modifications. Peptide mass tolerance was set to 0.05Da and fragment mass tolerance was set to 0.01Da. We used target-decoy search strategy [61] to identify the matrix proteins, and the False Discovery Rate (PDR) was ≤1%.

Extraction of matrix proteins from the nacre and prismatic layer. Shells of freshly collected oysters were thoroughly cleaned by hand and treated with sodium hypochlorite solution (6-14% active chlorine) to remove organic surface contaminants [62]. The prismatic layer was separated from the edges of pearl oyster shells without nacre. The nacre was directly scraped from the internal shell surfaces dominated by aragonite. These samples were thoroughly ground and soaked in acetic acid solution (5%, v/v) for at least 12 h to dissolve calcium carbonate, before being centrifuged at 14,000 g and 4 °C for 1 h. Acid-soluble proteins were in the supernatant, and acid-insoluble proteins were in the residue.

Samples were electrophoresed on 12% polyacrylamide gels and stained with Coomassie blue R-250. The extracted peptides were dried and stored at -80 °C until liquid chromatography/tandem mass spectrometry (LC-MS/MS) analysis.

Chitin identification in shell matrix. We decalcified the shells in 1 M acetic acid at 4 °C for one week, and the acid-insoluble material was collected. This insoluble material was washed with distilled water and embedded in paraffin for sectioning. The sections were placed on slides and stained for 5 min with 0.1% Calcofluor White M2R (Flupstain Ⅰ) (Sigma-Aldrich). Excess dye was rinsed off with distilled water. The stained specimens were observed under a confocal laser microscope using filters with 492 nm excitation and 520 nm emission [63].

RNAi experiment. The primers used for generating the *COL6L* double-strand RNA (dsRNA) are shown in Additional file 1: Table S16. DsRNAs were synthesized following the method of Suzuki et al. [64], and injected into the adductor muscle every 4 days at 100 μg per 100 μl per pearl oyster each time. The effects of RNAi of the six *COL6L* genes on nacre formation were detected by SEM.

Identification of GAGs in shell and pearl. Shells were decalcified in 1 M acetic acid

at 4 °C for 1 week and then in 10% EDTA-2Na solution at room temperature for 10 days. The fixed materials were embedded in paraffin and stained with AB/PAS (Alcian blue/periodic acid-Schiff) and observed under a OlympusBX51 optical microscope.

Nitrobluetetrazolium (NBT)/glycinate assay for dopa and dopaquione protein. Sections of decalcified shells were stained with 100 μL of solution containing 0.24 mM NBT and 2 M potassium glycinate (pH10) for nearly 5 min in darkness until violet positive signals appeared [65]. The sections were rinsed with double-distilled water to stop the reaction and then mounted for microscopic examination.

Co-expression network analysis. We used WGCNA to reconstruct the co-expression network for biomineralization [66]. A weighted correlation network was constructed between all pairs of genes across four mantle tissue samples [67]. The adjacency matrix was calculated through a so-called 'soft' thresholding framework (power β=9) that converted the co-expression measure to a connection weight. Based on the adjacency matrix, we implemented a topological overlap dissimilarity measure to reflect relative inter-connectedness, which may represent a meaningful biological network. Hub genes (highly connected genes), by definition, tend to have high connectivity in the constructed network.

Availability of supporting data

Data from the pearl oyster (*P. f. martensii*) genome projects have been deposited at DDBJ/EMBL/GenBank under the accession number XXX.

List of abbreviations

CHS: chitin synthases; COL6: collagen VI; BMPs: bone morphogenetic proteins; VWA: von Willebrand factor A; CHST11: chondroitin 4-sulfotransferase 11; CHST3: chondroitin 6-sulfotransferase 3; CHST6: carbohydrate 6-sulfotransferase 6; CHST9: carbohydrate 4-sulfotransferase 9; D4ST1: dermatan 4-sulfotransferase 1; Tyr: Tyrosinase; WGCNA: Weighted-gene co-expression network analysis;

Competing interests

The authors declare that they have no competing interests.

Funding

This research is partly supported the Guangdong Ocean University Nature Science Foundation (University program: Genome studies of pearl oyster), the National Nature Science Foundation of China (31272635, 31372526, 41206141), Modern Agro-industry Technology Research System (CARS-48), USDA/NJAES Project

1004475/NJ32920 and "Taishan Oversea Scholar" program.

Authors' contributions

X.D., G.Z., and X.G. designed the study and its scientific objectives. Y.J, R.H. W.C and G.F. managed the project. C.B., F.S., X.L., C.S., W.L. and Z.W. performed genome assembly, gene annotation and evolution analysis. Y.D. and Q.W. cultured *P. f. martensii* and provided materials. Y.D., Q.W., F.S., J.B. and Z.W. constructed the genetic map. Q.W., Z.Z., and R.H. performed the acid GAG analysis. Y.J., R.H., Z.W., Z.Z, J.L. performed the chitin and COL6L related analysis. R.H. and C.B. performed the tyrosinase related analysis. Z.Z and H.Z. performed the WGCNA analysis. X.D., G.F. and X.G. directed final data analyses. X.L., Q.S, X.D., G.F, Y.J., H.Z., X.G., R.H., C.B, Y.D, Q.W., Z.Z. did most of the writing with contributions from all authors.

Acknowledgments

We thank Z. He, W. Liu, Z. Wu, C. Liu, J. Jian, B. Tan for their supports of the pearl oyster genome project. We thank Y. Guo, X. Chen, M. Xue and Xuwen Pearl Oyster Farm for assistance with DNA, RNA and protein extraction, data analysis and oyster culture. We thank L. Goodman for helping to edit the manuscript. We thank other faculty and staff at Guangdong Ocean University, BGI-Shenzhen and Rutgers who contributed to the genome project.

Author Information

Correspondence and requests for materials should be addressed to X.D. (zjduxd@126.com), X.G. (xguo@hsrl.rutgers.edu), G.Z. (gzhang@qdio.ac.cn) and X.L. (liuxin@genomics.cn).

References

1. Knoll AH. Biomineralization and evolutionary history. Rev Mineral Geochem. 2003;54:329-56.
2. Drake JL, Mass T, Falkowski PG. The evolution and future of carbonate precipitation in marine invertebrates: Witnessing extinction or documenting resilience in the Anthropocene? Elementa: Science of the Anthropocene. 2014;2:000026.
3. Ehrlich H. Chitin and collagen as universal and alternative templates in biomineralization. Int Geol Rev. 2010;52:661-99.
4. Furuhashi T, Schwarzinger C, Miksik I, Smrz M, Beran A. Molluscan shell evolution with review of shell calcification hypothesis. Comp Biochem Phys B. 2009;154:351-71.
5. Addadi L, Joester D, Nudelman F, Weiner S. Mollusk shell formation: a source of new concepts for understanding biomineralization processes. Chem-Eur J. 2006;12:980-7.
6. Zhang G, Fang X, Guo X, Li L, Luo R, Xu F, et al. The oyster genome reveals stress adaptation and complexity of shell formation. Nature. 2012;490:49-54.
7. Mount AS, Wheeler A, Paradkar RP, Snider D. Hemocyte-mediated shell mineralization in the eastern oyster. Science. 2004;304:297-300.
8. Marin F, Luquet G, Marie B, Medakovic D. Molluscan shell proteins: primary structure, origin, and evolution. Curr Top Dev Biol. 2007;80:209-76.
9. Murdock DJ, Donoghue PC. Evolutionary origins of animal skeletal biomineralization. Cells Tissues Organs. 2011;194:98-102.
10. Takeuchi T, Kawashima T, Koyanagi R, Gyoja F, Tanaka M, Ikuta T, et al. Draft genome of the pearl oyster *Pinctada fucata*: a platform for understanding bivalve biology. DNA Res. 2012;19:117-30.
11. You M, Yue Z, He W, Yang X, Yang G, Xie M, et al. A heterozygous moth genome provides insights into herbivory and detoxification. Nature genetics. 2013;45:220-5.
12. Murgarella M, Puiu D, Novoa B, Figueras A, Posada D, Canchaya C. A First Insight into the Genome of the Filter-Feeder Mussel *Mytilus galloprovincialis*. Plos One. 2016;11:e0151561.
13. Zhang H, Ahmad M, Gronowicz G. Effects of transforming growth factor-beta 1 (TGF-β1) on in vitro mineralization of human osteoblasts on implant materials. Biomaterials. 2003;24:2013-20.
14. Miron RJ, Saulacic N, Buser D, Iizuka T, Sculean A. Osteoblast proliferation and differentiation on a barrier membrane in combination with BMP2 and TGFβ1. Clin Oral Invest. 2013;17:981-8.
15. Yan F, Luo S, Jiao Y, Deng Y, Du X, Huang R, et al. Molecular characterization of the BMP7 gene and its potential role in shell formation in *Pinctada martensii*. Int J Mol Sci. 2014;15:21215-28.
16. Becker A-KA, Mikolajek H, Paulsson M, Wagener R, Werner JM. A structure of a collagen VI VWA domain displays N and C termini at opposite sides of the protein. Structure. 2014;22:199-208.

17. Nudelman F, Shimoni E, Klein E, Rousseau M, Bourrat X, Lopez E, et al. Forming nacreous layer of the shells of the bivalves Atrina rigida and *Pinctada margaritifera*: an environmental-and cryo-scanning electron microscopy study. J Struct Biol. 2008;162:290-300.
18. Luo R, Liu B, Xie Y, Li Z, Huang W, Yuan J, et al. SOAPdenovo2: an empirically improved memory-efficient short-read de novo assembler. Gigascience. 2012;1:18.
19. Myers EW, Sutton GG, Delcher AL, Dew IM, Fasulo DP, Flanigan MJ, et al. A whole-genome assembly of Drosophila. Science. 2000;287:2196-204.
20. Stanke M, Keller O, Gunduz I, Hayes A, Waack S, Morgenstern B. AUGUSTUS: ab initio prediction of alternative transcripts. Nucleic Acids Res. 2006;34:W435-9.
21. Elsik CG, Mackey AJ, Reese JT, Milshina NV, Roos DS, Weinstock GM. Creating a honey bee consensus gene set. Genome Biol. 2007;8:R13.
22. Cantarel BL, Korf I, Robb SM, Parra G, Ross E, Moore B, et al. MAKER: an easy-to-use annotation pipeline designed for emerging model organism genomes. Genome Res. 2008;18:188-96.
23. Simao FA, Waterhouse RM, Ioannidis P, Kriventseva EV, Zdobnov EM. BUSCO: assessing genome assembly and annotation completeness with single-copy orthologs. Bioinformatics. 2015;31:3210-2.
24. Addadi L, Weiner S. Biomineralization: A pavement of pearl. Nature. 1997;389:912-5.
25. Nassif N, Pinna N, Gehrke N, Antonietti M, Jäger C, Cölfen H. Amorphous layer around aragonite platelets in nacre. P Natl Acad Sci USA. 2005;102:12653-5.
26. Levi-Kalisman Y, Falini G, Addadi L, Weiner S. Structure of the nacreous organic matrix of a bivalve mollusk shell examined in the hydrated state using cryo-TEM. J Struct Biol. 2001;135:8-17.
27. Weiss IM, Schönitzer V. The distribution of chitin in larval shells of the bivalve mollusk *Mytilus galloprovincialis*. J Struct Biol. 2006;153:264-77.
28. Furuhashi T, Beran A, Blazso M, Czegeny Z, Schwarzinger C, Steiner G. Pyrolysis GC/MS and IR spectroscopy in chitin analysis of molluscan shells. Biosci Biotech Bioch(BBB). 2009;73:93-103.
29. Pereira- Mouriès L, Almeida MJ, Ribeiro C, Peduzzi J, Barthélémy M, Milet C, et al. Soluble silk- like organic matrix in the nacreous layer of the bivalve *Pinctada maxima*. Eur J Biochem. 2002;269:4994-5003.
30. Sudo S, Fujikawa T, Nagakura T, Ohkubo T, Sakaguchi K, Tanaka M, et al. Structures of mollusc shell framework proteins. Nature. 1997;387:563-4.
31. Nudelman F, Chen HH, Goldberg HA, Weiner S, Addadi L. Spiers Memorial Lecture Lessons from biomineralization: comparing the growth strategies of mollusc shell prismatic and nacreous layers in *Atrina rigida*. Faraday discussions. 2007;136:9-25.
32. Weiner S, Hood L. Soluble protein of the organic matrix of mollusk shells: a potential template for shell formation. Science. 1975;190:987-9.

33. Fu G, Valiyaveettil S, Wopenka B, Morse DE. CaCO3 biomineralization: acidic 8-kDa proteins isolated from aragonitic abalone shell nacre can specifically modify calcite crystal morphology. Biomacromolecules. 2005;6:1289-98.
34. Evans JS. “Tuning in” to Mollusk Shell Nacre-and Prismatic-Associated Protein Terminal Sequences. Implications for Biomineralization and the Construction of High Performance Inorganic− Organic Composites. Chem Rev. 2008;108:4455-62.
35. Li S, Liu Y, Liu C, Huang J, Zheng G, Xie L, et al. Hemocytes participate in calcium carbonate crystal formation, transportation and shell regeneration in the pearl oyster *Pinctada fucata*. Fish Shellfish Immun. 2016;51:263-70.
36. Liu C, Li S, Kong J, Liu Y, Wang T, Xie L, et al. In-depth proteomic analysis of shell matrix proteins of *Pinctada fucata*. Sci Rep-UK. 2015;5.
37. Kadler KE, Baldock C, Bella J, Boot-Handford RP. Collagens at a glance. J Cell Sci. 2007;120:1955-8.
38. Van der Rest M, Garrone R. Collagen family of proteins. Faseb J. 1991;5:2814-23.
39. Fitzgerald J, Mörgelin M, Selan C, Wiberg C, Keene DR, Lamandé SR, et al. The N-terminal N5 subdomain of the α3 (VI) chain is important for collagen VI microfibril formation. J Biol Chem. 2001;276:187-93.
40. Suhre MH, Gertz M, Steegborn C, Scheibel T. Structural and functional features of a collagen-binding matrix protein from the mussel byssus. Nat Commun. 2014;5:3392.
41. Whittaker CA, Hynes RO. Distribution and evolution of von Willebrand/integrin A domains: widely dispersed domains with roles in cell adhesion and elsewhere. Mol Biol Cell. 2002;13:3369-87.
42. Boot- Handford RP, Tuckwell DS. Fibrillar collagen: the key to vertebrate evolution? A tale of molecular incest. Bioessays. 2003;25:142-51.
43. Aouacheria A, Geourjon C, Aghajari N, Navratil V, Deléage G, Lethias C, et al. Insights into early extracellular matrix evolution: spongin short chain collagen-related proteins are homologous to basement membrane type IV collagens and form a novel family widely distributed in invertebrates. Mol Biol Evol. 2006;23:2288-302.
44. Brodsky B, Persikov AV. Molecular structure of the collagen triple helix. Adv Protein Chem. 2005;70:301-39.
45. Kouchinsky A. Shell microstructures in Early Cambrian molluscs. Acta Palaeontol Pol. 2000;45.
46. Heinegård D, Oldberg A. Structure and biology of cartilage and bone matrix noncollagenous macromolecules. Faseb J. 1989;3:2042-51.
47. Mow VC, Ratcliffe A, Poole AR. Cartilage and diarthrodial joints as paradigms for hierarchical materials and structures. Biomaterials. 1992;13:67-97.
48. Goldberg WM. Acid polysaccharides in the skeletal matrix and calicoblastic epithelium of the stony coral Mycetophyllia reesi. Tissue Cell.

2001;33:376-87.

49. Vejlens L. Glycosaminoglycans of human bone tissue. Calcified Tissue Research. 1971;7:175-90.

50. Aguilera F, McDougall C, Degnan BM. Evolution of the tyrosinase gene family in bivalve molluscs: Independent expansion of the mantle gene repertoire. Acta Biomater. 2014;10:3855-65.

51. Decker H, Schweikardt T, Tuczek F. The first crystal structure of tyrosinase: all questions answered? Angew Chem Int Edit. 2006;45:4546–50.

52. Kitano Y, Kanamori N, Yoshioka S. Adsorption of zinc and copper ions on calcite and aragonite and its influence on the transformation of aragonite to calcite. Geochem J. 1976;10:175-9.

53. Berner R. The role of magnesium in the crystal growth of calcite and aragonite from sea water. Geochim Cosmochim Ac. 1975;39:489-504.

54. Nassrallah-Aboukais N, Boughriet A, Laureyns J, Aboukais A, Fischer J, Langelin H, et al. Transformation of vaterite into cubic calcite in the presence of copper (II) species. Chem Mater. 1998;10:238-43.

55. Kanteev M, Goldfeder M, Fishman A. Structure–function correlations in tyrosinases. Protein Sci. 2015;24:1360-69.

56. Higgins CF. ABC transporters: from microorganisms to man. Cell and Developmental Biology. 1992;8:67-113.

57. Hecker A, Mikulski Z, Lips KS, Pfeil U, Zakrzewicz A, Wilker S, et al. Pivotal Advance: Up-regulation of acetylcholine synthesis and paracrine cholinergic signaling in intravascular transplant leukocytes during rejection of rat renal allografts. J Leukocyte Biol. 2009;86:13-22.

58. Li R, Li Y, Fang X, Yang H, Wang J, Kristiansen K, et al. SNP detection for massively parallel whole-genome resequencing. Genome Res. 2009;19:1124-32.

59. Van Ooijen J. Multipoint maximum likelihood mapping in a full-sib family of an outbreeding species. Genet Res. 2011;93:343-9.

60. Wagner GP, Kin K, Lynch VJ. Measurement of mRNA abundance using RNA-seq data: RPKM measure is inconsistent among samples. Theor Bios. 2012;131:281-5.

61. Elias JE, Gygi SP. Target-decoy search strategy for increased confidence in large-scale protein identifications by mass spectrometry. Nat Methods. 2007;4:207-14.

62. Mann K, Edsinger-Gonzales E, Mann M. In-depth proteomic analysis of a mollusc shell: acid-soluble and acid-insoluble matrix of the limpet *Lottia gigantea*. Proteome Sci. 2012;10:1.

63. Su X, Matthay MA, Malik AB. Requisite role of the cholinergic α7 nicotinic acetylcholine receptor pathway in suppressing gram-negative sepsis-induced acute lung inflammatory injury. J Immunol. 2010;184:401-10.

64. Suzuki M, Saruwatari K, Kogure T, Yamamoto Y, Nishimura T, Kato T, et al. An acidic matrix protein, Pif, is a key macromolecule for nacre formation. Science. 2009;325:1388-90.

65. Paz M, Flückiger R, Boak A, Kagan H, Gallop PM. Specific detection of quinoproteins by redox-cycling staining. J Biol Chem. 1991;266:689-92.
66. Langfelder P, Horvath S. WGCNA: an R package for weighted correlation network analysis. BMC Bioinformatics. 2008;9:559.
67. Zhang B, Horvath S. A general framework for weighted gene co-expression network analysis. Stat Appl Genet Mol. 2005;4:1128.

Figure legend

Figure 1. Genome organization of *P. f. martensii*.

a. Genetic map of *P. f. martensii* constructed with RAD single-nucleotide polymorphisms (SNPs). The lines on linkage groups represent SNP positions. **b.** The distribution of GC, gene, repetitive elements and SNPs on *P. f. martensii* pseudochromosomes. **c.** Synteny blocks between *C. gigas* (Cg) and *P. f. martensii* (PIN).

Figure 2. Expression and structure analysis of COL6Lgenes in *P. f. martensii*.

a. Expression of six COL6L genes implicated in nacreous shell formation showing higher expression in the mantle pallium (MP) and pearl sac (PS) than in other organs. Y-axis represents the normalized RPKM value. X-axis represents nine organs (MP, mantle pallium; ME, mantle edge; A, adductor muscle; He, hepatopancreas; BC, hemocyte; Go, gonad; Gi, gill; F, foot; PS, pearl sac at 180 days after nucleus transplantation). **b.** Expression of six COL6L genes during early development with *COL6L*-Pma_10019835 up-regulated at the trochophore (T) stage and all highly expressed at and after the post-veliger (PV) stage, corresponding to active adult shell formation. E, egg; Fe, fertilization; B, blastula; G, gastrula; ET, early trochophore; T, trochophore; D, D-larvae; DF, D-shaped larvae before feeding; EU, early umbo larvae; U, eye-larvae; PV, post-veliger; J, juveniles. **c.** Left: Disordered microstructure of nacre observed after inhibition of two *COL6L* genes (Pma_10015641 and Pma_44.543) with RNA interference (bar = 5 um). Right: Relative expression of Pma_10015641 and Pma_44.534 in mantle under RNAi: PBS, control; RPF, red fluorescent protein; dsRNA, RNAi. **d.** Structural analysis of COL6L genes from *P. f. martensii* (Pma), *C. gigas* (Cgi), *L. gigantea* (Lgi), *A. queenslandica* (Aqu), *A. digitifera* (Adi), *H. sapiens* (Hsa) and *D. rerio* (Dre). P, G, L, Aq and Ad are various unique domains found in different taxa. **e.** Phylogenetic analysis of collagen proteins. Genes in green are the COL6L genes found in the shell matrix proteome of *P. f.*

martensii. Genes shaded in pink are collagens without triple-helix regions (THRs), and genes shaded in blue are COL6 and COL1/2/4 with THRs.

Figure 3. GAGs and tyrosinase genes in *P. f. martensii*.

a. The shell matrix extracted from the nacre of *P. f. martensii* contains abundant acid glycosaminoglycans (GAGs) stained blue (I), whereas matrices extracted from the prismatic layer of *P. f. martensii* (I) and *C. gigas* (II) contain neutral GAGs stained red. Secretory cells (arrow) in the mantle pallium of *P. f. martensii* are filled with acid GAGs stained blue (III), whereas cells in the mantle pallium of *C. gigas* contains neutral GAGs stained red (IV). **b.** Expression (y-axis) of *CHST3*, *CHST11*, *CHST6* and *D4ST1* genes in the mantle pallium (MP) and the mantle edge (ME). **c.** Phylogenetic tree of the tyrosinase proteins from *P. f. martensii* and *C. gigas*. Tyrosinase genes specifically expanded in *P. f. martensii* are shaded in purple, and their expression patterns during early development are presented in the heat map. E, egg; Fe, fertilization; B, blastula; G, gastrula; ET, early trochophore; T, trochophore; D, D-shaped larvae; DF, D-larvae before feeding; EU, early umbo larvae; U, eye-larvae; PV, post-veliger; J, juveniles.

Figure 4. Co-expression network of nacre formation-related genes of *P. f. martensii*.

Hub genes are illustrated in the internal circle, where connections among them are coloured red. The number of visible links for each hub gene is represented by the size of the node. Links and their corresponding hub genes are in the same colour.

Figure 5. A model of nacre formation in *P. f. martensii*.

In this model, new nacre (N) is formed in an organic matrix secreted by haemocytes or epithelial (Ep) cells beneath the mature nacre (M). Chitin provides the core of the polymer framework of the organic matrix. COL6 with chitin-binding domains binds to chitin and interacts with fibronectins and other COL6 proteins, forming the matrix networks. Asp-rich acid glycoproteins and acid GAGs function as the hydrogel substances. Tyrs catalyse the oxidation of tyrosine and dopamine and function in cross-linking and shell matrix maturation. Protease inhibitors, proteases and other enzymes regulate the biosynthesis or degradation of the organic matrix.

Additional file 2: Figure S1. Sequencing date and k-mer analysis.

a. The distribution of 17-mer depth derived from WGS sequence reads. X-axis is the K-mer depth and the Y-axis is the percentage of each K-mer depth. The first peak is

created by sequence polymorphism and its relative height provides a measure of heterozygosity in the diploid genome. **b.** The heterozygous ratio of oyster genome estimated by k-mer analysis (left). The sequencing depth obtained by WGS reads mapped against assembly and GC content of our genome (right). **c.** The assembled length of the BACs of four pooling libraries. Four libraries were randomly selected and the total length of each assembly was calculated.

Additional file 3: Figure S2. Assembly coverage of BACs.

Sequencing depth on the BACs was calculated by mapped sequence reads. The annotated transposable elements (TEs) are shown in black or red, and the remaining unclosed gaps on the scaffolds are marked as white blocks.

Additional file 4: Figure S3. The analysis of codon usage and GC content

a. Comparison of the distribution of codon usage among 9 species. **b.** The GC content distribution for each codon position. **c.** The GC content distribution of exon, intron and inter-genetic regions.

Additional file 5: Figure S4. Phylogenetic analysis and gene clustering.

a. Species tree of *P. f. martensii* and 6 selected species. The number is the divergence time of the clades with ranges in parenthesis. **b.** Unique and shared gene families between *P. f. martensii* (*P. mar*) and other three species including *Crassostrea gigas* (*C. gig*), *Lottia gigantea* (*L. gig*) and *Homo sapiens* (*H. sap*).

Additional file 6: Figure S5. Phylogenetic analysis of TGF-β1/2/3 and bone morphogenetic proteins (BMP) from different species.

Proteins and accession numbers are listed in *SI Appendix*, Table S12.

Additional file 7: Figure S6. CHS and chitinase genes in *P. f. martensii*.

a. Chitin in the shell matrix of *P. f. martensii* and *C. gigas* stained green with Calcofluor White M2R. **b.** Expression of *CHS* in different organs. One *CHS* (Pma_10008435) is highly expressed in both the mantle pallium and the pearl sac. **c.** Expression of *chitinase* in mantle pallium (MP), mantle edge (ME) and in pearl sac (PS), compared with non-calcifying tissues (including A, adductor muscle; He, hepatopancreas; BC, hemocyte; Go, gonad; Gi, gill; F, foot). **d.** Expression of *Chitinases* and *CHS* at different developmental stages of *P. f. martensii*. Most of the *chitinases* are highly expressed at T and PV stages. The expression of one *CHS* (Pma_10008435) gene, which is highly expressed both in mantle pallium and pearl sac, is also induced at T and PV stages. E, egg; Fe, fertilization; B, blastula; G, gastrula; ET, early trochophore; T, trochophore; D, D-shaped larvae; DF, D-larvae

before feeding; EU, early umbo larvae; U, eye-larvae; PV, post-veliger; J, juveniles.

Additional file 9: Figure S7. COL6L RNAi analysis in *P. f. martensii.*

Knockdown of four *COL6Ls* with RNAi. The expression profiles of four *COLL6* genes in the mantle, Pma_530.149, Pma_10019835, Pma_10019836 and Pma_1011175, were determined using real-time quantitative PCR, with GAPDH as the internal reference gene. *COL6Ls* were significantly inhibited in the treatment group ($P < 0.05$). SEM images of the surface of the nacre from *P. f. martensii* injected with PBS and 100 µg RFP (red fluorescent protein) dsRNA demonstrated a normal growth status of nacre formation, whereas *P. f. martensii* in the treatment group injected with Pma_530.149, Pma_10019835, Pma_10019836 and Pma_1011175 dsRNA showed disruptions in crystal growth during nacre formation.

Additional file 10: Figure S8. Tyrosinases and sulfotransferases in *P. f. martensii.*

a. Expression of *sulfotransferase* genes in early development. *CHST11* (Pma_133.4) and *D4ST1* (Pma_10006752) showed expression at the PV stage, whereas *CHST6* (Pma_279.110) and *CHST3* (Pma_10022575) were mostly up-regulated at the T stage. E, egg; Fe, fertilization; B, blastula; G, gastrula; ET, early trochophore; T, trochophore; D, D-shaped larvae; DF, D-larvae before feeding; EU, early umbo larvae; U, eye-larvae; PV, post-veliger; J, juveniles. **b, c.** *Tyr* expression in the mantle and pearl sac, respectively, compared with other non-calcifying tissues (including A, adductor muscle; He, hepatopancreas; BC, hemocyte; Go, gonad; Gi, gill; F, foot). *Tyrs* that were highly expressed in mantle pallium (MP) or mantle edge (ME) are shown in b, the different cycles represent different *Tyrs* (inside-out: Pma_10005159, Pma_10013533, Pma_10015392, Pma_10016044, Pma_10021421, Pma_10021422, Pma_10022578, Pma_10001525, Pma_10004452, Pma_10005803, Pma_10013532, Pma_10014430, Pma_10015306, Pma_10018719, Pma_10018775, Pma_10021425, Pma_10024726, Pma_10028201, Pma_10028307, Pma_10028311). Expression of *Tyrs* in pearl sac (PS) compared with other non-calcifying tissues are presented in c, with nine *Tyrs* highly expressed in PS marked with red frame. **d.** Abundance of quinoproteins (stained purple) in the nacre matrix revealed by a NBT/glycinate assay. Triangle represented prismatic layer and arrows represented nacreous layer.

Figure 1

Click here to download Figure Fig 1-06-01.tif

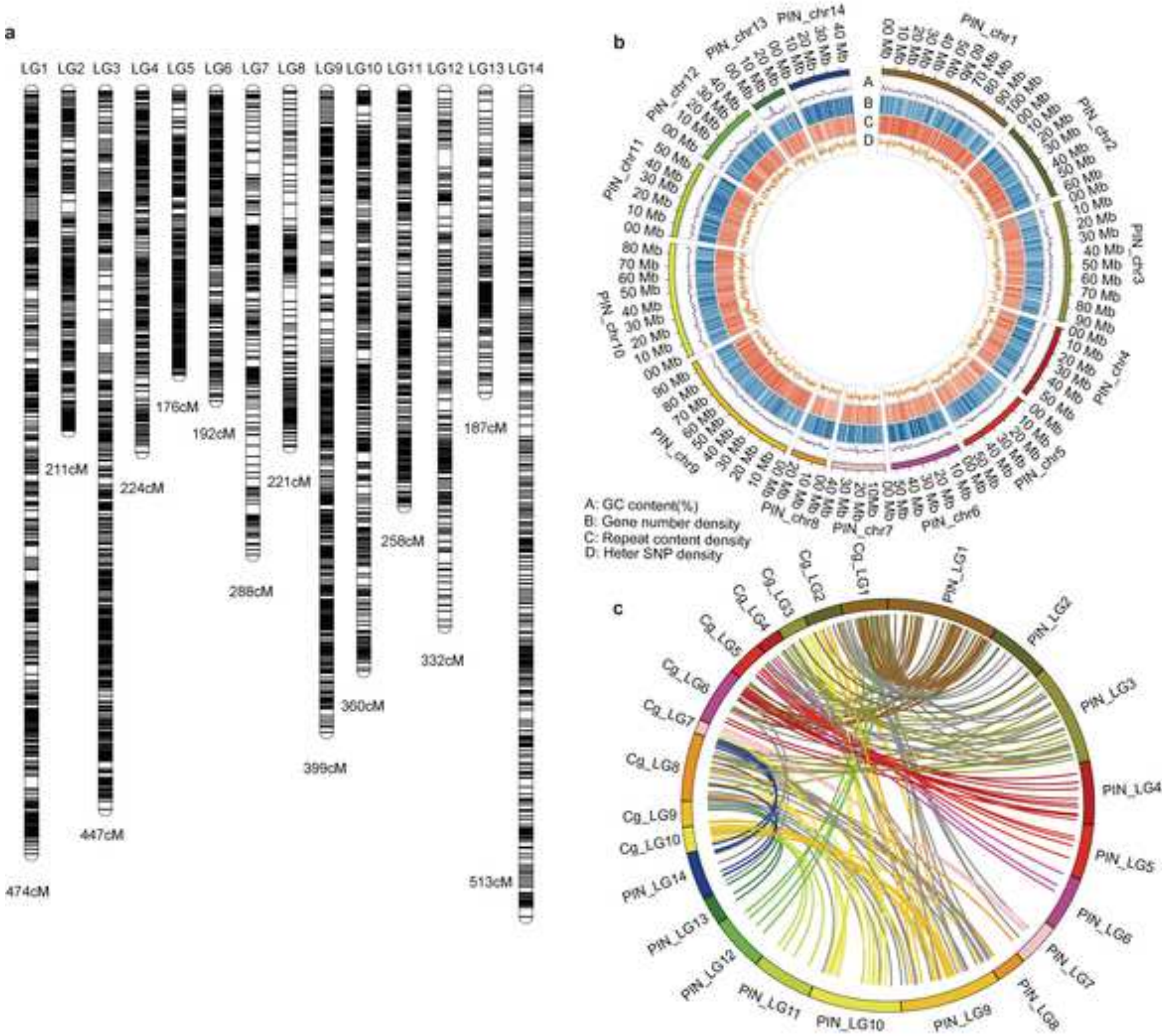

Figure 2

Click here to download Figure Fig.2_1-01.tif

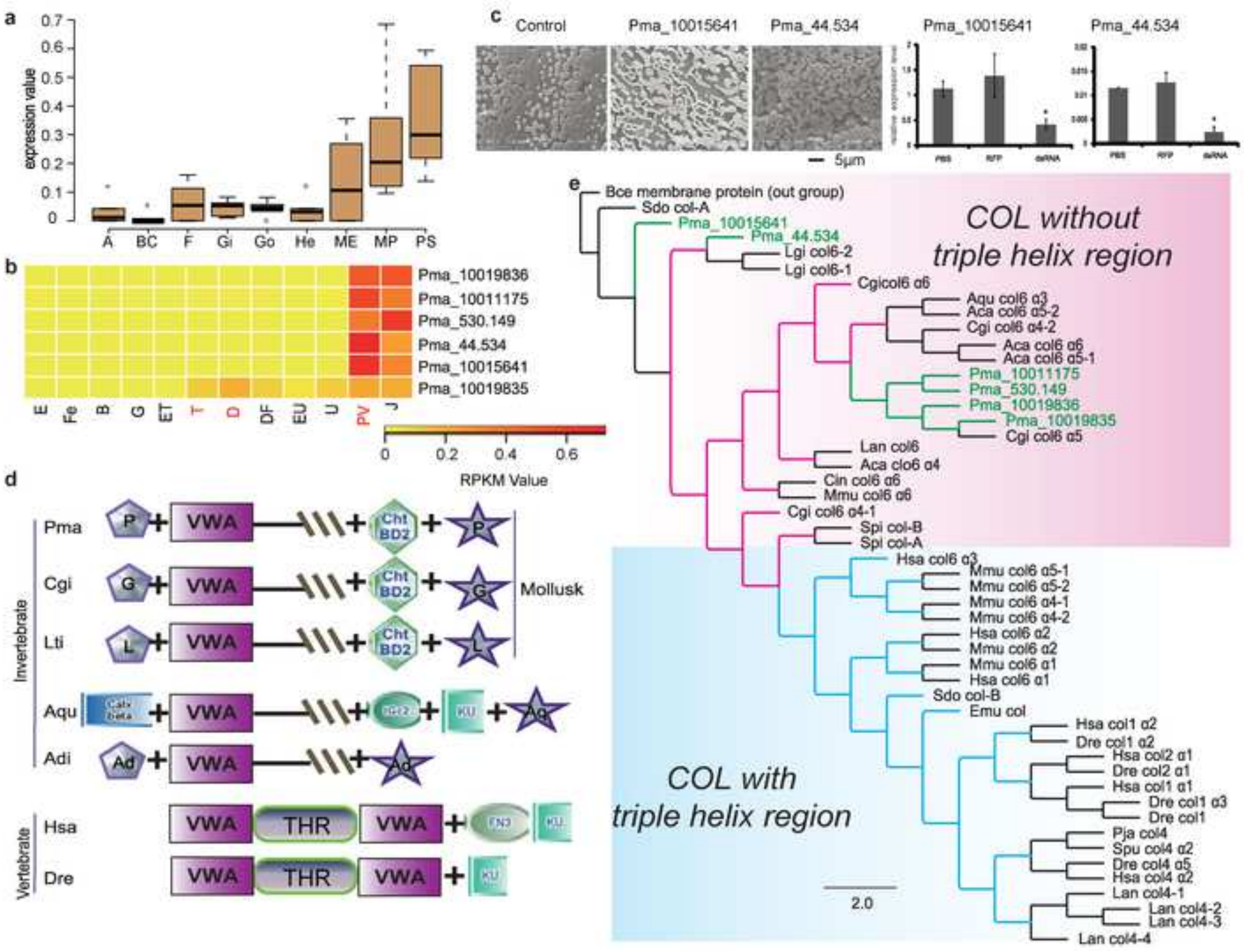

Figure 3 Click here to download Figure Fig.3-01.tif

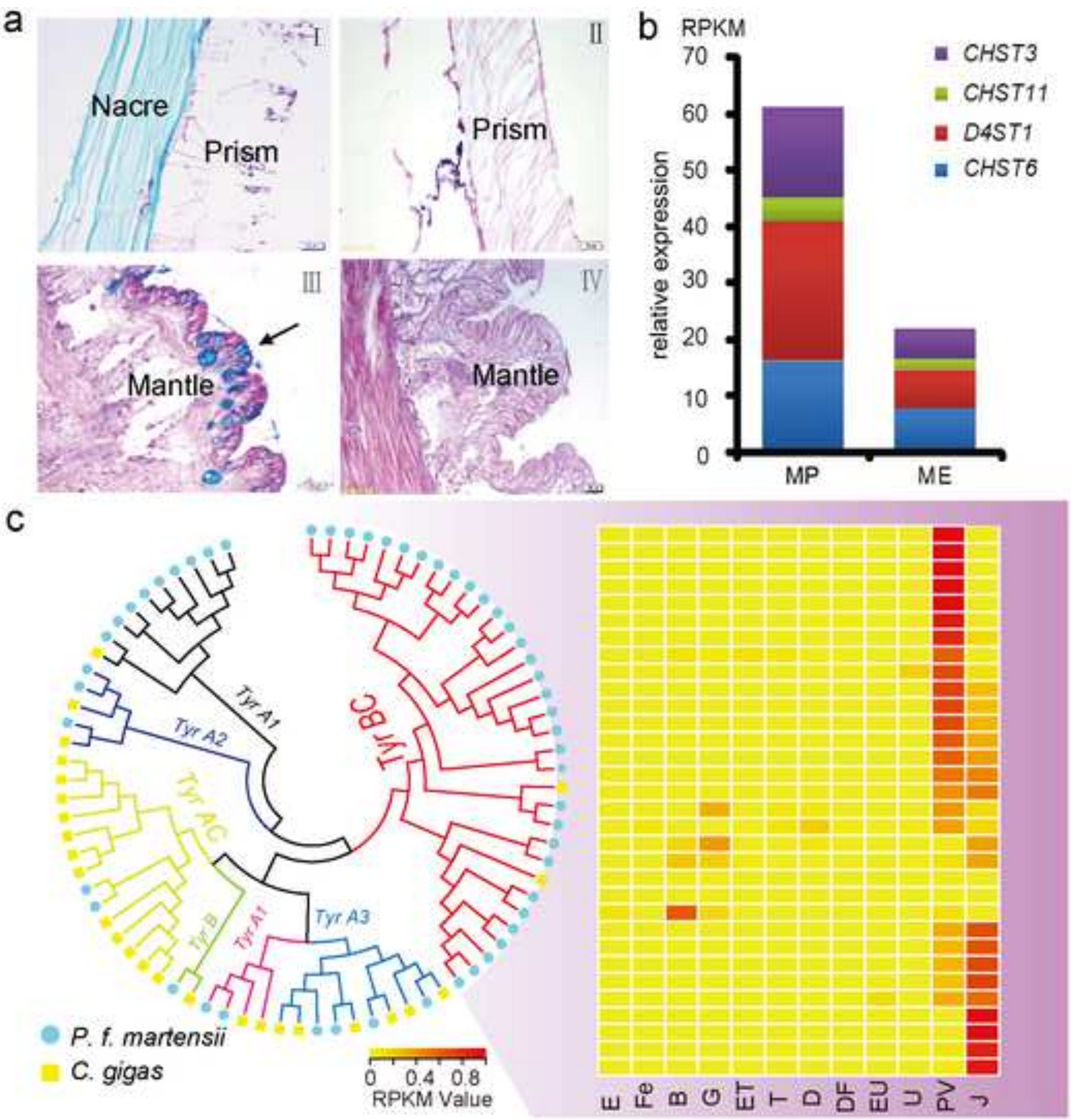

Figure 4

Click here to download Figure Fig.4-170106-01.tif

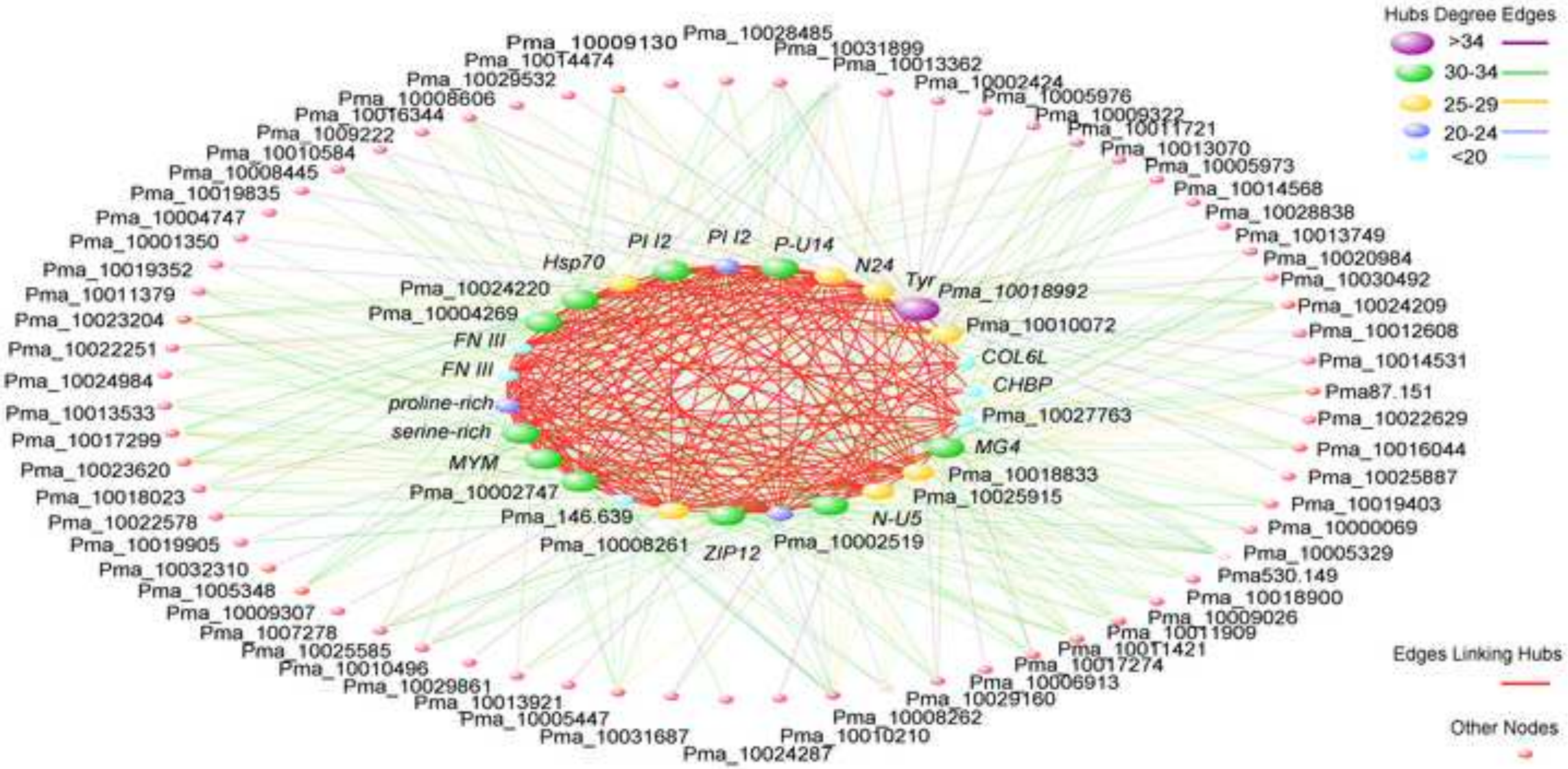

Figure 5

Click here to download Figure Fig.5170406.tif

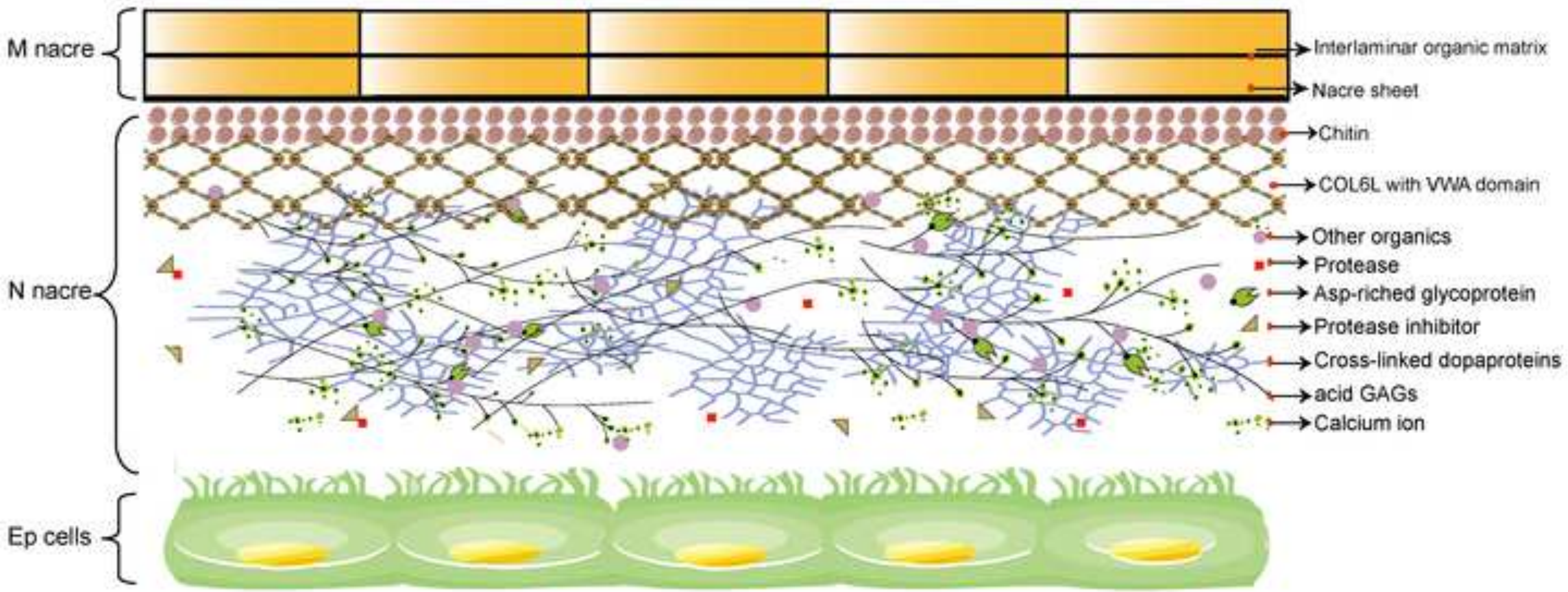

Click here to access/download
Supplementary Material
Additional file 1-06.docx

Additional file 2.figure S1

Click here to access/download
Supplementary Material
Additional file 2.figure S1.tif

Additional file 3.figure S2

Click here to access/download
Supplementary Material
Additional file 3.figure S2.tif

Additional file 4.figure S3

Click here to access/download
Supplementary Material
Additional file 4.figure S3.tif

Additional file 5.figure S4

Click here to access/download
Supplementary Material
Additional file 5.figure S4.tif

Additional file 6.figure S5

Click here to access/download
Supplementary Material
Additional file 6.figure S5.tif

Additional file 7.figure S6

Click here to access/download
Supplementary Material
Additional file7.figure S6-070106.tif

Additional file 8

Click here to access/download
Supplementary Material
Additional file 8-01-06.xlsx

Additional file 9.figure S7

Click here to access/download
Supplementary Material
Additional file9. figure S7-01.tif

Additional file 10.figure S8

Click here to access/download
Supplementary Material
Additional file 10.figure S8.tif

Dear Dr. Zauner,

Thank you for the reviews. We have carefully revised our manuscript according to comments and suggestions from the reviewers. A point-to-point response is provided below. We greatly appreciate the reviewers' comments and suggestions which are very constructive and helpful.

We hope the revised manuscript is now acceptable for publication in GigaScience. Please feel free to contact me if you have any questions. We look forward to hearing from you soon.

Sincerely yours,

Xiaodong Du

Point-to-point responses

Please consider to add BUSCO results in addition or instead of CEGMA. BUSCO results are reported for most new genomics submissions to Gigascience and is becoming a standard feature of our genomics papers.

Authors: Thank you for the suggestion. We have included BUSCO results for evaluating the integrity of our assembly and gene annotation. BUSCO analysis shows that our assembly is 82.80% complete and 7.35% fragmented, indicating our assembly is complete enough for further analysis. All the analysis results were added into the revised manuscript.

Responses to Reviewer reports:

Reviewer #1: The manuscript by Du and colleagues reports the assembly and annotation of a high-quality genome of the pearl oyster Pinctada fucata martensii, providing new insights into the genetic basis of mineralization in bivalve molluscs. This work also takes advantage of RADseq and RNA-seq data, which altogether contribute to a significant improvement of the genome

assembly and annotation, as well as of a series of additional experiments to investigate specific aspects of the study.

Although a draft genome of the same organism had already been published by Takeuchi and colleagues back in 2012, there is no doubt that the work presented in this study is a significant improvement, as it basically reaches a chromosome-scale quality, allowing large-scale comparative genomics analyses. The annotation process, a relevant issue for non-model lophotrochozoans, also appears to have been greatly implemented in this case. The authors focused the main body of their analysis on the genetic basis of biomineralization, offering a comprehensive model and evidencing the expansion of several gene families which might have a role in this process.

Overall, this ambitious work is of high value and, in my opinion, the P. fucata martensii genome could become a reference species for comparative genomics due to the superior quality of this assembly compared to that of the Pacific oyster C. gigas. However, as any ambitious paper, this work requires some adjustments before it can be accepted for publication. Please find attached below a list of my concerns and comments:

Authors: We appreciate the positive assessment. Below we are point-by-point responses to the comments showing how we revised the manuscript.

Major concerns

I strongly advise the authors to use TPM (Transcripts Per Million) instead of RPKM for the quantification of gene expression levels, as it enables a more reliable comparability across samples. See Wagner et al. 2012 (doi:10.1007/s12064-012-0162-3). This will likely have only minor effects on the results, assuming that the number of reads per sample and the structure of the transcripomes were similar to each other. A few figures and tables might need to be slightly fixed, e.g. the network analysis and heat maps. Overall this should not bring too many modifications to the interpretation of the results, but in my opinion this is needed to ensure the use of the "gold-standard" methodology available at this time.

Authors: Thank you for the suggestion. We used TPM (Transcripts Per Million) to calculate gene expression of 14 samples, and found excellent correspondence between TPM and RPKM (Correlation coefficient are more than 0.93, Fig. 1), and we especially checked genes appeared in the manuscript and found they are almost

identical. Thus, we did not modify the network and heatmap figures in the revised. But we uploaded results of TPM to the GigaDB and the details were added to Methods section.

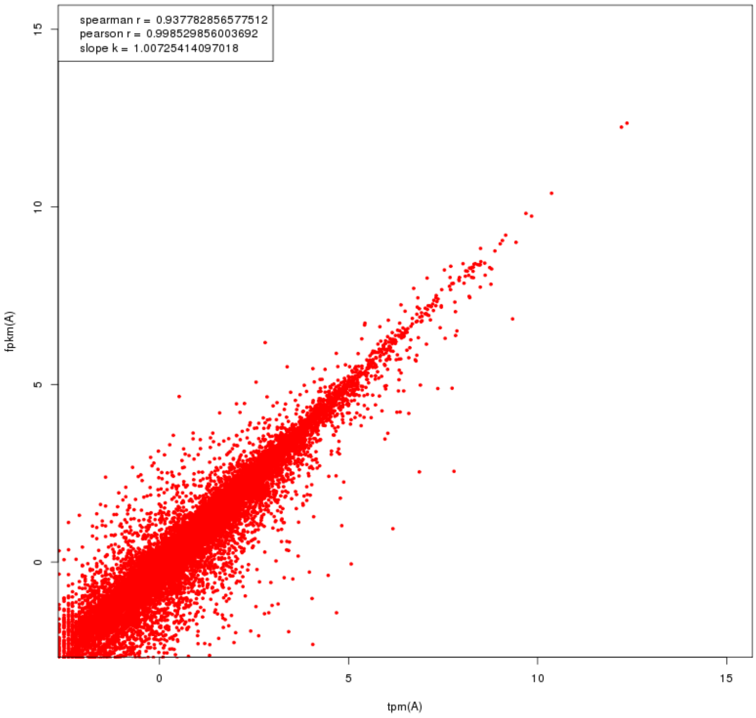

Fig.1. Correlation between gene expression calculated by TMP and RPKM.

Table S10 and some relevant part of the discussion of results (page 6 in particular) would benefit from the inclusion of a mollusc without a carbonate skeleton,such as Octopus bimaculoides and/or Aplysia californica. The lack or non-expansion of chitinase for example, would de facto confirm the intuition of the authors, or otherwise indicate that this expansion event is not related to the acquisition of a shell. Tyrosinases might be useful for nacre matric cross-linking but, at the same time, they could have other functions in bivalves (some authors have previously suggested a possible role in melanisation/immunity). Use the data from Albertin et al. 2015 (doi:10.1038/nature14668) for what concerns O. bimaculoides, data from Aplysia can be found in NCBI genomes.

Authors: This is a good point. We included *Octopus bimaculoides* for comparison and added the results to Table S10 in the revised. We revised the results and discussion to include comparison with molluscs without shells.

Page 6, line 47: "expanded". This needs to be better contextualized. Expanded compared to what? The use of such terms should always be referred to a general "baseline" situation which, in this case, could be represented by the inferred most recent common ancestor to all lophotrochozoa or, even better, to the most recent common ancestor of all molluscs (please add Octopus bimaculoides and Aplysia californica in your analysis). EDIT: This part became much more clear after the reading of additional files. However, I still think that the addition of the two aforementioned mollusc species will be beneficial.

Authors: We used the same pipeline and database as before for KEGG annotation of *Octopus bimaculoides* and *Aplysia californica* as suggested. Please see the results in the revised Table S10. We also found the expansion of collagen VI and chitinase in *Octopus bimaculoides* just like in *Pinctada fucata martensii* and *Crassostrea gigas*. According to the phylogenetic tree of these species (Fig. 2), our results indicate that the expansion occurred in the recent common ancestor of all molluscs. The expansion of chitin synthase in *Lotta gigantea* as well as *P. fucata martensii* and *C. gigas*, suggests that this expansion occurred in the common ancestor of gastropods and bivalve. The expansion of sulfotransferase in *P. fucata martensii* and *C. gigas* suggested that this specific expansion occurred in the common ancestor of *P. fucata martensii* and *C. gigas* or bivalves. We revised the manuscript accordingly.

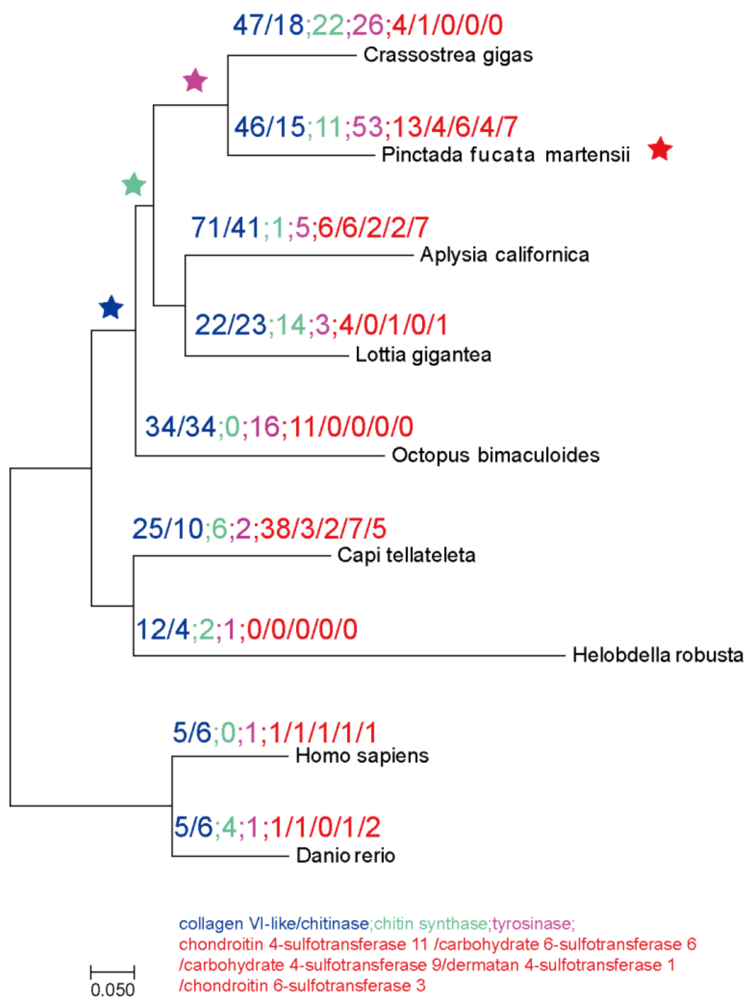

Fig. 2. The phylogenetic tree of 9 species analyzed and the numbers of genes related to shell matrix formation.

Table S14 and "Regulation network of the nacre matrix proteins" section. The authors relied on KEGG annotations for elucidating known pathways enriched in the network of nacre matrix co-expressed genes. KEGG annotation is quite trustworthy, as it relies on a well-known set of evolutionarily conserved genes, but the annotation rate observed in invertebrate genomes with KEGG is systematically lower than that observed with Gene Ontology (in other words, KEGG is somewhat biased on vertebrates). Why did the authors choose not to use GO terms in addition to KEGG annotations for this enrichment analysis? My guess is that the use of GOs might add some useful information to the findings reported in this section.

Authors: The GO enrichment of the co-expressed genes showed that transporter activity (GO:0005215, P=1.86e-10) and ATPase activity (GO: 0042626, P=6.79e-07) are significantly enriched, which is consistent with the enrichment of ABP transporters in KEGG enrichment results. We have added these results in Additional File 1 Table S15.

Minor issues

Page 3, line 33: I would suggest to add a reference from Murgarella et al. 2016 (http://dx.doi.org/10.1371/journal.pone.0151561), as this is a useful example in support of the complexity of bivalve genome assembly, high polymorphism and repetitive content.

Authors: The reference has been added to revised. Thanks.

Page 3, line 59: please define "relatively distant".

Authors: We replaced "two relatively distant parents" with "two genetically distant parents"

Page 4: the first sentence (Raw […]c technology) is unnecessary and can be removed here. Move it to the Additional File 1.

Authors: We removed the sentence (Raw […] technology) as suggested.

Page 5, lines 42-49: it is worth mentioning that these estimates are in agreement with the most up-to-date phylogenomic studies on the evolution of bivalve molluscs (e.g. Bieler et al. 2014,

http://dx.doi.org/10.1071/IS13010).

Authors: We agree and have added the sentence. Thanks.

Figure 1e: What is the bootstrap support value for the nodes shown? Did the authors use a bootstrap threshold to show or to collapse nodes? This should be specified in the figure caption.

Authors: We used FastTree to construct the gene tree and FastTree computes local support values with the Shimodaira-Hasegawa test, which resembles the parameter of "SH-like local supports" in PhyML 3. FastTree includes the following stages: 1) Heuristic neighbor-joining; 2) reducing the length of the tree including nearest-neighbor interchanges, subtree-prune-regraft moves and distance model; 3) maximizing the tree's likelihood with NNIs; 4) local support values. We set SH-like local supports =1000 in our analysis. Our detailed parameters are “amino acid distances: BLOSUM45, joins: balanced, support: SH-like 1000, search: normal +NNI +SPR (2 rounds range 10) +ML-NNI, opt-each=1, tophits: 1.00*sqrtN, close=default refresh=0.80, ML model: Jones-Taylor-Thorton, CAT approximation with 20 rate categories”. The local support values are given as names for the internal nodes, and range from 0 to 1, not from 0 to 100. The resulting support values are strongly correlated with the traditional bootstrap (r=0.975). Please see the gene tree figure below with the support values added.

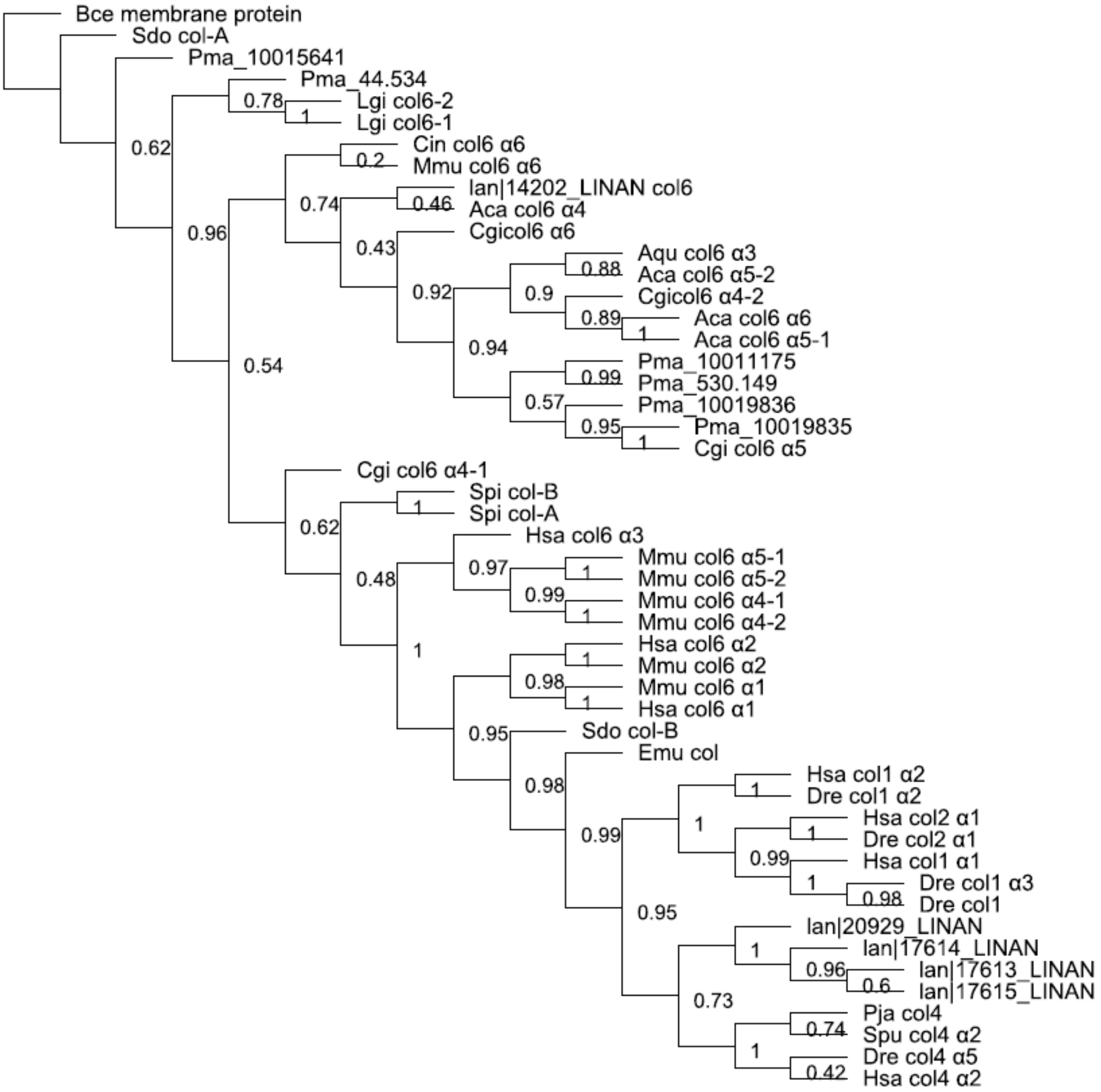

Fig. 3. The gene tree with the local support values for each node.

Figure 2c: same as above. Please replace "value" with RPKM (or TPM, if this is changed).

Authors: We replaced "value" with "RPKM value" in the figure 2b. Because of the excellent correspondence between RPKM and TPM, we did not change RPKM to TPM. Because RPKM values of those genes exhibited significant variation and were difficult to show in one figure, we standardized the RPKM values with the following process. We summed up the RPKM of A gene at twelve developmental stages: ΣA. The value of A gene at i developmental stage: PAi=Ai/ΣA. We sum up the RPKM of A gene in different tissues: ΣAT. The value of A gene in j tissue: PAj=Aj/ΣAT.

Additional File 1, page 10: the sequencing strategy should be specified: was it a single-end or a

paired-end strategy? What was the length of the reads? Which software was used to perform the trimming of the reads?

Authors: Paired-end libraries with insert-size of 500 bp were generated from each sample and sequenced separately using Illumina Hiseq 2000. Raw reads, with read length 100bp, were filtered using SOAPfilter (V2.2) to remove those containing adapter sequences, high numbers of "N" and having low sequence quality scores, before further analysis. As suggested, we added these details to Additional File 1.

Additional File 1, page 4: "Genome characterization by K-mer analysis". This should be better explained. Did the authors use Jellyfish or a similar software to perform this analysis?

Authors: We used Kmerfreq (our inner program) for the k-mer analysis but re-analyzed using Jellyfish. We found that the results from the two software are very similar as shown in Fig 4 below. The result and figure have been uploaded into GigaDB.

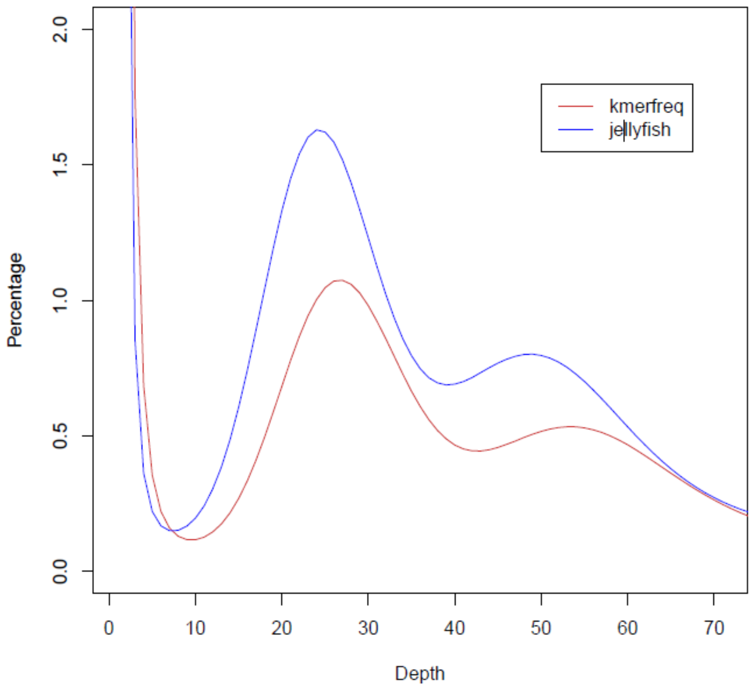

Fig. 4. K-mer distribution analyzed using Jellyfish and inner kmerfreq.

Repeat content analysis: the repeat library should be made available as a supplementary file.

Authors: Thank you for the suggestion. Because the file of the repeat library is about

26 Mb, which is over the limit for supplementary file, we have uploaded the file into GigaDB database.

Additional File 1, page 8: how was the set of 114 single-copy genes determined? Also, the authors should mention that the sequences were concatenated prior to analysis.

Authors: We identified single-copy genes that have only one gene per family in each species based on gene family results from Treefam. The results of gene families clustering and single-copy genes identified have been uploaded into GigaDB, and the method was described in additional file 1.

Table S11: Cte and Hro species abbreviations are missing. Please check if other are missing as well.

Authors: Added. Thanks.

Additional file 7 (Figure 6): RPKM (or, hopefully, TPM) should be added near "values" under the colour bars.

Authors: Added. Thanks.

The authors are encouraged to add an additional supplementary table summarizing codon usage in P. fucata martensii. While this statistics is often neglected, it is important to investigate selection of certain gene families for translational efficiency and/or accuracy. I would also like to see some additional information concerning GC content of introns, exons, inter-genic regions and GC content at each codon position.

Authors: As suggested, we summarized codon usage in *P. fucata martensii* and added the statistics to the supplementary file. We found the number of codon usage in *P. fucata martensii* is similar with other 8 species (Fig. 5). Also, we calculated the GC content of introns (34.1%), exons (42.4%), inter-genic regions (35.2%) and GC content at each codon position (see Fig. 6 below). The results show the GC content of exon is notably higher than that of intron and inter-genic regions. The GC content of position 1 of codon (48.4%) is notably higher than that of position 3 (40.6%) and

position 2 of codon (38.3%) (Fig. 7). These figures added in the additional file 4, figure S3

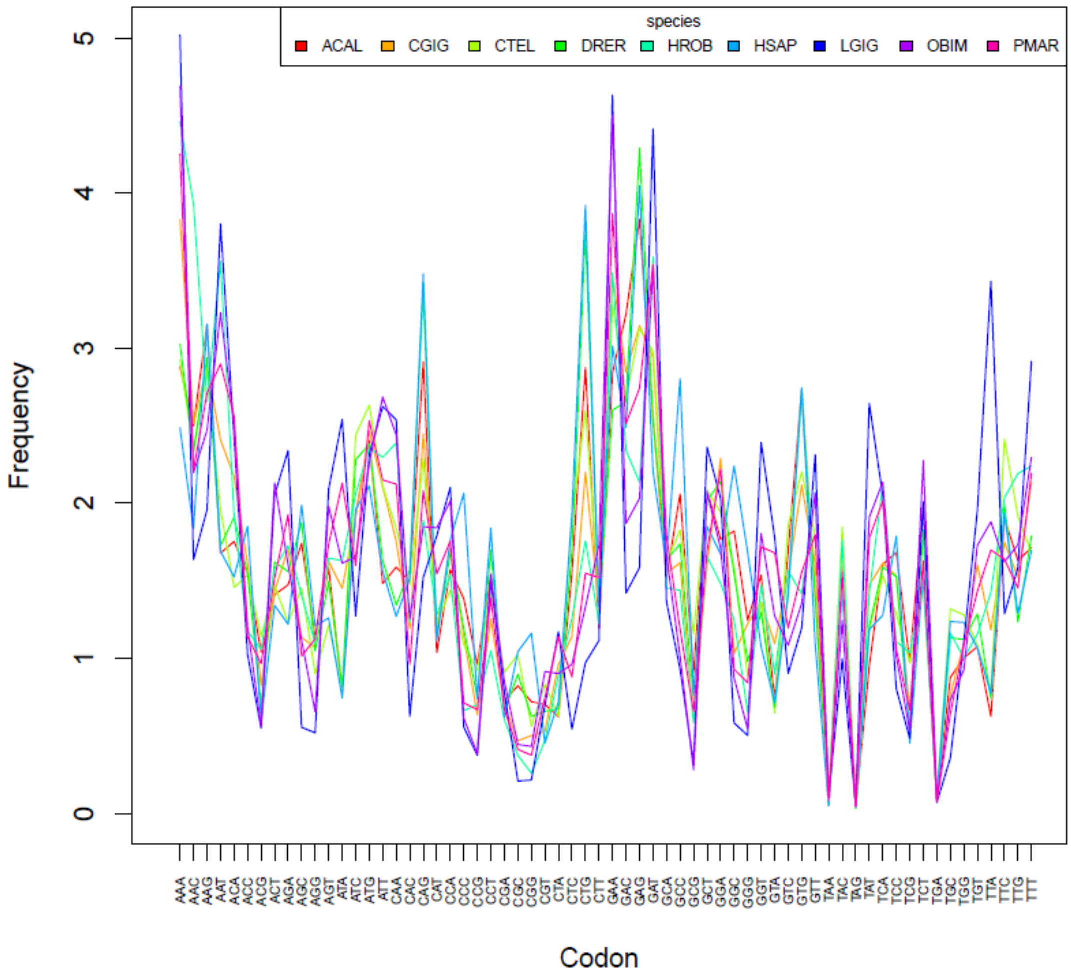

Fig. 5. Comparison of the distribution of codon usage among 9 species.

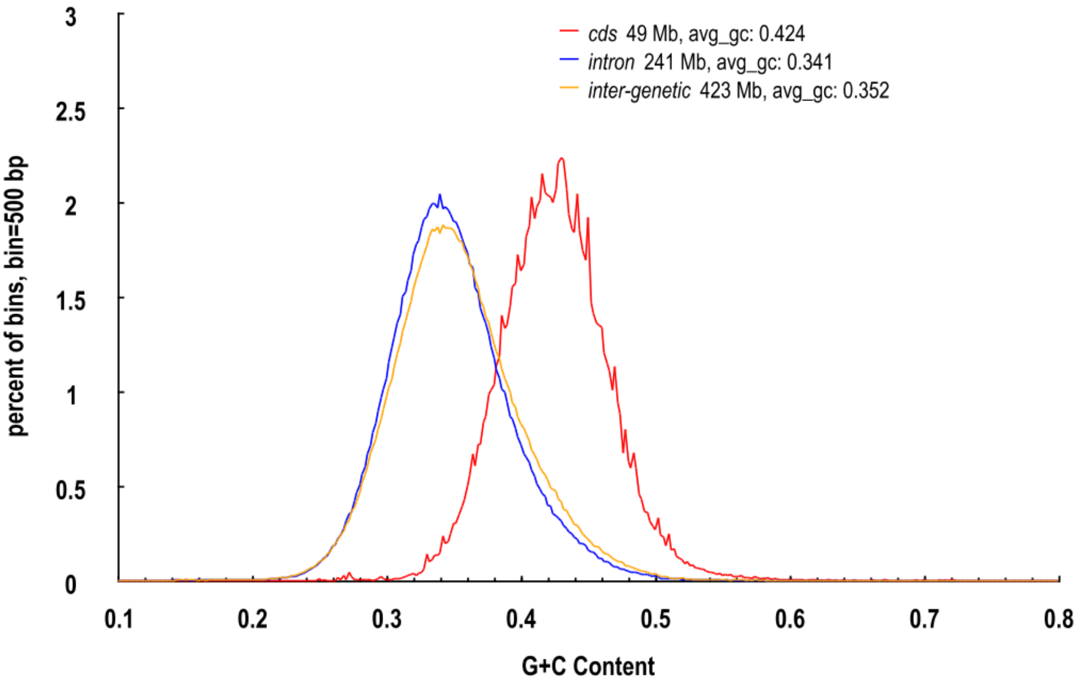

Fig. 6. The GC content distribution of exon, intron and inter-genetic regions.

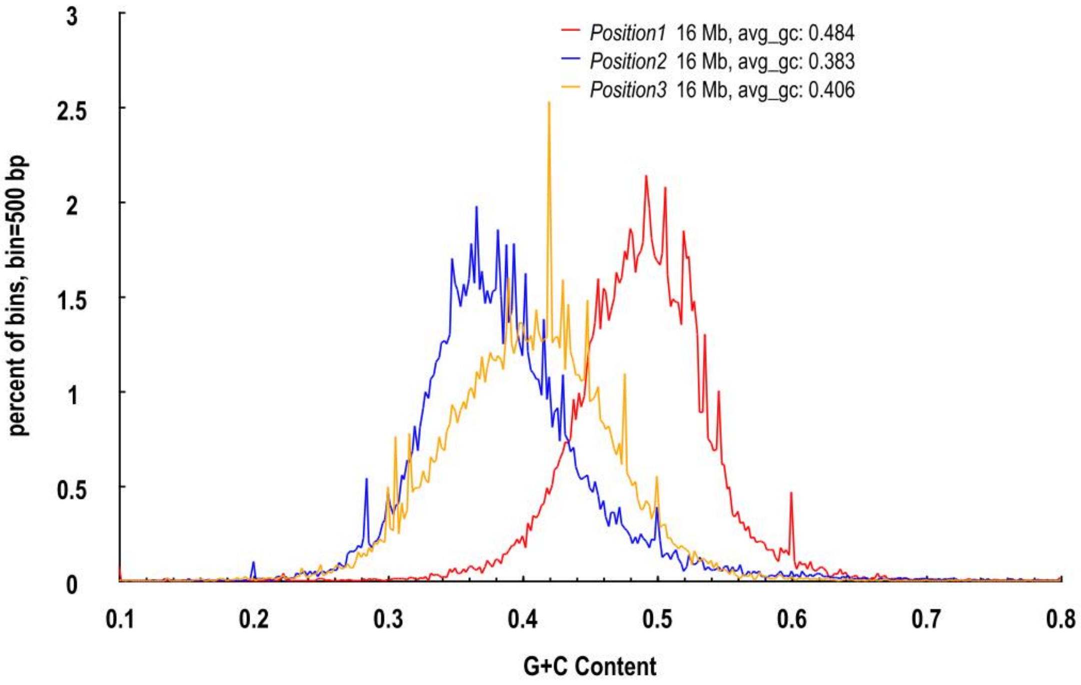

Fig. 7. The GC content distribution for each codon position.

Another aspect which has not been investigated concerns the presence of non-coding genes. Currently, the number of non-coding genes annotated in the human genome surpasses that of protein-coding ones. For obvious reasons, our knowledge of such genes in non-model organisms

is not equally advanced. How many out of the gene predictions are likely to be non-coding? The authors used an annotation protocol which included the alignment with RNA-seq data, which should be optimal for this purpose. There are a number of ways to predict this, such as CPC (doi: 10.1093/nar/gkm391).

Authors: Thank you for the suggestion. We used CPC to do the prediction and we added these results in the revised. For non-coding RNA prediction, we randomly selected 15 samples from our transcriptome datasets and used TopHat2 [1] to map the raw data to the reference assembly. We used Cufflinks and Cuffmerge to predict transcripts based on TopHat2 alignments, then novel transcripts were generated by performing Cuffcompare against the annotated CDS [2]. Totally 64,409 novel transcripts were generated. We then used coding potential calculator (CPC) [3] based on the UniRef90 database to access the coding potential of these transcripts. We used the same threshold as the work of Jiang et al. [4] and kept the transcripts with coding potential score $\leq$ -1. Finally, 35,245 transcripts are predicted to be non-coding. All the results have been uploaded into GigaDB.

While the two last points concern topics which go beyond the scope of the paper (mineralization), I believe their assessment will add further value to this interesting paper.

Authors: Thank you for the positive assessment.

Reviewer #2: In their manuscript entitled "The genome of Pinctada fucata martensii and multi-omic analyses provide insights into Matrices of biomineralization", Du and co-workers described the release of a novel and clearly improved version of the pearl oyster genome, that had been previously described by Takeushi et al. (2012), and provide input to biomineralization process characterization, thanks to combined transcriptomic, proteomic and functional characterizations via RNAi experiments. This MS represents an impressive amount of work and also an ambitious challenge for the sequencing and the assembly of this large genome presenting high level of repeated sequences. Although, I like the study that represents a very interesting set of data, I have also some concerns, including significant technical and data interpretation issues, and the MS need major revisions before being considered for publication in GigaScience or in another

journal.

Authors: Thank you for the positive assessment and helpful suggestions. Below we provide point-by-point responses to your comments and indicate how we have revised the manuscript.

I have listed here my main concerns that aim at been provided to the authors:

- My main concern go to the over interpretation of some blast results for some identified proteins that provide erroneous indication leading to wrong conclusions. For example, according to supplementary information, Pinctada_martensi_GLEAN_10028879 gave "Tyrosine-protein phosphatase" as first hit of the blast search, however it is not and should be considered at all as a tyrosine-protein phosphatase. Indeed, this protein sequence exhibits 5 fibronectin 3 domains that contribute to give a significant alignment (first rang blast hit) with mammalian tyrosine-protein phosphatase because it contains also 5 FN3 domains, BUT this true tyrosine-protein phosphatase contains also specific tyrosine-protein phosphatase domains, that are critically missing in the Pinctada protein, then it should simply not be considered as a fibronectin. Indeed, various proteins contain FN3 domains which are not exclusive to tyrosine-protein phosphatase. See smart domain list for FN3:

http://smart.embl-heidelberg.de/smart/selective.cgi?domains=FN3&terms=&taxon_text=&input=Architecture+query

- This is also the case of collagen IV blast hit. For similar reason, it seems to me that the different protein that gave "Collagen IV" for Blast hit shouldn't be considered as true Collagen IV proteins. Indeed, when looking at the sequence of these proteins they all exhibit between 1 and 4 VWA domains (ex. Pinctada_martensi_GLEAN_10019836), but far less than the true vertebrate collagen IV that exhibit at least 8 VWA domains in addition to other collagen specific domains (M7B9T5) and sometimes few EGF domains too. For example, the blue mussel PIF isoform, called BMSP by Suzuki and co-workers 2011 in Chembiochem, exhibits similarly 4 VWA domains, together with 3 CBD2, a LamG-like domains and an acidic low-complexity domains, but this protein is, from any literature, not considered and/or named as a "Collagen IV". Indeed various proteins belonging to diverse family exhibits VWA domains (see smart server results for VWA at:

http://smart.embl-heidelberg.de/smart/selective.cgi?domains=vwa&terms=&taxon_text=&input=Architecture+query).

From now, although it represents an appealing idea (with indeed important potential consequences on evolution scenario…) that has been discussed in different article, there is still no evidence of the presence of collagen in mollusc shell. Taken together, I insistently recommend to the author to reconsider this point and to modify their MS accordingly.

Authors: We appreciate the comment. We are very sorry that the descriptions line in the uploaded data was the annotation results using Nr database, which may be inappropriate for some gene, such as Tyrosine-protein phosphatase. Now, we have added the other three annotation results using KO, GO, IPR database, which may be helpful for gene characterization. Regarding the annotation of collagen gene in our manuscripts, firstly, we like to clarify that the collagen type mentioned in our manuscript is "Collagen VI" not "Collagen IV". Concerning the six proteins with VWA domains found in the shell matrix proteins, we annotated them as "Collagen VI" because they are highly homologous with "Collagen VI" annotated by KO database. The annotated results by KO were helpful for us to elucidate the signal transduction pathways underlying biomineralization, which is our focus in the last part of the manuscript. In contrast, Nr database contains miscellaneous information uploaded by researchers with personal views, such as PIF and BMSP, which was annotated by the authors based on gene function and characterization. Among the six shell matrix proteins with VWA domain, 44.534 is annotated as "BMSP" by NCBI blast, but it is annotated as "Collagen VI" by KO database. In this situation, we considered 44.534 as "Collagen VI". In our opinion, this annotation is more informative and helpful for us to explain the evolutionary dynamics of 44.534. PIF is annotated as midasin by KO database, and we didn't target it for further analysis.

We recognize and agree that "various proteins belonging to diverse family exhibits VWA domains", but VWA domains from different families vary in sequences and can be classified by phylogenetic analysis. In the shell matrix proteome, 12 proteins with VWA domains were detected, and only 6 proteins were annotated as "Collagen VI" by KO database. That's to say that we didn't annotate all of the proteins with VWA

domains as "Collagen VI". Further, phylogenetic analysis showed the six proteins clustered with "Collagen VI" from other animals, which supports our annotation.

Although structural analysis showed the six matrix proteins don't have the short triple-helix region (THR), we found that all "Collagen VIs" from invertebrates don't have THR (Fig. 2d), and some COL6s without THRs still exist in the human and zebrafish genomes, and more importantly, phylogenetic analysis indicates that COL6 without THR region emerged early during evolution, and fibrillar COL1/2/4 are derived from collagens without THRs (Fig. 2e).

Even so, we agreed with the reviewer's suggestion of caution, and we changed the name of these proteins to "Collagen VI like proteins" and have modified our manuscript accordingly. We feel it is important to highlight the homology between these proteins and collagen VI, which has significant implications to the evolution of collagens and shell Matrices.

- I also notice from the amino acid sequences of the matrix proteins the absence of peptide signal (predicted by SignalP4.0) for many of these proteins suggesting that these sequences could be incomplete/not correct and/or that somehow the gene model do not accurately predict all proteins exon/intron (especially in their N-termini). I assume that gene model prediction from a non-model organism, such as Pinctada, constitutes a big challenge, however I think that it more interesting to discuss this point within the MS (rather to simply omit it), as accurate gene prediction is still a change for the scientific community and current strategies are still not perfect, constituting a limitation to search automatic and non manually curated approach.

Authors: Thank you. It is a big scientific challenge for the assembly of genome with high heterozygous ratio and the gene prediction of a non-model organism. The software based on a de Bruijn Graph, such as SOAPdenovo[5], is difficult to obtain a satisfied result due to the increased complexity of the de Bruijn graph structure. Overlap-Layout-Consensus assembler, such as Celera Assembler[6], based on the data of fosmids or BACs hierarchical sequencing and third-generation long reads (such as PacBio long reads) are employed with overcoming such problems. However, the best choice for solving the assembly of complex genome is to obtain the haploiod or

homozygous samples to sequence. For the ab initio gene prediction software, such AUGUSTUS[7], the aim of them is to find potential coding sequences with sufficiently long open reading frames, but the translated regions may be very short and the absence of stop codons becomes meaningless. The similarity-based approaches including homologous protein sequences, EST sequences and transcripts assembled from RNA-seq reads can produce biologically relevant predictions, but they may not cover entire coding exons. Due to their strengths and weaknesses, generally, a synthesis software, such as GLEAN[8] and MAKER[9], is used to synthesize these evidences obtained from ab initio gene predictions and similarity-based approaches into the final gene annotation. Further, in our study, we performed the BUSCO analysis[10] to evaluate the integrity of gene annotation, indicating our assembly is complete enough for further analysis. We added this part in the manuscript.

Suggestion: The presence of signal peptide and the accuracy of the gene model could also be investigated and discussed in the MS, as from now most if not all testified mollusc shell proteins exhibit such signal peptide, and that exception remains rare and based on alternative secretion scenario via transmembranal release mechanism (Silva-Ramos et al. 2013 in Mol Biol Evol).

Authors: Thank you for the suggestion. We added the peptide signal prediction of the shell matrix proteins by SignalP4.0. As showed in supplement, there are 269 proteins without typical peptide signal. We agree with the reviewer's suggestion that there may be some alternative secretion scenario via transmembranal release mechanism. We have added the corresponding discuss in the manuscript.

- I really regret that there is no (semi-)quantification of the different proteins described here, as some of the matrix identified proteins from the list are very likely contaminants (not true matrix proteins, rather cellular debris), such as actins, actin-binding proteins, tubulins, myosins, HSP70, ATP-synthase and perhaps many others…. Indeed, it has been now well known that biominerals may contain various contaminants (such as cellular debris proteins…) in addition to the calcifying matrix that is embedded within the mineral phase during the biomineralization processes. It has been shown (Silva-Ramos et al. 2013 in PNAS; Marie et al. in Proteomics; Mann et al., 2012 and 2013 in Proteome Science) that an extent and adequate cleaning of the shell fine powder with

HOCl can somehow reduce this contamination. However as specified by Mann et al 2013 in Proteome Science a rational way to deal with this potential contamination (that can theoretically never been completely

removed…) is to quantified proteins and to focus only the attention on main and truly matrix proteins.

All proteins that are patent cellular component (such as actins, tubulins, histones, ATPase, …) should be reconsidered and removed from matrix protein list. Mass spectrometry is a very sensitive technics and one should be careful about such contamination of extracellular matrices by cellular contents. The lack of criticism of the proposed protein list represents a significant issue, and the protein list proposed here should be deeply reconsidered (critically refined/refiltered…).

Authors: Concerning the cellular debris proteins in the shell proteome, we agree with the reviewer's opinion that they may be contaminants not real matrix protein, as the amount of these proteins was less abundant than the known shell matrix proteins. Thus, to find the crucial molecular functional in biomineralization, we removed the cellular debris (Additional file 8, S1), and even without removing them, it did not affect COL6-like proteins being one of the most abundant (Additional file 8, S1).

- Tyrosinases were detected in nacre, but from previous literature, they have been mostly described from the prismatic layer (by proteomic). It would be interesting to specify in Pincata fucata martensii the tyrosinase identifications in nacre and to compare them to those in prisms. Previous work have shown that some tyrosinases are more over-expressed in ME than in MP, it seems to be a different picture here, and this point aim being discussed and should gave interesting consideration about the role of these proteins and their activity in nacre, but also prisms and periostracum formation…

Authors: Tyrosinases (Tyr) was reported to be functioning in mediating the crosslink between shell matrix protein by converting tyrosine residues into adhesive DOPA [11, 12]. A total of twelve Tyr were identified from shell proteome. Six of them were detected in both nacreous layer and prismatic layer, indicating their similar function. Two Tyrs were specifically detected in the nacreous layer, and they were over-expressed in MP than ME. Four Tyrs were specifically detected in prismatic layer, and most of them were over-expressed in ME than MP. These results suggest

that Tyr functions both in nacreous layer and prismatic layer formation in *Pincatada fucata martensii*.

The high expression of Tyrs in MP and ME as well as their abundance in shell matrix suggest that Tyrs are an important structural component of shell. Tyr belong to the “type-3 copper” family and have a conserved active site of six histidine residues mediating the binding of copper ion as cofactor [13]. Metal ions such as Cu2+, Zn2+ and Mg2+ are important factor to stable the crystalline form of calcium carbonate [14-16]. The deposition of Tyr and metal ions in the shell may contribute to controlling the concentration of metal ions of extrapallial fluid, and finally help to stabilize the crystalline form. Interestingly, we found that the histidione residues in nacre-specific Tyr (Pm10005159 and 10016044) were seriously replaced with other residues, suggesting the loss of metal ion binding ability. Therefore, we suggest that the function of nacre-specific Tyrs may be different from that of prism-specific Tyrs and need to be further elucidated. We recognize that some Tyrs may have other functions unrelated to shell or shell matrix formation.

- By the way, it seems that the author avoid to cite previous published works on Pinctada shell proteomics by Marie et al. (2012) in PNAS and more recently Liu et al. (2015) in Scientific Reports, then to integrate and compare these information. This curious omission makes the reader (and the rewiever too) getting suspicious about the objectivity of the MS (that is not a good think for a reader…), and I recommend to add and to discuss adequately these references (at least in the discussion).

Authors: Thank you for pointing us to these references which were added and discussed.

- There is no description of the bioinformatics identification of the matrix proteins using a search engine (such as Mascot, ProteinPilot or Xtandem!....), the parameter used, the filters selected…... This information is critically missing. For example, the reader (and I) cannot guess what mean "LSBD", "LSRD", "ZSBD" or "ZSRD", then appreciate this data…

Authors: We used the Mascot software (v 2.3.02) to query the MS/MS spectra data of

matrix proteins in the transcriptome database (36396 sequences). We applied the trypsin cleavage rule with one missed cleavage site. Carbamidomethylation of cysteines was considered as the fixed modifications while Gln->pyro-Glu (N-term Q), Oxidation (M) and Deamidated (NQ) were considered as the variable modifications. Peptide mass tolerance was set to 0.05Da and fragment mass tolerance was set to 0.01Da. We used target-decoy search strategy [17] to identify the matrix proteins, and the False Discovery Rate (PDR) was ≤1%. We added the details into Methods and spelled out “PAIP”, “PASP”, “NAIP”, “NASP”, which are abbreviations for “prism acid insoluble protein”, “prism acid soluble protein”, “Nacre acid insoluble protein”, “Nacre acid soluble protein”, respectively.

- It is not specify how the gene model lists (generated according to the three different strategies: de novo, comparison of reference metazoan genomes and comparison with reference Pinctada transcriptomes) were merged into GLEAN db in order to avoid any gene/protein redundancy. This point aim at been specified.

Authors: Thanks for your suggestion. We used the trainGlimmerHMM tool included in the GlimmerHMM software package to estimate a Markov model with 1000 high-quality genes (The genes were previously used to train Augustus). Then we used the Markov model to identify the coding potential of each transcript assembled from the transcriptome data. So, transcripts with complete ORFs were extracted and multiple isoforms from the same locus were collapsed by retaining the longest ORF. Next, we integrated these ORFs with homology-based gene models to form the core gene set. Then we added the homology-based gene models which not supported by transcriptome-based evidence but supported by homologous evidence from at least two species to the core gene set. Finally, we added the de novo-based gene models not supported by homology-based and transcriptome-based evidence to the core gene set. As a result, a total of 32,937 non-redundant protein-coding genes were annotated in our genome assembly.

We also added the BUSCO results in the supplementary file. These method details

were added to the revised. We also added BUSCO results in the supplementary file which show that our assembly is 82.80% complete and 7.35% fragmented, complete enough for further analysis.

- As Alcian blue staining specificity is depending on the pH of the buffered solution (Wall & Gui, 1988 in Anal Chem), this information should be provided in the M&M. Alcian blue reveals acidic compounds comprising GAGs, but very acidic protein can also be stained by this method (Marin et al. 2005 in J Biol Chem). Are you sure it is GAGs or not acidic protein that are stained here? Indeed, previous works as shown that Pinctada nacre exhibit a limited amount of acidic polysaccharides (Bédouet et al 2001 in Comp Biochem Physiol B). On the figure 3, I am wondering if it cannot be mucocytes that are stained by blue alcian?

Authors: Periodic acid Schiff (PAS) reaction is a method for detecting neutral and acid polysaccharides. When using a double staining method of pH 2.5 alcian blue and PAS (AB-PAS), neutral glycosaminoglycans (GAG) are stained "red" with Schiff's reagent, and acid GAG are stained "blue" with alcian blue [18].

- I do not find interests in Figure 4, as this model remains unpleasant and poorly informative, and I recommend to remove it (but other supplementary figures are much more interesting and aim at being integrated in the MS as regular, as for example figures S3 or Figure S6 are very fine).

Authors: Thanks. We move the figure S3 into the main manuscript. And we also maintained the Figure 4.

- I do not agree with the assumption that "no silk protein can be found in the transcriptome and the proteome of Pinctada" (P6, L34-38). The original idea that silk-like protein contribute in matrix-regulated processes of nacre formation was suggested with the identification in 1997 by Sudo et al in Nature of MSI60, which exhibits several poly-Ala domains, such as silk proteins do. Other poly-Ala containing proteins have also been retrieved from the Pinctada shell nacre (NUSP-2 or NUSP-9) by Marie and co-worker (2012) in PNAS.

Authors: It is arguable whether MSI60 and NUSPs are homologs of silk proteins. In our opinion, they are not silk proteins as they show little sequence homology with silk proteins other than poly-Ala and lack the ensemble internal repeats that are

characteristic of silk proteins (see also Furuhashi et al., 2009)[19]. We respect the difference in opinion here.

Minor comments:

- The text should be improved, and some sentences remains awkward. I suggest to the authors to get assistance from an English editing service. Some example of sentence to reword: P2, L48-50; P3, L2-3; P3, L42-44; P3, L5; P9, L19-26;…

Authors: Thanks. We have modified the sentences and improved the manuscript in general.

- There is no legend for the "GC content" figure of figure S1.

Authors: Added, thanks.

- Figure S7: expression profile of Pma_44.534 and Pma_10015641 (a) are without shell phenotyping then look quite superflux in comparison with other genes presenting phenotypes (b).

Authors: Thanks, we have modified it.

- Concerning the WGCNA, in the text of the M&M it is indicated that it has been performed with 158 biomineralization genes, but in the result section it is specified that it has been performed with the 234 nacre matrix proteins. Please clarify this point.

Authors: We are so sorry for this mistake. We first used 158 biomineralization genes with at least 5 unique peptides to draw a preliminary network. But, in the final analyses, we used all 234 proteins isolated from nacre to draw and show a more comprehensive co-expression network for nacre formation.

- The most important pathway highlighted by the KEGG analysis is by far "metabolism". Maybe the authors should also specify (and discuss) this observation in their MS?

Authors: We discussed this point in the revised manuscript.

- Please remove "constitute a gel-like substance" (subtitle P7, L37), because no evidence that

GAGs indeed constitute a gel-like substance have been provided here.

Authors: Reference is provided to support the statement.

- P9, L16: The shell also contains the myostracum and the periostracum layers.

Authors: We agree and modified accordingly.

- The discussion is too long, and some aspect sounds too speculative. This section should be shorten.

Authors: We have shorten the discussion somewhat.

- "Matrices": maybe more conventional is "Matrices".

Authors: Corrected, thanks.

- I cannot find a mention on how to access/download the genome or gene model data.

Authors: Added.

Reviewer #3: I was asked to review only the methods used for the de novo genome assembly of this oyster species.

De novo genome assembly can be challenging especially in mullusks where many of the species are known to be highly repetitive and or contain high levels of heterozygosity that can confound an assembly program. As they stated in the manuscript a straightforward assembly resulted in an inadequate assembly. Upon discovering this, the researchers adopted a BAC clone assembly method that is known to overcome some of these issues. The methods of assembling individual BACs and taking the best assembly from each BAC is reasonable. These assembled BACs were scaffold using a program I am not familiar with Rabbit, however, since it requires long overlaps for the assembly I suspect this program is more than adequate to perform the higher level assembly of joining BACs. The quality of the assembly appears to be high with scaffolds containing almost all of the CEGMA genes. The use of the genetic map to pull 86% of the

assembly into chromosomes suggests this is a good assembly.

Authors: Thanks for your appreciation.

I believe this assembly can be published as is. However, I would like to make sure that all raw data is available (raw reads used to assemble BACs, assembled BACs, assembled Scaffolds, genetic map marker information (not just the centamorgan position but also the sequence for each marker).

Authors: We have uploaded assembled BACs, assembled Scaffolds and genetic map marker information to GigaDB. We did not upload raw reads that were used to assemble BACs to GigaDB, because the amount of data is too large.

Any further speculation of the quality of the assembled scaffolds could easily be put to rest if they used the genetic map to show there were no inconsistencies with the assembled scaffolds. I suspect they did this and broke scaffolds that were inconsistent. SSPACE is known to be a greedy scaffolder so I wouldn't be surprised if there are a couple of scaffolds that needed to be broken apart based on the genetic map. If this was done by the authors a short addition to the supplementary methods would be good to see.

Authors: Thanks a lot. It is a good suggestion. Firstly, we set a strict cutoff of pair-end reads supports (>=5) in the SSPACE to reduce the errors of link in the scaffolds. For the conflicts between genetic map and scaffolds from SSPACE, we chose to trust the results of SSPACE, if the number of genetic markers located on this scaffold is less than 3, i.e., we maintained the scaffolds and ignored their distributions on the genetic map. If the number of markers of this scaffold is more than 3 and there is no conflict between markers, we will check the links of scaffold, such as the length of gaps and the supports of pair-end reads. Particularly, we pay special attention to the scaffolds with a gap (the length of them is more than 10kb), but the length of each end of the contig sequences are less than 200bp. If the insertsize of supporting pair-end reads is abnormal, we will break the links.

Overall, I feel the authors did a great job with this assembly considering the challenges they faced

and with the anchored scaffolds may represent one of the best assembly we currently have for a mullusk species.

Authors: Thanks again.

--

Please also take a moment to check our website at http://giga.edmgr.com/l.asp?i=5221&l=GKT84E5U for any additional comments that were saved as attachments. Please note that as GigaScience has a policy of open peer review, you will be able to see the names of the reviewers.

Reference

1. Kim D, Pertea G, Trapnell C, Pimentel H, Kelley R, Salzberg SL. TopHat2: accurate alignment of transcriptomes in the presence of insertions, deletions and gene fusions. Genome Biol. 2013;14:R36.
2. Trapnell C, Williams BA, Pertea G, Mortazavi A, Kwan G, van Baren MJ, et al. Transcript assembly and quantification by RNA-Seq reveals unannotated transcripts and isoform switching during cell differentiation. Nat Biotechnol. 2010;28:511-515.
3. Kong L, Zhang Y, Ye ZQ, Liu XQ, Zhao SQ, Wei L, et al. CPC: assess the protein-coding potential of transcripts using sequence features and support vector machine. Nucleic Acids Res. 2007;35:W345-349.
4. Jiang JJ, Cheng LH, Wu H, He YH, Kong QP. Insights into long noncoding RNAs of naked mole rat (Heterocephalus glaber) and their potential association with cancer resistance. Epigenetics Chromatin. 2016;9:51.
5. Luo R, Liu B, Xie Y, Li Z, Huang W, Yuan J, et al. SOAPdenovo2: an empirically improved memory-efficient short-read de novo assembler. Gigascience. 2012;1:18.
6. Myers EW, Sutton GG, Delcher AL, Dew IM, Fasulo DP, Flanigan MJ, et al. A whole-genome assembly of Drosophila. Science. 2000;287:2196-2204.
7. Stanke M, Keller O, Gunduz I, Hayes A, Waack S, Morgenstern B. AUGUSTUS: ab initio prediction of alternative transcripts. Nucleic Acids Res. 2006;34:W435-439.
8. Elsik CG, Mackey AJ, Reese JT, Milshina NV, Roos DS, Weinstock GM. Creating a honey bee consensus gene set. Genome Biol. 2007;8:R13.
9. Cantarel BL, Korf I, Robb SM, Parra G, Ross E, Moore B, et al. MAKER: an easy-to-use annotation pipeline designed for emerging model organism genomes. Genome Res. 2008;18:188-196.
10. Simao FA, Waterhouse RM, Ioannidis P, Kriventseva EV, Zdobnov EM. BUSCO: assessing genome assembly and annotation completeness with single-copy orthologs. Bioinformatics. 2015;31:3210-3212.

11. Aguilera F, Mcdougall C, Degnan BM. Evolution of the tyrosinase gene family in bivalve molluscs: Independent expansion of the mantle gene repertoire ☆. Acta Biomaterialia. 2014;10:3855-3865.
12. Zhang G, Fang X, Guo X, Li L, Luo R, Xu F, et al. The oyster genome reveals stress adaptation and complexity of shell formation. Nature. 2012;490:49-54.
13. Decker H, Schweikardt T, Tuczek F. The first crystal structure of tyrosinase: all questions answered? ChemInform. 2006;45:4546–4550.
14. Kitano Y, Kanamori N, Yoshioka S. Adsorption of zinc and copper ions on calcite and aragonite and its influence on the transformation of aragonite to calcite. Geochemical Journal. 1976;10:175-179.
15. Berner R. The role of magnesium in the crystal growth of calcite and aragonite from sea water. Geochimica et Cosmochimica Acta. 1975;39:489-504.
16. Nassrallah-Aboukais N, Boughriet A, Laureyns J, Aboukais A, Fischer J, Langelin H, et al. Transformation of vaterite into cubic calcite in the presence of copper (II) species. Chemistry of materials. 1998;10:238-243.
17. Elias JE, Gygi SP. Target-decoy search strategy for increased confidence in large-scale protein identifications by mass spectrometry. Nature methods. 2007;4:207-214.
18. Hasumi M, Nagahama Y. Seasonal Dynamics of Reproductive Organs in Male Salamanders of the Species Hynobius nigrescens. Copeia. 1990;1990:367.
19. Furuhashi T, Schwarzinger C, Miksik I, Smrz M, Beran A. Molluscan shell evolution with review of shell calcification hypothesis. Comparative biochemistry and physiology Part B: Biochemistry and molecular biology. 2009;154:351-371.
