# Supplementary material for: The pearl oyster Pinctada fucata martensii genome and multi-omic analyses provide insights into biomineralization
Source: Gigascience. 2017 Jul 25;6(8):1–12. doi: 10.1093/gigascience/gix059 (PMC5597905; doi:10.1093/gigascience/gix059)
Supplement: GIGA-D-16-00075_Revision-3.pdf [file gix059_GIGA-D-16-00075_Revision-3.pdf]

Manuscript

Click here to download Manuscript manuscript 0410-0523.doc

The pearl oyster *Pinctada fucata martensii* genome and multi-omic analyses provide insights into biomineralization

Xiaodong Du[a,*,#], Guangyi Fan[b,g*], Yu Jiao[a,*], He Zhang[g,*], Ximing Guo[c,*,#], Ronglian Huang[a,*], Zhe Zheng[a,*], Chao Bian[g], Yuewen Deng[a], Qingheng Wang[a], Zhongduo Wang[a], Xinming Liang[g], Haiying Liang[a], Chengcheng Shi[g], Xiaoxia Zhao[a], Fengming Sun[g], Ruijuan Hao[a], Jie Bai[g], Jialiang Liu[a], Wenbin Chen[g], Jinlian Liang[a], Weiqing Liu[g], Zhe Xu[e], Qiong Shi[g], Xun Xu[g], Guofan Zhang[d,f,#], Xin Liu[g,#]

*These authors contributed equally.

#Corresponding authors: X.D. (zjduxd@126.com), X.G. (xguo@hsrl.rutgers.edu), G.Z. (gzhang@qdio.ac.cn) and X.L. (liuxin@genomics.cn).

[a]Fishery College, Guangdong Ocean University, Zhanjiang, 524025, China;

[b]BGI-Qingdao, Qingdao 266555, China

[c]Haskin Shellfish Research Laboratory, Department of Marine and Coastal Sciences, Rutgers University, Port Norris, New Jersey 08349, USA;

[d]Key Laboratory of Experimental Marine Biology, Institute of Oceanology, Chinese Academy of Sciences, Qingdao, China;

[e]Atlantic Cape Community College, Mays Landing, New Jersey 08330, USA;

[f]Laboratory for Marine Biology and Biotechnology, Qingdao National Laboratory for Marine Science and Technology, Qingdao, China

[g]BGI-Shenzhen, Shenzhen, 518083 China;

Abstract

Background: Nacre, the iridescent material found in pearls and shells of molluscs, is formed through an extraordinary process of matrix-assisted biomineralization. Despite recent advances, many parts of the biomineralization process and its evolutionary origin remain a mystery. The pearl oyster *Pinctada fucata martensii* is a well-known master of biomineralization, but the molecular mechanisms underlie its production of remarkable shells and pearls is not fully understood.

Results: We sequenced the highly polymorphic genome of the pearl oyster and conducted multi-omic and biochemical studies to probe nacre formation. We identified a large set of novel proteins participating in matrix-framework formation, many in expanded families, including components similar to that found in vertebrate bones such as collagen-related VWA-containing proteins (VWAP), chondroitin sulfotransferases and regulatory elements.

Conclusions: Considering that there are only collagen-based matrices in vertebrate bones and chitin-based matrices in most invertebrate skeletons, the presence of both chitin and elements of collagen-based matrices in nacre matrices suggests that elements of chitin- and collagen-based matrices are deeply rooted and might be part of an ancient biomineralizing matrix. Our results expand the current shell matrix-framework model and provide new insights into the evolution of diverse biomineralization systems.

Keywords: genome, biomineralization, nacre, VWA-containing protein, *Pinctada fucata martensii*

Background

Biomineralization is an extraordinary process where minerals form not following rules of inorganic chemistry but through active biological facilitation and control. Biomineralization is widely distributed and essential to the lives of diverse organisms, ranging from algae to vertebrates that rely on mineralized materials for morphology, structure, protection, movement and feeding. Three principal classes of skeletal biominerals exist on earth: calcium carbonate, calcium phosphate and silica [1]. Whether these skeletal biominerals evolved independently or derived from a common origin is controversial, although current thinking favours independent evolution [2].

One of the remarkable characteristics of biomineralization is its precise control by organic matrices [3]. Organic matrices are complex and variable but can be classified into two basic and highly conserved types that use either chitin or collagen as the templating framework [3]. Despite great interest in harnessing the power of biomineralization for the production of novel materials, our understanding of biomineralization and associated matrices is limited in many taxa, including the well-known masters of biomineralization - shelled molluscs.

Nacre is the remarkable biomineral found in pearls and shells of molluscs that provides lustre and enhanced toughness. The formation of lustrous pearls and shells in molluscs such as the pearl oyster *Pinctada fucata martensii* has long fascinated humans. The biomineralization process of nacre formation is complex and involves sophisticated organic matrices as well as cells, many aspects of which remain elusive [4-8]. The origin and homology of nacre formation with other biomineralization processes such as crustacean shell and vertebrate bone formation is not understood [9]. Studies of biomineralization and other fundamental questions in biology and evolution can be greatly empowered by whole genome analyses, which have been difficult in molluscs owing to challenges in assembling their highly polymorphic and complex genomes [6, 10]. To understand the biomineralization process of nacre, we sequenced and assembled the *P. f. martensii* genome and generated transcriptomes from 11 organs/tissues and 12 developmental stages, along with the proteomes of shell organic matrices.

Data description

We used a pearl oyster from a third-generation line selected for fast growth for sequencing and assembly using a BAC-to-BAC strategy. In addition to BAC sequencing, we also constructed whole genome shotgun (WGS) libraries including 3 with short insert-sizes and 4 with long insert-sizes. We used a draft assembly from a previous study [10], Sanger-sequenced BACs and transcripts generated by RNA-seq to assess the integrity of our assembly. Furthermore, to anchor scaffolds to chromosomes, we constructed a genetic map using restriction-site associated DNA sequencing (RAD-seq) using 148 F1 offspring obtained by crossing two genetically distant parents.

To determine gene expression profile in different organs or tissues, we performed transcriptome sequencing on eleven organs and tissues, including adductor muscle,

mantle, mantle pallium, mantle edge, hepatopancreas, hemocyte, gonad, gill, foot, and pearl sac at 180 days (d) after nucleus transplantation. Further, we performed transcriptome sequencing on 12 developmental samples to determine gene expression profile during development. Developmental samples included unfertilized eggs, 11 samples obtained at 30 min, 5 h, 6 h, 8 h, 16 h, 19 h, 4 d, 14 d, 28 d, 40 d, and 90 d after fertilization. To understand gene regulation during nacre formation, we analyzed transcriptome data from mantle edge, mantle pallial and two entire mantle tissues representing fast and slow growing pearl oysters with WGCNA (weighted-gene co-expression network analysis), and obtained co-expression network patterns. All sequencing and genome data were uploaded to GigaDB under the accession number BioProject:PRJNA283019.

Results

Genome assembly and characterization. As our initial assembly of ~130 Gb (134-fold coverage) of whole-genome shotgun (WGS) Illumina sequences (Additional file 1: Table S1) was too fragmented for annotation and analysis, probably due to high polymorphism and repetitive sequences (Additional file 2: Figure S1a and b), we subsequently adopted a BAC-to-BAC (bacterial artificial chromosome) sequencing strategy [6, 11]. We sequenced 46,080 BACs (5-fold genome coverage) to a depth of 100X using Illumina next-generation sequencing (NGS), assembled each BAC separately (Additional file 2: Figure S1c), and then built supercontigs after merging and filtering redundant sequences. After constructing scaffolds and filling gaps with WGS reads, we obtained a final assembly of 990,658,107 bp with a contig N50 size of 21 kb and a scaffold N50 of 324 kb (Additional file 1: Table S2), which was a significant improvement compared with the contig N50 of 1.6 kb of the previous draft assembly [10].

The coverage of our assembly was demonstrated by the successful mapping of 90.5% of contigs, 95.5% (coverage ≥50%) of gene-model regions of the previous draft assembly [10], 99.8% of transcripts (coverage ≥50%), and all four BACs (coverage ≥93.2%) sequenced with Sanger technology (Additional file 1: Table S3 and S4; Additional file 3: Figure S2). We constructed a high-density genetic map of 14 linkage groups in accordance with the haploid number, using RAD-seq of a full-sib family (Fig. 1). We were able to anchor 857.07 Mb (86.5%) scaffolds to the genetic map with 4,463 single-nucleotide polymorphisms (SNPs) (Additional file 1: Table S5;

Fig. 1b). Through alignment of our pseudochromosomes to that of *Crassostrea gigas*, we identified 2,240 syntenic blocks and several possible chromosome rearrangements (Fig. 1c).

Combining *de novo* prediction and evidence-based annotation using published data and transcriptomes from 11 organs/tissues and 12 developmental stages (Additional file 1: Table S6), we identified 32,937 protein-coding gene models (Additional file 1: Table S7), which is comparable to the gene numbers found in *Capitella teleta* (32,389) and *C. gigas* (28,027) but higher than those in *Drosophila melanogaster* (23,847), *Helobdella robusta* (23,400) and *Lottia gigantea* (23,800). Searches against public databases showed that 84.0% of the gene models matched known proteins (Additional file 1: Table S8). Further, BUSCO analysis shows that 82.8% of predicted genes are completed and 7.4% of them are fragmented, indicating our assembly is adequate for further analysis. To assess the impact of selection, we determined codon usage, GC content of intron, exon and inter-genic regions, and GC content at each codon position, which were similar in *P. f. martensii* and other 8 species (Additional file 4: Figure S3).

Phylogenetic analysis of the sequenced genomes of *P. f. martensii*, *C. gigas* and *L. gigantea* along with selected model organisms provided estimates of divergence times: 485 million years ago (mya) between *P. f. martensii* (Bivalvia) and *L. gigantea* (Gastropoda) and 316 mya between *P. f. martensii* (Pteriidae) and *C. gigas* (Ostreidae) (Additional file 5: Figure S4). These estimates are in agreement with the most up-to-date phylogenetic analyses of molluscan evolution [12]. Compared to *Homo sapiens* and *Danio rerio*, molluscan genomes do not have transforming growth factor (TGF)-beta factors but only bone morphogenetic proteins (BMPs), but these two proteins share a common origin with TGF-beta being derived from BMPs (Additional file 1: Table S10, S11 and S12; Additional file 6: Figure S5,). TGF-beta factors are crucial in regulating osteoblast proliferation, differentiation and bone matrix maturation in vertebrates [13, 14]. This finding suggests that molluscs have maintained an ancient BMP-regulatory system for shell formation [15], while TGF-beta emerged in vertebrates to regulate bone matrix.

Chitin is a basic component of the nacre matrix. Consistent with the matrix model of molluscan shell formation, we demonstrated the abundant presence of chitin in the shell matrix of *P. f. martensii* (in both prismatic and nacreous layers) and *C. gigas* (mostly prismatic) by Calcofluor white M2R staining (Additional file 7: Figure S6a).

Transcriptome analysis of different tissues indicated that some *chitin synthases* (*CHSs*) and *chitinase* were highly expressed in the mantle and pearl sac, the two main calcifying tissues responsible for shell and pearl formation (Additional file7: Figure S6b and S6c). During larval development, some *CHSs* and *chitinases* were highly expressed at the trochophore and post-veliger stages (Additional file 7: Figure S6d), corresponding to prodissoconch and dissoconch/adult shell formation, respectively. Furthermore, the gene family of *CHS* was significantly expanded in *P. f. martensii* and other shelled molluscs, but not in molluscs without shells, *Octopus bimaculoides* (Additional file 1: Table S10). These results suggest that chitin is a key component of the shell matrix, and *CHS* genes in *P. f. martensii* and other shelled molluscs might have played crucial roles in the evolution of advanced shells in molluscs.

The presence and involvement of VWA containing proteins (VWAP). According to the current model, silk proteins are major components of the organic matrix in molluscan shells. We searched for silk proteins in the *P. f. martensii* genome and the proteome of the shell matrix but found none. Interestingly, a total of 10 VWAPs were detected in the nacre proteome with 372 unique spectra, compared with 146 spectra in the prismatic layer proteome (Additional file 8: Datasets S1). Among the 10 VWAPs, 8 VWAPs specifically existed in the nacre and not found in the prismatic layer proteome, and 8 VWAPs contained VWA domains that show highest sequence homology with VWA domains of human or mouse collagens (Additional file 1: Table S13). Corresponding to the abundance of VWAPs in the nacre proteome, the *P. f. martensii* genome has an expanded family of 164 VWAPs, similar to the 162 found in *C. gigas* [6] but more than the 94 found in *L. gigantea* and 91 in humans (Fig. 2a). The 10 *VWAPs* were highly expressed in the mantle pallium and pearl sac, which are responsible for nacreous layer production (Fig. 2b). All 10 *VWAPs* were up-regulated (at least 5X of the level in egg) in post-veliger larvae with nacreous/aragonite shells, again suggesting their crucial role in nacreous matrix formation. Meanwhile, two of the 10 *VWAPs* (Pma_10019835, Pma_10011421) was significantly up-regulated (40X of egg) at the trochophore stage, in correlation with aragonite shell formation (Fig. 2c). After inhibition of six *VWAPs* (Pma_44.534, Pma_530.149, Pma_10011175, Pma_10019835, Pma_10019836, Pma_10015641) by RNA interference, the microstructure of the nacre showed disordered growth, as observed by scanning electron microscopy (SEM) (Fig. 2d and Additional file 9: Figure S7). These results suggest that VWAPs are a major component of the nacreous organic matrix and play a

key role in nacreous shell formation in *P. f. martensii*. Structure analysis indicated two of the 10 VWAPs (Pma_10015641 and Pma_10011421) has a chitin-binding domain, supporting its possible function in interacting with the chitin framework during matrix formation. Surprisingly, there are no collagens containing both VWA and triple helix repeat (THRs) in genomes of the three molluscs analyzed (*P. f. martensii*, *C. gigas* and *L. gigantea*). Collagens with VWA and THR are found in some invertebrates (*C. teleta*, *H. robusta* and *Mytilus coruscus* [16]) (Additional file 10: Figure S8), suggesting that THRs may be lost in some lineages. THR containing genes showed contraction in the mollusc genomes, over expansion in annulata and vertebrates (Fig. 2a).

Acidic glycosaminoglycans (GAGs) constitute a gel-like substance. According to the matrix model, the shell matrix contains a gel-like substance where acidic proteins induce the nucleation of calcium carbonate crystals [17]. Consistent with the model and previous reports, we identified a list of acid proteins that might be involved in shell formation (Additional file 8: Datasets S2). In addition to the acidic proteins that are unique to molluscan shells, we also found acidic GAGs, fibronectin-like proteins and chondroitin sulfotransferases that are characteristic components of vertebrate bone matrices. By Alcian blue-periodic acid Schiff staining (AB-PAS), we found that the organic matrix extracted from nacreous shells contained large amounts of acidic GAGs compared with mainly neutral GAGs in prismatic layers of *P. f. martensii* and *C. gigas* shells. In addition, we detected acid GAGs in secretory cells of the mantle pallium of *P. f. martensii*, but mainly neutral GAGs in the mantle of *C. gigas* (Fig. 3a). Further, our data show that the *P. f. martensii* genome has an expanded set of five types of sulfotransferase (Additional file 1: Table S10), including chondroitin 4-sulfotransferase 11 (CHST11), chondroitin 6-sulfotransferase 3 (CHST3), carbohydrate 6-sulfotransferase 6 (CHST6), carbohydrate 4-sulfotransferase 9 (CHST9) and dermatan 4-sulfotransferase 1 (D4ST1). Corresponding to large amounts of acidic GAGs in the mantle pallium, some of the sulfotransferases (*CHST3*, *CHST11*, *CHST6* and *D4ST1*) exhibited higher expression levels in the mantle pallium than in the mantle edge (Fig. 3b). *CHST11* and *D4ST1* expressed at the post-veliger stage, whereas *CHST6* and *CHST3* were mostly up-regulated at the trochophore stage (Additional file 11: Figure S9a).

Tyrosinase may participate in the nacre matrix cross-linking. *C. gigas* has an expanded set of 26 *tyrosinases* (*Tyrs*) [6], and we observed an even larger expansion of *Tyrs* in *P. f. martensii* to 53 genes compared with 3 genes in *L. gigantea*, 1 in humans and 4 in coral (Additional file 1: Table S10). Phylogenetic analysis of these Tyrs from *P. f. martensii* and *C. gigas* revealed unbalanced and lineage-specific expansion in both species (Fig. 3c, Additional file 1: Table S14). Their expression profiles in calcifying tissues and at shelled larval stages indicate that 29 of the expanded *P. f. martensii Tyr*s may be involved in shell formation, among which 23 were highly expressed after the post-veliger/spat stage, pointing to possible functions in adult shell formation (Fig. 3c). Seven *Tyr*s showed high expression levels in the mantle pallium (MP, Additional file 11: Figure S9b) and 9 *Tyrs* highly expressed in the pearl sac (Additional file 11: Figure S9c), compared with 13 *Tyrs* highly expressed in mantle edge (ME). Twelve Tyrs were identified from shell proteome: 2 specific to nacreous layer and highly expressed in MP, 4 specific to prismatic layer and highly expressed in ME and 6 found in both nacreous and prismatic layers. Greater abundance of quinoproteins was observed in the nacreous than in the prismatic layers (Additional file 11: Figure S9d). These results indicate that dopaquinone catalysed by Tyr may be essential for the assembly and maturation of both nacreous and prismatic shell matrices.

Regulation network of the nacre matrix proteins. WGCNA of the 234 nacre matrix protein genes revealed 27 hub genes at the centre of the network (Fig. 4, Additional file 8: Datasets S3 and S4), including well-known as well as novel genes for shell formation, such as *fibronectin III*, *VWAP* and *Tyr*, which reinforced our findings and demonstrated the usefulness of WGCNA. In addition, heat shock protein 70 (Hsp70), proteinase inhibitor I2-containing proteins and proteins with chitin-binding domains were also included in the hub genes, indicating their possible roles in nacre formation. Furthermore, we filtered the adjacent coefficients (no less than 0.5) and obtained 3245 crucial genes co-expressed with the nacre matrix proteins. These co-expressed crucial genes were significantly enriched ($P<0.05$) in the ErbB signalling pathway, the Jak-STAT signalling pathway, the Wnt signalling pathway, osteoclast differentiation signalling pathways, ECM-receptor interactions and the vascular endothelial growth factor (VEGF) signalling pathway, which are all involved in bone formation (Additional file 1: Table S15). Meanwhile, metabolism of polysaccharide such as glycosaminoglycan, N-glycan and O-glycan were also implicated. Analysis by Gene

Ontology indicated that genes related to transmembrane transporter activity were significantly enriched, which is consistent with the enrichment of ABC transporters in KEGG analysis (Additional file 1: Table S15, S16).

Discussion

The assembly of highly polymorphic genomes and gene prediction in non-model organisms remain challenging. Software based on *de Bruijn* Graph, such as SOAP*denov*o [18], is inadequate in producing satisfactory results due to the increased complexity of *de Bruijn* graph structure. Overlap-Layout-Consensus assembler, such as Celera Assembler [19], based on the data of fosmids or BACs hierarchical sequencing and third-generation long reads (such as PacBio long reads) are employed to overcome such problems. However, the best choice for assembling complex genomes is to sample haploid or homozygous sequences. For the *ab initio* gene prediction software, such AUGUSTUS [20], the aim is to find potential coding sequences with sufficiently long open reading frames, but the translated regions may be too short making the absence of stop codons meaningless. The similarity-based approaches including homologous protein sequences, EST sequences and transcripts assembled from RNA-seq reads can produce biologically relevant predictions, but they may not cover all coding exons. Considering their strengths and weaknesses, synthesis software, such as GLEAN [21] and MAKER [22], were used to synthesize these evidences obtained from *ab initio* gene predictions and similarity-based approaches into the final gene annotation. BUSCO [23] analysis indicates our assembly is sufficiently complete.

The aragonite nacre that gives pearls and certain shells their lustre and enhanced toughness is the target of many studies and modelling. According to the matrix model of molluscan shell formation, the mineralization of calcium carbonate is directed by a mantle-secreted organic matrix [24, 25], which is not fully understood but may contain chitin [26-28] and silk fibroin [29-31] for the structural framework and soluble acidic proteins for crystal nucleation [32-34]. Alternatively, the cellular hypothesis argues that biomineralization may be directed by hemocytes [7, 35] although there is no dispute about the involvement of organic matrices which are the focus of our study. Chitin is an ancient macromolecule and the primary framework component of organic matrices in cell walls of fungi and diatoms, sponge skeletons and arthropod shells [3]. It is possible that the chitin component of lophotrochozoan

and ecdysozoan shells and of sponge skeletons constitute a shared feature and have the same ancient origin. Our results provide strong evidence that chitin is the basic component of *P. f. martensii* shell matrices.

While silk proteins, which are also considered as the major components of the organic matrix in the molluscan shell, were not found in the *P. f. martensii* genome and the proteome of the nacreous shell matrix, abundant signatures of expanded VWAPs were detected in the nacre proteome. VWAPs were also reported to be found in *C. gigas*, *Mytilus edulis* and *Pecten maximus* [36]. The VWA domains are a family of 200-amino-acid residues and function as interaction modules in many intra- and extracellular proteins, such as copines, integrins, von Willebrand factor, complement factors B and C2, matrilins, and collagens [37]. Collagens are a large family of extracellular matrix proteins with typical THRs. Eight of the 28 known collagens (collagen VI, VII, XII, XIV, XX, XXI, XXII, and XXVIII) contain VWA domains in addition to THRs. The finding that VWAs of VWAPs from shell matrix show the highest homology with VWAs of vertebrate collagens, suggest that these VWAPs and vertebrate collagens may have a common origin. It is possible that collagens with VWAs are evolved from VWAPs through the addition of THRs, and VWAPs of *P. f. martensii* represents an ancient form that never acquired THRs. It is also possible and that some of the VWAPs were collagens that lost THRs. Collagens with both VWAs and THRs are found in some invertebrates such as *C. teleta*, *H. robusta* and *Mytilus coruscus* [16], but not in *P. f. martensii*, *C. gigas* and *L. gigantea*, which argues for the loss of THRs in some molluscan lineages.

THRs are crucial for the self-assembly of collagen subunits into triple-helix protomers and the formation of fibrillar collagens [38, 39]. The absence of THRs in VWAPs indicates that VWAPs may function differently in the nacreous shells of *P. f. martensii* from collagens in bone. VWAPs may not self-assemble into fibrous structures but instead cross-link with each other and other matrix proteins to form a network structure [40-42]. The finding of VWAPs with chitin-binding domains further highlights their function in interacting with the chitin framework during matrix formation. In addition, VWA domains bind to positive ions that attract water, and may cooperate with GAGs or other proteins and provide initial hydrogel properties for biomineralization [37].

Mammalian cartilage and bone matrices consist of collagen fibrils and a gel-like ground substance that is rich in chondroitin-containing proteoglycans, fibronectins

and link proteins [43]. Our results confirm the presence of fibronectin-like proteins in shells of *P. f. martensii* and *C. gigas* [6]. Proteoglycans or GAGs, which have strong water-binding capabilities and have been detected in the shell [29], may function as the gel-like substance [44]. In the nacre and the secretory cells of the mantle pallium of *P. f. martensii,* we found large amounts of acidic GAGs, which have also been detected in coral [45] and bone [46], and this finding argues that the acidic GAGs might also play key roles in crystal nucleation during nacre formation. Combining this finding with the findings of collagen-related VWAPs and other elements shared by bone formation, our results suggest that the nacreous shell matrix, while having a chitin-based framework, also possesses key elements of collagen-based matrices, such as fibronectins, proteoglycans and chondroitin sulfotransferases. Chitin- and collagen-based matrices are considered as two basic types of biomineralizing framework, and our results suggest that they may share some basic components and have a common origin, or might have co-existed as parts of an ancient/ancestral matrix with dual-elements, despite subsequent divergence in different taxa into chitin- or collagen-based organic matrices.

The shell organic matrix, rather than being a simple self-assembling structure, might instead be a complex and dynamic matrix that requires active construction, regulation and remodelling. Tyrs, which can catalyse the formation of dopa and dopaquinone, were highly abundant in the shell of bivalves, and may function in mediating intermolecular cross-links [6, 47], or as a structural component of the shell. Tyrs belong to the "type-3 copper" family and have a conserved active site of six histidine residues mediating the binding of copper ion as cofactor [48]. Metal ions such as Cu^{2+}, Zn^{2+} and Mg^{2+} are important factors for stabilizing the crystalline form of calcium carbonate [49-51]. Therefore, the deposition of Tyrs and associated metal ions in the matrix may regulate metal ion concentration in the extrapallial fluid and help to stabilize the crystalline form. Interestingly, we found that the histidine residues were retained in the 4 prism-specific Tyrs but mostly lost in the two nacre-specific Tyrs (Pma_10005159 and Pma_10016044), suggesting possible divergence in metal ion binding capability between nacre-specific and prism-specific Tyrs. It should be noted that many of expanded Tyrs may be unrelated to shell formation as shell-less *Octopus bimaculoides* also shows some expansion (Table S10), and instead they may function in their well-established roles in melanin pigment production, wound healing and immune responses in *P. f. martensii* also [52].

The complexity of the shell matrix and biomineralization processes is further demonstrated by co-expression network analysis, which indicates that genes related to polysaccharide metabolism are significantly co-expressed with nacre proteins. This result is consistent with the abundance of chitin and acid GAGs in the nacreous layer. Interestingly, nacre proteins were also co-expressed with ABC-transporters known as ATP-dependent transport proteins. ABC-transporters may mediate the secretion of proteins without signal peptide [53], which are not uncommon among nacre proteins and may be also secreted through other mechanisms such as exosomes [6]. Some nacre proteins without signal peptide may be due to assembly and annotation errors. More importantly, signal pathway related to bone formation, such as Wnt signalling pathway and osteoclast differentiation signalling pathway, were also implicated. Together, these results suggest that molluscan shell formation is an elaborate and dynamic process that shares certain basic elements with mammalian bone formation, but with added complexity. Although molluscan shells have a chitin-dominated framework, the identification of key elements shared by collagen-based matrices supports a single origin for the two types of matrices or a common set of tools that may have been lost, modified and reorganized during evolution to produce diverse forms of biomineralized structures in adaptation to new environments and in assuming new functions.

In conclusion, we sequenced and assembled the highly polymorphic genome of *P. f. martensii* using NGS and the BAC-to-BAC strategy. Based on genomic, transcriptomic, and proteomic analyses and experimental studies, we identified a large number of genes related to shell nacre formation, which helped us to re-construct the shell matrix model (Fig. 5). The identification of collagen-related VWAPs and other elements of collagen-based matrices in the chitin-rich nacre matrix supports the homology and single evolutionary origin of the common biomineralization toolkit. The hypothesis of a single evolutionary origin challenges the prevailing idea of independent evolution [2] and may stimulate homology-based studies towards a better understanding of the diverse forms of biomineralization.

Methods

SI Appendix has additional information relating to the methodologies described below.

Library construction and sequencing. We constructed all sequencing libraries

according to protocols from Illumina and sequenced these libraries on a HiSeq 2000 sequencing system.

Hierarchical BAC-to-BAC assembly strategy. We used a hierarchical BAC-to-BAC assembly approach as used for the moth genome [11]. Before the hierarchical assembly of BACs, we used SOAPdenovo to assemble the reads of each BAC with odd numbered K-mers from 27 to 63 and selected the best results with the longest scaffold N50 and total length, as primary scaffolds. Then, we used the paired-end reads information of the BACs and locally assembled the reads in the gap regions to fill in the gaps within the primary BAC scaffolds. Our custom assembly software (Rabbit) [11] was used to assemble scaffolds of BACs with large overlaps. After finding relationship among sequences, merging overlapping sequences and removing redundant sequences, we obtained longer segments as secondary scaffolds. Finally, SSPACE was used to join the secondary scaffolds to form final scaffolds, and SOAP-Gapcloser was used to fill in the gaps in the final scaffolds using all WGS reads with short insert sizes.

Linkage group construction. We constructed a genetic map using RAD-seq of 148 F1 progeny from a family obtained by crossing two genetically distant parents. We used SOAP2 [54] to map the reads to the reference genome sequences of *P. f. martensii* (scaffolds) and performed SNP calling using SOAPsnp [55]. After SNP calling, we extracted genotypes by combining all SNPs among the 148 progeny and the 2 parents and constructed linkage map using JoinMap 4.1 [56].

Phylogenetic tree construction and divergence time estimation. We used Treefam to obtain gene families and one-to-one orthologs, and used MrBayes to construct the phylogenetic tree.

Transcriptome analysis. We extracted total RNA from each sample and isolated mRNA using oligo (dT) magnetic beads. Then, the mRNA was fragmented into short fragments (200~500 bp) for construction of RNA-seq libraries that were sequenced on an Illumina HiSeq2000. Using SOAP2, all clean reads were mapped to the genome assembly with less than 5 mismatches. We used the *RPKM* method (Reads per kilobase transcript per million mapped reads) to calculate the gene expression levels. We also tested TPM (Transcripts Per Million) [57] for quantifying gene expression and found excellent correspondence between RPKM and TRM for our samples.

Identification of the matrix proteins. We used the Mascot software (v 2.3.02) to query the MS/MS spectra data of matrix proteins in the database. We applied the

trypsin cleavage rule with one missed cleavage site. Carbamidomethylation of cysteines was considered as the fixed modifications while Gln->pyro-Glu (N-term Q), Oxidation (M) and Deamidated (NQ) were considered as the variable modifications. Peptide mass tolerance was set to 0.05Da and fragment mass tolerance was set to 0.01Da. We used target-decoy search strategy [58] to identify the matrix proteins, and the False Discovery Rate (PDR) was ≤1%.

Extraction of matrix proteins from the nacre and prismatic layer. Shells of freshly collected oysters were thoroughly cleaned by hand and treated with sodium hypochlorite solution (6-14% active chlorine) to remove organic surface contaminants [59]. The prismatic layer was separated from the edges of pearl oyster shells without nacre. The nacre was directly scraped from the internal shell surfaces dominated by aragonite. These samples were thoroughly ground and soaked in acetic acid solution (5%, v/v) for at least 12 h to dissolve calcium carbonate, before being centrifuged at 14,000 g and 4 °C for 1 h. Acid-soluble proteins were in the supernatant, and acid-insoluble proteins were in the residue.

Samples were electrophoresed on 12% polyacrylamide gels and stained with Coomassie blue R-250. The extracted peptides were dried and stored at -80 °C until liquid chromatography/tandem mass spectrometry (LC-MS/MS) analysis.

Chitin identification in shell matrix. We decalcified the shells in 1 M acetic acid at 4 °C for one week, and the acid-insoluble material was collected. This insoluble material was washed with distilled water and embedded in paraffin for sectioning. The sections were placed on slides and stained for 5 min with 0.1% Calcofluor White M2R (Flupstain Ⅰ) (Sigma-Aldrich). Excess dye was rinsed off with distilled water. The stained specimens were observed under a confocal laser microscope using filters with 492 nm excitation and 520 nm emission [60].

RNAi experiment. The primers used for generating the *VWAPs* double-strand RNA (dsRNA) are shown in Additional file 1: Table S17. DsRNAs were synthesized following the method of Suzuki et al. [61], and injected into the adductor muscle every 4 days at 100 μg per 100 μl per pearl oyster each time. The effects of RNAi of the six *VWAPs* genes on nacre formation were detected by SEM.

Identification of GAGs in shell and pearl. Shells were decalcified in 1 M acetic acid at 4 °C for 1 week and then in 10% EDTA-2Na solution at room temperature for 10 days. The fixed materials were embedded in paraffin and stained with AB/PAS (Alcian blue/periodic acid-Schiff) and observed under a OlympusBX51 optical

microscope.

Nitrobluetetrazolium (NBT)/glycinate assay for dopa and dopaquione protein. Sections of decalcified shells were stained with 100 μL of solution containing 0.24 mM NBT and 2 M potassium glycinate (pH10) for nearly 5 min in darkness until violet positive signals appeared [62]. The sections were rinsed with double-distilled water to stop the reaction and then mounted for microscopic examination.

Co-expression network analysis. We used WGCNA to reconstruct the co-expression network for biomineralization [63]. A weighted correlation network was constructed between all pairs of genes across four mantle tissue samples. The adjacency matrix was calculated through a so-called 'soft' thresholding framework (power β=9) that converted the co-expression measure to a connection weight. Based on the adjacency matrix, we implemented a topological overlap dissimilarity measure to reflect relative inter-connectedness, which may represent a meaningful biological network. Hub genes (highly connected genes), by definition, tend to have high connectivity in the constructed network.

Availability of supporting data

Data from the pearl oyster (*P. f. martensii*) genome projects are available from NCBI BioProject:PRJNA283019. Data supporting the manuscript is also available via the GigaDB database [64].

List of abbreviations

CHS: chitin synthases; VWAP: VWA domain containing protein; BMPs: bone morphogenetic proteins; VWA: von Willebrand factor A; CHST11: chondroitin 4-sulfotransferase 11; CHST3: chondroitin 6-sulfotransferase 3; CHST6: carbohydrate 6-sulfotransferase 6; CHST9: carbohydrate 4-sulfotransferase 9; D4ST1: dermatan 4-sulfotransferase 1; Tyr: Tyrosinase; WGCNA: Weighted-gene co-expression network analysis; ITIH4 : Inter alpha-trypsin inhibitor, heavy chain 4; MATN2/3: Matrilin-2/3; Col12A1: Collagen alpha-1(XII) chain; Col14A1: Collagen alpha-1(XIV) chain; Col22A1: Collagen alpha-1(XXII) chain; Col6A3/4/6: Collagen alpha-3/4/6(VI) chain;

Competing interests

The authors declare that they have no competing interests.

Funding

This research is partly supported the Guangdong Ocean University Nature Science Foundation (University program: Genome studies of pearl oyster), the National

Nature Science Foundation of China (31272635, 31372526, 31672626), Modern Agro-industry Technology Research System (CARS-48), USDA/NJAES Project 1004475/NJ32920 and “Taishan Oversea Scholar” program.

Authors' contributions

X.D., X.G., G.Z. and X.L. designed scientific objectives. Y.J., R.H., W.C., X.X, Q.S. and G.F. managed the project. H.Z., F.S., X.L., C.S., W.L., H.L. and Z.W. performed genome assembly, gene annotation and evolution analyses. Y.D., Q.W. and X.Z. cultured P. f. martensii and provided materials. Y.D., Q.W., F.S., J.B. and Z.W. constructed the genetic map. Q.W., Z.Z., J.L. and R.H. performed the acid GAG analysis. Y.J., Z.Z., R.H. and J.L. performed chitin and VWAPs related analyses. R.H., C.B. and Z.Z. performed tyrosinase related analysis. Z.Z. and H.Z. performed the WGCNA analysis. X.D., G.F. and X.G. directed final data analyses. Y.J., X.G. and Z.X. directed critical revisions of intellectual content. X.D., X.G. and Y.J. supervised all aspects of the work to ensure the accuracy or integrity of the research and data. X.D., G.F., Y.J., H.Z., X.G., R.H., Z.Z. did most of the writing with contributions from all authors.

Acknowledgments

We thank Z. He, W. Liu, Z. Wu, C. Liu, J. Jian, B. Tan for their supports of the pearl oyster genome project. We thank Y. Guo, X. Chen, M. Xue and Xuwen Pearl Oyster Farm for assistance with DNA, RNA and protein extraction, data analysis and oyster culture. We thank L. Goodman for helping to edit the manuscript. We thank other faculty and staff at Guangdong Ocean University, BGI-Shenzhen and Rutgers who contributed to the genome project.

Author Information

Correspondence and requests for materials should be addressed to X.D. (zjduxd@126.com), X.G. (xguo@hsrl.rutgers.edu), G.Z. (gzhang@qdio.ac.cn) and X.L. (liuxin@genomics.cn).

Reference

1. Knoll AH. Biomineralization and evolutionary history. Rev Mineral Geochem. 2003;54:329-56.
2. Drake JL, Mass T, Falkowski PG. The evolution and future of carbonate precipitation in marine invertebrates: Witnessing extinction or documenting resilience in the Anthropocene? Elementa Science of the Anthropocene.

2014;2:000026.
3. Ehrlich H. Chitin and collagen as universal and alternative templates in biomineralization. Int Geol Rev. 2010;52:661-99.
4. Furuhashi T, Schwarzinger C, Miksik I, Smrz M, Beran A. Molluscan shell evolution with review of shell calcification hypothesis. Comp Biochem Phys B. 2009;154:351-71.
5. Addadi L, Joester D, Nudelman F, Weiner S. Mollusk shell formation: a source of new concepts for understanding biomineralization processes. Chem-Eur J. 2006;12:980-7.
6. Zhang G, Fang X, Guo X, Li L, Luo R, Xu F, et al. The oyster genome reveals stress adaptation and complexity of shell formation. Nature. 2012;490:49-54.
7. Mount AS, Wheeler AP, Paradkar RP, Snider D. Hemocyte-mediated shell mineralization in the eastern oyster. Science. 2004;304:297-300.
8. Marin F, Luquet G, Marie B, Medakovic D. Molluscan shell proteins: primary structure, origin, and evolution. Curr Top Dev Biol. 2007;80:209-76.
9. Murdock DJ, Donoghue PC. Evolutionary origins of animal skeletal biomineralization. Cells Tissues Organs. 2011;194:98-102.
10. Takeuchi T, Kawashima T, Koyanagi R, Gyoja F, Tanaka M, Ikuta T, et al. Draft genome of the pearl oyster *Pinctada fucata*: a platform for understanding bivalve biology. DNA Res. 2012;19:117-30.
11. You M, Yue Z, He W, Yang X, Yang G, Xie M, et al. A heterozygous moth genome provides insights into herbivory and detoxification. Nat Genet. 2013;45:220-5.
12. Murgarella M, Puiu D, Novoa B, Figueras A, Posada D, Canchaya C. A First Insight into the Genome of the Filter-Feeder Mussel *Mytilus galloprovincialis*. Plos One. 2016;11:e0151561.
13. Zhang H, Ahmad M, Gronowicz G. Effects of transforming growth factor-beta 1 (TGF-β1) on in vitro mineralization of human osteoblasts on implant materials. Biomaterials. 2003;24:2013-20.
14. Miron RJ, Saulacic N, Buser D, Iizuka T, Sculean A. Osteoblast proliferation and differentiation on a barrier membrane in combination with BMP2 and TGFβ1. Clin Oral Invest. 2013;17:981-8.
15. Yan F, Luo S, Jiao Y, Deng Y, Du X, Huang R, et al. Molecular characterization of the BMP7 gene and its potential role in shell formation in *Pinctada martensii*. Int J Mol Sci. 2014;15:21215-28.
16. Qin CL, Pan QD, Qi Q, Fan MH, Sun JJ, Li NN, et al. In-depth proteomic analysis of the byssus from marine mussel *Mytilus coruscus*. J Proteomics. 2016;144:87-98.
17. Nudelman F, Shimoni E, Klein E, Rousseau M, Bourrat X, Lopez E, et al. Forming nacreous layer of the shells of the bivalves Atrina rigida and *Pinctada margaritifera*: an environmental-and cryo-scanning electron microscopy study. J Struct Biol. 2008;162:290-300.
18. Luo R, Liu B, Xie Y, Li Z, Huang W, Yuan J, et al. SOAPdenovo2: an empirically improved memory-efficient short-read de novo assembler.

GigaScience. 2012;1:18.
19. Myers EW, Sutton GG, Delcher AL, Dew IM, Fasulo DP, Flanigan MJ, et al. A whole-genome assembly of Drosophila. Science. 2000;287:2196.
20. Stanke M, Keller O, Gunduz I, Hayes A, Waack S, Morgenstern B. AUGUSTUS: ab initio prediction of alternative transcripts. Nucleic Acids Res. 2006;34:W435-9.
21. Elsik CG, Mackey AJ, Reese JT, Milshina NV, Roos DS, Weinstock GM. Creating a honey bee consensus gene set. Genome Biol. 2007;8: R13.
22. Cantarel BL, Korf I, Robb SMC, Parra G, Ross E, Moore B, et al. MAKER: An easy-to-use annotation pipeline designed for emerging model organism genomes. Genome Res. 2008;18:188-96.
23. Simão FA, Waterhouse RM, Ioannidis P, Kriventseva EV, Zdobnov EM. BUSCO: assessing genome assembly and annotation completeness with single-copy orthologs. Bioinformatics. 2015;31:3210.
24. Addadi L, Weiner S. Biomineralization: A pavement of pearl. Nature. 1997;389:912-5.
25. Nassif N, Pinna N, Gehrke N, Antonietti M, Jäger C, Cölfen H. Amorphous layer around aragonite platelets in nacre. P Natl Acad Sci USA. 2005;102:12653-5.
26. Levi-Kalisman Y, Falini G, Addadi L, Weiner S. Structure of the nacreous organic matrix of a bivalve mollusk shell examined in the hydrated state using Cryo-TEM. J Struct Biol. 2001;135:8-17.
27. Weiss IM, Schönitzer V. The distribution of chitin in larval shells of the bivalve mollusk *Mytilus galloprovincialis*. J Struct Biol. 2006;153:264-77.
28. Furuhashi T, Beran A, Blazso M, Czegeny Z, Schwarzinger C, Steiner G. Pyrolysis GC/MS and IR spectroscopy in chitin analysis of molluscan shells. Biosci Biotech Bioch. 2009;73:93-103.
29. Pereira- Mouriès L, Almeida MJ, Ribeiro C, Peduzzi J, Barthélémy M, Milet C, et al. Soluble silk- like organic matrix in the nacreous layer of the bivalve *Pinctada maxima*. Eur J Biochem. 2002;269:4994-5003.
30. Sudo S, Fujikawa T, Nagakura T, Ohkubo T, Sakaguchi K, Tanaka M, et al. Structures of mollusc shell framework proteins. Nature. 1997;387:563-4.
31. Nudelman F, Chen HH, Goldberg HA, Weiner S, Addadi L. Spiers Memorial Lecture Lessons from biomineralization: comparing the growth strategies of mollusc shell prismatic and nacreous layers in *Atrina rigida*. Faraday discussions. 2007;136:9-25.
32. Weiner S, Hood L. Soluble protein of the organic matrix of mollusk shells: a potential template for shell formation. Science. 1975;190:987-9.
33. Fu G, Valiyaveettil S, Wopenka B, Morse DE. CaCO3 Biomineralization: Acidic 8-kDa Proteins Isolated from Aragonitic Abalone Shell Nacre Can Specifically Modify Calcite Crystal Morphology. Biomacromolecules. 2005;6:1289-98.
34. Evans JS. "Tuning in" to Mollusk Shell Nacre-and Prismatic-Associated Protein Terminal Sequences. Implications for Biomineralization and the

Construction of High Performance Inorganic− Organic Composites. Chem Rev. 2008;108:4455-62..
35. Li S, Liu Y, Liu C, Huang J, Zheng G, Xie L, et al. Hemocytes Participate in Calcium Carbonate Crystal Formation, Transportation and Shell Regeneration in the Pearl Oyster *Pinctada fucata*. Fish Shellfish Immun. 2016;51:263-70.
36. Arivalagan J, Yarra T, Marie B, Sleight VA, Duvernoisberthet E, Clark MS, et al. Insights from the Shell Proteome: Biomineralization to Adaptation. Mol Biol Evol. 2016;34:66-77.
37. Whittaker CA, Hynes RO. Distribution and evolution of von Willebrand/integrin A domains: widely dispersed domains with roles in cell adhesion and elsewhere. Mol Biol of cell. 2002;13:3369-87.
38. Kadler KE, Bella BJ, Boot-Handford RP. Collagens at a glance. J Cell Sci. 2007;120:1955-8.
39. Van der Rest M, Garrone R. Collagen family of proteins. The FASEB journal. 1991;5:2814-23.
40. Becker A-KA, Mikolajek H, Paulsson M, Wagener R, Werner JM. A structure of a collagen VI VWA domain displays N and C termini at opposite sides of the protein. Structure. 2014;22:199-208.
41. Fitzgerald J, Mörgelin M, Selan C, Wiberg C, Keene DR, Lamandé SR, et al. The N-terminal N5 subdomain of the α3 (VI) chain is important for collagen VI microfibril formation. J Biol Chem. 2001;276:187-93.
42. Suhre MH, Gertz M, Steegborn C, Scheibel T. Structural and functional features of a collagen-binding matrix protein from the mussel byssus. Nat Commun. 2014;5:3392.
43. Heinegård D, Oldberg A. Structure and biology of cartilage and bone matrix noncollagenous macromolecules. The FASEB Journal. 1989;3:2042-51.
44. Mow VC, Ratcliffe A, Poole AR. Cartilage and diarthrodial joints as paradigms for hierarchical materials and structures. Biomaterials. 1992;13:67-97.
45. Goldberg WM. Acid polysaccharides in the skeletal matrix and calicoblastic epithelium of the stony coral *Mycetophyllia reesi*. Tissue Cell. 2001;33:376–87.
46. Vejlens L. Glycosaminoglycans of human bone tissue. Calcified tissue research. 1971;7:175-90.
47. Aguilera F, Mcdougall C, Degnan BM. Evolution of the tyrosinase gene family in bivalve molluscs: Independent expansion of the mantle gene repertoire . Acta Biomater. 2014;10:3855-65.
48. Decker H, Schweikardt T, Tuczek F. The first crystal structure of tyrosinase: all questions answered? ChemInform. 2006;45:4546–50.
49. Kitano Y, Kanamori N, Yoshioka S. Adsorption of zinc and copper ions on calcite and aragonite and its influence on the transformation of aragonite to calcite. Geochem J. 1976;10:175-9.
50. Berner R. The role of magnesium in the crystal growth of calcite and aragonite from sea water. Geochim Cosmochim Ac. 1975;39:489-504.

51. Nassrallah-Aboukais N, Boughriet A, Laureyns J, Aboukais A, Fischer J, Langelin H, et al. Transformation of vaterite into cubic calcite in the presence of copper (II) species. Chem Mate. 1998;10:238-43.
52. Kanteev M, Goldfeder M, Fishman A. Structure–function correlations in tyrosinases. Protein Sci. 2015;24:1360-9.
53. Higgins CF. ABC transporters: from microorganisms to man. Annu Rev Cell Bio. 1992;8:67-113.
54. Hecker A, Mikulski Z, Lips KS, Pfeil U, Zakrzewicz A, Wilker S, et al. Pivotal Advance: Up-regulation of acetylcholine synthesis and paracrine cholinergic signaling in intravascular transplant leukocytes during rejection of rat renal allografts. J Leukoc Biol. 2009;86:13-22.
55. Li R, Li Y, Fang X, Yang H, Wang J, Kristiansen K, et al. SNP detection for massively parallel whole-genome resequencing. Genome Res. 2009;19:1124-32.
56. Van Ooijen J. Multipoint maximum likelihood mapping in a full-sib family of an outbreeding species. Genet Res. 2011;93:343-9.
57. Wagner GP, Kin K, Lynch VJ. Measurement of mRNA abundance using RNA-seq data: RPKM measure is inconsistent among samples. Theory Biosci. 2012;131:281-5.
58. Elias JE, Gygi SP. Target-decoy search strategy for increased confidence in large-scale protein identifications by mass spectrometry. Nat methods. 2007;4:207-14.
59. Mann K, Edsinger-Gonzales E, Mann M. In-depth proteomic analysis of a mollusc shell: acid-soluble and acid-insoluble matrix of the limpet *Lottia gigantea*. Proteome Sci. 2012;10:1.
60. Su X, Matthay MA, Malik AB. Requisite role of the cholinergic α7 nicotinic acetylcholine receptor pathway in suppressing gram-negative sepsis-induced acute lung inflammatory injury. J immunol. 2010;184:401-10.
61. Suzuki M, Saruwatari K, Kogure T, Yamamoto Y, Nishimura T, Kato T, et al. An acidic matrix protein, Pif, is a key macromolecule for nacre formation. Science. 2009;325:1388-90.
62. Paz MA, Flückiger R, Boak A, Kagan HM, Gallop PM. Specific detection of quinoproteins by redox-cycling staining. J Bio Chem. 1991;266:689-92.
63. Langfelder P, Horvath S. WGCNA: an R package for weighted correlation network analysis. BMC bioinformatics. 2008;9:1.
64. Xiaodong Du, Guangyi Fan, He Zhang, Yu Jiao, Ximing Guo, Ronglian Huang, et al. Supporting data for " The pearl oyster *Pinctada fucata martensii* genome and multi-omic analyses provide insights into biomineralization ". GigaScience Database. 2017. http://dx.doi.org/xxxxxxxxx.

Figure legend

Figure 1. Genome organization of *P. f. martensii*.

a. Genetic map of *P. f. martensii* constructed with RAD single-nucleotide polymorphisms (SNPs). The lines on linkage groups represent SNP positions. **b.** The

distribution of GC, gene, repetitive elements and SNPs on *P. f. martensii* pseudochromosomes. **c.** Synteny blocks between *C. gigas* (Cg) and *P. f. martensii* (PIN).

Figure 2. Expression and functional analysis of VWAPs in *P. f. martensii*.

a. Number of VWAPs and THR-containing proteins in different species. *P. f. martensii* (Pma), *C. gigas* (Cgi), *L. gigantea* (Lgi), *O. bimaculoides* (Obi), *C. teleta* (Cte), *H. robusta* (Hro), *H. sapiens* (Hsa) and *D. rerio* (Dre). **b.** Expression of 10 genes encoding VWAPs from nacreous shell matrix showing higher expression in the mantle pallium (MP) and pearl sac (PS) than in other organs. Y-axis is the normalized RPKM value. X-axis represents nine organs (MP, mantle pallium; ME, mantle edge; A, adductor muscle; He, hepatopancreas; BC, hemocyte; Go, gonad; Gi, gill; F, foot; PS, pearl sac at 180 days after nucleus transplantation). **c.** Expression pattern of the 10 *VWAPs* during early development and the homology of their VWA domains to that from human and mouse proteins. E, egg; Fe, fertilization; B, blastula; G, gastrula; ET, early trochophore; T, trochophore; D, D-larvae; DF, D-shaped larvae before feeding; EU, early umbo larvae; U, eye-larvae; PV, post-veliger; J, juveniles. **d**. Expression of *Pma_10015641* and *Pma_44.543* and nacre growth after RNA interference. Left: Relative expression of *Pma_10015641* and *Pma_44.534* in mantle after RNAi; PBS, control; RPF, red fluorescent protein; dsRNA, RNAi. Right: Disordered microstructure of nacre observed after inhibition of the two *VWAP* genes (bar = 5μm). col, collagen; ITIH4, Inter-alpha-trypsin inhibitor heavy chain H4; MATR, Matrilin.

Figure 3. GAGs and tyrosinase genes in *P. f. martensii*.

a. The shell matrix extracted from the nacre of *P. f. martensii* contains abundant acid glycosaminoglycans (GAGs) stained blue (I), whereas matrices extracted from the prismatic layer of *P. f. martensii* (I) and *C. gigas* (II) contain neutral GAGs stained red. Secretory cells (arrow) in the mantle pallium of *P. f. martensii* are filled with acid GAGs stained blue (III), whereas cells in the mantle pallium of *C. gigas* contains neutral GAGs stained red (IV). **b.** Expression (y-axis) of *CHST3*, *CHST11*, *CHST6* and *D4ST1* genes in the mantle pallium (MP) and the mantle edge (ME). **c.** Phylogenetic tree of the tyrosinase proteins from *P. f. martensii* and *C. gigas*. Tyrosinase genes specifically expanded in *P. f. martensii* are shaded in purple, and their expression patterns during early development are presented in the heat map. E, egg; Fe, fertilization; B, blastula; G, gastrula; ET, early trochophore; T, trochophore;

D, D-shaped larvae; DF, D-larvae before feeding; EU, early umbo larvae; U, eye-larvae; PV, post-veliger; J, juveniles.

Figure 4. Co-expression network of nacre formation-related genes of *P. f. martensii*.

Hub genes are illustrated in the internal circle, where connections among them are coloured red. The number of visible links for each hub gene is represented by the size of the node. Links and their corresponding hub genes are in the same colour.

Figure 5. A model of nacre formation in *P. f. martensii*.

In this model, new nacre (N) is formed in an organic matrix secreted by haemocytes or epithelial (Ep) cells beneath the mature nacre (M). Chitin provides the core of the polymer framework of the organic matrix. VWAP with chitin-binding domains binds to chitin and interacts with fibronectins and other VWAPs, forming the matrix networks. Asp-rich acid glycoproteins and acid GAGs function as the hydrogel substances. Tyrs catalyse the oxidation of tyrosine and dopamine and function in cross-linking and shell matrix maturation. Protease inhibitors, proteases and other enzymes regulate the biosynthesis or degradation of the organic matrix.

Additional file 1: methods and related tables

Additional file 2: Figure S1. Sequencing date and k-mer analysis.

a. The distribution of 17-mer depth derived from WGS sequence reads. X-axis is the K-mer depth and the Y-axis is the percentage of each K-mer depth. The first peak is created by sequence polymorphism and its relative height provides a measure of heterozygosity in the diploid genome. **b.** The heterozygous ratio of oyster genome estimated by k-mer analysis (left). The sequencing depth obtained by WGS reads mapped against assembly and GC content of our genome (right). **c.** The assembled length of the BACs of four pooling libraries. Four libraries were randomly selected and the total length of each assembly was calculated.

Additional file 3: Figure S2. Assembly coverage of BACs.

Sequencing depth on the BACs was calculated by mapped sequence reads. The annotated transposable elements (TEs) are shown in black or red, and the remaining unclosed gaps on the scaffolds are marked as white blocks.

Additional file 4: Figure S3. Codon usage and GC content analyses

a. Comparison of the distribution of codon usage among 9 species. ACAL, *Aplysia californica*; CGIG, *C. gigas*; CTEL, *C. teleta*; DRER, *D. rerio*; HROB, *H. robusta*; HSAP, *H. sapiens*; LGIG, *L. gigantea*; OBIM, *O. bimaculoides*; PMAR**,** *P. f.*

martensii. **b.** The GC content distribution for each codon position. **c.** The GC content distribution of exon, intron and inter-genetic regions.

Additional file 5: Figure S4. Phylogenetic analysis and gene clustering.

a. Species tree of *P. f. martensii* and 6 selected species. The number is the divergence time of the clades with ranges in parenthesis. **b.** Unique and shared gene families between *P. f. martensii* (*P. mar*) and other three species including *Crassostrea gigas* (*C. gig*), *Lottia gigantea* (*L. gig*) and *Homo sapiens* (*H. sap*).

Additional file 6: Figure S5. Phylogenetic analysis of TGF-β1/2/3 and bone morphogenetic proteins (BMP) from different species.

Proteins and accession numbers are listed in *SI Appendix*, Table S12.

Additional file 7: Figure S6. CHS and chitinase genes in *P. f. martensii*.

a. Chitin in the shell matrix of *P. f. martensii* and *C. gigas* stained green with Calcofluor White M2R. **b.** Expression of *CHS* in different organs. One *CHS* (Pma_10008435) is highly expressed in both the mantle pallium and the pearl sac. **c.** Expression of *chitinase* in mantle pallium (MP), mantle edge (ME) and in pearl sac (PS), compared with non-calcifying tissues (including A, adductor muscle; He, hepatopancreas; BC, hemocyte; Go, gonad; Gi, gill; F, foot). **d.** Expression of *Chitinases* and *CHS* at different developmental stages of *P. f. martensii*. Most of the *chitinases* are highly expressed at T and PV stages. The expression of one *CHS* (*Pma_10008435*), which is highly expressed both in mantle pallium and pearl sac, is also induced at T and PV stages. E, egg; Fe, fertilization; B, blastula; G, gastrula; ET, early trochophore; T, trochophore; D, D-shaped larvae; DF, D-larvae before feeding; EU, early umbo larvae; U, eye-larvae; PV, post-veliger; J, juveniles.

Additional file 9: Figure S7. RNAi analysis of four *VWAPs* in *P. f. martensii*.

Suppression of four *VWAPs* with RNAi. The expression profiles of four *VWAP* genes in the mantle, Pma_530.149, Pma_10019835, Pma_10019836 and Pma_1011175, were determined using real-time quantitative PCR, with GAPDH as the internal reference gene. *VWAPs* were significantly inhibited in the treatment group ($P < 0.05$). SEM images of the surface of the nacre from *P. f. martensii* injected with PBS and 100 μg RFP (red fluorescent protein) dsRNA demonstrated a normal growth status of nacre formation, whereas *P. f. martensii* in the treatment group injected with Pma_530.149, Pma_10019835, Pma_10019836 and Pma_10011175 dsRNA showed disruptions in crystal growth during nacre formation.

Additional file 10: Figure S8. Domain structure of collagens containing both

VWA and THR in *C. teleta* (Cte), *H. robusta* (Hro) and *M. coruscus* (Mco).

The accession number: a. ELU11155.1; b. ELT92434.1; c. WP_021368082.1; d. XP_009018142.1; e. XP_009024759.1; f. ALA16011.1.

Additional file 11: Figure S9. Tyrosinases and sulfotransferases in *P. f. martensii.*

a. Expression of *sulfotransferase* genes in early development. *CHST11* (Pma_133.4) and *D4ST1* (Pma_10006752) showed expression at the PV stage, whereas *CHST6* (Pma_279.110) and *CHST3* (Pma_10022575) were mostly up-regulated at the T stage. E, egg; Fe, fertilization; B, blastula; G, gastrula; ET, early trochophore; T, trochophore; D, D-shaped larvae; DF, D-larvae before feeding; EU, early umbo larvae; U, eye-larvae; PV, post-veliger; J, juveniles. **b, c.** *Tyr* expression in the mantle and pearl sac, respectively, compared with other non-calcifying tissues (including A, adductor muscle; He, hepatopancreas; BC, hemocyte; Go, gonad; Gi, gill; F, foot). *Tyrs* that were highly expressed in mantle pallium (MP) or mantle edge (ME) are shown in b, the different cycles represent different *Tyrs* (inside-out: Pma_10005159, Pma_10013533, Pma_10015392, Pma_10016044, Pma_10021421, Pma_10021422, Pma_10022578, Pma_10001525, Pma_10004452, Pma_10005803, Pma_10013532, Pma_10014430, Pma_10015306, Pma_10018719, Pma_10018775, Pma_10021425, Pma_10024726, Pma_10028201, Pma_10028307, Pma_10028311). Expression of *Tyrs* in pearl sac (PS) compared with other non-calcifying tissues are presented in c, with nine *Tyrs* highly expressed in PS marked with red frame. **d.** Abundance of quinoproteins (stained purple) in the nacre matrix revealed by a NBT/glycinate assay. Triangle represented prismatic layer and arrows represented nacreous layer.

Figure 1

Click here to download Figure Fig 1-06-01.tif

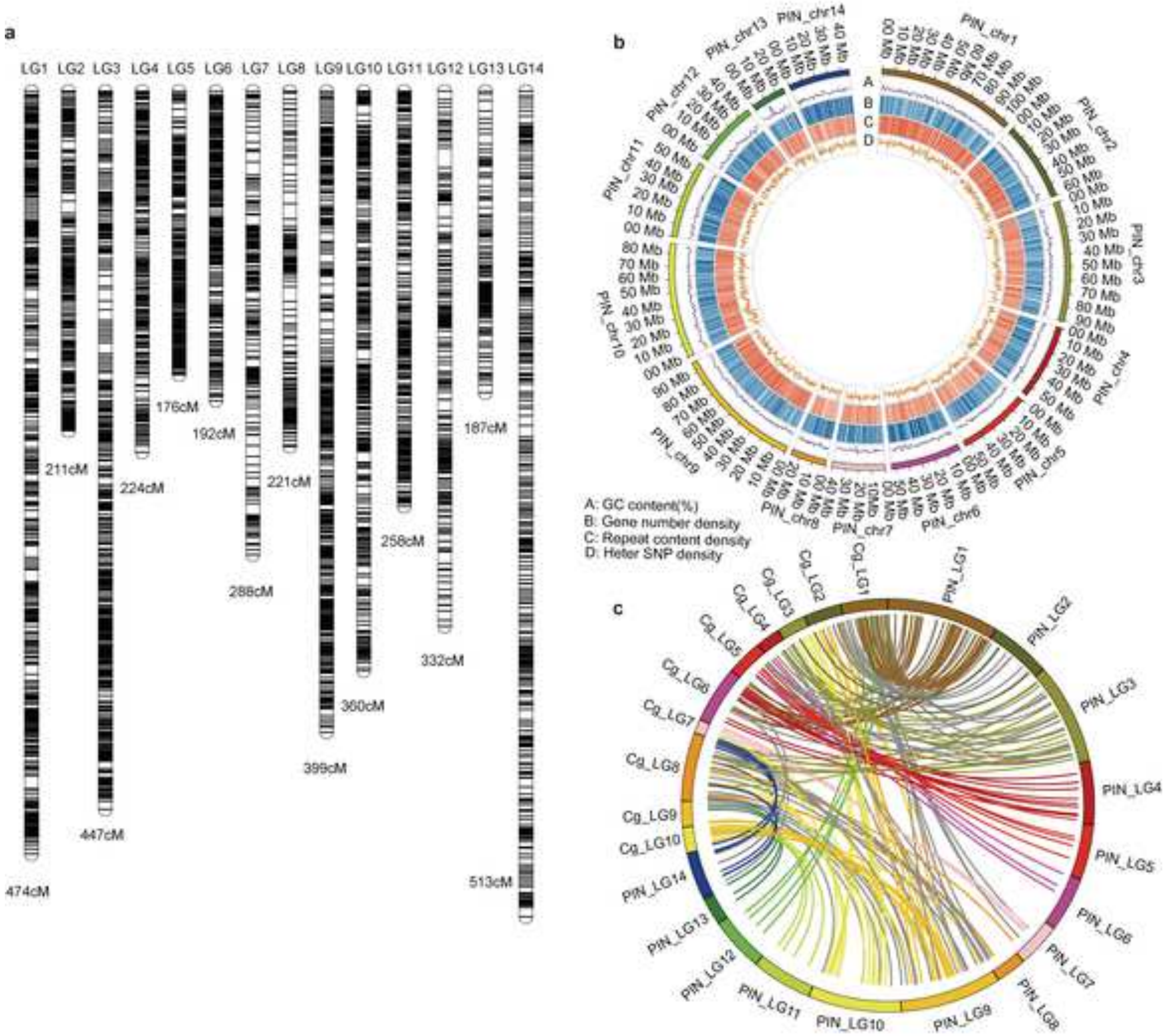

Figure 2

Click here to download Figure figure 2-4.pdf

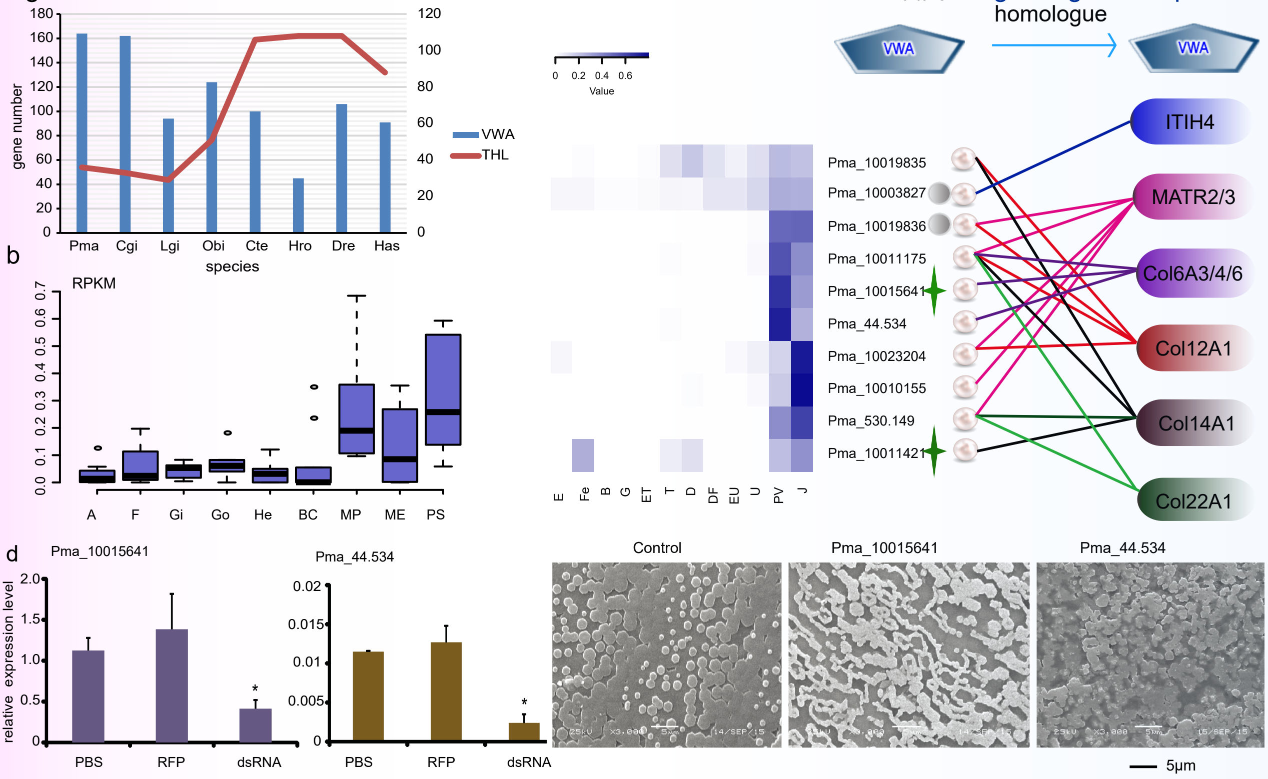

Figure 3

Click here to download Figure Fig.3-01.tif

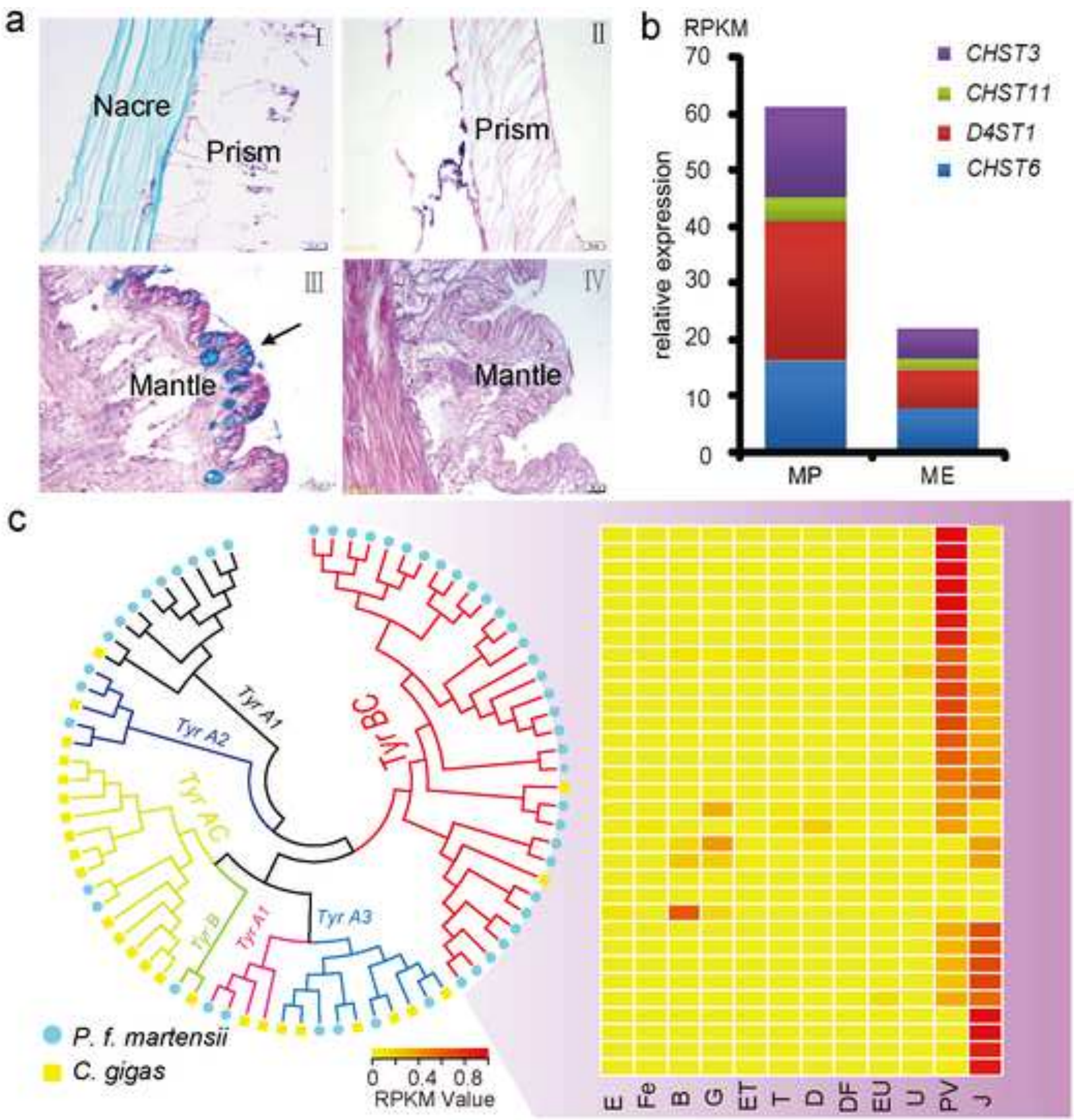

Figure 4

Click here to download Figure Fig.4-170106-01.tif

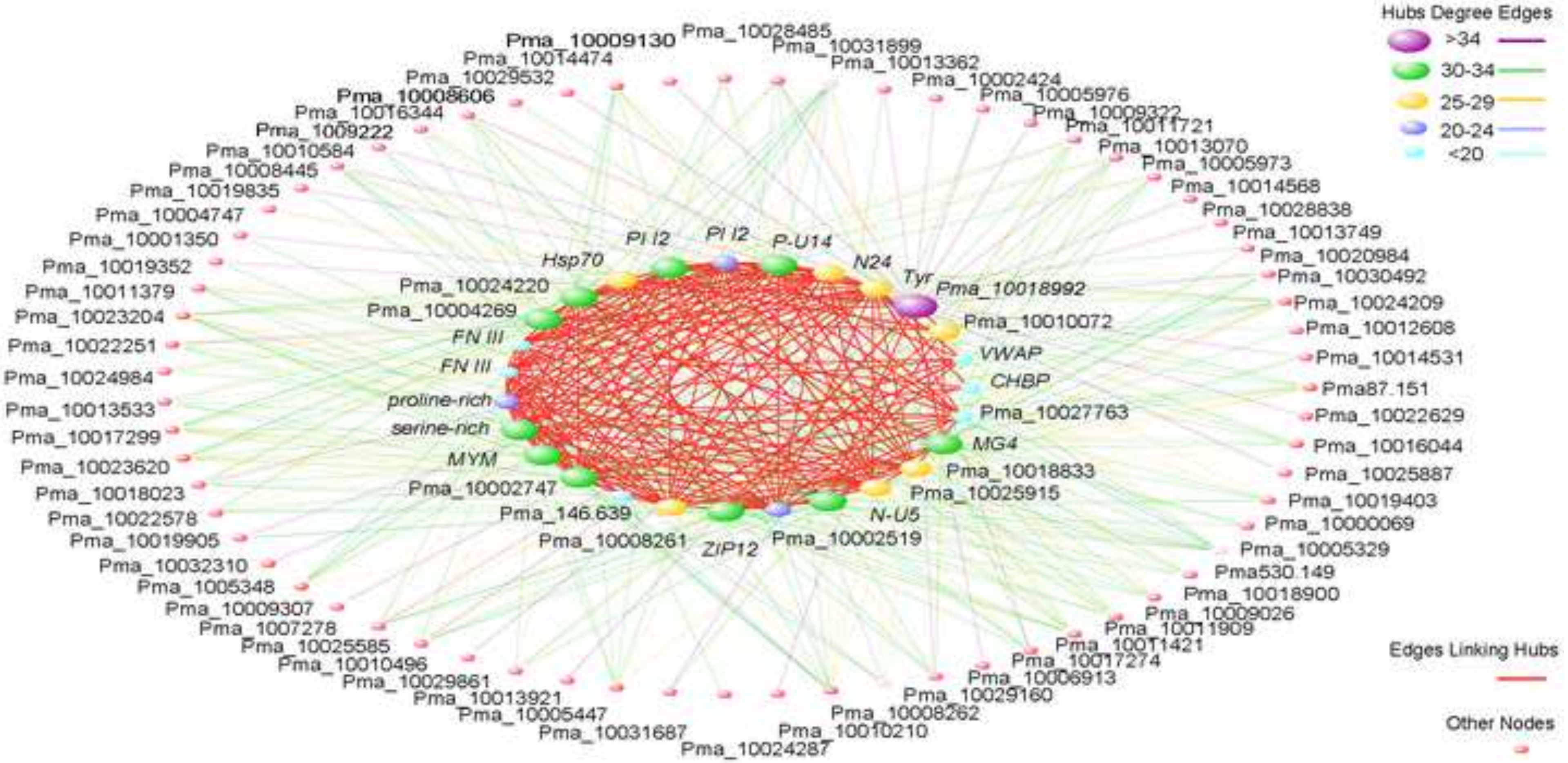

Figure 5

Click here to download Figure Fig.5.pdf

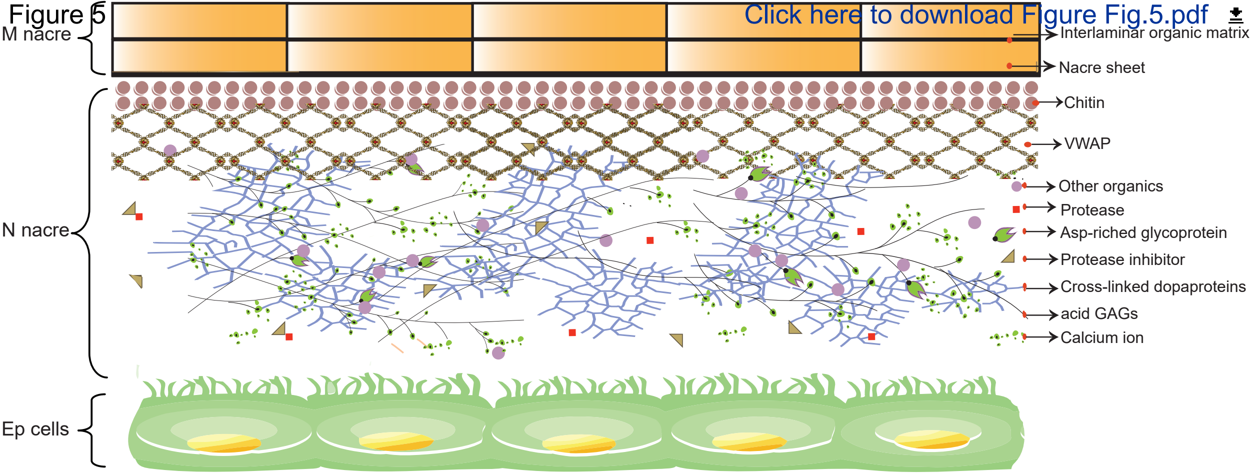

Figure 6

Click here to download Figure 7.jpg

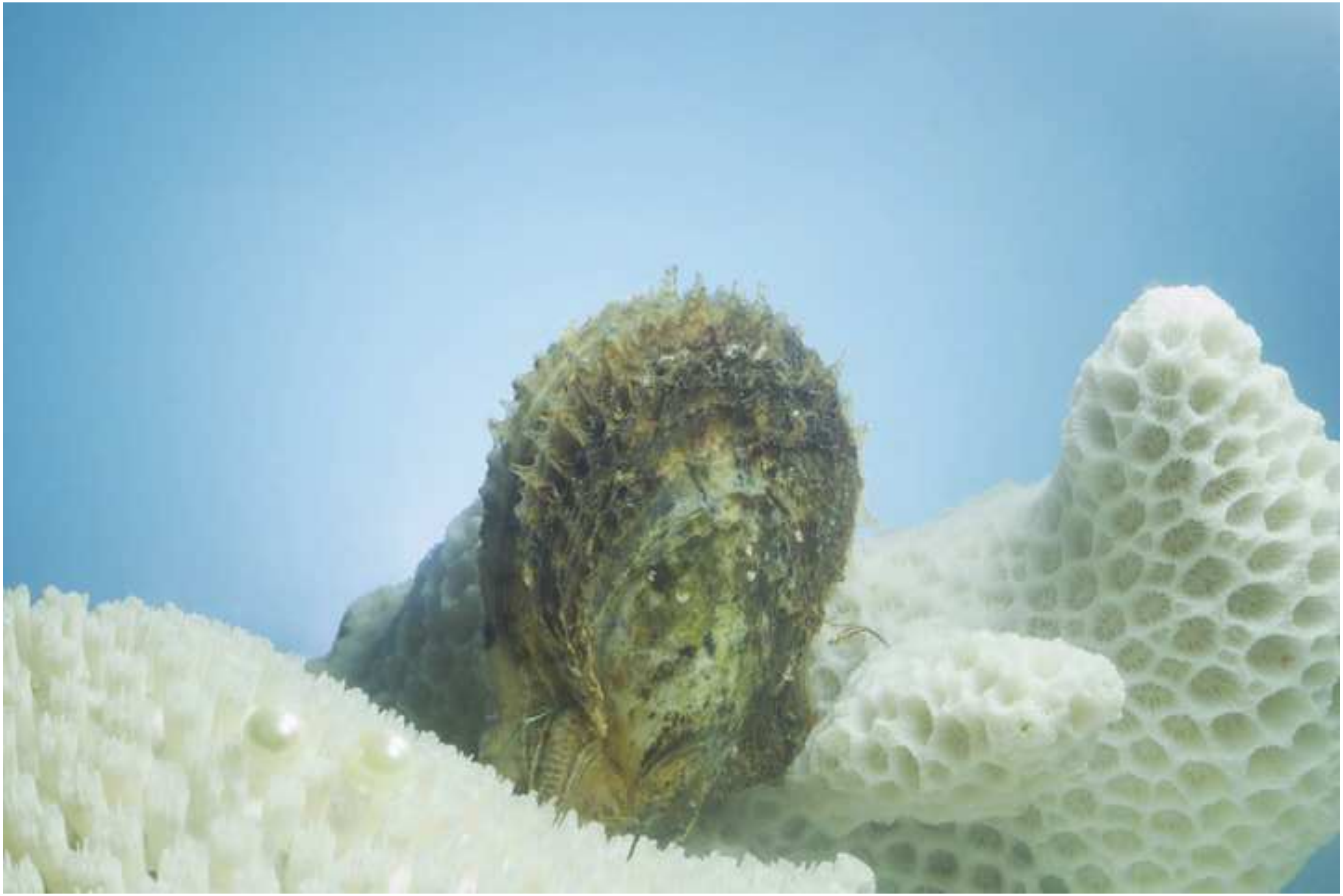

Additional file 1

Click here to access/download
Supplementary Material
Additional file 1-0405 XG-0523 (1).docx

Additional file 2.figure S1

Click here to access/download
Supplementary Material
Additional file 2.figure S1.tif

Additional file 3.figure S2

Click here to access/download
Supplementary Material
Additional file 3.figure S2.tif

Additional file 4.figure S3

Click here to access/download
Supplementary Material
Additional file 4.figure S3.tif

Additional file 5.figure S4

Click here to access/download
Supplementary Material
Additional file 5.figure S4.tif

Additional file 6.figure S5

Click here to access/download
Supplementary Material
Additional file 6.figure S5.tif

Additional file 7.figure S6

Click here to access/download
Supplementary Material
Additional file7.figure S6-070106.tif

Additional file 8: Datasets

Click here to access/download

Supplementary Material

Additional file 8-0405.xlsx

Additional file 9.figure S7

Click here to access/download
Supplementary Material
Additional file9. figure S7-01.tif

Additional file 10. figure S8

Click here to access/download
Supplementary Material
Additional file 10 Figure S8.pdf

Additional file 11. figure S9

Click here to access/download
Supplementary Material
Additional file 11.figure S9.tif

Dear Dr. Zauner,

Thank you for the comments and suggestions. We have carefully revised our manuscript accordingly. A point-to-point response is provided below. We hope the revised manuscript is now acceptable for publication in GigaScience. Please feel free to contact me if you have any questions. We look forward to hearing from you soon.

Sincerely yours,

Xiaodong Du

Point-to-point responses

- Please re-consider the number of joint first authors. We notice you have 11 joint first authors. It does not seem feasible that every single author has done exactly the same amount of work. You can provide information on everyones roles in the appropriate section at the end of the paper, so people are aware who did what. Please make sure you follow our criteria for authorship. This is based on definitions from International Committee of Medical Journal Editors and Committee on Publication Ethics, authorship is about responsibility and credit for making an intellectual contribution to the study. Acquisition of funding, collection of data, or general supervision of the research group, alone, does not justify authorship. I have pasted excerpts from our guidelines before.

Authors: Thank you for the comment. After discusions among our team, we decide to retain the following seven first authors: Xiaodong Du, Guangyi Fan, Yu Jiao, He Zhang, Ximing Guo, Ronglian Huang and Zhe Zheng. We feel contributions from these seven authors are equally important. The following is a brief summary of author contributions that is added to the revised ms:

X.D., X.G., G.Z. and X.L. designed scientific objectives. Y.J., R.H., W.C., X.X, Q.S. and G.F. managed the project. H.Z., F.S., X.L., C.S., W.L., H.L. and Z.W. performed genome assembly, gene annotation and evolution analyses. Y.D., Q.W. and X.Z. cultured *P. f. martensii* and provided materials. Y.D., Q.W., F.S., J.B. and Z.W. constructed the genetic map. Q.W., Z.Z., J.L. and R.H. performed the acid GAG analysis. Y.J., Z.Z., R.H. and J.L. performed chitin and VWAPs related analyses. R.H., C.B. and Z.Z. performed tyrosinase related analysis. Z.Z. and H.Z. performed the WGCNA analysis. X.D., G.F. and X.G. directed final data analyses. Y.J., X.G. and Z.X. directed critical revisions of intellectual content. X.D., X.G. and Y.J. supervised all aspects of the work to ensure the accuracy or integrity of the research and data. X.D., G.F., Y.J., H.Z., X.G., R.H., Z.Z. did most of the writing with contributions from all authors.

- We also note that you indicate four corresponding authors. OUP has a policy of only taking one - most responsive - author as corresponding author. Please note that, according to the guidelines, the definition of corresponding authorship is one of

responsiveness rather than seniority. The corresponding author is the one individual who takes primary responsibility for communication with the journal during the manuscript submission, peer review, and publication process, Again, please refer to the information below and on our homepage and please decide based on these guidelines who should be corresponding author.

Authors: Thank you for the suggestion. We see that the OUP's policy was adopted starting January 1, 2017. Our article was submitted in 2016. The four corresponding authors did a lot of work and contributed significantly to the correspondence related this manuscript. They represent four principle institutions that jointly led the project. We hope we could keep the four corresponding authors. Thank you for your understanding.

- Please add all relevant accession numbers to your manuscript (such as NCBI, PRIDE) and make sure the data are released and accessible. (If the PRIDE submission is a full submission, please cite the DOI. If it's a partial submission, add the accession.)

Authors: All relevant accession numbers were added to the revised paper.

- We recommend to mention the common name of the species in the title, in addition to the scientific name.

Authors: Thank you for the recommendation. The title was modified as "The pearl oyster *Pinctada fucata martensii* genome and multi-omic analyses provide insights into biomineralization".

- Please mention the GigaDB dataset in your data availability section, using the following format: "Data supporting the manuscript is also available via the GigaDB database [64]" and please add the citation of the GigaDB dataset to your reference list, using this format as template:

(this is just an example): Zheng L-Y, Guo X-S, He B, Sun L-J, Peng Y, Dong S-S, et al. Supporting data for "Title of your paper". GigaScience Database. 2017. http://dx.doi.org/xxxxxxxxx.

(If you do not have your final GigaDB DOI yet, just put XXX instead of the doi, and we'll add this for you)

Authors: Thanks. We have added the citation in reference list in this article.

- please remove highlighting/tracking of changes that was made for the purpose of review only.

Authors: Thanks. Highlighting/tracking of changes were removed.

- If you wish and it is available, please feel free to add a photograph of the oyster species as a Figure to the paper.

Authors: Thanks. We have prepared a picture of pearl oyster *P. f. martensii* as a Figure (figure 6) to the paper and submitted in the submission system.
