# Supplementary material for: The pearl oyster Pinctada fucata martensii genome and multi-omic analyses provide insights into biomineralization
Source: Gigascience. 2017 Jul 25;6(8):1–12. doi: 10.1093/gigascience/gix059 (PMC5597905; doi:10.1093/gigascience/gix059)
Supplement: Additional Files [file gix059_Supp.zip › Additional file 1-0405 XG-0523-0619XG-002xg.docx]

**Additional file 1:**

**The pearl oyster *Pinctada fucata martensii* genome and multi-omic analyses provide insights into biomineralization**

Xiaodong Du^a,*,#^, Guangyi Fan^b,g*^, Yu Jiao^a,*^, He Zhang^g,*^, Ximing Guo^c,*,#^, Ronglian Huang^a,*^, Zhe Zheng^a,*^, Chao Bian^g^, Yuewen Deng^a^, Qingheng Wang^a^, Zhongduo Wang^a^, Xinming Liang^g^, Haiying Liang^a^, Chengcheng Shi^g^, Xiaoxia Zhao^a^, Fengming Sun^g^, Ruijuan Hao^a^, Jie Bai^g^, Jialiang Liu^a^, Wenbin Chen^g^, Jinlian Liang^a^, Weiqing Liu^g^, Zhe Xu^e^, Qiong Shi^g^, Xun Xu^g^, Guofan Zhang^d,f,#^, Xin Liu^g,#^

*These authors contributed equally.

#Corresponding authors: X.D. ([zjduxd@126.com](mailto:zjduxd@126.com)), X.G. ([xguo@hsrl.rutgers.edu](mailto:xguo@hsrl.rutgers.edu)), G.Z. (gzhang@qdio.ac.cn) and X.L. ([liuxin@genomics.cn](mailto:liuxin@genomics.cn)).

^a^Fishery College, Guangdong Ocean University, Zhanjiang, 524025, China;

^b^BGI-Qingdao, Qingdao 266555, China

^c^Haskin Shellfish Research Laboratory, Department of Marine and Coastal Sciences, Rutgers University, Port Norris, New Jersey 08349, USA;

^d^Key Laboratory of Experimental Marine Biology, Institute of Oceanology, Chinese Academy of Sciences, Qingdao, China;

^e^Atlantic Cape Community College, Mays Landing, New Jersey 08330, USA;

^f^Laboratory for Marine Biology and Biotechnology, Qingdao National Laboratory for Marine Science and Technology, Qingdao, China

^g^BGI-Shenzhen, Shenzhen, 518083 China;

**Keywords:** genome, biomineralization, nacre, VWA-containing protein, *Pinctada fucata martensii*

**Contents**

[1 Organism background and sequencing 3](#_Toc479682794)

[2 Genome characterization by K-mer analysis 4](#_Toc479682795)

[3 BAC clones and sequencing 4](#_Toc479682796)

[4 Quality validation of assembly 5](#_Toc479682797)

[5 Linkage map construction 5](#_Toc479682798)

[6 Repeat content analysis 6](#_Toc479682799)

[7 Gene model prediction 7](#_Toc479682800)

[8 Gene family clustering 8](#_Toc479682801)

[9 Phylogenetic tree construction and divergence time estimation 8](#_Toc479682802)

[10 Gene family expansion and contraction 9](#_Toc479682803)

[11 Sample preparation for developmental transcriptomes 9](#_Toc479682804)

[12 Transcriptome analysis 10](#_Toc479682805)

[13 Methods for paraffin section 12](#_Toc479682806)

[13.1 Sample preparation 12](#_Toc479682807)

[13.1.1 Decalcification of shells and pearls 12](#_Toc479682808)

[13.1.2 Preparation of tissue samples 12](#_Toc479682809)

[13.2 Specimen embedding and sectioning 12](#_Toc479682810)

[14 Nacreous and prismatic layer protein 12](#_Toc479682811)

[15 Identification of glycosaminoglycans (GAGs) in shells 14](#_Toc479682812)

[16 Nitrobluetetrazolium (NBT)/glycinate assay for dopa and dopaquione proteins 15](#_Toc479682813)

[17 RNAi experiment 15](#_Toc479682814)

[18 Co-expression network analysis 15](#_Toc479682815)

[18.1 Co-expression pattern analysis for reconstruction of co-expression network for bio-mineralization 16](#_Toc479682816)

[18.2 Pathway enrichment of genes co-expressed with nacre genes 16](#_Toc479682817)

[References 17](#_Toc479682818)

[SI Tables 19](#_Toc479682819)

# **1 Organism background and sequencing**

Although a draft pearl oyster genome assembly based on Roche 454 GS-FLX and Illumina GAIIx reads was published in 2011 [1], its scaffold N50 (~14.5Kb) and contig N50 (~1.6Kb) are too short for in-depth analysis. The problem is likely caused by high polymorphism and repetitive content. To overcome this problem, we used a pearl oyster (*Pinctada fucata martensii*) from a line selected for fast growth for 3 generations and a BAC-to-BAC strategy to obtain a greatly improved assembly.

In addition to the production and sequencing of BAC clones, we also constructed and sequenced whole genome shotgun (WGS) libraries with 3 short insert-sizes and 4 long insert-sizes. We isolated, fragmented, end-repaired and A-tailed the genomic DNA and then ligated it with Illumina paired-end adapters, according to protocols from Illumina. For the short insert-size (including 170bp, 500bp and 800bp) libraries, size selection was performed on agarose gels and amplified by LM-PCR. For long insert-size (including 2kb, 5kb, 10kb and 20kb) mate-pair libraries, we used nebulization to shear the DNA to the target sizes and biotinylated nucleotide analogues for end-repair, and circularized DNA by intra-molecular ligation. For circular DNA molecules, we used Adaptive Focused Acoustics (Covaris) to shear to an average size of 500 bp. Biotinylated fragments were purified on magnetic beads (Invitrogen), end-repaired, A-tailed and ligated to Illumina paired-end adapters, size-selected again and purified by LM-PCR. Finally, we sequenced all libraries on the Illumina Hiseq 2000 sequencing system.

To ensure high quality of sequencing reads for assembly, we filtered all raw WGS reads for sequencing errors with a series of strict criteria by removing: 1) reads contained more than 2% Ns or with polyA structure; 2) reads contained 20% or more low quality bases; 3) reads polluted by adapters; 4) reads with overlap between read1 and read2; and 5) reads with duplication (one read pair is identical to the other). For criteria 4, except for the 170bp library with read length of 100bp, reads were filtered out when there was a minimum of 10bp overlap between read1 and read2, and less than 10% mismatches in overlapping region.

# 2 Genome characterization by K-mer analysis

We used Kmerfreq (our inner program) for the K-mer analysis to estimate genome size and heterozygosity. K-mer is a sequence with K bp (K was a positive integer) such as a 17 bp sequence being called a 17-mer. K-mers can be used to correct sequencing errors, construct contigs and estimate genome size, heterozygosity and repeat content [2].

# 3 BAC clones and sequencing

To overcome assembly difficulties associated with highly polymorphic and complex genomes, we used the BAC-to-BAC assembly strategy. We constructed 46,080 BAC clones of 5-fold genome size. We used the Agilent Bravo Automated Liquid Handling Platform and Agilent BenchCel Microplate Handler to construct the BAC libraries. First, we used the Adaptive Focused Acoustics DNA fragmentation system to shear DNA samples and used the automated batch processing capability and 96-microTUBE plates as sample vessels. Secondly, we used T4 DNA polymerase and *E. coli* DNA polymerase I Klenow fragment to convert overhangs and used the polymerase activity of Klenow fragment to ligate index adapters and added an ‘A’ bases to the 3’ ends of blunt DNA fragments. Thirdly, based on the sample’s position on the plate, we pooled DNA samples with different index adapters and then, we removed unligated index adapters and selected DNA segments within the target size range. Index primers were ligated to DNA segments, and PCR was used to enrich the DNA fragments containing index adapters and index primers on both ends, and to amplify the DNA. Finally, we used gel electrophoresis to remove unligated index primers and to select DNA segments again, and we performed quality control tests using Agilent 2100 Bioanalyzer and StepOnePlus Real-Time PCR System.

After the construction of all BAC clones, we constructed one 500 bp library and sequenced to 100-fold depth using Hiseq 2000 at read length of 100 bp for each BAC clone. After filtering with criteria described above, about 546.8Gb high quality reads were obtained for further assembly.

# 4 Quality validation of assembly

We used three datasets to assess assembly integrity: 1) We compared our assembly with the draft assembly of a previous study [1] using LASTZ [3], with parameters of “T=2 C=2 H=2000 Y=3400 L=6000 K=2200 --format=axt”, and the alignments were linked by chains and nets; 2) We aligned four Sanger-sequenced BACs against our assembly to find candidate scaffolds using nucmer, delta-filter, and show-coords of MUMmer [4] with default parameters and then locally compared the BACs and candidate scaffolds using BLASTN; and 3) We evaluated the gene regions of the assembly by mapping transcripts derived from RNA-seq using BLAT [5], with default parameters and an identity cut-off of 90%.

# 5 Linkage map construction

To anchor our scaffolds to chromosomes, we constructed a genetic map for *P. f. martensii* using restriction site associated DNA sequencing (RAD-seq) [6]. After filtering, we used SOAP2 [7] to map the raw reads to the reference genome of *P. f. martensii* (scaffolds). Parameters for SOAP2 were set to “-m 0, -x 1000, -s 35, -l 32, -v and –p 4”. Based on alignment results of SOAP2, we performed single nucleotide polymorphism (SNP) calling using SOAPsnp [8], with parameters set to “-L100, -u, -F 1”. After SNP calling, we combined all SNPs from all 150 individuals and extracted genotypes according to the SNP results. Finally, 81,039 SNP makers were obtained.

To reduce complexity for linkage analysis, only top three makers from each scaffold were selected for analysis based on maker quality. First, the missing rate of a maker should be lower than 30%. Then, Chi-square test was used to detect segregation distortion before the top three markers were chosen.

We constructed linkage map using JoinMap 4.1 [9]. First, linkage group clustering was performed with the grouping method of JoinMap using the CP (cross pollen) model. Fourteen linkage groups were obtained, which is consistent with the haploid chromosomal number of *P. f. martensii* (2N=28). The maximum likelihood mapping algorithm was then selected to calculate the linkage distance within each group. Because the default parameters for calculating linkage distance were optimized for fewer than 100 markers, we optimized parameters for our data. The parameters for distance calculation were set to “chain length=1000, initial acceptance probability=0.250, cooling control parameter=0.00100, stop after # chains without improvement=10000, length of burn-in chain=50000, Nr. of Monte Carlo EM cycle=12, chain length per Monte Carlo EM cycle=10000 and sampling period for recombinant frequency matrix sample=5”. Finally, approximately 86.5% of the assembled genome length was anchored to the linkage map.

# 6 Repeat content analysis

Two complementary methods, homology-based search and *de novo* prediction, were used to identify repeat elements. We obtained known and classified repeat elements from Repbase [10], and then used those sequences as library for RepeatMasker [11]. All potential repeat elements were obtained based on similarity with known elements databases. For *de novo* prediction, repeat sequence library was obtained by combining results from two software LTR-finder [12] and RepeatModeler [13]. Then we used RepeatModeler again to classify the library based on Repbase taxonomy system. The *de novo* library was used to prediction repeat elements by using RepeatMasker. We used TRF with default parameters to predict tandem repeats.

# 7 Gene model prediction

Three methods were used to predict gene models based on homology, *ab initio* prediction and transcriptome evidence. All predicted gene models were integrated with GLEAN [14] to obtain a consensus gene set.

1) Protein sequences were collected from public databases for six well-characterized animal genomes: *Anopheles gambiae*, *Caenorhabditis elegans*, *Capitella teleta*, *Drosophila melanogaster*, *Lottia gigantea* and *Crassostrea gigas*. BLAST was used to query those protein sequences in *P. f. martensii* genome sequences to identify potential gene locations (E-value<=1e-5). We extended 1000 base pairs for two ends of potential gene locations, and then applied GeneWise [15] to predict the exact gene structures (Default parameters).

2) Augustus [16] and GENESCAN [17] were used for *de novo* gene prediction using TE-masked genome sequences. Hidden markov models (HMM) parameters were trained by 800 high-score gene models of GeneWise to improve prediction results.

3) We randomly selected 15 samples from our transcriptome datasets and used their cDNA sequences as transcription evidence. We used TopHat 2 [18] to map the raw data to the reference assembly, and obtained the junction alignments. We then used the Cufflinks [19] to predict transcripts based on TopHat2 alignments.

4) We combined and used all predictions as input evidences for GLEAN, and obtained 32,937 high-confidence gene models. To evaluate the final gene set, we divided all gene models into three classes based on types of supporting evidences: homology, *de novo* or RNA transcripts.

For annotation, we queried all *P. f. martensii* proteins against four functional databases, Nr, Swissport [20], TrEMBL [20] and KEGG [21], using BLAST (E-value<=1-e5), and accepted results with the best scores for each query protein. We also used InterProScan [22, 23] to predict gene function based on domain information.

# 8 Gene family clustering

To identify common and specific gene families, we analyzed gene sets from the following species: *C. teleta*, *D. rerio*, *H. robusta*, *H. sapiens*, *L. gigantea*, *C. gigas* and *P. f. martensii*. Based on Treefam [24], we used the following steps to obtain gene families:

(1) Pair-wise alignment: First, we combined all proteins together and did all-to-all alignment using BLASTP (E-value<=1e-7), and then collected all matches into one group for each query sequence using Solar (inner) (-a prot2prot). If the identity between two genes is more than 30%, the two genes are considered homologous. We also used another criterion, the H-score, to evaluate the similarity. For example, gene A and gene B have three types of BLAST scores SCORE_A-B, SCORE_A-A and SCORE_B-B, and the H-score for A-B is SCORE_A-B divided by the maximum value of SCORE_A-A and SCORE_B-B.

(2) Hierarchical clustering: We clustered all genes into families by using average distances for hierarchical clustering algorithm based on all-versus-all H-scores. Two kinds of criteria were taken. First, the H-score must be more than 10. Secondly, the value that total pairs of one group divided by theoretical maximum pairs between all genes of the same group, should be more than 1/3.

# 9 Phylogenetic tree construction and divergence time estimation

We used MrBayes [25] for phylogenetic analysis of seven species: *C. teleta*, *D. rerio*, *H. robusta*, *H. sapiens*, *L. gigantea*, *C. gigas* and *P. f. martensii*. We identified single-copy genes that have only one gene per family in each species based on gene family results from Treefam. We performed multiple protein sequence alignment for 114 single copy families using MUSCLE [26], and then transformed the protein sequences to nucleotide coding sequences (CDS). Based on CDS alignments, a total of 81,840 nucleic acid sites were used for tree construction by extracting and concatenating phase one sites for all families.

The mcmc tree program within PAML package [27] was used to estimate divergence time for the species tree. To estimate the time accurately, the divergence time between *D. rerio* and *H. sapiens* from fossil records [28] was used as a reference.

# 10 Gene family expansion and contraction

To explore the evolutionary history of *P. f. martensii* gene families, we investigated the expansion or contraction of gene families. We classified all proteins into different families according to KEGG functional annotation. For comparison between *P. f. martensii* and *C. gigas*, we used Fisher’s exact test to detect significant differences for each gene family. For comparing seven species in phylogenetic tree, we used CAFÉ [29] to infer expansion or contraction against the latest common ancestor for each two branches.

# 11 Sample preparation for developmental transcriptomes

To assist studies on biomineralization, we sequenced transcriptomes of 12 developmental samples. Mature *P. f. martensii* were sampled from our selectively bred population and induced to spawn by flowing water after brief drying. Fertilized eggs were incubated in 100-L buckets and then transferred to 1000-L tanks. Larval rearing was the same as previously described by Deng et al (32). Culture density was kept at 1 embryo or larva per ml. Daily feeding consisted of *Isochrysis galbana* from Day 2 to 5, and a mixture of *I. galbana* and *I. zhanjiangensis* from Day 6 to 50. Every other day 300-L filtrated seawater was replaced in each tank. Water temperature was at 25±1^o^C and salinity was 30‰. At approximately day 50, juveniles with a size of 2-3 mm were removed from the plastic film and put into 45 × 45cm pearl oyster nets at a density of 400 individuals per net. The pearl oysters were reared in Chengwu, Xuwen of Zhanjiang. They were cleaned and placed in new nets with dead individuals removed at regular intervals.

Developmental samples included unfertilized eggs and 11 samples collected at 30 min, 5 h, 6 h, 8 h, 16 h, 19 h, 4 d, 14 d, 28 d, 40 d and 90 d after fertilization. Samples used for scanning electron microscopy were produced from the same stock. Larvae were cultured at the same temperature and salinity as mentioned above. Eggs, embryos and larvae were fixed with 5% glutaraldehyde in PBS for 3 h at room temperature. The fixed samples were rinsed twice in 0.1 M cacodylate buffer adjusted to pH 7.2 and 1,000 mOsM, and then dehydrated through a graded acetone series, before drying by carbon dioxide at critical point. Then samples were sputter-coated with gold and observed at 20/25 kV with a KYKY-2800B scanning electron microscope.

# 12 Transcriptome analysis

To characterize spatial gene expression profile, we sequenced transcriptomes of 9 organs or tissues (pooled from five adults): adductor muscle (A), mantle pallium (MP), mantle edge (ME), hepatopancreas (He), hemocyte (BC), gonad (Go), gill (Gi), foot (F), along with pearl sac (PS) at 180 day after nucleus transplantation. Two mantle tissue samples that were pooled from five adults within fast (DYW-L) and slow (DYW-S) growing pearl oysters were also sequenced.

We extracted total RNA from each sample and enriched mRNA using oligo (dT) magnetic beads. The mRNA was fragmented into short fragments (200~500 bp). We synthesized the first-strand cDNA with random hexamer-primers using mRNA fragments as templates. Then, dNTPs, RNase H, DNA polymerase I and buffer were added for the second strand synthesis. Double strand cDNA was purified and end-repaired with addition of A using the QIAquick PCR extraction kit. Sequencing adapters were ligated to the fragments. The fragments were purified by agarose gel electrophoresis and enriched by PCR amplification. Paired-end libraries with insert-size of 500 bp were generated for each sample and sequenced separately using Illumina Hiseq 2000.

Raw reads, with read length of 100bp, were filtered using SOAP*filter* (V2.2) to remove those containing the sequence of adapters, high number of “N” and having low sequence quality scores, before further analysis. Filtering was performed in the following steps: 1) filtering reads with adapters; 2) removing reads with more than 10% Ns; and 3) removing reads containing >50% low quality bases whose sequencing quality scores are less than 10. After filtering, the remaining reads were considered as high-quality "clean reads".

All clean reads were mapped to the assembly sequences with SOAP2 allowing less than 5 mismatches.

We used the *RPKM* method [30] (Reads per kilobase transcriptome per million mapped reads) to calculate the gene expression level as defined by the formula:.


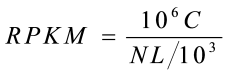


Given that: gene is *A*, *C* is the number of reads which are uniquely mapped to gene A , N is total number of reads which are uniquely mapped to all genes, and L is number of bases located on gene A.

We performed functional enrichment analysis of our target genes (TGs) with the commonly used Gene Ontology (GO), KEGG and IPR databases. GO provides three ontologies: molecular function, cellular component and biological process. By comparing with the background of all genes, enrichment analysis provides all terms (GO term, pathway ID and IPR ID) that are significantly enriched in the TGs. We developed a strict algorithm for the analysis, with p-value defined as:


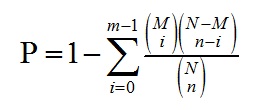


Where N is the count of all genes with functional annotation; n is the count of TGs in N; M is the count of all genes that are annotated to certain functional terms; and m represents the count of TGs in M. The calculated p-value was subjected to Bonferroni Correction, taking corrected p-value ≤ 0.05 as the threshold. Functional terms fulfilling this condition were defined as significantly enriched functional terms in TGs.

# 13 Methods for paraffin section

## 13.1 Sample preparation

### 13.1.1 Decalcification of shells and pearls

Shell samples from *P. f. martensii* and *C. gigas* were washed with distilled water to remove fouling matters. Samples for chitin detection were soaked in 1M acetic acid at 4 ^o^C for 1 week. Samples for Alcian blue-periodic acid schiff (AB-PAS) staining for glycosaminoglycan were immersed in 10% EDTA-2NA solution at room temperature for 10 days. The decalcified shells became soft, and they were cleaned with distilled water 5 times and prepared for paraffin embedding.

### 13.1.2 Preparation of tissue samples

Whole mantles separated from *P. f. martensii* and *C. gigas* were trimmed into 2-4 mm thick slices with at least one flat surface and fixed in 4% paraformaldehyde at 4^o^C overnight.

## 13.2 Specimen embedding and sectioning

After washing off fixative with PBS, samples were dehydrated with a series of ethanol gradient and washed in xylene [31]. And then, the specimens were embedded in four consecutive baths of filtered paraffin at 56 ^o^C for 1 to 2 h each. Embedded specimens were solidified with cold water. Sectioning was carried out on LEICA RM2235. The prepared sections were about 6 mm thickness. Before use, samples of mantle tissue were deparaffinized and re-hydrated:

# 14 Nacreous and prismatic layer proteins

To understand the composition and function of the organic matrix in biomineralization, we conducted proteomic studies to identify proteins, both soluble and insoluble, present in the nacre and prismatic layers of *P. f. martensii* shells.

1. Isolation and sequencing of nacreous layer and prismatic layer proteins

Shells were brushed and washed with water to remove fouling matters and placed in sodium hypochlorite solution for 24 h to bleach the shells and remove organic materials and possible microbe contamination on the surface. The prismatic layer was separated from the edge of pearl oyster shells without nacre. The nacre was directly scraped from the internal surface of shells with abundant aragonite. These samples were thoroughly grinded for the extraction of shell proteins. The acetic acid solution (5%, v/v) was slowly added to dissolve the calcium carbonate (for at least 12 h). Finally, the samples were centrifuged at 14000g and 4 ^o^C for 1 h. The supernatant contained the acid-soluble proteins and the residue contained the acid-insoluble proteins.

1. Protein separation by 1D SDS-PAGE and in-gel digestion.

Samples were electrophoresed in 12% polyacrylamide gel. The gel was stained with Coomassie blue R-250. Ten bands were excised and destained using 50 mM ammonium bicarbonate in 50% ACN (acetonitrile). Gel pieces were incubated with 10 mM DTT in 25 mM ammonium bicarbonate for 1 h at 60 °C to reduce disulfide bonds. Alkylation of cysteines was performed by incubating the samples with 55 mM iodoacetamide in 25 mM ammonium bicarbonate for 45 min at room temperature in dark. Then, Trypsin Gold (Promega, Madison, WI, USA) digestion was carried out at 37 °C for 16 h. After digestion, the peptides were extracted sequentially from gel bands using 0.1% formic acid in 50% ACN twice and 100% ACN twice. The extracted peptides were dried and stored at -80 °C until LC-MS/MS analysis.

1. LC-MS/MS analysis.

Each fraction was resuspended in buffer A (2% acetonitrile, 0.1% formic acid) and centrifuged at 20000g for 10 min. The final concentration of peptides was about 0.5 µg/µl on average. 10 µl supernatant was loaded on a LC-20AD nanoHPLC (Shimadzu, Kyoto, Japan) by the autosampler onto a 2 cm C18 trap column. Then, the peptides were eluted onto a 10 cm analytical C18 column (inner diameter 75 µm) packed in-house. The samples were loaded at 8 µL/min for 4 min, then the 44 min gradient was run at 300 nL/min starting from 2 to 35% B (98%ACN, 0.1%FA), followed by 2 min linear gradient to 80%, and maintenance at 80% B for 4 min, and finally return to 5% in 1 min. The peptides were subjected to nanoelectrospray ionization followed by tandem mass spectrometry (MS/MS) in a LTQ OrbitrapVelos (Thermo) coupled online to the HPLC. Intact peptides were detected in the Orbitrap at a resolution of 60000. Peptides were selected for MS/MS using the collision induced dissociation (CID) operating mode with a normalized collision energy setting of 35%. Ion fragments were detected in the LTQ. A data-dependent procedure that alternated between one MS scan followed by ten MS/MS scans was applied for the ten most abundant precursor ions above a threshold ion count of 5000 in the MS survey scan with the following Dynamic Exclusion settings: repeat counts, 2; repeat duration, 30 s; and exclusion duration, 120 s. The applied electrospray voltage was 1.5 kV. Automatic gain control (AGC) was used to prevent overfilling of the ion trap; 1×104 ions were accumulated in the ion trap to generate CID spectra. For MS scans, the m/z scan range was 350 to 2,000 Da.

# 15 Identification of glycosaminoglycans (GAGs) in shell

In mollusks, GAGs have been considered as one of the major components of the organic matrix due to the acidic nature of their functional groups including sulfate and carboxyl [32]. To observe the distribution of GAGs, pearl oyster shells were decalcified in 1M acetic acid at 4 ^o^C for 1 week (10% EDTA-2Na solution at room temperature for 10 days), before collecting the decalcified material. This insoluble material was washed with distilled water and post-fixed with 4% paraformaldehyde overnight. Then the fixed material was embedded in parafﬁn and stained with Alcian blue/periodic acid–Schiff (AB/PAS). Parafﬁned sections were first stained with Alcian blue for 5 min, periodic acid for 2 min (periodic acid must be freshly made) and Schiff for 10 min [33]. Stained sections were dehydrated in a gradient of ethanol solutions (50%, 70%, 85%, 95%, 100%), rinsed in xylene, and observed under a BX51 optical microscope.

# 16 Nitrobluetetrazolium (NBT)/glycinate assay for dopa and dopaquione proteins

Shells were decalcified, and the decalcified material was embedded and sectioned as described above. One ml nictrobluetetrazolium (0.24 Mm NBT) in 2 M potassium glycinate (pH 10) was used to stain the sections for nearly 5 min until the appearance of violet positive signals [34]. The staining was then stopped with double distilled water, and the stained material was mounted.

# 17 RNAi experiment

Sequence-specific primers were designed from cDNA sequences of six *VWA containing proteins* (*VWAPs*). The primers used for generating the *VWAPs* double-strand RNA (dsRNA) are shown in *SI Appendix*, Table S17. DsRNAs were synthesized following the method of Suzuki et al [35] and injected into the adductor muscle of *P. f. martensii* every 4 days at 100 μg per 100 μL per pearl oyster each time. *P. f. martensii* injected with 100μg red fluorescent protein (RFP) dsRNA was used as the negative control, with PBS as the blank control. Each group contained at least eight individuals. Eight days after the first injection, mRNAs of the mantle pallium of *P. f. martensii* in each group were extracted using TRIzol for first-strand cDNA synthesis. Glyceraldehyde 3-phosphate dehydrogenase (GAPDH) was used as the internal reference gene for calculating gene expression. The effects of RNAi of the six *VWAPs* on nacre formation were detected by SEM.

# 18 Co-expression network analysis

In previous studies, some proteins isolated from the mantle or nacre have been shown to direct and fine-tune crystallization or framework assembling [35, 36]. We also provided some evidence of several factors involved in nacre formation. In fact, diverse proteins have been found in the shell, highlighting the complexity of shell formation [37]. To identify the gene network for nacre formation, we conducted weighted gene co-expression network analysis (WGCNA) of transcriptomes from four mantle samples including mantle edge, mantle pallial and two entire mantles representing fast and slow growing pearl oysters. Further, we identified genes interrelated with the hub genes, and revealed potential regulatory mechanisms of nacre formation by KEGG enrichment analysis.

## 18.1 Co-expression pattern analysis for reconstruction of co-expression network for bio-mineralization

Networks can provide a straight-forward representation of interactions among nodes (genes). We used WGCNA to reconstruct co-expression network for biomineralization [38]. To identify the hub genes among the 234 biomineralization protein genes, we extracted and exported the weighted network of these genes, restricted the genes through calculating the connectivity for each node, selected the top 30 highly connected nodes and applied a weight cut-off of 0.55. Finally, the top 27 nodes (hub genes) were retained. We used VisANT to visualize the connections among these hub genes and other genes related to nacre formation.

## 18.2 Pathway enrichment of genes co-expressed with nacre genes

Genes co-expressed with nacre genes were extracted from the network construction results with a weight cut-off of 0.5, and 3245 genes were retained for KEGG pathway enrichment analysis. Significantly enriched pathways in target genes were identified using the whole genome as a background, and a hyper-geometric test was introduced to calculate the significance as described above. Pathways with significance values greater than 95% (p-value<0.05) were defined as significantly enriched in the target genes.

# References

1. Takeuchi T, Kawashima T, Koyanagi R, Gyoja F, Tanaka M, Ikuta T, et al. Draft genome of the pearl oyster *Pinctada fucata*: a platform for understanding bivalve biology. DNA Res. 2012;19:117-30.

2. Li R, Fan W, Tian G, Zhu H, He L, Cai J, et al. The sequence and de novo assembly of the giant panda genome. Nature. 2010;463:311-17.

3. Harris RS: Improved pairwise alignment of genomic DNA*.* ProQuest. 2007.

4. Kurtz S, Phillippy A, Delcher AL, Smoot M, Shumway M, Antonescu C, et al. Versatile and open software for comparing large genomes. Genome Biol. 2004;5:1.

5. Kent WJ. BLAT—the BLAST-like alignment tool. Genome Res. 2002;12:656-64.

6. Baird NA, Etter PD, Atwood TS, Currey MC, Shiver AL, Lewis ZA, et al. Rapid SNP discovery and genetic mapping using sequenced RAD markers. Plos One. 2008;3:e3376.

7. Hecker A, Mikulski Z, Lips KS, Pfeil U, Zakrzewicz A, Wilker S, et al. Pivotal Advance: Up-regulation of acetylcholine synthesis and paracrine cholinergic signaling in intravascular transplant leukocytes during rejection of rat renal allografts. J Leukocyte Biol. 2009;86:13-22.

8. Li R, Li Y, Fang X, Yang H, Wang J, Kristiansen K, et al. SNP detection for massively parallel whole-genome resequencing. Genome Res. 2009;19:1124-32.

9. Van Ooijen J. Multipoint maximum likelihood mapping in a full-sib family of an outbreeding species. Genet Res. 2011;93:343-9.

10. Jurka J, Kapitonov VV, Pavlicek A, Klonowski P, Kohany O, Walichiewicz J. Repbase Update, a database of eukaryotic repetitive elements. Cytogenet Genome Res. 2005;110:462-7.

11. Jurka J. Repbase update: a database and an electronic journal of repetitive elements. Trends Genet. 2000;16:418-20.

12. Xu Z, Wang H. LTR_FINDER: an efficient tool for the prediction of full-length LTR retrotransposons. Nucleic Acids Res. 2007;35:W265-8.

13. Abrusán G, Grundmann N, DeMester L, Makalowski W. TEclass—a tool for automated classification of unknown eukaryotic transposable elements. Bioinformatics. 2009;25:1329-30.

14. Elsik CG, Mackey AJ, Reese JT, Milshina NV, Roos DS, Weinstock GM. Creating a honey bee consensus gene set. Genome Biol. 2007;8:1.

15. Birney E, Clamp M, Durbin R. GeneWise and genomewise. Genome Res. 2004;14:988-95.

16. Stanke M, Keller O, Gunduz I, Hayes A, Waack S, Morgenstern B. AUGUSTUS: ab initio prediction of alternative transcripts. Nucleic acids Res. 2006;34:W435-9.

17. Aggarwal G, Ramaswamy R. Ab initio gene identification: prokaryote genome annotation with GeneScan and GLIMMER. J Biosciences. 2002;27:7-14.

18. Trapnell C, Pachter L, Salzberg SL. TopHat: discovering splice junctions with RNA-Seq. Bioinformatics. 2009;25:1105-11.

19. Trapnell C, Williams BA, Pertea G, Mortazavi A, Kwan G, Van Baren MJ, et al. Transcript assembly and quantification by RNA-Seq reveals unannotated transcripts and isoform switching during cell differentiation. Nat Biotechnol. 2010;28:511-5.

20. Bairoch A, Apweiler R. The SWISS-PROT protein sequence data bank and its supplement TrEMBL. Nucleic Acids Res. 1997;25:31-6.

21. Kanehisa M, Goto S. KEGG: kyoto encyclopedia of genes and genomes. Nucleic Acids Res. 2000;28:27-30.

22. Glaros D, LoMonte A, Ellis K, Yasumura S, Stoenner R, Cohn S. Invivo measurement of lithium in the body by a neutron activation analysis technique. Med Phys. 1986;13:45-9.

23. Quevillon E, Silventoinen V, Pillai S, Harte N, Mulder N, Apweiler R, et al. InterProScan: protein domains identifier. Nucleic Acids Res. 2005;33:W116-20.

24. Ruan J, Li H, Chen Z, Coghlan A, Coin LJM, Guo Y, et al. TreeFam: 2008 update. Nucleic Acids Res. 2008;36:D735-40.

25. Huelsenbeck JP, Ronquist F. MRBAYES: Bayesian inference of phylogenetic trees. Bioinformatics. 2001;17:754-5.

26. Edgar RC. MUSCLE: multiple sequence alignment with high accuracy and high throughput. Nucleic Acids Res. 2004;32:1792-7.

27. Yang Z. PAML 4: phylogenetic analysis by maximum likelihood. Mol Biol Evol. 2007;24:1586-91.

28. Hedges SB, Dudley J, Kumar S. TimeTree: a public knowledge-base of divergence times among organisms. Bioinformatics. 2006;22:2971-72.

29. De Bie T, Cristianini N, Demuth JP, Hahn MW. CAFE: a computational tool for the study of gene family evolution. Bioinformatics. 2006;22:1269-71.

30. Mortazavi A, Williams BA, McCue K, Schaeffer L, Wold B. Mapping and quantifying mammalian transcriptomes by RNA-Seq. Nat Method. 2008;5:621-8.

31. Gavrieli Y, Sherman Y, Ben-Sasson SA. Identification of programmed cell death in situ via specific labeling of nuclear DNA fragmentation. J Cell Biol. 1992;119:493-501.

32. Giuffre AJ, Hamm LM, Han N, De Yoreo JJ, Dove PM. Polysaccharide chemistry regulates kinetics of calcite nucleation through competition of interfacial energies. P Natl Acad Sci USA. 2013;110:9261-66.

33. Ghaleb AM, Aggarwal G, Bialkowska AB, Nandan MO, Yang VW. Notch inhibits expression of the Krüppel-like factor 4 tumor suppressor in the intestinal epithelium. Mol Cancer Res. 2008;6:1920-7.

34. Paz M, Flückiger R, Boak A, Kagan H, Gallop PM. Specific detection of quinoproteins by redox-cycling staining. J Biol Chem. 1991;266:689-92.

35. Suzuki M, Saruwatari K, Kogure T, Yamamoto Y, Nishimura T, Kato T, et al. An acidic matrix protein, Pif, is a key macromolecule for nacre formation. Science. 2009;325:1388-90.

36. Yano M, Nagai K, Morimoto K, Miyamoto H. A novel nacre protein N19 in the pearl oyster *Pinctada fucata*. Biochem Bioph Res Co. 2007;362:158-63.

37. Seaver RW. A partial skeletal proteome of the brittle star Ophiocoma wendtii. 2013.

38. Langfelder P, Horvath S. WGCNA: an R package for weighted correlation network analysis. BMC Bioinformatics. 2008;9:1.

# SI Tables

**Table S1. Summary statistics of WGS assembly produced with SOAPdenovo.**

|  | **Contig** | | **Scaffold** | |
| --- | --- | --- | --- | --- |
|  | **Size(bp)** | **Number** | **Size(bp)** | **Number** |
| N90 | 122 | 2,743,734 | 473 | 164,771 |
| N80 | 147 | 2,113,960 | 7,680 | 29,845 |
| N70 | 175 | 1,584,381 | 21,250 | 18,949 |
| N60 | 215 | 1,145,563 | 33,040 | 13,107 |
| N50 | 271 | 792,469 | 45,633 | 9,108 |
| Longest | 17,290 | ---- | 1,198,491 | ---- |
| Total Size | 847,256,355 | ---- | 1,555,715,031 | ---- |
| Total Number(≥100bp) | ---- | 3,513,616 | ---- | 1,088,247 |
| Total Number(≥2kb) | ---- | 9,387 | ---- | 44,060 |

**Table S2. Summary statistics of the final assembly produced with the BAC-to-BAC strategy.**

| Type | Contig  Size(bp) | Number | Scaffold  Size(bp) | Number |
| --- | --- | --- | --- | --- |
| N90 | 5,299 | 43,153 | 100,146 | 2,991 |
| N80 | 9,090 | 30,602 | 161,814 | 2,225 |
| N70 | 12,923 | 22,499 | 211,875 | 1,692 |
| N60 | 16,927 | 16,539 | 267,129 | 1,274 |
| N50 | 21,481 | 11,908 | 324,310 | 939 |
| Longest | 243,375 | ----- | 5,897,787 | ----- |
| Total Size | 882,582,812 | ----- | 990,658,107 | ----- |
| Total Number(≥150bp) | ----- | 94,490 | ----- | 8,621 |
| Total Number(≥2kb) | ----- | 63,301 | ----- | 8,587 |

**Table S3. Mapping assessment of the assembly with 4 Sanger-sequenced BACs.**

| BAC ID | BAC length | Coverage (%) | Scaffold number | Scaffold length |
| --- | --- | --- | --- | --- |
| ibmaxa | 111,512 | 94.81 | 2 | 155,405 |
| ibmbxa | 96,567 | 95.58 | 1 | 82,983 |
| ibmdxa | 114,776 | 93.2 | 2 | 103,955 |
| ibmexa | 105,430 | 94.49 | 2 | 108,426 |

**Table S4. Mapping assessment of the genome assembly with transcripts assembled from RNA-seq reads and CDS sequences of Takeuchi et al. (2011)**

| **Dataset** | **Number** | **Total Length (bp)** | **Covered by Assembly** | **With >90% Sequence in one Scaffold** | | **With >50% Sequence in one Scaffold** | |
| --- | --- | --- | --- | --- | --- | --- | --- |
|  |  |  |  | **Number** | **Percent (%)** | **Number** | **Percent (%)** |
| All | 117,102 | 70,840,225 | 98.1 | 110,532 | 94.39 | 116,902 | 99.83 |
| >200bp | 117,102 | 70,840,225 | 98.1 | 110,532 | 94.39 | 116,902 | 99.83 |
| >500bp | 39,691 | 47,175,672 | 97.95 | 37,080 | 93.42 | 39,616 | 99.81 |
| >1000bp | 16,281 | 30,997,699 | 97.75 | 14,971 | 91.95 | 16,247 | 99.79 |
| pfu_aug1.0 | 72,597 | 44,346,368 | 92.55 | 66,582 | 91.71 | 69,318 | 95.48 |

**Table S5. Statistics of the linkage map and assembly anchored by genetic markers.**

| **Linkage Group** | **Marker NO.** | **Linkage Size(cM)** | **Average space(cM)** | **Physical size(Mb)** |
| --- | --- | --- | --- | --- |
| 1 | 516 | 474.36 | 0.92 | 104.9 |
| 2 | 259 | 211.05 | 0.81 | 60.16 |
| 3 | 506 | 449.4 | 0.88 | 91.75 |
| 4 | 305 | 224.37 | 0.73 | 57.29 |
| 5 | 276 | 176.29 | 0.63 | 54.68 |
| 6 | 248 | 192.45 | 0.77 | 53.64 |
| 7 | 200 | 288.26 | 1.44 | 40.41 |
| 8 | 156 | 220.88 | 1.42 | 26.97 |
| 9 | 501 | 398.59 | 0.8 | 98.08 |
| 10 | 421 | 360.38 | 0.86 | 87.8 |
| 11 | 325 | 258.07 | 0.79 | 59.23 |
| 12 | 231 | 332.67 | 1.44 | 48.97 |
| 13 | 127 | 187.42 | 1.47 | 26.15 |
| 14 | 392 | 513.42 | 1.31 | 47.03 |
| Total | 4,463 | 4,287.61 | 0.96 | 857.07 |

**Table S6. Description of 11 organs/tissues and 12 developmental samples used for transcriptome sequencing.**

| **Sample name** | **Figure label** | **Sampling time** | **Seawater temperature** |
| --- | --- | --- | --- |
| Egg | E | - | 24 |
| Fertilization | Fe | 30min | 24 |
| Blastula | B | 5h25min | 24.2 |
| Gastrula | G | 6h30min | 24.2 |
| Early trochophore | ET | 8h25min | 24.2 |
| Trochophore | T | 15h45min | 24.5 |
| D-stage | D | 19h5min | 24.5 |
| D-stage Before feeding | DF | 4d | 25 |
| Early umbo larvae | EU | 14d | 26.8 |
| Eyed larvae | EL | 28d | 27.5 |
| Spat | S | 40d | 28.6 |
| Juveniles | J | 90d | 30.5 |
| Adductor muscle | A | - | - |
| Mantle pallium | MP | - | - |
| Mantle edge | ME | - | - |
| Mantle | DYW-L | - | - |
| Mantle | DYW-S | - | - |
| Hepatopancreas | He | - | - |
| Hemocyte | BC | - | - |
| Gonad | Go | - | - |
| Gill | Gi | - | - |
| Foot | F | - | - |
| Pearl sac at 180 d after nucleus transplantation | PS | - | - |

**Table S7. Summary of genes annotated in *P. f. martensii* genome.**

| **Gene set** | **Number** | **Average transcript length (bp)** | **Average CDS length (bp)** | **Average exons per gene** | **Average exon length (bp)** | **Average intron length (bp)** |
| --- | --- | --- | --- | --- | --- | --- |
| Augustus | 47,509 | 7296.73 | 1300.07 | 4.52 | 287.66 | 1703.84 |
| Genscan | 65,131 | 8154.44 | 1054.28 | 4.5 | 234.07 | 2026.28 |
| *C. gigas* | 29,323 | 5077.47 | 948.31 | 4 | 237.68 | 1381.06 |
| *L. gigantea* | 32,286 | 3523.05 | 785.35 | 3.31 | 237.22 | 1184.81 |
| *C. teleta* | 37,717 | 2722.77 | 623.89 | 2.76 | 225.74 | 1189.99 |
| *D. melanogaster* | 11,212 | 4543.98 | 808.72 | 4.02 | 201.31 | 1237.95 |
| RNA-seq | 41,741 | 5193.19 | 780.24 | 3.7 | 210.79 | 1631.74 |
| Glean | 36,120 | 9130.1 | 1496.54 | 5.26 | 284.38 | 1790.88 |
| Final gene set | 32,937 | 9655.79 | 1515.47 | 5.84 | 259.64 | 1682.97 |

**Table S8. Summary of gene functional annotation of *P. f. martensii*.**

|  | **Number** | **Percent** |
| --- | --- | --- |
| Total | 32,937 | 100 |
| Annotated | 27,654 | 83.96 |
| Swissprot | 20,834 | 63.25 |
| TrEMBL | 26,186 | 79.5 |
| InterPro | 21,951 | 66.65 |
| KEGG | 16,859 | 51.19 |
| GO | 16,674 | 50.62 |
| Unannotated | 5,283 | 16.04 |

**Table S9. Summary of repeat content of *P. f. martensii* genome.**

| **Type** | **RepBase TEs** | | **TE Proteins** | | ***De novo*** | | **Combined TEs** | |
| --- | --- | --- | --- | --- | --- | --- | --- | --- |
|  | **Length (bp)** | **% in Genome** | **Length (bp)** | **% in Genome** | **Length (bp)** | **% in Genome** | **Length (bp)** | **% in Genome** |
| DNA | 9,725,120 | 0.981683 | 5,443,222 | 0.549455 | 61,835,240 | 6.241835 | 68,348,668 | 6.89932 |
| LINE | 3,305,503 | 0.333667 | 14,407,599 | 1.454346 | 74,357,736 | 7.505893 | 79,428,523 | 8.017753 |
| SINE | 252,824 | 0.025521 | 0 | 0 | 3,127,333 | 0.315682 | 3,216,289 | 0.324662 |
| LTR | 5,964,006 | 0.602025 | 9,050,009 | 0.913535 | 11,895,922 | 1.20081 | 17,398,676 | 1.756275 |
| Other | 1,738 | 0.000175 | 0 | 0 | 0 | 0 | 1,738 | 0.000175 |
| Unknown | 0 | 0 | 0 | 0 | 343,126,397 | 34.63621 | 343,126,397 | 34.63621 |
| Total | 18,699,383 | 1.887572 | 28,882,471 | 2.915483 | 467,689,014 | 47.20993 | 480,505,615 | 48.50368 |

**Table S10. Gene number in biomineralization-related gene families in skeleton-containing species.**

| **kegg ontology** | **Aqu^*^** | **Adi^*^** | **Pma^*^** | **Cgi^*^** | **Lgi^*^** | **Dre^#^** | **Lan^#^** | **Hsa^#^** | **Obi** | **Cte** | **Hro** | **Descriptions** |
| --- | --- | --- | --- | --- | --- | --- | --- | --- | --- | --- | --- | --- |
| K01183 | 2 | 0 | 15 | 18 | 23 | 6 | 23 | 6 | 34 | 10 | 4 | chitinase (EC:3.2.1.14) |
| K00698 | 3 | 1 | 11 | 22 | 14 | 4 | 23 | 0 | 0 | 6 | 2 | chitin synthase (EC:2.4.1.16) |
| K00505 | 4 | 4 | 53 | 26 | 3 | 1 | 5 | 1 | 16 | 2 | 1 | tyrosinase (EC:1.14.18.1) |
| K01017 | 0 | 1 | 13 | 4 | 4 | 1 | 16 | 1 | 11 | 38 | 0 | chondroitin 4-sulfotransferase 11 (EC:2.8.2.5) |
| K09671 | 0 | 0 | 4 | 1 | 0 | 1 | 0 | 1 | 0 | 3 | 0 | carbohydrate 6-sulfotransferase 6 (EC:2.8.2.-) |
| K09673 | 0 | 0 | 6 | 0 | 1 | 0 | 10 | 1 | 0 | 2 | 0 | carbohydrate 4-sulfotransferase 9 (EC:2.8.2.-) |
| K08105 | 3 | 3 | 4 | 0 | 0 | 1 | 0 | 1 | 0 | 7 | 0 | dermatan 4-sulfotransferase 1 (EC:2.8.2.-) |
| K01020 | 0 | 2 | 7 | 0 | 1 | 2 | 6 | 1 | 0 | 5 | 0 | chondroitin 6-sulfotransferase 3 (EC:2.8.2.17) |
| K05496 | 0 | 0 | 1 | 2 | 0 | 3 | 0 | 2 | 0 | 1 | 0 | bone morphogenetic protein 3/3B |
| K04662 | 0 | 2 | 3 | 3 | 2 | 6 | 3 | 2 | 4 | 1 | 3 | bone morphogenetic protein 2/4 |
| K04663 | 4 | 4 | 2 | 2 | 3 | 5 | 7 | 5 | 4 | 3 | 1 | bone morphogenetic protein 5/6/7/8 |
| K05503 | 0 | 0 | 2 | 0 | 1 | 3 | 0 | 2 | 1 | 1 | 1 | bone morphogenetic protein 9/10 |
| K13375 | 0 | 0 | 0 | 0 | 0 | 2 | 0 | 1 | 0 | 0 | 0 | transforming growth factor beta-1 |
| K13376 | 1 | 0 | 0 | 0 | 0 | 2 | 0 | 1 | 0 | 0 | 0 | transforming growth factor beta-2 |
| K13377 | 1 | 0 | 0 | 0 | 0 | 1 | 0 | 1 | 0 | 0 | 0 | transforming growth factor beta-3 |

Species with calcium carbonate skeleton are remarked by **^*^.** Species with calcium phosphate skeleton are marked by **^#^**. Species names are abbreviated is provided in Table S11 below.

**Table S11. Abbreviations of domain and species** **names.**

| **Abbreviation** | **Full** |
| --- | --- |
| THR | Collagen triple helix repeat (IPR008160) |
| VWA | von Willebrand factor, type A (IPR002035) |
| Adi | *Acropora digitifera* |
| Aqu | *Amphimedon queenslandica* |
| Cgi | *Crassostrea gigas* |
| Cin | *Ciona intestinalis* |
| Cte | *Capitella teleta* |
| Dme | *Drosophila melanogaster* |
| Dre | *Danio rerio* |
| Hro | *Helobdella robusta* |
| Hsa | *Homo sapiens* |
| Lan | *Lingula anatina* |
| Lgi | *Lottia gigantea* |
| Mmu | *Mus musculus* |
| Obi | *Octopus bimaculoides* |
| Pma | *Pinctada fucata martensii* |
| Ptr | *Pan troglodytes* |
|  |  |

**Table S12. Proteins and their accession numbers used for phylogenetic analysis of TGF-β1/2/3 and bone morphogenetic proteins (BMP).**

| **Protein name** | **accession numbers** |
| --- | --- |
| Ptr BMP10 | XP_525772 |
| Lan BMP7-5 | XP_013393347 |
| Lan BMP7-4 | XP_013408966 |
| Lan BMP7-3 | XP_013393349 |
| Lan BMP7-2 | XP_013393348 |
| Lan BMP7-1 | XP_013385477 |
| Lan BMP6-2 | XP_013385478 |
| Lan BMP4-2 | XP_013396300 |
| Lan BMP4-1 | XP_013396301 |
| Lan BMP3 | XP_013420458 |
| Lan BMP1-6 | XP_013400108 |
| Lan BMP1-5 | XP_013405264 |
| Lan BMP1-4 | XP_013415894 |
| Lan BMP1-3 | XP_013388808 |
| Lan BMP1-2 | XP_013385673 |
| Lan BMP1-1 | XP_013390346 |
| Hsa TGF-β3 | AAC79727 |
| Hsa TGF-β2 | AAA50405 |
| Hsa TGF-β1 | NP_000651 |
| Hsa BMP15 | NP_005439 |
| Hsa BMP11 | AAC72852 |
| Hsa BMP10 | O95393 |
| Hsa BMP9 | AAD56960 |
| Hsa BMP7 | AAH08584 |
| Hsa BMP6 | EAW55219 |
| Hsa BMP5 | AAA36736 |
| Hsa BMP4 | AAH20546 |
| Hsa BMP3 | AAI17515 |
| Hsa BMP2 | NP_001191 |
| Hsa BMP1 | AAI36680 |
| GDNF_MOUSE | P48540 |
| Dre TGF-β3 | AAH81579 |
| Dre TGF-β2 | NP_919366 |
| Dre TGF-β1 | AAI62366 |
| Dre BMP15 | AAI24107 |
| Dre BMP10 | NP_001124072 |
| Dre BMP8A | NP_001038436 |
| Dre BMP7 | AAF17558 |
| Dre BMP6 | AAH90689 |
| Dre BMP5 | AAH54647 |
| Dre BMP4 | AAC13302 |
| Dre BMP3 | NP_001071233 |
| Dre BMP2 | AAC25595 |
| Dre BMP1 | ABD90687 |
| Cgi gonadal TGF-βlike | ABU50369 |
| Cgi BMP7-2 | XP_011426448 |
| Cgi BMP7-1 | EKC34211 |
| Cgi BMP3-3 | EKC42418 |
| Cgi BMP3-2 | EKC19783 |
| Cgi BMP3-1 | EKC34713 |
| Cgi BMP2B | EKC42419 |
| Cgi BMP2 | EKC18750 |

**Table S13. The best hits of VWA domains of VWAPs from shell matrix of *P. f. martensii* on proteins of *H. sapiens* and *M. musculus*.**

| geneID | Best hit VWA domain | Name of best hit protein |
| --- | --- | --- |
| Pma_44.534 | A2AX52 _1028-1204 | Col6A4 |
| Pma_10010155 | O08746_653-831 | Matrilin-2 |
| Pma_10011421 | B7ZNH7 _1028-1208 | Col14A1 |
| Pma_10015641 | A2AX52 _1028-1204 | Col6A4 |
| Pma_10023204-1 | O00339 _55-237 | Matrilin-2 |
| Pma_10023204-2 | Q60847-2_1197-1376 | Col12A1 |
| Pma_10003827 | Q14624 _272-456 | Inter-alpha-trypsin inhibitor heavy chain H4 |
| Pma_384.57 | A6X935 _272-456 | Inter-alpha-trypsin inhibitor heavy chain H4 |
| Pma_10006533 | P20702 _149-334 | Intrigin X |
| Pma_10011175-1 | Q99715 _438-617 | Col12A1 |
| Pma_10011175-2 | Q05707 _156-335 | Col14A1 |
| Pma_10011175-3 | O15232 _81-260 | Matrilin-3 |
| Pma_10011175-4 | O35701E_76-255 | Matrilin-3 |
| Pma_10011175-5 | B7ZNH7 _1028-1208 | Col14A1 |
| Pma_10011175-6 | Q8C6K9 _806-982 | Col6A6 |
| Pma_10011175-7 | Q60847_138-317 | Col12A1 |
| Pma_10011175-8 | Q99715 _138-317 | Col12A1 |
| Pma_10011175-9 | Q99715 _138-317 | Col12A1 |
| Pma_10011175-10 | B7ZNH7 _157-336 | Col14A1 |
| Pma_10011175-11 | Q99715 _138-317 | Col12A1 |
| Pma_10011175-12 | Q05707 _156-335 | Col14A1 |
| Pma_10011175-13 | Q60847_138-317 | Col12A1 |
| Pma_10011175-14 | Q99715 _1197-1376 | Col12A1 |
| Pma_10011175-15 | Q99715 _138-317 | Col12A1 |
| Pma_10011175-16 | Q05707 _156-335 | Col14A1 |
| Pma_10011175-17 | Q60847_138-317 | Col12A1 |
| Pma_10011175-18 | Q05707 _1030-1210 | Col14A1 |
| Pma_10011175-19 | Q99715 _138-317 | Col12A1 |
| Pma_10011175-20 | Q99715 _138-317 | Col12A1 |
| Pma_10011175-21 | Q99715 _438-617 | Col12A1 |
| Pma_10011175-22 | Q05707 _156-335 | Col14A1 |
| Pma_10011175-23 | Q05707 _156-335 | Col14A1 |
| Pma_10011175-24 | Q99715 _138-317 | Col14A1 |
| Pma_10011175-25 | E9Q7P1 _45-227 | Col22A1 |
| Pma_10011175-26 | P12111 _37-215 | Col6A3 |
| Pma_10011175-27 | Q60847_138-317 | Col12A1 |
| Pma_10011175-28 | A2AX52 _1028-1204 | Col6A4 |
| Pma_10019835-1 | Q99715 _1197-1376 | Col12A1 |
| Pma_10019835-2 | B7ZNH7 _1028-1208 | Col14A1 |
| Pma_10019835-3 | Q05707 _1030-1210 | Col14A1 |
| Pma_10019835-4 | Q05707 _1030-1210 | Col14A1 |
| Pma_530.149-1 | Q8NFW1 _36-218 | Col22A1 |
| Pma_530.149-2 | B7ZNH7 _1028-1208 | Col14A1 |
| Pma_530.149-3 | O08746_653-831 | Matrilin-2 |
| Pma_10019836-1 | Q99715 _138-317 | Col12A1 |
| Pma_10019836-2 | Q60847_138-317 | Col12A1 |
| Pma_10019836-3 | O35701 _76-255 | Matrilin-3 |

**Table S14. Gene IDs of *P. f. martensii* tyrosinases used for phylogenetic analysis.**

| **GeneID** | |
| --- | --- |
| Pma_10031907 | Pma _10018719 |
| Pma_10031227 | Pma_10018717 |
| Pma_10030752 | Pma_10016804 |
| Pma_10029262 | Pma_10016803 |
| Pma_10029261 | Pma_10016802 |
| Pma_10029259 | Pma_10016801 |
| Pma_10029257 | Pma_10016600 |
| Pma_10028751 | Pma_10016044 |
| Pma_10028311 | Pma_10015392 |
| Pma_10028307 | Pma_10015306 |
| Pma_10028201 | Pma_10014430 |
| Pma_10028200 | Pma_10013987 |
| Pma_10027316 | Pma_10013533 |
| Pma_10027315 | Pma_10013532 |
| Pma_10024934 | Pma_10012735 |
| Pma_10024726 | Pma_10012622 |
| Pma_10024674 | Pma_10011382 |
| Pma_10023166 | Pma_10007695 |
| Pma_10022578 | Pma_10007409 |
| Pma_10021622 | Pma_10005803 |
| Pma_10021425 | Pma_10005299 |
| Pma_10021422 | Pma_10005159 |
| Pma_10021421 | Pma_10004511 |
| Pma_10020692 | Pma_10004452 |
| Pma_10018775 | Pma_10004451 |
| Pma_10018772 | Pma_10003264 |
| Pma_10001525 |  |

**Table S15. KEGG enrichment of genes co-expressed with genes encoding nacre proteins.**

| **Pathway** | **DEGs with pathway annotation (2135)** | **All genes with pathway annotation (15372)** | **P value** |
| --- | --- | --- | --- |
| Lysosome | 73 (3.42%) | 260 (1.69%) | 1.28E-09 |
| ABC transporters | 27 (1.26%) | 73 (0.47%) | 6.85E-07 |
| Metabolic pathways | 338 (15.83%) | 1967 (12.8%) | 5.88E-06 |
| Glycerophospholipid metabolism | 35 (1.64%) | 123 (0.8%) | 1.80E-05 |
| Sphingolipid metabolism | 19 (0.89%) | 56 (0.36%) | 0.000118 |
| Glycosaminoglycan degradation | 18 (0.84%) | 53 (0.34%) | 0.000174 |
| Protein processing in endoplasmic reticulum | 55 (2.58%) | 247 (1.61%) | 0.000214 |
| Glycerolipid metabolism | 22 (1.03%) | 74 (0.48%) | 0.000314 |
| ErbB signaling pathway | 22 (1.03%) | 78 (0.51%) | 0.000702 |
| Gap junction | 26 (1.22%) | 101 (0.66%) | 0.001103 |
| Glycolysis / Gluconeogenesis | 17 (0.8%) | 58 (0.38%) | 0.001733 |
| Amino sugar and nucleotide sugar metabolism | 26 (1.22%) | 105 (0.68%) | 0.002014 |
| Epithelial cell signaling in Helicobacter pylori infection | 21 (0.98%) | 86 (0.56%) | 0.006156 |
| Vibrio cholerae infection | 20 (0.94%) | 82 (0.53%) | 0.007497 |
| Cyanoamino acid metabolism | 6 (0.28%) | 14 (0.09%) | 0.007878 |
| Bile secretion | 27 (1.26%) | 122 (0.79%) | 0.008632 |
| Glycosylphosphatidylinositol(GPI)-anchor biosynthesis | 10 (0.47%) | 32 (0.21%) | 0.009191 |
| Other types of O-glycan biosynthesis | 14 (0.66%) | 53 (0.34%) | 0.011514 |
| Wnt signaling pathway | 31 (1.45%) | 148 (0.96%) | 0.011636 |
| Neurotrophin signaling pathway | 39 (1.83%) | 198 (1.29%) | 0.014248 |
| Aldosterone-regulated sodium reabsorption | 10 (0.47%) | 34 (0.22%) | 0.014412 |
| Pancreatic cancer | 16 (0.75%) | 66 (0.43%) | 0.01666 |
| Adherens junction | 30 (1.41%) | 146 (0.95%) | 0.016727 |
| Colorectal cancer | 16 (0.75%) | 67 (0.44%) | 0.019146 |
| Jak-STAT signaling pathway | 13 (0.61%) | 51 (0.33%) | 0.019613 |
| Protein digestion and absorption | 40 (1.87%) | 209 (1.36%) | 0.020669 |
| Melanoma | 9 (0.42%) | 31 (0.2%) | 0.021586 |
| Citrate cycle (TCA cycle) | 9 (0.42%) | 31 (0.2%) | 0.021586 |
| Dilated cardiomyopathy | 31 (1.45%) | 155 (1.01%) | 0.021831 |
| Cytokine-cytokine receptor interaction | 15 (0.7%) | 63 (0.41%) | 0.023447 |
| Pancreatic secretion | 35 (1.64%) | 181 (1.18%) | 0.025128 |
| Fc epsilon RI signaling pathway | 12 (0.56%) | 48 (0.31%) | 0.028212 |
| Riboflavin metabolism | 16 (0.75%) | 70 (0.46%) | 0.028317 |
| Collecting duct acid secretion | 9 (0.42%) | 33 (0.21%) | 0.032007 |
| Other glycan degradation | 11 (0.52%) | 44 (0.29%) | 0.034803 |
| Fat digestion and absorption | 14 (0.66%) | 61 (0.4%) | 0.037407 |
| Nicotinate and nicotinamide metabolism | 9 (0.42%) | 34 (0.22%) | 0.038341 |
| Butirosin and neomycin biosynthesis | 3 (0.14%) | 6 (0.04%) | 0.038596 |
| Circadian rhythm - mammal | 6 (0.28%) | 19 (0.12%) | 0.038622 |
| Osteoclast differentiation | 15 (0.7%) | 67 (0.44%) | 0.039075 |
| Mucin type O-Glycan biosynthesis | 11 (0.52%) | 45 (0.29%) | 0.040484 |
| N-Glycan biosynthesis | 18 (0.84%) | 85 (0.55%) | 0.042222 |
| B cell receptor signaling pathway | 14 (0.66%) | 62 (0.4%) | 0.042366 |
| ECM-receptor interaction | 47 (2.2%) | 265 (1.72%) | 0.04453 |
| Pantothenate and CoA biosynthesis | 5 (0.23%) | 15 (0.1%) | 0.046357 |
| Non-small cell lung cancer | 11 (0.52%) | 46 (0.3%) | 0.046775 |

**Table S16. Gene Ontology enrichment of genes co-expressed with nacre protein encoding genes.**

| **Accession** | **Gene Ontology term** | **Corrected P-value** | **Category** |
| --- | --- | --- | --- |
| GO:0016020 | membrane | 5.90E-25 | Component |
| GO:0044425 | membrane part | 4.61E-07 | Component |
| GO:0016021 | integral to membrane | 1.74E-05 | Component |
| GO:0031224 | intrinsic to membrane | 2.24E-05 | Component |
| GO:0030136 | clathrin-coated vesicle | 0.00034 | Component |
| GO:0008021 | synaptic vesicle | 0.00058 | Component |
| GO:0030135 | coated vesicle | 0.00101 | Component |
| GO:0012505 | endomembrane system | 0.00484 | Component |
| GO:0016023 | cytoplasmic membrane-bounded vesicle | 0.00888 | Component |
| GO:0031410 | cytoplasmic vesicle | 0.00888 | Component |
| GO:0031982 | vesicle | 0.00888 | Component |
| GO:0031988 | membrane-bounded vesicle | 0.00888 | Component |
| GO:0005215 | transporter activity | 1.86E-10 | Function |
| GO:0022804 | active transmembrane transporter activity | 7.68E-08 | Function |
| GO:0008324 | cation transmembrane transporter activity | 1.45E-07 | Function |
| GO:0022857 | transmembrane transporter activity | 4.05E-07 | Function |
| GO:0042626 | ATPase activity, coupled to transmembrane movement of substances | 6.79E-07 | Function |
| GO:0043492 | ATPase activity, coupled to movement of substances | 6.79E-07 | Function |
| GO:0016820 | hydrolase activity, acting on acid anhydrides, catalyzing transmembrane movement of substances | 1.01E-06 | Function |
| GO:0015399 | primary active transmembrane transporter activity | 2.21E-06 | Function |
| GO:0015405 | P-P-bond-hydrolysis-driven transmembrane transporter activity | 2.21E-06 | Function |
| GO:0022891 | substrate-specific transmembrane transporter activity | 6.32E-06 | Function |
| GO:0022890 | inorganic cation transmembrane transporter activity | 1.46E-05 | Function |
| GO:0022892 | substrate-specific transporter activity | 2.56E-05 | Function |
| GO:0046873 | metal ion transmembrane transporter activity | 3.99E-05 | Function |
| GO:0015075 | ion transmembrane transporter activity | 5.82E-05 | Function |
| GO:0015077 | monovalent inorganic cation transmembrane transporter activity | 6.84E-05 | Function |
| GO:0016773 | phosphotransferase activity, alcohol group as acceptor | 9.91E-05 | Function |
| GO:0016301 | kinase activity | 0.00034 | Function |
| GO:0042625 | ATPase activity, coupled to transmembrane movement of ions | 0.00067 | Function |
| GO:0015662 | ATPase activity, coupled to transmembrane movement of ions, phosphorylative mechanism | 0.00406 | Function |
| GO:0016798 | hydrolase activity, acting on glycosyl bonds | 0.01144 | Function |
| GO:0003824 | catalytic activity | 0.01208 | Function |
| GO:0003924 | GTPase activity | 0.01961 | Function |
| GO:0004553 | hydrolase activity, hydrolyzing O-glycosyl compounds | 0.04454 | Function |
| GO:0015079 | potassium ion transmembrane transporter activity | 0.04912 | Function |
| GO:0051179 | localization | 2.11E-09 | process |
| GO:0006810 | transport | 5.42E-09 | process |
| GO:0051234 | establishment of localization | 5.42E-09 | process |
| GO:0055085 | transmembrane transport | 3.07E-06 | process |
| GO:0006812 | cation transport | 2.23E-05 | process |
| GO:0015672 | monovalent inorganic cation transport | 0.00158 | process |
| GO:0008610 | lipid biosynthetic process | 0.0034 | process |
| GO:0006913 | nucleocytoplasmic transport | 0.00878 | process |
| GO:0051169 | nuclear transport | 0.00878 | process |
| GO:0055086 | nucleobase-containing small molecule metabolic process | 0.00884 | process |
| GO:0006629 | lipid metabolic process | 0.01544 | process |
| GO:0044281 | small molecule metabolic process | 0.01855 | process |
| GO:0009150 | purine ribonucleotide metabolic process | 0.03232 | process |
| GO:0009141 | nucleoside triphosphate metabolic process | 0.04535 | process |
| GO:0009144 | purine nucleoside triphosphate metabolic process | 0.04535 | process |
| GO:0009199 | ribonucleoside triphosphate metabolic process | 0.04535 | process |
| GO:0009205 | purine ribonucleoside triphosphate metabolic process | 0.04535 | process |
| GO:0030001 | metal ion transport | 0.04711 | process |

**Table S17. Primer sequences for generating dsRNAs and for qRT-PCR quantification of expression of 6 *P. f. martensii VWAP*s.**

| **Primer** | **Sequence** |
| --- | --- |
| Pma_10019836-RNAi-F | GCGTAATACGACTCACTATAGGGTGAACTGACAGGAATGGTTGGAGGAA |
| Pma_10019836-RNAi-R | GCGTAATACGACTCACTATAGGGGCTGACCGAGACCCGCTACAAAT |
| Pma_530.149-RNAi-R | GCGTAATACGACTCACTATAGGGACCACTCCTATCCTTGGAACCCC |
| Pma_530.149-RNAi-F | GCGTAATACGACTCACTATAGGGGTTGTTCAGATTGCCATCGTGCT |
| Pma_10019835-RNAi-R | GCGTAATACGACTCACTATAGGGAAGAGATGGTCGCTTACGGCATTAGAT |
| Pma_10019835-RNAi-F | GCGTAATACGACTCACTATAGGGAAAGGAGTGGAACGGTGGGTGTG |
| Pma_44.534-RNAi-F | GCGTAATACGACTCACTATAGGGGCTTGGTGAACTAACGAAAGATGGA |
| Pma_44.534-RNAi-R | GCGTAATACGACTCACTATAGGGTTGAACTAGGAATATTGATTGGCGG |
| Pma_10015641-RNAi-F | GCGTAATACGACTCACTATAGGGACCGACCTCCTGTCCAGTTCTTG |
| Pma_10015641-RNAi -R | GCGTAATACGACTCACTATAGGGCTGTGGCATTGTCTCCATTACCG |
| Pma_10011175-RNAi-F | GCGTAATACGACTCACTATAGGGTGTCAGATCATTGCGTGTGTTACC |
| Pma_10011175-RNAi-R | GCGTAATACGACTCACTATAGGGTTGTCTTTTCCTTCTTCGGGAAG |
| RFP-RNAi-F | GCGTAATACGACTCACTATAGGGCTGTCCCCCCAGTTCCAGTAC |
| RFP-RNAi-R | GCGTAATACGACTCACTATAGGGCGTTGTGGGAGGTGATGTCCAGCT |
| Pma_10011175-qPCR-F | CATTCCCGAGGCAGAGCA |
| Pma_10011175-qPCR-R | TGACGATTGGCTGTGTTGTAGG |
| Pma_10019835-qPCR–F | TGAAAATCGCCGTGGTGA |
| Pma_10019835-qPCR–R | CAACATTGCTAATCCGTCCAC |
| Pma_44.534-qPCR-F | ACAAATGCCATCGGAACGG |
| Pma_44.534-qPCR-R | CAGGTGGTAAGCGAGAGGAGAA |
| Pma_10015641-qPCR-F | ATTGGTGCCCTCGTTTTCA |
| Pma_10015641-qPCR-R | GCGTCTCCTTCTCCCTTGCTA |
| Pma_10011175-qPCR-F | CGTCAGTGCATGCGTTCG |
| Pma_10011175-qPCR-R | TGGTGGGAGCCAGCGTAG |
| Pma_530.149-qPCR-R | GCGAATACGATCAATCCAACC |
| Pma_530.149-qPCR-F | CCACTACCGCAGGAAATCAG |
| GAPDH-F | GCAGATGGTGCCGAGTATGT |
| GAPDH-R | CGTTGATTATCTTGGCGAGTG |
