# Supplementary figures and images for: The pearl oyster Pinctada fucata martensii genome and multi-omic analyses provide insights into biomineralization
Source: Gigascience. 2017 Jul 25;6(8):1–12. doi: 10.1093/gigascience/gix059 (PMC5597905; doi:10.1093/gigascience/gix059)

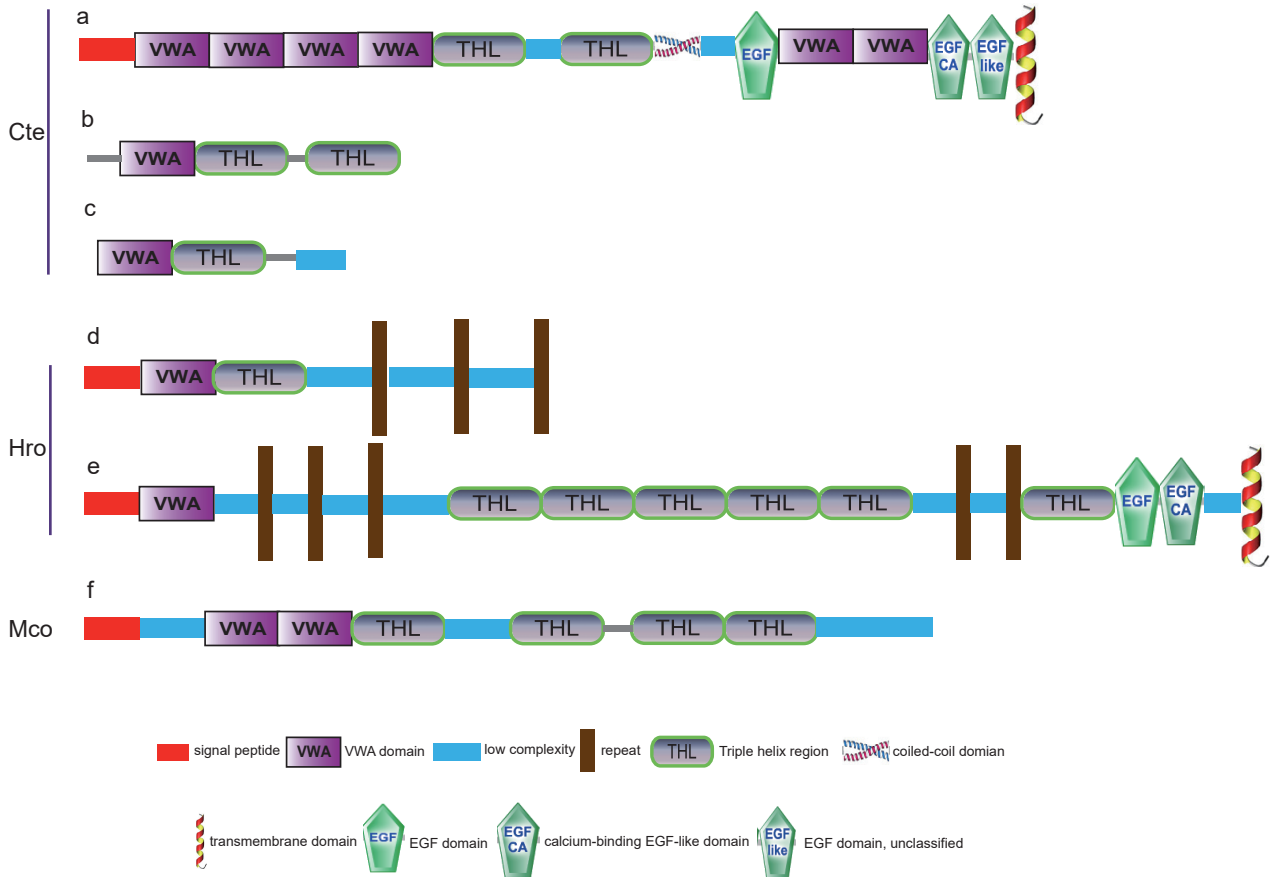

Supplement: Additional Files [file gix059_Supp.zip › Additional file 10 Figure S8.pdf]

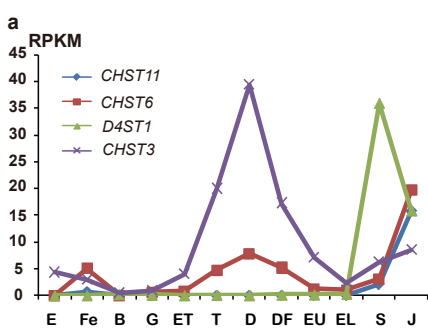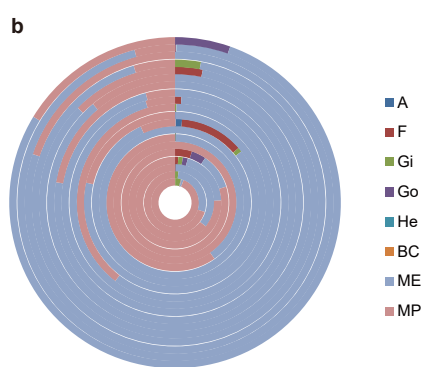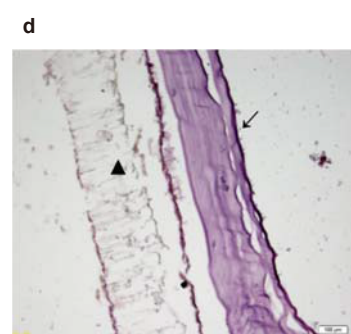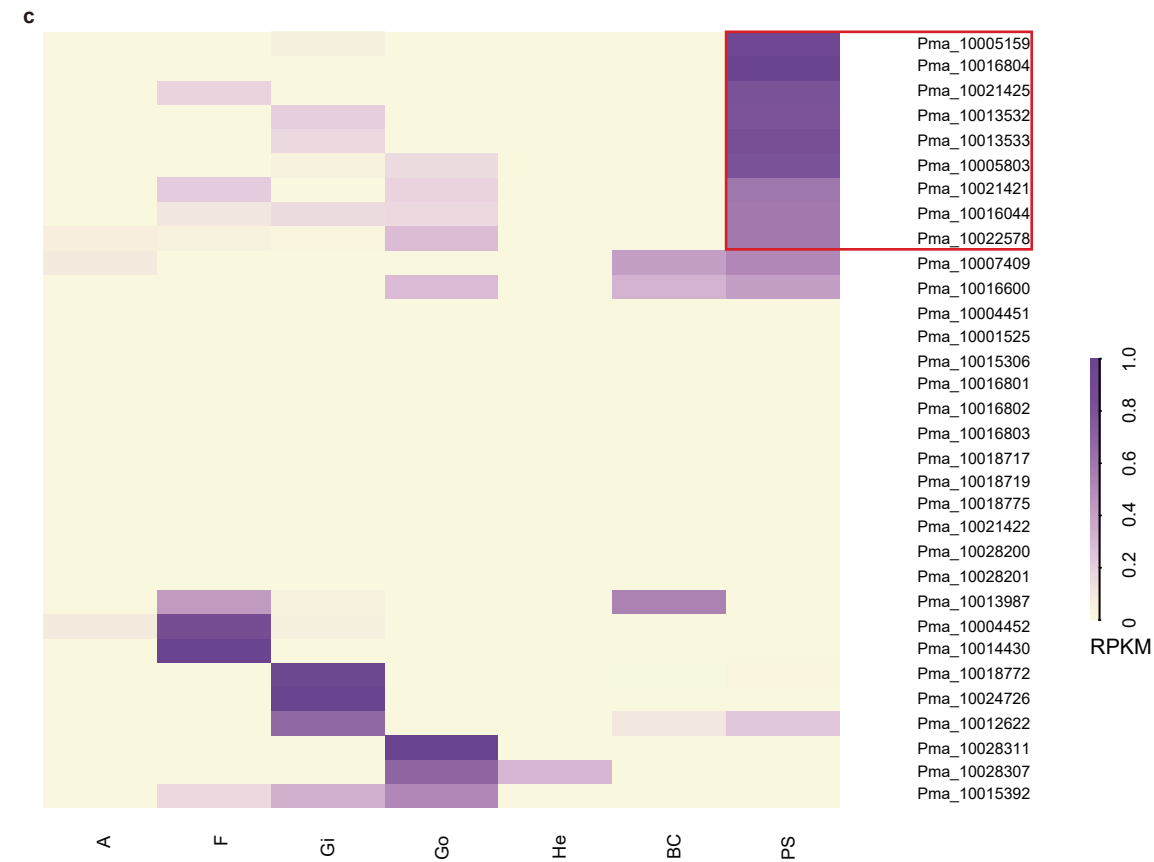

Supplement: Additional Files [file gix059_Supp.zip › Additional file 11.figure S9-0619.pdf]

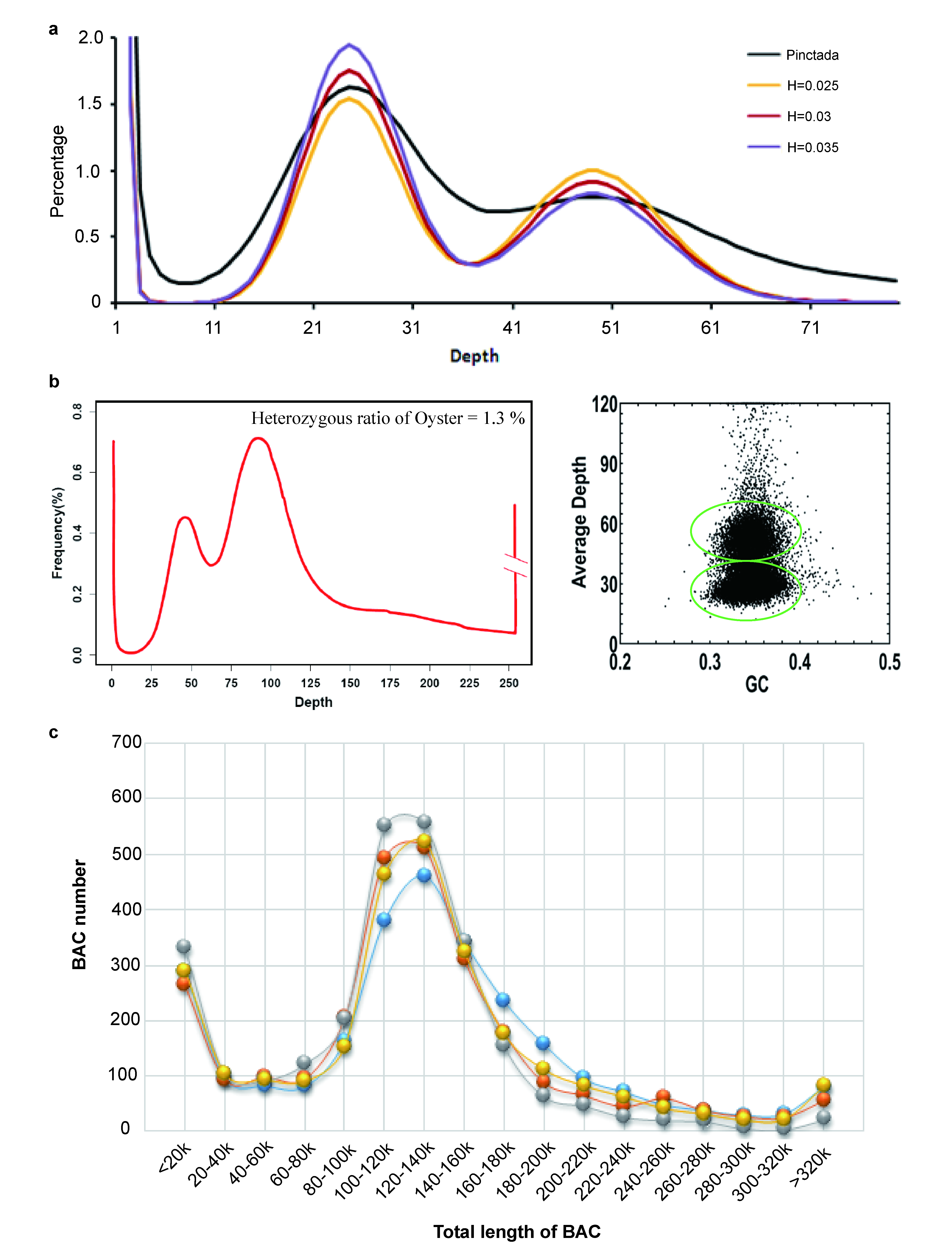

Supplement: Additional Files [file gix059_Supp.zip › Additional file 2.figure S1.tif]

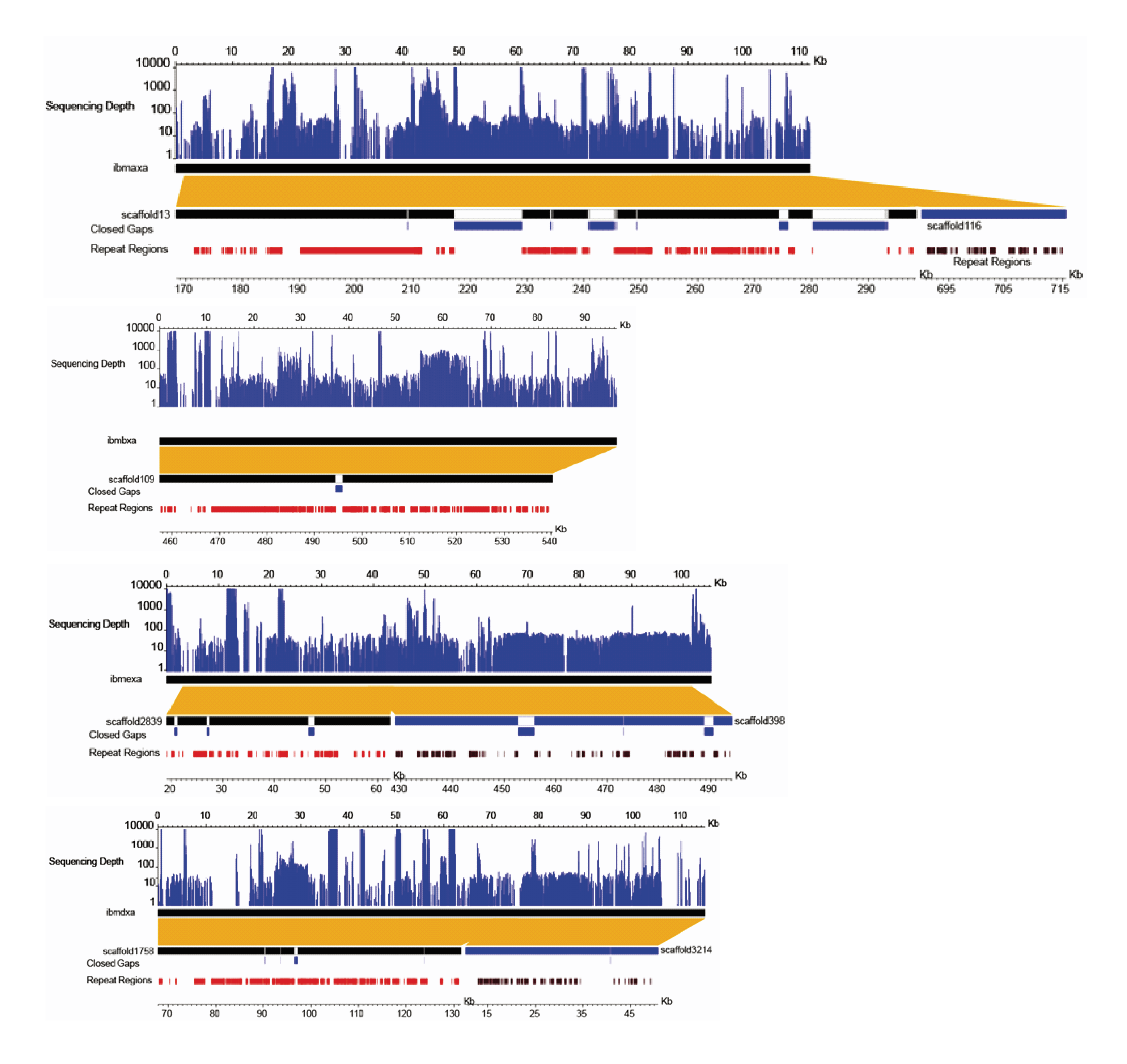

Supplement: Additional Files [file gix059_Supp.zip › Additional file 3.figure S2.tif]

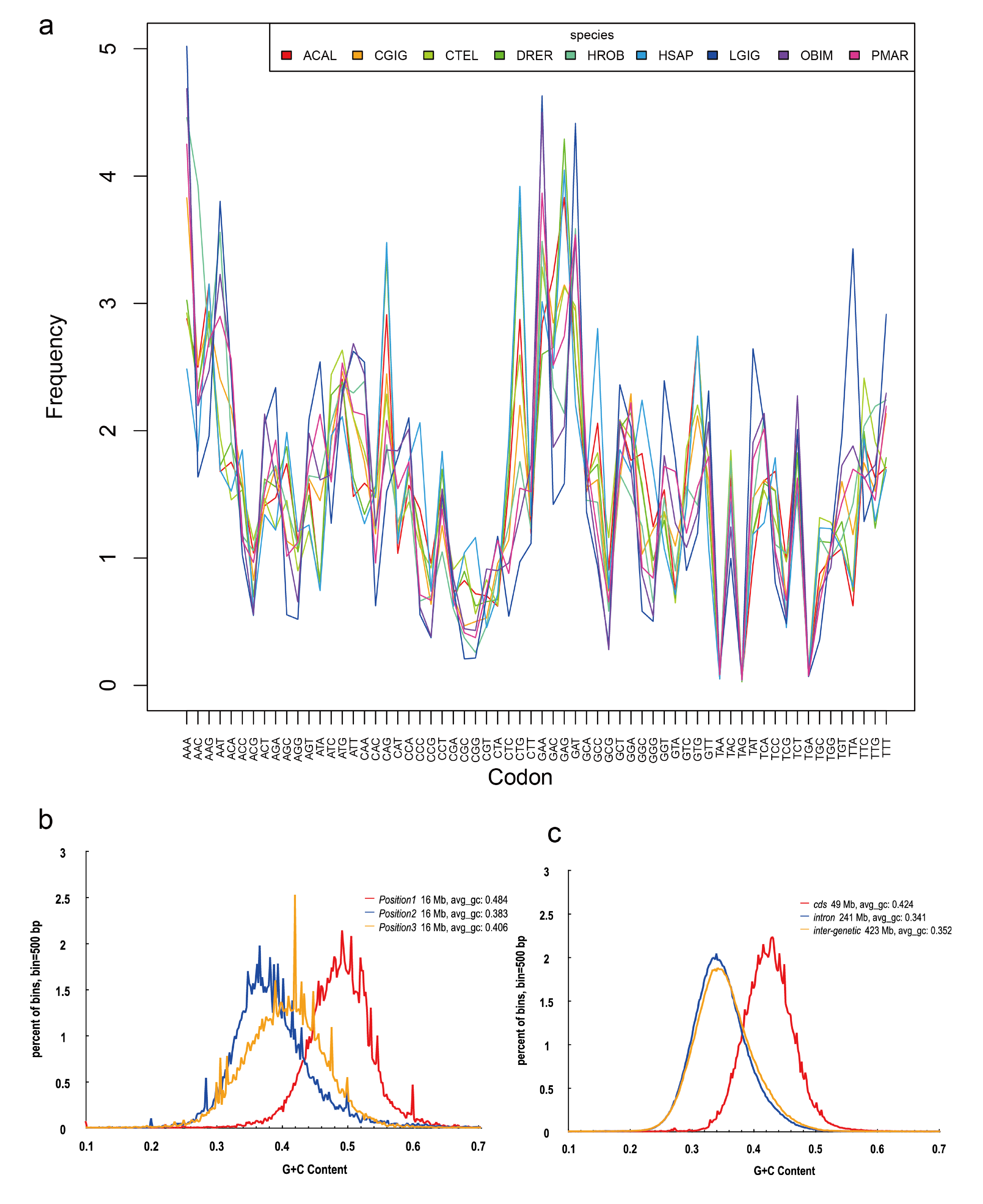

Supplement: Additional Files [file gix059_Supp.zip › Additional file 4.figure S3.tif]

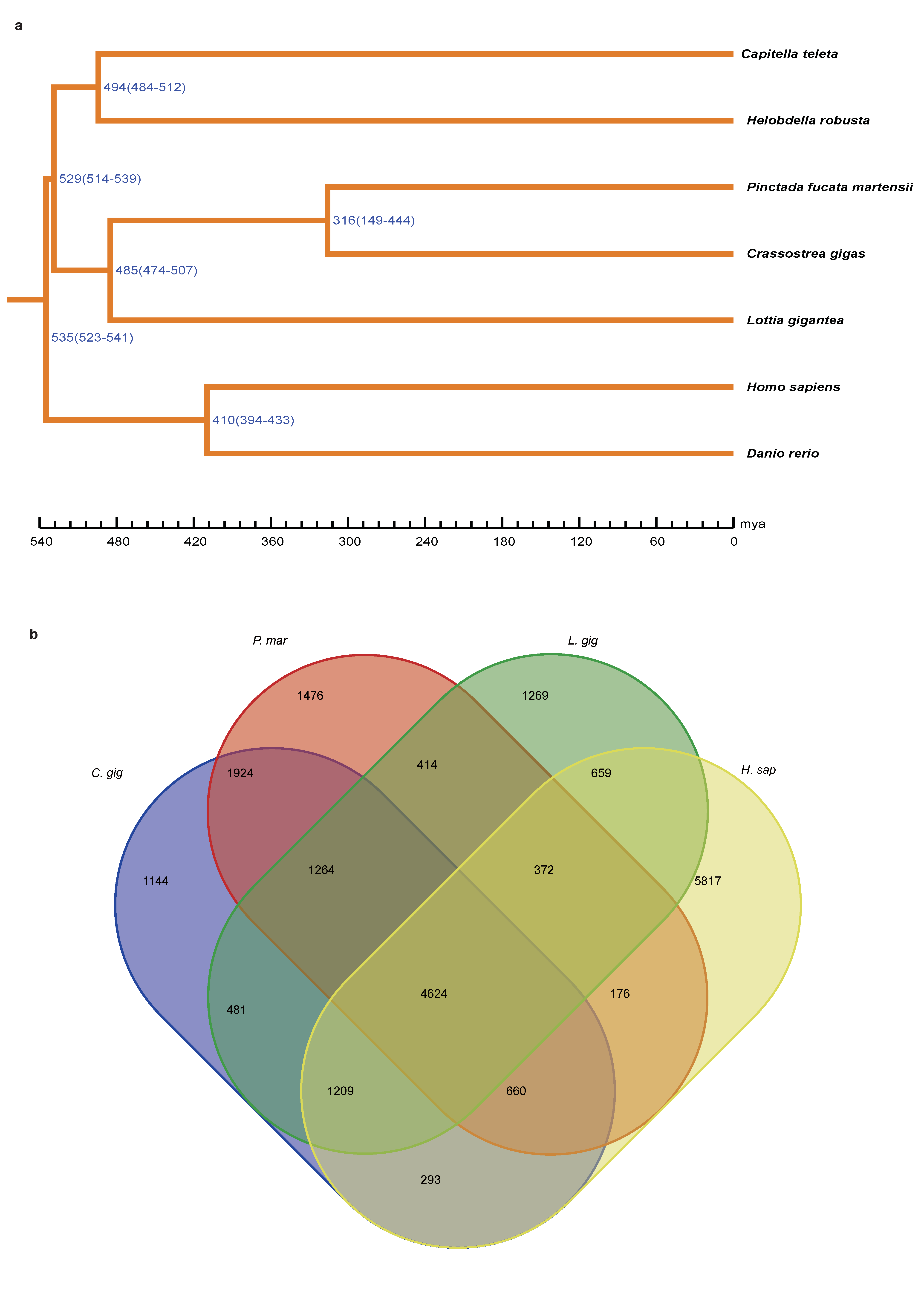

Supplement: Additional Files [file gix059_Supp.zip › Additional file 5.figure S4.tif]

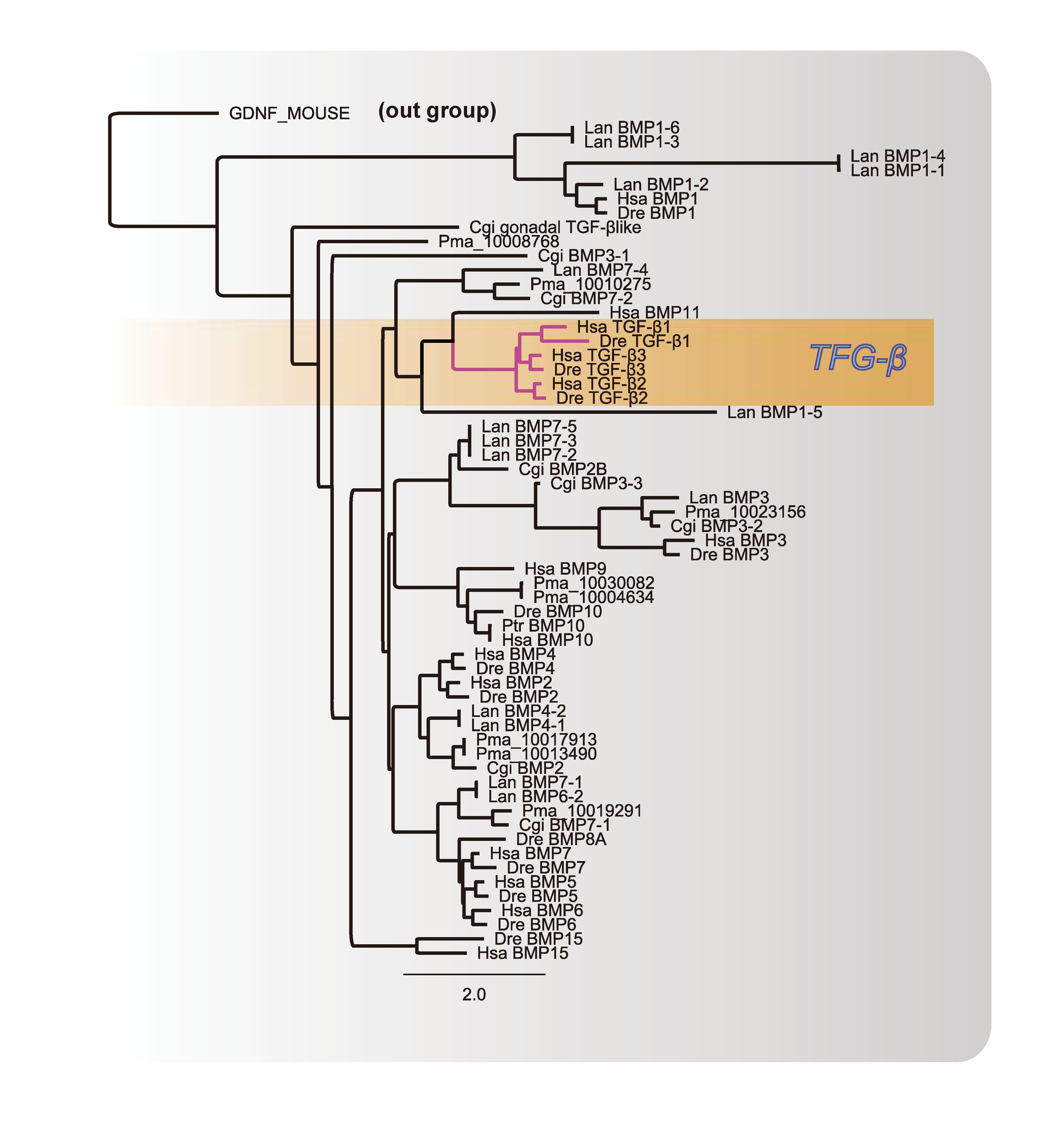

Supplement: Additional Files [file gix059_Supp.zip › Additional file 6.figure S5.tif]

**a***P. f. martensii*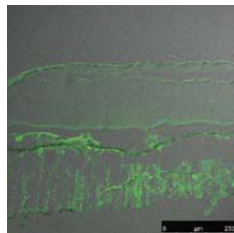*C. gigas*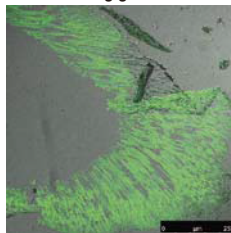**b** *CHS*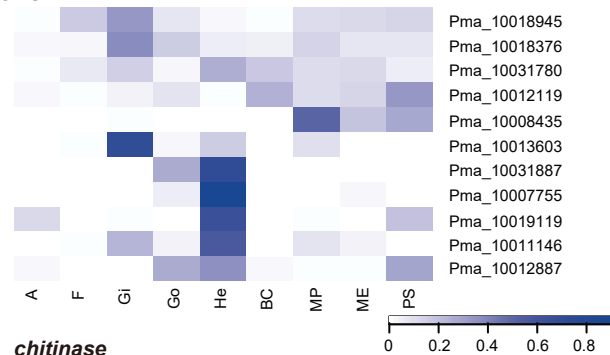**c** *chitinase*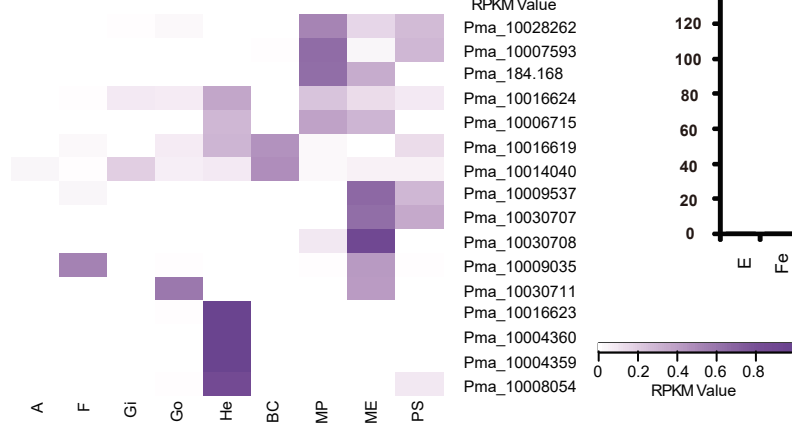**d***chitinase*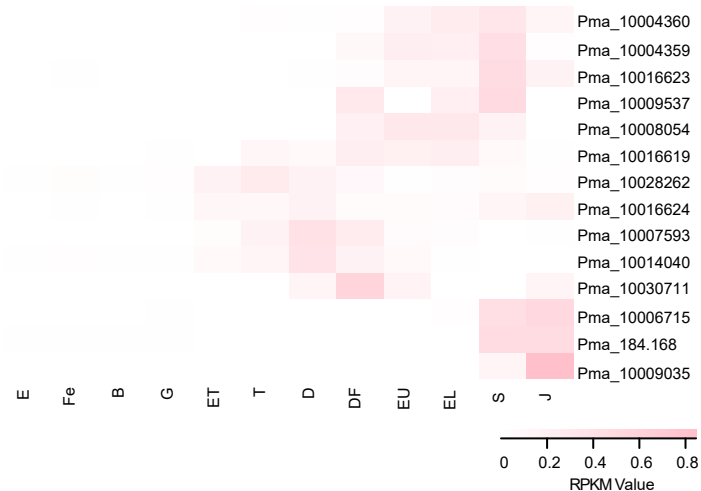

RPKM

*CHS*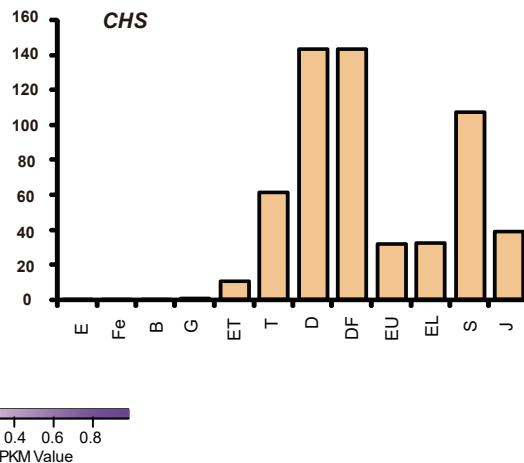

Supplement: Additional Files [file gix059_Supp.zip › Additional file 7. fig S6-0619.pdf]

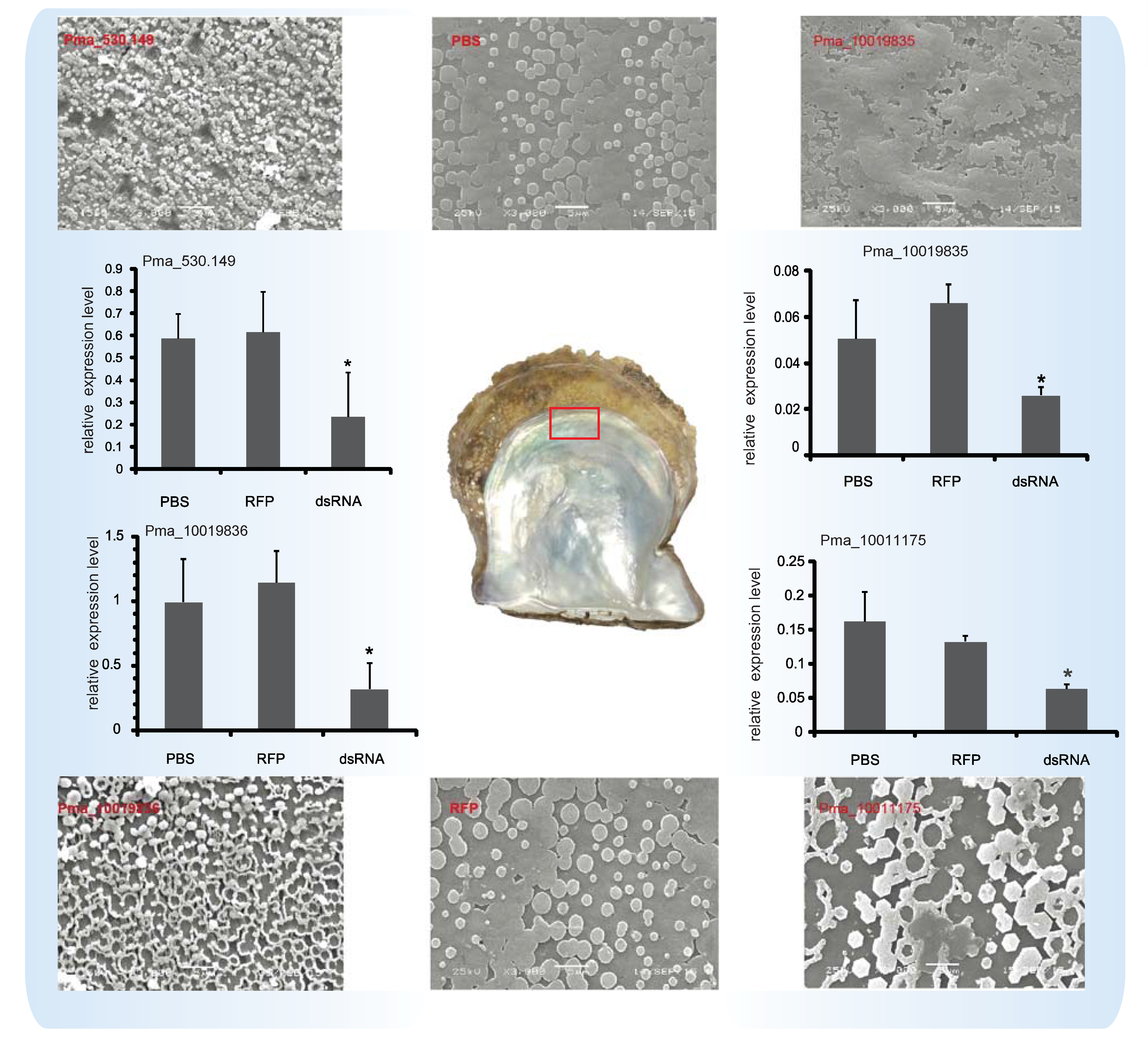

Supplement: Additional Files [file gix059_Supp.zip › Additional file9. figure S7-01.tif]
