# Supplementary material for: The pearl oyster Pinctada fucata martensii genome and multi-omic analyses provide insights into biomineralization
Source: Gigascience. 2017 Jul 25;6(8):1–12. doi: 10.1093/gigascience/gix059 (PMC5597905; doi:10.1093/gigascience/gix059)
Supplement: Response-to-Reviewer-Comments_Original-Submission.pdf [file gix059_Response-to-Reviewer-Comments_Original-Submission.pdf]

Author's Response To Reviewer Comments

Dear Dr. Zauner,

Thank you for the reviews. We have carefully revised our manuscript according to comments and suggestions from the reviewers. A point-to-point response is provided below. We greatly appreciate the reviewers' comments and suggestions which are very constructive and helpful.

We hope the revised manuscript is now acceptable for publication in GigaScience. Please feel free to contact me if you have any questions. We look forward to hearing from you soon.

Sincerely yours,

Xiaodong Du
Point-to-point responses
Please consider to add BUSCO results in addition or instead of CEGMA. BUSCO results are reported for most new genomics submissions to Gigascience and is becoming a standard feature of our genomics papers.
Authors: Thank you for the suggestion. We have included BUSCO results for evaluating the integrity of our assembly and gene annotation. BUSCO analysis shows that our assembly is 82.80% complete and 7.35% fragmented, indicating our assembly is complete enough for further analysis. All the analysis results were added into the revised manuscript.
Responses to Reviewer reports:
Reviewer #1: The manuscript by Du and colleagues reports the assembly and annotation of a high-quality genome of the pearl oyster Pinctada fucata martensii, providing new insights into the genetic basis of mineralization in bivalve molluscs. This work also takes advantage of RADseq and RNA-seq data, which altogether contribute to a significant improvement of the genome assembly and annotation, as well as of a series of additional experiments to investigate specific aspects of the study.
Although a draft genome of the same organism had already been published by Takeuchi and colleagues back in 2012, there is no doubt that the work presented in this study is a significant improvement, as it basically reaches a chromosome-scale quality, allowing large-scale comparative genomics analyses. The annotation process, a relevant issue for non-model lophotrochozoans, also appears to have been greatly implemented in this case. The authors focused the main body of their analysis on the genetic basis of biomineralization, offering a comprehensive model and evidencing the expansion of several gene families which might have a role in this process.
Overall, this ambitious work is of high value and, in my opinion, the P. fucata martensii genome could become a reference species for comparative genomics due to the superior quality of this assembly compared to that of the Pacific oyster C. gigas. However, as any ambitious paper, this work requires some adjustments before it can be accepted for publication. Please find attached below a list of my concerns and comments:
Authors: We appreciate the positive assessment. Below we are point-by-point responses to the comments showing how we revised the manuscript.
Major concerns
I strongly advise the authors to use TPM (Transcripts Per Million) instead of RPKM for the

quantification of gene expression levels, as it enables a more reliable comparability across samples. See Wagner et al. 2012 (doi:10.1007/s12064-012-0162-3). This will likely have only minor effects on the results, assuming that the number of reads per sample and the structure of the transcripomes were similar to each other. A few figures and tables might need to be slightly fixed, e.g. the network analysis and heat maps. Overall this should not bring too many modifications to the interpretation of the results, but in my opinion this is needed to ensure the use of the "gold-standard" methodology available at this time.
Authors: Thank you for the suggestion. We used TPM (Transcripts Per Million) to calculate gene expression of 14 samples, and found excellent correspondence between TPM and RPKM (Correlation coefficient are more than 0.93, Fig. 1), and we especially checked genes appeared in the manuscript and found they are almost identical. Thus, we did not modify the network and heatmap figures in the revised. But we uploaded results of TPM to the GigaDB and the details were added to Methods section.

Fig.1. Correlation between gene expression calculated by TMP and RPKM.

Table S10 and some relevant part of the discussion of results (page 6 in particular) would benefit from the inclusion of a mollusc without a carbonate skeleton,such as Octopus bimaculoides and/or Aplysia californica. The lack or non-expansion of chitinase for example, would de facto confirm the intuition of the authors, or otherwise indicate that this expansion event is not related to the acquisition of a shell. Tyrosinases might be useful for nacre matric cross-linking but, at the same time, they could have other functions in bivalves (some authors have previously suggested a possible role in melanisation/immunity). Use the data from Albertin et al. 2015 (doi:10.1038/nature14668) for what concerns O. bimaculoides, data from Aplysia can be found in NCBI genomes.
Authors: This is a good point. We included Octopus bimaculoides for comparison and added the results to Table S10 in the revised. We revised the results and discussion to include comparison with molluscs without shells.

Page 6, line 47: "expanded". This needs to be better contextualized. Expanded compared to what? The use of such terms should always be referred to a general "baseline" situation which, in this case, could be represented by the inferred most recent common ancestor to all lophotrochozoa or, even better, to the most recent common ancestor of all molluscs (please add Octopus bimaculoides and Aplysia californica in your analysis). EDIT: This part became much more clear after the reading of additional files. However, I still think that the addition of the two aforementioned mollusc species will be beneficial.
Authors: We used the same pipeline and database as before for KEGG annotation of Octopus bimaculoides and Aplysia californica as suggested. Please see the results in the revised Table S10. We also found the expansion of collagen VI and chitinase in Octopus bimaculoides just like in Pinctada fucata martensii and Crassostrea gigas. According to the phylogenetic tree of these species (Fig. 2), our results indicate that the expansion occurred in the recent common ancestor of all molluscs. The expansion of chitin synthase in Lotta gigantea as well as P. fucata martensii and C. gigas, suggests that this expansion occurred in the common ancestor of gastropods and bivalve. The expansion of sulfotransferase in P. fucata martensii and C. gigas suggested that this specific expansion occurred in the common ancestor of P. fucata martensii and C. gigas or bivalves. We revised the manuscript accordingly.

Fig. 2. The phylogenetic tree of 9 species analyzed and the numbers of genes related to shell matrix formation.

Table S14 and "Regulation network of the nacre matrix proteins" section. The authors relied on KEGG annotations for elucidating known pathways enriched in the network of nacre matrix co-expressed genes. KEGG annotation is quite trustworthy, as it relies on a well-known set of evolutionarily conserved genes, but the annotation rate observed in invertebrate genomes with KEGG is systematically lower than that observed with Gene Ontology (in other words, KEGG is somewhat biased on vertebrates). Why did the authors choose not to use GO terms in addition to KEGG annotations for this enrichment analysis? My guess is that the use of GOs might add some useful information to the findings reported in this section.
Authors: The GO enrichment of the co-expressed genes showed that transporter activity (GO:0005215, P=1.86e-10) and ATPase activity (GO: 0042626, P=6.79e-07) are significantly enriched, which is consistent with the enrichment of ABP transporters in KEGG enrichment results. We have added these results in Additional File 1 Table S15.

Minor issues
Page 3, line 33: I would suggest to add a reference from Murgarella et al. 2016 (http://dx.doi.org/10.1371/journal.pone.0151561), as this is a useful example in support of the complexity of bivalve genome assembly, high polymorphism and repetitive content.
Authors: The reference has been added to revised. Thanks.

Page 3, line 59: please define "relatively distant".
Authors: We replaced "two relatively distant parents" with "two genetically distant parents"

Page 4: the first sentence (Raw […]c technology) is unnecessary and can be removed here. Move it to the Additional File 1.
Authors: We removed the sentence (Raw […] technology) as suggested.

Page 5, lines 42-49: it is worth mentioning that these estimates are in agreement with the most up-to-date phylogenomic studies on the evolution of bivalve molluscs (e.g. Bieler et al. 2014, http://dx.doi.org/10.1071/IS13010).
Authors: We agree and have added the sentence. Thanks.

Figure 1e: What is the bootstrap support value for the nodes shown? Did the authors use a bootstrap threshold to show or to collapse nodes? This should be specified in the figure caption.
Authors: We used FastTree to construct the gene tree and FastTree computes local support values with the Shimodaira-Hasegawa test, which resembles the parameter of "SH-like local supports" in PhyML 3. FastTree includes the following stages: 1) Heuristic neighbor-joining; 2) reducing the length of the tree including nearest-neighbor interchanges, subtree-prune-regraft moves and distance model; 3) maximizing the tree's likelihood with NNIs; 4) local support values. We set SH-like local supports =1000 in our analysis. Our detailed parameters are "amino acid distances: BLOSUM45, joins: balanced, support: SH-like 1000, search: normal +NNI +SPR (2 rounds range 10) +ML-NNI, opt-each=1, tophits: 1.00*sqrtN, close=default refresh=0.80, ML model: Jones-Taylor-Thorton, CAT approximation with 20 rate categories". The local support

values are given as names for the internal nodes, and range from 0 to 1, not from 0 to 100. The resulting support values are strongly correlated with the traditional bootstrap (r=0.975). Please see the gene tree figure below with the support values added.

Fig. 3. The gene tree with the local support values for each node.

Figure 2c: same as above. Please replace "value" with RPKM (or TPM, if this is changed).
Authors: We replaced "value" with "RPKM value" in the figure 2b. Because of the excellent correspondence between RPKM and TPM, we did not change RPKM to TPM. Because RPKM values of those genes exhibited significant variation and were difficult to show in one figure, we standardized the RPKM values with the following process. We summed up the RPKM of A gene at twelve developmental stages: ΣA. The value of A gene at i developmental stage: PAi=Ai/ΣA. We sum up the RPKM of A gene in different tissues: ΣAT. The value of A gene in j tissue: PAj=Aj/ΣAT.

Additional File 1, page 10: the sequencing strategy should be specified: was it a single-end or a paired-end strategy? What was the length of the reads? Which software was used to perform the trimming of the reads?
Authors: Paired-end libraries with insert-size of 500 bp were generated from each sample and sequenced separately using Illumina Hiseq 2000. Raw reads, with read length 100bp, were filtered using SOAPfilter (V2.2) to remove those containing adapter sequences, high numbers of "N" and having low sequence quality scores, before further analysis. As suggested, we added these details to Additional File 1.

Additional File 1, page 4: "Genome characterization by K-mer analysis". This should be better explained. Did the authors use Jellyfish or a similar software to perform this analysis?
Authors: We used Kmerfreq (our inner program) for the k-mer analysis but re-analyzed using Jellyfish. We found that the results from the two software are very similar as shown in Fig 4 below. The result and figure have been uploaded into GigaDB.

Fig. 4. K-mer distribution analyzed using Jellyfish and inner kmerfreq.

Repeat content analysis: the repeat library should be made available as a supplementary file.
Authors: Thank you for the suggestion. Because the file of the repeat library is about 26 Mb, which is over the limit for supplementary file, we have uploaded the file into GigaDB database.

Additional File 1, page 8: how was the set of 114 single-copy genes determined? Also, the authors should mention that the sequences were concatenated prior to analysis.
Authors: We identified single-copy genes that have only one gene per family in each species based on gene family results from Treefam. The results of gene families clustering and single-copy genes identified have been uploaded into GigaDB, and the method was described in additional file 1.

Table S11: Cte and Hro species abbreviations are missing. Please check if other are missing as well.
Authors: Added. Thanks.

Additional file 7 (Figure 6): RPKM (or, hopefully, TPM) should be added near "values" under the colour bars.
Authors: Added. Thanks.

The authors are encouraged to add an additional supplementary table summarizing codon usage in P. fucata martensii. While this statistics is often neglected, it is important to investigate selection of certain gene families for translational efficiency and/or accuracy. I would also like to see some additional information concerning GC content of introns, exons, inter-genic regions and GC content at each codon position.
Authors: As suggested, we summarized codon usage in P. fucata martensii and added the statistics to the supplementary file. We found the number of codon usage in P. fucata martensii is similar with other 8 species (Fig. 5). Also, we calculated the GC content of introns (34.1%), exons (42.4%), inter-genic regions (35.2%) and GC content at each codon position (see Fig. 6 below). The results show the GC content of exon is notably higher than that of intron and inter-genic regions. The GC content of position 1 of codon (48.4%) is notably higher than that of position 3 (40.6%) and position 2 of codon (38.3%) (Fig. 7). These figures added in the additional file 4, figure S3

Fig. 5. Comparison of the distribution of codon usage among 9 species.

Fig. 6. The GC content distribution of exon, intron and inter-genetic regions.

Fig. 7. The GC content distribution for each codon position.

Another aspect which has not been investigated concerns the presence of non-coding genes. Currently, the number of non-coding genes annotated in the human genome surpasses that of protein-coding ones. For obvious reasons, our knowledge of such genes in non-model organisms is not equally advanced. How many out of the gene predictions are likely to be non-coding? The authors used an annotation protocol which included the alignment with RNA-seq data, which should be optimal for this purpose. There are a number of ways to predict this, such as CPC (doi: 10.1093/nar/gkm391).
Authors: Thank you for the suggestion. We used CPC to do the prediction and we added these results in the revised. For non-coding RNA prediction, we randomly selected 15 samples from our transcriptome datasets and used TopHat2 [1] to map the raw data to the reference assembly. We used Cufflinks and Cuffmerge to predict transcripts based on TopHat2 alignments, then novel transcripts were generated by performing Cuffcompare against the annotated CDS [2]. Totally 64,409 novel transcripts were generated. We then used coding potential calculator (CPC) [3] based on the UniRef90 database to access the coding potential of these transcripts. We used the same threshold as the work of Jiang et al. [4] and kept the transcripts with coding potential score ≤ -1. Finally, 35,245 transcripts are predicted to be non-coding. All the results have been uploaded into GigaDB.

While the two last points concern topics which go beyond the scope of the paper (mineralization), I believe their assessment will add further value to this interesting paper.

Authors: Thank you for the positive assessment.

Reviewer #2: In their manuscript entitled "The genome of Pinctada fucata martensii and multi-omic analyses provide insights into Matrices of biomineralization", Du and co-workers described the release of a novel and clearly improved version of the pearl oyster genome, that had been previously described by Takeushi et al. (2012), and provide input to biomineralization process characterization, thanks to combined transcriptomic, proteomic and functional characterizations via RNAi experiments. This MS represents an impressive amount of work and also an ambitious challenge for the sequencing and the assembly of this large genome presenting high level of repeated sequences. Although, I like the study that represents a very interesting set of data, I have also some concerns, including significant technical and data interpretation issues, and the MS need major revisions before being considered for publication in GigaScience or in another journal.
Authors: Thank you for the positive assessment and helpful suggestions. Below we provide point-by-point responses to your comments and indicate how we have revised the manuscript.

I have listed here my main concerns that aim at been provided to the authors:
- My main concern go to the over interpretation of some blast results for some identified proteins that provide erroneous indication leading to wrong conclusions. For example, according to supplementary information, Pinctada_martensi_GLEAN_10028879 gave "Tyrosine-protein phosphatase" as first hit of the blast search, however it is not and should be considered at all as a tyrosine-protein phosphatase. Indeed, this protein sequence exhibits 5 fibronectin 3 domains that contribute to give a significant alignment (first rang blast hit) with mammalian tyrosine-protein phosphatase because it contains also 5 FN3 domains, BUT this true tyrosine-protein phosphatase contains also specific tyrosine-protein phosphatase domains, that are critically missing in the Pinctada protein, then it should simply not be considered as a fibronectin. Indeed, various proteins contain FN3 domains which are not exclusive to tyrosine-protein phosphatase. See smart domain list for FN3:
http://smart.embl-heidelberg.de/smart/selective.cgi?domains=FN3&terms=&taxon_text=&input=Architecture+query

- This is also the case of collagen IV blast hit. For similar reason, it seems to me that the different protein that gave "Collagen IV" for Blast hit shouldn't be considered as true Collagen IV proteins. Indeed, when looking at the sequence of these proteins they all exhibit between 1 and 4 VWA domains (ex. Pinctada_martensi_GLEAN_10019836), but far less than the true vertebrate collagen IV that exhibit at least 8 VWA domains in addition to other collagen specific domains (M7B9T5) and sometimes few EGF domains too. For example, the blue mussel PIF isoform, called BMSP by Suzuki and co-workers 2011 in Chembiochem, exhibits similarly 4 VWA domains, together with 3 CBD2, a LamG-like domains and an acidic low-complexity domains, but this protein is, from any literature, not considered and/or named as a "Collagen IV". Indeed various proteins belonging to diverse family exhibits VWA domains (see smart server results for VWA at:
http://smart.embl-heidelberg.de/smart/selective.cgi?domains=vwa&terms=&taxon_text=&input=Architecture+query).

From now, although it represents an appealing idea (with indeed important potential consequences on evolution scenario…) that has been discussed in different article, there is still no evidence of the presence of collagen in mollusc shell. Taken together, I insistently recommend to the author to reconsider this point and to modify their MS accordingly.
Authors: We appreciate the comment. We are very sorry that the descriptions line in the uploaded data was the annotation results using Nr database, which may be inappropriate for some gene, such as Tyrosine-protein phosphatase. Now, we have added the other three annotation results using KO, GO, IPR database, which may be helpful for gene characterization. Regarding the annotation of collagen gene in our manuscripts, firstly, we like to clarify that the collagen type mentioned in our manuscript is "Collagen VI" not "Collagen IV". Concerning the six proteins with VWA domains found in the shell matrix proteins, we annotated them as "Collagen VI" because they are highly homologous with "Collagen VI" annotated by KO database. The annotated results by KO were helpful for us to elucidate the signal transduction pathways underlying biomineralization, which is our focus in the last part of the manuscript. In contrast, Nr database contains miscellaneous information uploaded by researchers with personal views, such as PIF and BMSP, which was annotated by the authors based on gene function and characterization. Among the six shell matrix proteins with VWA domain, 44.534 is annotated as "BMSP" by NCBI blast, but it is annotated as "Collagen VI" by KO database. In this situation, we considered 44.534 as "Collagen VI". In our opinion, this annotation is more informative and helpful for us to explain the evolutionary dynamics of 44.534. PIF is annotated as midasin by KO database, and we didn't target it for further analysis.
We recognize and agree that "various proteins belonging to diverse family exhibits VWA domains", but VWA domains from different families vary in sequences and can be classified by phylogenetic analysis. In the shell matrix proteome, 12 proteins with VWA domains were detected, and only 6 proteins were annotated as "Collagen VI" by KO database. That's to say that we didn't annotate all of the proteins with VWA domains as "Collagen VI". Further, phylogenetic analysis showed the six proteins clustered with "Collagen VI" from other animals, which supports our annotation.
Although structural analysis showed the six matrix proteins don't have the short triple-helix region (THR), we found that all "Collagen VIs" from invertebrates don't have THR (Fig. 2d), and some COL6s without THRs still exist in the human and zebrafish genomes, and more importantly, phylogenetic analysis indicates that COL6 without THR region emerged early during evolution, and fibrillar COL1/2/4 are derived from collagens without THRs (Fig. 2e). Even so, we agreed with the reviewer's suggestion of caution, and we changed the name of these proteins to "Collagen VI like proteins" and have modified our manuscript accordingly. We feel it is important to highlight the homology between these proteins and collagen VI, which has significant implications to the evolution of collagens and shell Matrices.

- I also notice from the amino acid sequences of the matrix proteins the absence of peptide signal (predicted by SignalP4.0) for many of these proteins suggesting that these sequences could be incomplete/not correct and/or that somehow the gene model do not accurately predict all proteins exon/intron (especially in their N-termini). I assume that gene model prediction from a non-model organism, such as Pinctada, constitutes a big challenge, however I think that it more interesting to discuss this point within the MS (rather to simply omit it), as accurate gene prediction is still a change for the scientific community and current strategies are still not perfect, constituting a limitation to search automatic and non manually curated approach.

Authors: Thank you. It is a big scientific challenge for the assembly of genome with high heterozygous ratio and the gene prediction of a non-model organism. The software based on a de Bruijn Graph, such as SOAPdenovo[5], is difficult to obtain a satisfied result due to the increased complexity of the de Bruijn graph structure. Overlap-Layout-Consensus assembler, such as Celera Assembler[6], based on the data of fosmids or BACs hierarchical sequencing and third-generation long reads (such as PacBio long reads) are employed with overcoming such problems. However, the best choice for solving the assembly of complex genome is to obtain the haploiod or homozygous samples to sequence. For the ab initio gene prediction software, such AUGUSTUS[7], the aim of them is to find potential coding sequences with sufficiently long open reading frames, but the translated regions may be very short and the absence of stop codons becomes meaningless. The similarity-based approaches including homologous protein sequences, EST sequences and transcripts assembled from RNA-seq reads can produce biologically relevant predictions, but they may not cover entire coding exons. Due to their strengths and weaknesses, generally, a synthesis software, such as GLEAN[8] and MAKER[9], is used to synthesize these evidences obtained from ab initio gene predictions and similarity-based approaches into the final gene annotation. Further, in our study, we performed the BUSCO analysis[10] to evaluate the integrity of gene annotation, indicating our assembly is complete enough for further analysis. We added this part in the manuscript.
Suggestion: The presence of signal peptide and the accuracy of the gene model could also be investigated and discussed in the MS, as from now most if not all testified mollusc shell proteins exhibit such signal peptide, and that exception remains rare and based on alternative secretion scenario via transmembranal release mechanism (Silva-Ramos et al. 2013 in Mol Biol Evol).
Authors: Thank you for the suggestion. We added the peptide signal prediction of the shell matrix proteins by SignalP4.0. As showed in supplement, there are 269 proteins without typical peptide signal. We agree with the reviewer's suggestion that there may be some alternative secretion scenario via transmembranal release mechanism. We have added the corresponding discuss in the manuscript.
- I really regret that there is no (semi-)quantification of the different proteins described here, as some of the matrix identified proteins from the list are very likely contaminants (not true matrix proteins, rather cellular debris), such as actins, actin-binding proteins, tubulins, myosins, HSP70, ATP-synthase and perhaps many others…. Indeed, it has been now well known that biominerals may contain various contaminants (such as cellular debris proteins…) in addition to the calcifying matrix that is embedded within the mineral phase during the biomineralization processes. It has been shown (Silva-Ramos et al. 2013 in PNAS; Marie et al. in Proteomics; Mann et al., 2012 and 2013 in Proteome Science) that an extent and adequate cleaning of the shell fine powder with HOCl can somehow reduce this contamination. However as specified by Mann et al 2013 in Proteome Science a rational way to deal with this potential contamination (that can theoretically never been completely removed…) is to quantified proteins and to focus only the attention on main and truly matrix proteins.
All proteins that are patent cellular component (such as actins, tubulins, histones, ATPase, …) should be reconsidered and removed from matrix protein list. Mass spectrometry is a very sensitive technics and one should be careful about such contamination of extracellular matrices by cellular contents. The lack of criticism of the proposed protein list represents a significant issue, and the protein list proposed here should be deeply reconsidered (critically refined/refiltered…).

Authors: Concerning the cellular debris proteins in the shell proteome, we agree with the reviewer's opinion that they may be contaminants not real matrix protein, as the amount of these proteins was less abundant than the known shell matrix proteins. Thus, to find the crucial molecular functional in biomineralization, we removed the cellular debris (Additional file 8, S1), and even without removing them, it did not affect COL6-like proteins being one of the most abundant (Additional file 8, S1).
- Tyrosinases were detected in nacre, but from previous literature, they have been mostly described from the prismatic layer (by proteomic). It would be interesting to specify in Pincata fucata martensii the tyrosinase identifications in nacre and to compare them to those in prisms. Previous work have shown that some tyrosinases are more over-expressed in ME than in MP, it seems to be a different picture here, and this point aim being discussed and should gave interesting consideration about the role of these proteins and their activity in nacre, but also prisms and periostracum formation…
Authors: Tyrosinases (Tyr) was reported to be functioning in mediating the crosslink between shell matrix protein by converting tyrosine residues into adhesive DOPA [11, 12]. A total of twelve Tyr were identified from shell proteome. Six of them were detected in both nacreous layer and prismatic layer, indicating their similar function. Two Tyrs were specifically detected in the nacreous layer, and they were over-expressed in MP than ME. Four Tyrs were specifically detected in prismatic layer, and most of them were over-expressed in ME than MP. These results suggest that Tyr functions both in nacreous layer and prismatic layer formation in Pincatada fucata martensii.
The high expression of Tyrs in MP and ME as well as their abundance in shell matrix suggest that Tyrs are an important structural component of shell. Tyr belong to the "type-3 copper" family and have a conserved active site of six histidine residues mediating the binding of copper ion as cofactor [13]. Metal ions such as Cu2+, Zn2+ and Mg2+ are important factor to stable the crystalline form of calcium carbonate [14-16]. The deposition of Tyr and metal ions in the shell may contribute to controlling the concentration of metal ions of extrapallial fluid, and finally help to stabilize the crystalline form. Interestingly, we found that the histidione residues in nacre-specific Tyr (Pm10005159 and 10016044) were seriously replaced with other residues, suggesting the loss of metal ion binding ability. Therefore, we suggest that the function of nacre-specific Tyrs may be different from that of prism-specific Tyrs and need to be further elucidated. We recognize that some Tyrs may have other functions unrelated to shell or shell matrix formation.

- By the way, it seems that the author avoid to cite previous published works on Pinctada shell proteomics by Marie et al. (2012) in PNAS and more recently Liu et al. (2015) in Scientific Reports, then to integrate and compare these information. This curious omission makes the reader (and the rewiever too) getting suspicious about the objectivity of the MS (that is not a good think for a reader…), and I recommend to add and to discuss adequately these references (at least in the discussion).
Authors: Thank you for pointing us to these references which were added and discussed.

- There is no description of the bioinformatics identification of the matrix proteins using a search engine (such as Mascot, ProteinPilot or Xtandem!....), the parameter used, the filters selected….. This information is critically missing. For example, the reader (and I) cannot guess what mean "LSBD", "LSRD", "ZSBD" or "ZSRD", then appreciate this data…

Authors: We used the Mascot software (v 2.3.02) to query the MS/MS spectra data of matrix proteins in the transcriptome database (36396 sequences). We applied the trypsin cleavage rule with one missed cleavage site. Carbamidomethylation of cysteines was considered as the fixed modifications while Gln->pyro-Glu (N-term Q), Oxidation (M) and Deamidated (NQ) were considered as the variable modifications. Peptide mass tolerance was set to 0.05Da and fragment mass tolerance was set to 0.01Da. We used target-decoy search strategy [17] to identify the matrix proteins, and the False Discovery Rate (PDR) was ≤1%. We added the details into Methods and spelled out “PAIP”, “PASP”, “NAIP”, “NASP”, which are abbreviations for “prism acid insoluble protein”, “prism acid soluble protein”, “Nacre acid insoluble protein”, “Nacre acid soluble protein”, respectively.

- It is not specify how the gene model lists (generated according to the three different strategies: de novo, comparison of reference metazoan genomes and comparison with reference Pinctada transcriptomes) were merged into GLEAN db in order to avoid any gene/protein redundancy. This point aim at been specified.
Authors: Thanks for your suggestion. We used the trainGlimmerHMM tool included in the GlimmerHMM software package to estimate a Markov model with 1000 high-quality genes (The genes were previously used to train Augustus). Then we used the Markov model to identify the coding potential of each transcript assembled from the transcriptome data. So, transcripts with complete ORFs were extracted and multiple isoforms from the same locus were collapsed by retaining the longest ORF. Next, we integrated these ORFs with homology-based gene models to form the core gene set. Then we added the homology-based gene models which not supported by transcriptome-based evidence but supported by homologous evidence from at least two species to the core gene set. Finally, we added the de novo-based gene models not supported by homology-based and transcriptome-based evidence to the core gene set. As a result, a total of 32,937 non-redundant protein-coding genes were annotated in our genome assembly.
We also added the BUSCO results in the supplementary file. These method details were added to the revised. We also added BUSCO results in the supplementary file which show that our assembly is 82.80% complete and 7.35% fragmented, complete enough for further analysis.

- As Alcian blue staining specificity is depending on the pH of the buffered solution (Wall & Gui, 1988 in Anal Chem), this information should be provided in the M&M. Alcian blue reveals acidic compounds comprising GAGs, but very acidic protein can also be stained by this method (Marin et al. 2005 in J Biol Chem). Are you sure it is GAGs or not acidic protein that are stained here? Indeed, previous works as shown that Pinctada nacre exhibit a limited amount of acidic polysaccharides (Bédouet et al 2001 in Comp Biochem Physiol B). On the figure 3, I am wondering if it cannot be mucocytes that are stained by blue alcian?
Authors: Periodic acid Schiff (PAS) reaction is a method for detecting neutral and acid polysaccharides. When using a double staining method of pH 2.5 alcian blue and PAS (AB-PAS), neutral glycosaminoglycans (GAG) are stained "red" with Schiff's reagent, and acid GAG are stained "blue" with alcian blue [18].
- I do not find interests in Figure 4, as this model remains unpleasant and poorly informative, and I recommend to remove it (but other supplementary figures are much more interesting and aim at being integrated in the MS as regular, as for example figures S3 or Figure S6 are very fine).
Authors: Thanks. We move the figure S3 into the main manuscript. And we also maintained the

Figure 4.

- I do not agree with the assumption that "no silk protein can be found in the transcriptome and the proteome of Pinctada" (P6, L34-38). The original idea that silk-like protein contribute in matrix-regulated processes of nacre formation was suggested with the identification in 1997 by Sudo et al in Nature of MSI60, which exhibits several poly-Ala domains, such as silk proteins do. Other poly-Ala containing proteins have also been retrieved from the Pinctada shell nacre (NUSP-2 or NUSP-9) by Marie and co-worker (2012) in PNAS.
Authors: It is arguable whether MSI60 and NUSPs are homologs of silk proteins. In our opinion, they are not silk proteins as they show little sequence homology with silk proteins other than poly-Ala and lack the ensemble internal repeats that are characteristic of silk proteins (see also Furuhashi et al., 2009)[19]. We respect the difference in opinion here.

Minor comments:
- The text should be improved, and some sentences remains awkward. I suggest to the authors to get assistance from an English editing service. Some example of sentence to reword: P2, L48-50; P3, L2-3; P3, L42-44; P3, L5; P9, L19-26;…
Authors: Thanks. We have modified the sentences and improved the manuscript in general.

- There is no legend for the "GC content" figure of figure S1.
Authors: Added, thanks.

- Figure S7: expression profile of Pma_44.534 and Pma_10015641 (a) are without shell phenotyping then look quite superflux in comparison with other genes presenting phenotypes (b).
Authors: Thanks, we have modified it.

- Concerning the WGCNA, in the text of the M&M it is indicated that it has been performed with 158 biomineralization genes, but in the result section it is specified that it has been performed with the 234 nacre matrix proteins. Please clarify this point.
Authors: We are so sorry for this mistake. We first used 158 biomineralization genes with at least 5 unique peptides to draw a preliminary network. But, in the final analyses, we used all 234 proteins isolated from nacre to draw and show a more comprehensive co-expression network for nacre formation.

- The most important pathway highlighted by the KEGG analysis is by far "metabolism". Maybe the authors should also specify (and discuss) this observation in their MS?
Authors: We discussed this point in the revised manuscript.

- Please remove "constitute a gel-like substance" (subtitle P7, L37), because no evidence that GAGs indeed constitute a gel-like substance have been provided here.
Authors: Reference is provided to support the statement.

- P9, L16: The shell also contains the myostracum and the periostracum layers.
Authors: We agree and modified accordingly.

- The discussion is too long, and some aspect sounds too speculative. This section should be

shorten.
Authors: We have shorten the discussion somewhat.

- "Matrices": maybe more conventional is "Matrices".
Authors: Corrected, thanks.

- I cannot find a mention on how to access/download the genome or gene model data.
Authors: Added.

Reviewer #3: I was asked to review only the methods used for the de novo genome assembly of this oyster species.

De novo genome assembly can be challenging especially in mullusks where many of the species are known to be highly repetitive and or contain high levels of heterozygosity that can confound an assembly program. As they stated in the manuscript a straightforward assembly resulted in an inadequate assembly. Upon discovering this, the researchers adopted a BAC clone assembly method that is known to overcome some of these issues. The methods of assembling individual BACs and taking the best assembly from each BAC is reasonable. These assembled BACs were scaffold using a program I am not familiar with Rabbit, however, since it requires long overlaps for the assembly I suspect this program is more than adequate to perform the higher level assembly of joining BACs. The quality of the assembly appears to be high with scaffolds containing almost all of the CEGMA genes. The use of the genetic map to pull 86% of the assembly into chromosomes suggests this is a good assembly.
Authors: Thanks for your appreciation.

I believe this assembly can be published as is. However, I would like to make sure that all raw data is available (raw reads used to assemble BACs, assembled BACs, assembled Scaffolds, genetic map marker information (not just the centamorgan position but also the sequence for each marker).
Authors: We have uploaded assembled BACs, assembled Scaffolds and genetic map marker information to GigaDB. We did not upload raw reads that were used to assemble BACs to GigaDB, because the amount of data is too large.

Any further speculation of the quality of the assembled scaffolds could easily be put to rest if they used the genetic map to show there were no inconsistencies with the assembled scaffolds. I suspect they did this and broke scaffolds that were inconsistent. SSPACE is known to be a greedy scaffolder so I wouldn't be surprised if there are a couple of scaffolds that needed to be broken apart based on the genetic map. If this was done by the authors a short addition to the supplementary methods would be good to see.
Authors: Thanks a lot. It is a good suggestion. Firstly, we set a strict cutoff of pair-end reads supports (>=5) in the SSPACE to reduce the errors of link in the scaffolds. For the conflicts between genetic map and scaffolds from SSPACE, we chose to trust the results of SSPACE, if the number of genetic markers located on this scaffold is less than 3, i.e., we maintained the scaffolds and ignored their distributions on the genetic map. If the number of markers of this scaffold is more than 3 and there is no conflict between markers, we will check the links of

scaffold, such as the length of gaps and the supports of pair-end reads. Particularly, we pay special attention to the scaffolds with a gap (the length of them is more than 10kb), but the length of each end of the contig sequences are less than 200bp. If the insertsize of supporting pair-end reads is abnormal, we will break the links.

Overall, I feel the authors did a great job with this assembly considering the challenges they faced and with the anchored scaffolds may represent one of the best assembly we currently have for a mullusk species.
Authors: Thanks again.
--
Please also take a moment to check our website at http://giga.edmgr.com/l.asp?i=5221&l=GKT84E5U for any additional comments that were saved as attachments. Please note that as GigaScience has a policy of open peer review, you will be able to see the names of the reviewers.

Reference

1. Kim D, Pertea G, Trapnell C, Pimentel H, Kelley R, Salzberg SL. TopHat2: accurate alignment of transcriptomes in the presence of insertions, deletions and gene fusions. Genome Biol. 2013;14:R36.
2. Trapnell C, Williams BA, Pertea G, Mortazavi A, Kwan G, van Baren MJ, et al. Transcript assembly and quantification by RNA-Seq reveals unannotated transcripts and isoform switching during cell differentiation. Nat Biotechnol. 2010;28:511-515.
3. Kong L, Zhang Y, Ye ZQ, Liu XQ, Zhao SQ, Wei L, et al. CPC: assess the protein-coding potential of transcripts using sequence features and support vector machine. Nucleic Acids Res. 2007;35:W345-349.
4. Jiang JJ, Cheng LH, Wu H, He YH, Kong QP. Insights into long noncoding RNAs of naked mole rat (Heterocephalus glaber) and their potential association with cancer resistance. Epigenetics Chromatin. 2016;9:51.
5. Luo R, Liu B, Xie Y, Li Z, Huang W, Yuan J, et al. SOAPdenovo2: an empirically improved memory-efficient short-read de novo assembler. Gigascience. 2012;1:18.
6. Myers EW, Sutton GG, Delcher AL, Dew IM, Fasulo DP, Flanigan MJ, et al. A whole-genome assembly of Drosophila. Science. 2000;287:2196-2204.
7. Stanke M, Keller O, Gunduz I, Hayes A, Waack S, Morgenstern B. AUGUSTUS: ab initio prediction of alternative transcripts. Nucleic Acids Res. 2006;34:W435-439.
8. Elsik CG, Mackey AJ, Reese JT, Milshina NV, Roos DS, Weinstock GM. Creating a honey bee consensus gene set. Genome Biol. 2007;8:R13.
9. Cantarel BL, Korf I, Robb SM, Parra G, Ross E, Moore B, et al. MAKER: an easy-to-use annotation pipeline designed for emerging model organism genomes. Genome Res. 2008;18:188-196.
10. Simao FA, Waterhouse RM, Ioannidis P, Kriventseva EV, Zdobnov EM. BUSCO: assessing genome assembly and annotation completeness with single-copy orthologs. Bioinformatics. 2015;31:3210-3212.
11. Aguilera F, Mcdougall C, Degnan BM. Evolution of the tyrosinase gene family in bivalve molluscs: Independent expansion of the mantle gene repertoire ☆. Acta Biomaterialia.

2014;10:3855-3865.
12. Zhang G, Fang X, Guo X, Li L, Luo R, Xu F, et al. The oyster genome reveals stress adaptation and complexity of shell formation. Nature. 2012;490:49-54.
13. Decker H, Schweikardt T, Tuczek F. The first crystal structure of tyrosinase: all questions answered? ChemInform. 2006;45:4546–4550.
14. Kitano Y, Kanamori N, Yoshioka S. Adsorption of zinc and copper ions on calcite and aragonite and its influence on the transformation of aragonite to calcite. Geochemical Journal. 1976;10:175-179.
15. Berner R. The role of magnesium in the crystal growth of calcite and aragonite from sea water. Geochimica et Cosmochimica Acta. 1975;39:489-504.
16. Nassrallah-Aboukais N, Boughriet A, Laureyns J, Aboukais A, Fischer J, Langelin H, et al. Transformation of vaterite into cubic calcite in the presence of copper (II) species. Chemistry of materials. 1998;10:238-243.
17. Elias JE, Gygi SP. Target-decoy search strategy for increased confidence in large-scale protein identifications by mass spectrometry. Nature methods. 2007;4:207-214.
18. Hasumi M, Nagahama Y. Seasonal Dynamics of Reproductive Organs in Male Salamanders of the Species Hynobius nigrescens. Copeia. 1990;1990:367.
19. Furuhashi T, Schwarzinger C, Miksik I, Smrz M, Beran A. Molluscan shell evolution with review of shell calcification hypothesis. Comparative biochemistry and physiology Part B: Biochemistry and molecular biology. 2009;154:351-371.
