# Supplementary material for: The pearl oyster Pinctada fucata martensii genome and multi-omic analyses provide insights into biomineralization
Source: Gigascience. 2017 Jul 25;6(8):1–12. doi: 10.1093/gigascience/gix059 (PMC5597905; doi:10.1093/gigascience/gix059)
Supplement: Response-to-Reviewer-Comments_Revision-1.pdf [file gix059_Response-to-Reviewer-Comments_Revision-1.pdf]

Author's Response To Reviewer Comments

Dear Dr. Zauner,

Thank you for the review. We have carefully revised our manuscript according to comments and suggestions from the reviewer. A point-to-point response is provided below. We hope the revised manuscript is now acceptable for publication in GigaScience. Please feel free to contact me if you have any questions. We look forward to hearing from you soon.

Sincerely yours,

Xiaodong Du

Point-to-point responses
The latest reports are below. As you will see, reviewer 2 insists that the protein annotation needs to be approached with more refined methods than automatic blast searches, to avoid wrong annotations that may cause confusion. I feel the reviewer is right to be concerned about this point and I hope you will be able to fully address this remaining concern in a second revised manuscript.
Authors: Thank you for the suggestion. We have conducted more detailed analysis and revised the gene annotation as suggested by the reviewer in the revised manuscript.

Responses to Reviewer reports:
My overall appreciation of this resubmitted MS is that, although the authors have provided a detailed point-by-point answer to the referee comments, they fail in fully considering the encountered issues concerning wrong automatic annotation of some of the proteins (simply based on Blast result, without any further validation that could have been provided by considering reciprocal blast or syntenic context of the genes), especially for those, such as collagen VI, on which the author have based their scenario of biomineralization evolution. Automatic annotation of the genomes remains a pitfall for genome description, and scientists should reinforce their effort in applying complementary approaches for validation of the data.
More specially, the proteins identified here as collagen VI present 3 VWA domains, such as many other non-collagenous proteins (BMSP-220 from the blue mussel for example!), when true collagen VI proteins exhibits a specific association of 8 VWA domains (plus additional domains…), and although the blast gave best hits with proteins wrongly called collagen VI from Crassostrea gigas automatic genome annotation (that contains many error and aim at being amended…) the authors should be much more careful before claiming such conclusions.
This case remains especially critical, as it is already a long debate to know if the biomineralized structures of non-bony fish contains collagen proteins (such as bones do) or not, and here the conclusion that collagen VI are part of Pinctada's shell structures is largely over-speculative and non-correct. If such mistaken interpretation of the data would be published, especially in a high impact

journal such as Gigasciences, it would be largely misleading for the scientific community.
So for these reason, I firmly still do not recommend the publication of the resubmitted manuscript in its present form and suggest to the author to perform in their MS some major corrections of their blast data, then their interpretation and discussion. This manuscript presents otherwise an important set of data presenting high interests for the scientific community.

Authors: We appreciate and understand the reviewer's comment. We conducted in-depth analyses of domain structures of the proteins under question and agree with the reviewer that these proteins should not be called collagen VI-like proteins as they lack the triple-helix repeat (THR) domain common to all collagens. Initially, we conducted reciprocal blasts of the six collagen-like proteins found in the shell matrix proteome of P. f. martensii. The best hits of five of these proteins were collagen VI (Table 1). Vice versa, blasting P. f. martensii genome with mouse collagen VI (NP_766515.2) also identified four of these proteins as the best hits (Table 2). These hits were primarily due to the VWA domains and the fact that collagen VI contains more VWAs. Since these P. f. martensii proteins do not have the THR domain characteristic for collagens, we agree with the reviewer it is prudent not naming them as collagen VI or collagen-like proteins. We renamed them as VWA-containing proteins (VWAPs) and revised our discussions accordingly.

Table 1 Best hit of five collagen 6-like protein
gene_ID Dre value Score Hsa value Score Mmu value Score
Pma_44.534 XP_009305855.2 col6a6 2.00E-13 76.6 XP_016865741.1 col12a1 2.00E-12 74.3
NP_081039.2 col6a4 3.00E-13 76.3
Pma_530.149 XP_009305855.2 col6a6 2.00E-55 207 XP_005246122.1 col6a3 3.00E-49 190 NP_081039.2
col6a4 1.00E-53 203
Pma_10011175 XP_017213296.1 col6a3 4.00E-170 599 NP_004360.2 col6a3 6.00E-119 429
NP_001229937.1col6a3 2.00E-111 404
Pma_10015641 XP_017211973.1 matrilin-4 1.00E-14 80.5 XP_016868907.1 matrilin-2 1.00E-11 72
NP_058042.2
matrilin-2 1.00E-11 72
Pma_10019835 XP_009305855.2 col6a6 9.00E-45 177 XP_016861200.1 col6a6 5.00E-43 173
NP_766515.2 col6a6 1.00E-42 171
Pma_10019836 XP_017207020.1 col6a6 1.00E-42 167 XP_016861200.1 col6a6 5.00E-41 163
NP_766515.2 col6a6 2.00E-41 163

Table 2 Reciprocal blast results by NP_766515.2 collagen alpha-6(VI) chain isoform 2 precursor [Mus musculus]

gene ID	score	E value	KO	IPR
Pma_10031686	413	e-115	K06238 collagen, type VI, alpha	IPR002035; von Willebrand factor, type A
Pma_10011175	328	1.00E-89	K06239 collagen, type VI, alpha	IPR000152; EGF-type aspartate/asparagine hydroxylation site
				IPR000742; Epidermal growth factor-like domain
				IPR000884; Thrombospondin, type 1 repeat
				IPR001881; EGF-like calcium-binding
				IPR002035; von Willebrand factor, type A
				IPR006209; EGF-like domain
				IPR006210; Epidermal growth factor-like
				IPR013032; EGF-like region, conserved site
				IPR018097; EGF-like calcium-binding, conserved site
Pma_10008424	283	8.00E-76	K06240 collagen, type VI, alpha	IPR000884; Thrombospondin, type 1 repeat
				IPR001007; von Willebrand factor, type C
				IPR002035; von Willebrand factor, type A
				IPR003582; Metridin-like ShK toxin IPR006552; VWC out
Pma_530.149	206	7.00E-53	K06241 collagen, type VI, alpha	IPR002035; von Willebrand factor, type A
Pma_10005105	185	2.00E-46	K06242 collagen, type VI, alpha	IPR002035; von Willebrand factor, type A
Pma_10019835	181	4.00E-45	K06243 collagen, type VI, alpha	IPR002035; von Willebrand factor, type A
Pma_10003943	177	6.00E-44	K06244 collagen, type VI, alpha	IPR002035; von Willebrand factor, type A
Pma_10019836	172	1.00E-42	K06245 collagen, type VI, alpha	IPR002035; von Willebrand factor, type A
