# Supplementary material for: The pearl oyster Pinctada fucata martensii genome and multi-omic analyses provide insights into biomineralization
Source: Gigascience. 2017 Jul 25;6(8):1–12. doi: 10.1093/gigascience/gix059 (PMC5597905; doi:10.1093/gigascience/gix059)
Supplement: Response-to-Reviewer-Comments_Revision-2.pdf [file gix059_Response-to-Reviewer-Comments_Revision-2.pdf]

Author's Response To Reviewer Comments

Dear Dr. Zauner,

Thank you for the comments and suggestions. We have carefully revised our manuscript accordingly. A point-to-point response is provided below. We hope the revised manuscript is now acceptable for publication in GigaScience. Please feel free to contact me if you have any questions. We look forward to hearing from you soon.

Sincerely yours,

Xiaodong Du

Point-to-point responses

- Please re-consider the number of joint first authors. We notice you have 11 joint first authors. It does not seem feasible that every single author has done exactly the same amount of work. You can provide information on everyones roles in the appropriate section at the end of the paper, so people are aware who did what. Please make sure you follow our criteria for authorship. This is based on definitions from International Committee of Medical Journal Editors and Committee on Publication Ethics, authorship is about responsibility and credit for making an intellectual contribution to the study. Acquisition of funding, collection of data, or general supervision of the research group, alone, does not justify authorship. I have pasted excerpts from our guidelines before.

Authors: Thank you for the comment. After discusions among our team, we decide to retain the following seven first authors: Xiaodong Du, Guangyi Fan, Yu Jiao, He Zhang, Ximing Guo, Ronglian Huang and Zhe Zheng. We feel contributions from these seven authors are equally important. The following is a brief summary of author contributions that is added to the revised ms:

X.D., X.G., G.Z. and X.L. designed scientific objectives. Y.J., R.H., W.C., X.X, Q.S. and G.F. managed the project. H.Z., F.S., X.L., C.S., W.L., H.L. and Z.W. performed genome assembly, gene annotation and evolution analyses. Y.D., Q.W. and X.Z. cultured P. f. martensii and provided materials. Y.D., Q.W., F.S., J.B. and Z.W. constructed the genetic map. Q.W., Z.Z., J.L. and R.H. performed the acid GAG analysis. Y.J., Z.Z., R.H. and J.L. performed chitin and VWAPs related analyses. R.H., C.B. and Z.Z. performed tyrosinase related analysis. Z.Z. and H.Z. performed the WGCNA analysis. X.D., G.F. and X.G. directed final data analyses. Y.J., X.G. and Z.X. directed critical revisions of intellectual content. X.D., X.G. and Y.J. supervised all aspects of the work to ensure the accuracy or integrity of the research and data. X.D., G.F., Y.J., H.Z., X.G., R.H., Z.Z. did most of the writing with contributions from all authors.

- We also note that you indicate four corresponding authors. OUP has a policy of only taking one - most responsive - author as corresponding author. Please note that, according to the guidelines, the definition of corresponding authorship is one of responsiveness rather than seniority. The corresponding author is the one individual who takes primary responsibility for communication with the journal during

the manuscript submission, peer review, and publication process, Again, please refer to the information below and on our homepage and please decide based on these guidelines who should be corresponding author.
Authors: Thank you for the suggestion. We see that the OUP's policy was adopted starting January 1, 2017. Our article was submitted in 2016. The four corresponding authors did a lot of work and contributed significantly to the correspondence related this manuscript. They represent four principle institutions that jointly led the project. We hope we could keep the four corresponding authors. Thank you for your understanding.
- Please add all relevant accession numbers to your manuscript (such as NCBI, PRIDE) and make sure the data are released and accessible. (If the PRIDE submission is a full submission, please cite the DOI. If it's a partial submission, add the accession.)
Authors: All relevant accession numbers were added to the revised paper.
- We recommend to mention the common name of the species in the title, in addition to the scientific name.
Authors: Thank you for the recommendation. The title was modified as "The pearl oyster Pinctada fucata martensii genome and multi-omic analyses provide insights into biomineralization".
- Please mention the GigaDB dataset in your data availability section, using the following format: "Data supporting the manuscript is also available via the GigaDB database [64]" and please add the citation of the GigaDB dataset to your reference list, using this format as template:
(this is just an example): Zheng L-Y, Guo X-S, He B, Sun L-J, Peng Y, Dong S-S, et al. Supporting data for "Title of your paper". GigaScience Database. 2017. http://dx.doi.org/xxxxxxxxx.
(If you do not have your final GigaDB DOI yet, just put XXX instead of the doi, and we'll add this for you)
Authors: Thanks. We have added the citation in reference list in this article.
- please remove highlighting/tracking of changes that was made for the purpose of review only.
Authors: Thanks. Highlighting/tracking of changes were removed.
- If you wish and it is available, please feel free to add a photograph of the oyster species as a Figure to the paper.
Authors: Thanks. We have prepared a picture of pearl oyster P. f. martensii as a Figure (figure 6) to the paper and submitted in the submission system.
