# Supplementary material for: The pearl oyster Pinctada fucata martensii genome and multi-omic analyses provide insights into biomineralization
Source: Gigascience. 2017 Jul 25;6(8):1–12. doi: 10.1093/gigascience/gix059 (PMC5597905; doi:10.1093/gigascience/gix059)
Supplement: Response-to-Reviewer-Comments_Revision-3.pdf [file gix059_Response-to-Reviewer-Comments_Revision-3.pdf]

Author's Response To Reviewer Comments

Dear Dr. Zauner,

Thank you for your comments and suggestions on our third revision. We have carefully considered the points you raised and revised the ms accordingly. A point-to-point response is provided below.

We hope that the revised ms is acceptable for GigaScience. Please feel free to contact me if you have other questions or comments.

Thank you again for considering our ms.

Sincerely yours,

Xiaodong Du

Response to comments

1. Please mention PRIDE accession numbers for you mass spectrometry data in the "data availability" section.

Response: PRIDE accession numbers have been added. Pinctada fucata martensii shell matrix protein LC-MSMS project accession is PXD006786.

2. Please carefully go over your manuscript once again to correct typos and grammar.

Response: We proof-read the ms and fixed typos and grammatical errors.

3. Regarding the multiple first and corresponding authors:

Response: We appreciate the exception that you and Dr. Goodman have granted for the author listing. We apologize for not providing a clear description and justification of authorship in our previous revisions.

We have reviewed ICMJE’s guidelines on authorship and the comments from Dr. Goodman and you, which we agree. We will follow these guidelines more closely in future submissions. Here we confirm that all authors have made substantial contributions that we believe merit author. All authors contributed to drafting and revising the ms, approved the submission and agreed to be accountable for all aspects of the work.

This genome project lasted four years and represent a major effort of a large research team. The team

were organized into different groups, focusing on different aspects of the study. The seven co-first authors represent key members/leaders of the team who has made critical contributions. Although their roles differed and their tasks are not identical, we believe that their contributions are equally important. It is difficult to present notebooks to document the diverse activities by the seven authors. The following is a summary description of their contributions:

X.D. conceived the study. X.D. and X.G. designed scientific objectives and directed final data analysis and interpretation. X.G. and X.D. contributed significantly to writing, revising and intellectual development of the ms.

G.F. and H.Z. designed sequencing strategies, directed and contributed the most to genome sequencing and assembly.

Y.J. designed and led studies on chitin and VWAPs, and contributed the most to findings on chitin and VWAPs. R.H. designed and led studies on tyrosinases, and contributed the most to findings related tryosinases. Z.Z designed and led studies on GAGs and WGCNA analysis, and contributed the most to findings on GAGs and WGCNA.

We revised the author's contribution as follow:

X.D. conceived the study. X.D., X.G., G.Z. and X.L. designed scientific objectives. X.D. and X.G. directed final data analysis and interpretation. G.F. and H.Z. designed sequencing strategies, directed and contributed the most to genome sequencing and assembly. Y.J. (designer and leader), J.L. and Z.W. conducted studies on chitin and VWAPs and performed data analysis. R.H. (designer and leader), C.B. and H.L. conducted studies on tyrosinases and performed data analysis. Z.Z. (designer and leader), Q.W. and H.Z. conducted studies on GAGs and performed data and WGCNA analyses. F.S., X.L., C.S., W.L. and X.X. participated in genome assembly, gene annotation and evolution analyses. Y.D., J.B., Q.S., W.C., and X.Z. constructed the genetic map. J.L. and R.H. conducted in RNAi experiments. Y.J., X.G. and Z.X. directed critical revisions of intellectual content. X.D., X.G., X.L. and G.Z. supervised all aspects of the work to ensure the accuracy or integrity of the research and data. All authors contributed to writing and revision, and approved the submission.
