# Supplementary material for: The pearl oyster Pinctada fucata martensii genome and multi-omic analyses provide insights into biomineralization
Source: Gigascience. 2017 Jul 25;6(8):1–12. doi: 10.1093/gigascience/gix059 (PMC5597905; doi:10.1093/gigascience/gix059)
Supplement: Reviewer-1-Report-(Original-Submission).pdf [file gix059_Reviewer-1-Report-(Original-Submission).pdf]

Reviewer Report

Title: The pearl oyster Pinctada fucata martensii genome and multi-omic analyses provide insights into biomineralization

Version: Original Submission **Date:** 9/19/2016

Reviewer name: Marco Gerdol

Reviewer Comments to Author:

The manuscript by Du and colleagues reports the assembly and annotation of a high-quality genome of the pearl oyster Pinctada fucata martensii, providing new insights into the genetic basis of mineralization in bivalve molluscs. This work also takes advantage of RADseq and RNA-seq data, which altogether contribute to a significant improvement of the genome assembly and annotation, as well as of a series of additional experiments to investigate specific aspects of the study.
Although a draft genome of the same organism had already been published by Takeuchi and colleagues back in 2012, there is no doubt that the work presented in this study is a significant improvement, as it basically reaches a chromosome-scale quality, allowing large-scale comparative genomics analyses. The annotation process, a relevant issue for non-model lophotrochozoans, also appears to have been greatly implemented in this case. The authors focused the main body of their analysis on the genetic basis of biomineralization, offering a comprehensive model and evidencing the expansion of several gene families which might have a role in this process.
Overall, this ambitious work is of high value and, in my opinion, the P. fucata martensii genome could become a reference species for comparative genomics due to the superior quality of this assembly compared to that of the Pacific oyster C. gigas. However, as any ambitious paper, this work requires some adjustments before it can be accepted for publication. Please find attached below a list of my concerns and comments:

Major concerns
I strongly advise the authors to use TPM (Transcripts Per Million) instead of RPKM for the quantification of gene expression levels, as it enables a more reliable comparability across samples. See Wagner et al. 2012 (doi:10.1007/s12064-012-0162-3). This will likely have only minor effects on the results, assuming that the number of reads per sample and the structure of the transcripomes were similar to each other. A few figures and tables might need to be slightly fixed, e.g. the network analysis and heat maps. Overall this should not bring too many modifications to the interpretation of the results, but in my opinion this is needed to ensure the use of the "gold-standard" methodology available at this time.

Table S10 and some relevant part of the discussion of results (page 6 in particular) would benefit from the inclusion of a mollusc without a carbonate skeleton, such as Octopus bimaculoides and/or Aplysia californica. The lack or non-expansion of chitinase for example, would de facto confirm the intuition of the authors, or otherwise indicate that this expansion event is not related to the acquisition of a shell.

Tyrosinases might be useful for nacre matric cross-linking but, at the same time, they could have other functions in bivalves (some authors have previously suggested a possible role in melanisation/immunity). Use the data from Albertin et al. 2015 (doi:10.1038/nature14668) for what concerns O. bimaculoides, data from Aplysia can be found in NCBI genomes.

Page 6, line 47: "expanded". This needs to be better contextualized. Expanded compared to what? The use of such terms should always be referred to a general "baseline" situation which, in this case, could be represented by the inferred most recent common ancestor to all lophotrochozoa or, even better, to the most recent common ancestor of all molluscs (please add Octopus bimaculoides and Aplysia californica in your analysis). EDIT: This part became much more clear after the reading of additional files. However, I still think that the addition of the two aforementioned mollusc species will be beneficial.

Table S14 and "Regulation network of the nacre matrix proteins" section. The authors relied on KEGG annotations for elucidating known pathways enriched in the network of nacre matrix co-expressed genes. KEGG annotation is quite trustworthy, as it relies on a well-known set of evolutionarily conserved genes, but the annotation rate observed in invertebrate genomes with KEGG is systematically lower than that observed with Gene Ontology (in other words, KEGG is somewhat biased on vertebrates). Why did the authors choose not to use GO terms in addition to KEGG annotations for this enrichment analysis? My guess is that the use of GOs might add some useful information to the findings reported in this section.

Minor issues
Page 3, line 33: I would suggest to add a reference from Murgarella et al. 2016 (http://dx.doi.org/10.1371/journal.pone.0151561), as this is a useful example in support of the complexity of bivalve genome assembly, high polymorphism and repetitive content.

Page 3, line 59: please define "relatively distant".

Page 4: the first sentence (Raw […] technology) is unnecessary and can be removed here. Move it to the Additional File 1.

Page 5, lines 42-49: it is worth mentioning that these estimates are in agreement with the most up-to-date phylogenomic studies on the evolution of bivalve molluscs (e.g. Bieler et al. 2014, http://dx.doi.org/10.1071/IS13010).

Figure 1e: What is the bootstrap support value for the nodes shown? Did the authors use a bootstrap threshold to show or to collapse nodes? This should be specified in the figure caption.

Figure 2c: same as above. Please replace "value" with RPKM (or TPM, if this is changed).

Additional File 1, page 10: the sequencing strategy should be specified: was it a single-end or a paired-end strategy? What was the length of the reads? Which software was used to perform the trimming of

the reads?

Additional File 1, page 4: "Genome characterization by K-mer analysis". This should be better explained. Did the authors use Jellyfish or a similar software to perform this analysis?

Repeat content analysis: the repeat library should be made available as a supplementary file.

Additional File 1, page 8: how was the set of 114 single-copy genes determined? Also, the authors should mention that the sequences were concatenated prior to analysis.

Table S11: Cte and Hro species abbreviations are missing. Please check if other are missing as well.

Additional file 7 (Figure 6): RPKM (or, hopefully, TPM) should be added near "values" under the colour bars.

The authors are encouraged to add an additional supplementary table summarizing codon usage in P. fucata martensii. While this statistics is often neglected, it is important to investigate selection of certain gene families for translational efficiency and/or accuracy. I would also like to see some additional information concerning GC content of introns, exons, inter-genic regions and GC content at each codon position.

Another aspect which has not been investigated concerns the presence of non-coding genes. Currently, the number of non-coding genes annotated in the human genome surpasses that of protein-coding ones. For obvious reasons, our knowledge of such genes in non-model organisms is not equally advanced. How many out of the gene predictions are likely to be non-coding? The authors used an annotation protocol which included the alignment with RNA-seq data, which should be optimal for this purpose. There are a number of ways to predict this, such as CPC (doi: 10.1093/nar/gkm391).

While the two last points concern topics which go beyond the scope of the paper (mineralization), I believe their assessment will add further value to this interesting paper.

Methods

Are the methods appropriate to the aims of the study, are they well described, and are necessary controls included? Yes

Conclusions

Are the conclusions adequately supported by the data shown? Yes

Reporting Standards

Does the manuscript adhere to the journal's guidelines on [minimum standards of reporting?]() YesChoose an item.

Statistics

Are you able to assess all statistics in the manuscript, including the appropriateness of statistical tests used? Yes, and I have assessed the statistics in my report.

Quality of Written English

Please indicate the quality of language in the manuscript: Acceptable

Declaration of Competing Interests

Please complete a declaration of competing interests, considering the following questions:

- Have you in the past five years received reimbursements, fees, funding, or salary from an organisation that may in any way gain or lose financially from the publication of this manuscript, either now or in the future?
- Do you hold any stocks or shares in an organisation that may in any way gain or lose financially from the publication of this manuscript, either now or in the future?
- Do you hold or are you currently applying for any patents relating to the content of the manuscript?
- Have you received reimbursements, fees, funding, or salary from an organization that holds or has applied for patents relating to the content of the manuscript?
- Do you have any other financial competing interests?
- Do you have any non-financial competing interests in relation to this paper?

If you can answer no to all of the above, write 'I declare that I have no competing interests' below. If your reply is yes to any, please give details below.

I declare that I have no competing interests

I agree to the open peer review policy of the journal. I understand that my name will be included on my report to the authors and, if the manuscript is accepted for publication, my named report including any attachments I upload will be posted on the website along with the authors' responses. I agree for my report to be made available under an Open Access Creative Commons CC-BY license (http://creativecommons.org/licenses/by/4.0/). I understand that any comments which I do not wish to be included in my named report can be included as confidential comments to the editors, which will not be published.

I agree to the open peer review policy of the journal

To further support our reviewers, we have joined with Publons, where you can gain additional credit to further highlight your hard work (see: https://publons.com/journal/530/gigascience). On publication of

this paper, your review will be automatically added to Publons, you can then choose whether or not to claim your Publons credit. I understand this statement.

Yes
