# Supplementary material for: The pearl oyster Pinctada fucata martensii genome and multi-omic analyses provide insights into biomineralization
Source: Gigascience. 2017 Jul 25;6(8):1–12. doi: 10.1093/gigascience/gix059 (PMC5597905; doi:10.1093/gigascience/gix059)
Supplement: Reviewer-2-Report-(Original-Submission).pdf [file gix059_Reviewer-2-Report-(Original-Submission).pdf]

Reviewer Report

Title: The pearl oyster Pinctada fucata martensii genome and multi-omic analyses provide insights into biomineralization

Version: Original Submission **Date:** 9/21/2016

Reviewer name: Benjamin Marie

Reviewer Comments to Author:

In their manuscript entitled "The genome of Pinctada fucata martensii and multi-omic analyses provide insights into matrixes of biomineralization", Du and co-workers described the release of a novel and clearly improved version of the pearl oyster genome, that had been previously described by Takeushi et al. (2012), and provide input to biomineralization process characterization, thanks to combined transcriptomic, proteomic and functional characterizations via RNAi experiments. This MS represents an impressive amount of work and also an ambitious challenge for the sequencing and the assembly of this large genome presenting high level of repeated sequences. Although, I like the study that represents a very interesting set of data, I have also some concerns, including significant technical and data interpretation issues, and the MS need major revisions before being considered for publication in GigaScience or in another journal.

I have listed here my main concerns that aim at been provided to the authors:
- My main concern go to the over interpretation of some blast results for some identified proteins that provide erroneous indication leading to wrong conclusions. For example, according to supplementary information, Pinctada_martensi_GLEAN_10028879 gave "Tyrosine-protein phosphatase" as first hit of the blast search, however it is not and should be considered at all as a tyrosine-protein phosphatase. Indeed, this protein sequence exhibits 5 fibronectin 3 domains that contribute to give a significant alignment (first rang blast hit) with mammalian tyrosine-protein phosphatase because it contains also 5 FN3 domains, BUT this true tyrosine-protein phosphatase contains also specific tyrosine-protein phosphatase domains, that are critically missing in the Pinctada protein, then it should simply not be considered as a fibronectin. Indeed, various proteins contain FN3 domains which are not exclusive to tyrosine-protein phosphatase. See smart domain list for FN3: http://smart.embl-heidelberg.de/smart/selective.cgi?domains=FN3&terms=&taxon_text=&input=Architecture+query

- This is also the case of collagen IV blast hit. For similar reason, it seems to me that the different protein that gave "Collagen IV" for Blast hit shouldn't be considered as true Collagen IV proteins. Indeed, when looking at the sequence of these proteins they all exhibit between 1 and 4 VWA domains (ex. Pinctada_martensi_GLEAN_10019836), but far less than the true vertebrate collagen IV that exhibit at least 8 VWA domains in addition to other collagen specific domains (M7B9T5) and sometimes few EGF domains too. For example, the blue mussel PIF isoform, called BMSP by Suzuki and co-workers 2011 in Chembiochem, exhibits similarly 4 VWA domains, together with 3 CBD2, a LamG-like domains and an acidic low-complexity domains, but this protein is, from any literature, not considered and/or named as

a "Collagen IV". Indeed various proteins belonging to diverse family exhibits VWA domains (see smart server results for VWA at: http://smart.embl-heidelberg.de/smart/selective.cgi?domains=vwa&terms=&taxon_text=&input=Architecture+query). From now, although it represents an appealing idea (with indeed important potential consequences on evolution scenario…) that has been discussed in different article, there is still no evidence of the presence of collagen in mollusc shell. Taken together, I insistently recommend to the author to reconsider this point and to modify their MS accordingly.

- I also notice from the amino acid sequences of the matrix proteins the absence of peptide signal (predicted by SignalP4.0) for many of these proteins suggesting that these sequences could be incomplete/not correct and/or that somehow the gene model do not accurately predict all proteins exon/intron (especially in their N-termini). I assume that gene model prediction from a non-model organism, such as Pinctada, constitutes a big challenge, however I think that it more interesting to discuss this point within the MS (rather to simply omit it), as accurate gene prediction is still a change for the scientific community and current strategies are still not perfect, constituting a limitation to search automatic and non manually curated approach.
Suggestion: The presence of signal peptide and the accuracy of the gene model could also be investigated and discussed in the MS, as from now most if not all testified mollusc shell proteins exhibit such signal peptide, and that exception remains rare and based on alternative secretion scenario via transmembranal release mechanism (Silva-Ramos et al. 2013 in Mol Biol Evol).

- I really regret that there is no (semi-)quantification of the different proteins described here, as some of the matrix identified proteins from the list are very likely contaminants (not true matrix proteins, rather cellular debris), such as actins, actin-binding proteins, tubulins, myosins, HSP70, ATP-synthase and perhaps many others…. Indeed, it has been now well known that biominerals may contain various contaminants (such as cellular debris proteins…) in addition to the calcifying matrix that is embedded within the mineral phase during the biomineralization processes. It has been shown (Silva-Ramos et al. 2013 in PNAS; Marie et al. in Proteomics; Mann et al., 2012 and 2013 in Proteome Science) that an extent and adequate cleaning of the shell fine powder with HOCl can somehow reduce this contamination. However as specified by Mann et al 2013 in Proteome Science a rational way to deal with this potential contamination (that can theoretically never been completely removed…) is to quantified proteins and to focus only the attention on main and truly matrix proteins.
All proteins that are patent cellular component (such as actins, tubulins, histones, ATPase, …) should be reconsidered and removed from matrix protein list. Mass spectrometry is a very sensitive technics and one should be careful about such contamination of extracellular matrices by cellular contents. The lack of criticism of the proposed protein list represents a significant issue, and the protein list proposed here should be deeply reconsidered (critically refined/refiltered…).

- Tyrosinases were detected in nacre, but from previous literature, they have been mostly described from the prismatic layer (by proteomic). It would be interesting to specify in Pincata fucata martensii the tyrosinase identifications in nacre and to compare them to those in prisms. Previous work have shown that some tyrosinases are more over-expressed in ME than in MP, it seems to be a different picture

here, and this point aim being discussed and should gave interesting consideration about the role of these proteins and their activity in nacre, but also prisms and periostracum formation…

- By the way, it seems that the author avoid to cite previous published works on Pinctada shell proteomics by Marie et al. (2012) in PNAS and more recently Liu et al. (2015) in Scientific Reports, then to integrate and compare these information. This curious omission makes the reader (and the rewiever too) getting suspicious about the objectivity of the MS (that is not a good think for a reader…), and I recommend to add and to discuss adequately these references (at least in the discussion).

- There is no description of the bioinformatics identification of the matrix proteins using a search engine (such as Mascot, ProteinPilot or Xtandem!....), the parameter used, the filters selected…... This information is critically missing. For example, the reader (and I) cannot guess what mean "LSBD", "LSRD", "ZSBD" or "ZSRD", then appreciate this data…

- It is not specify how the gene model lists (generated according to the three different strategies: de novo, comparison of reference metazoan genomes and comparison with reference Pinctada transcriptomes) were merged into GLEAN db in order to avoid any gene/protein redundancy. This point aim at been specified.

- As Alcian blue staining specificity is depending on the pH of the buffered solution (Wall & Gui, 1988 in Anal Chem), this information should be provided in the M&M. Alcian blue reveals acidic compounds comprising GAGs, but very acidic protein can also be stained by this method (Marin et al. 2005 in J Biol Chem). Are you sure it is GAGs or not acidic protein that are stained here? Indeed, previous works as shown that Pinctada nacre exhibit a limited amount of acidic polysaccharides (Bédouet et al 2001 in Comp Biochem Physiol B). On the figure 3, I am wondering if it cannot be mucocytes that are stained by blue alcian?

- I do not find interests in Figure 4, as this model remains unpleasant and poorly informative, and I recommend to remove it (but other supplementary figures are much more interesting and aim at being integrated in the MS as regular, as for example figures S3 or Figure S6 are very fine).

- I do not agree with the assumption that "no silk protein can be found in the transcriptome and the proteome of Pinctada" (P6, L34-38). The original idea that silk-like protein contribute in matrix-regulated processes of nacre formation was suggested with the identification in 1997 by Sudo et al in Nature of MSI60, which exhibits several poly-Ala domains, such as silk proteins do. Other poly-Ala containing proteins have also been retrieved from the Pinctada shell nacre (NUSP-2 or NUSP-9) by Marie and co-worker (2012) in PNAS.

Minor comments:
- The text should be improved, and some sentences remains awkward. I suggest to the authors to get assistance from an English editing service. Some example of sentence to reword: P2, L48-50; P3, L2-3; P3, L42-44; P3, L5; P9, L19-26;…

- There is no legend for the "GC content" figure of figure S1.
- Figure S7: expression profile of Pma_44.534 and Pma_10015641 (a) are without shell phenotyping then look quite superflux in comparison with other genes presenting phenotypes (b).
- Concerning the WGCNA, in the text of the M&M it is indicated that it has been performed with 158 biomineralization genes, but in the result section it is specified that it has been performed with the 234 nacre matrix proteins. Please clarify this point.

- The most important pathway highlighted by the KEGG analysis is by far "metabolism". Maybe the authors should also specify (and discuss) this observation in their MS?
- Please remove "constitute a gel-like substance" (subtitle P7, L37), because no evidence that GAGs indeed constitute a gel-like substance have been provided here.
- P9, L16: The shell also contains the myostracum and the periostracum layers.
- The discussion is too long, and some aspect sounds too speculative. This section should be shorten.
- "Matrixes": maybe more conventional is "Matrices".
- I cannot find a mention on how to access/download the genome or gene model data.

Methods

Are the methods appropriate to the aims of the study, are they well described, and are necessary controls included? Yes

Conclusions

Are the conclusions adequately supported by the data shown? No

Reporting Standards

Does the manuscript adhere to the journal's guidelines on minimum standards of reporting? YesChoose an item.

Statistics

Are you able to assess all statistics in the manuscript, including the appropriateness of statistical tests used? There are no statistics in the manuscript.

Quality of Written English

Please indicate the quality of language in the manuscript: Needs some language corrections before being published

Declaration of Competing Interests

Please complete a declaration of competing interests, considering the following questions:

- Have you in the past five years received reimbursements, fees, funding, or salary from an organisation that may in any way gain or lose financially from the publication of this manuscript, either now or in the future?
- Do you hold any stocks or shares in an organisation that may in any way gain or lose financially from the publication of this manuscript, either now or in the future?
- Do you hold or are you currently applying for any patents relating to the content of the manuscript?
- Have you received reimbursements, fees, funding, or salary from an organization that holds or has applied for patents relating to the content of the manuscript?
- Do you have any other financial competing interests?
- Do you have any non-financial competing interests in relation to this paper?

If you can answer no to all of the above, write 'I declare that I have no competing interests' below. If your reply is yes to any, please give details below.

I declare that I have no competing interests

I agree to the open peer review policy of the journal. I understand that my name will be included on my report to the authors and, if the manuscript is accepted for publication, my named report including any attachments I upload will be posted on the website along with the authors' responses. I agree for my report to be made available under an Open Access Creative Commons CC-BY license (http://creativecommons.org/licenses/by/4.0/). I understand that any comments which I do not wish to be included in my named report can be included as confidential comments to the editors, which will not be published.

I agree to the open peer review policy of the journal

To further support our reviewers, we have joined with Publons, where you can gain additional credit to further highlight your hard work (see: https://publons.com/journal/530/gigascience). On publication of this paper, your review will be automatically added to Publons, you can then choose whether or not to claim your Publons credit. I understand this statement.

Yes
