# Supplementary material for: The pearl oyster Pinctada fucata martensii genome and multi-omic analyses provide insights into biomineralization
Source: Gigascience. 2017 Jul 25;6(8):1–12. doi: 10.1093/gigascience/gix059 (PMC5597905; doi:10.1093/gigascience/gix059)
Supplement: Reviewer-2-Report-(Revision-1).pdf [file gix059_Reviewer-2-Report-(Revision-1).pdf]

Reviewer Report

Title: The pearl oyster Pinctada fucata martensii genome and multi-omic analyses provide insights into biomineralization

Version: Revision 1 **Date:** 1/22/2017

Reviewer name: Benjamin Marie

Reviewer Comments to Author:

My overall appreciation of this resubmitted MS is that, although the authors have provided a detailed point-by-point answer to the referee comments, they fail in fully considering the encountered issues concerning wrong automatic annotation of some of the proteins (simply based on Blast result, without any further validation that could have been provided by considering reciprocal blast or syntenic context of the genes), especially for those, such as collagen VI, on which the author have based their scenario of biomineralization evolution. Automatic annotation of the genomes remains a pitfall for genome description, and scientists should reinforce their effort in applying complementary approaches for validation of the data.

More specially, the proteins identified here as collagen VI present 3 VWA domains, such as many other non-collagenous proteins (BMSP-220 from the blue mussel for example!), when true collagen VI proteins exhibits a specific association of 8 VWA domains (plus additional domains…), and although the blast gave best hits with proteins wrongly called collagen VI from Crassostrea gigas automatic genome annotation (that contains many error and aim at being amended…) the authors should be much more careful before claiming such conclusions.

This case remains especially critical, as it is already a long debate to know if the biomineralized structures of non-bony fish contains collagen proteins (such as bones do) or not, and here the conclusion that collagen VI are part of Pinctada's shell structures is largely over-speculative and non-correct. If such mistaken interpretation of the data would be published, especially in a high impact journal such as Gigasciences, it would be largely misleading for the scientific community.

So for these reason, I firmly still do not recommend the publication of the resubmitted manuscript in its present form and suggest to the author to perform in their MS some major corrections of their blast data, then their interpretation and discussion. This manuscript presents otherwise an important set of data presenting high interests for the scientific community.

Methods

Are the methods appropriate to the aims of the study, are they well described, and are necessary controls included? Yes

Conclusions

Are the conclusions adequately supported by the data shown? No

Reporting Standards

Does the manuscript adhere to the journal's guidelines on [minimum standards of reporting?](#) YesChoose an item.

Statistics

Are you able to assess all statistics in the manuscript, including the appropriateness of statistical tests used? No, and I do not feel adequately qualified to assess the statistics.

Quality of Written English

Please indicate the quality of language in the manuscript: Acceptable

Declaration of Competing Interests

Please complete a declaration of competing interests, considering the following questions:

- Have you in the past five years received reimbursements, fees, funding, or salary from an organisation that may in any way gain or lose financially from the publication of this manuscript, either now or in the future?
- Do you hold any stocks or shares in an organisation that may in any way gain or lose financially from the publication of this manuscript, either now or in the future?
- Do you hold or are you currently applying for any patents relating to the content of the manuscript?
- Have you received reimbursements, fees, funding, or salary from an organization that holds or has applied for patents relating to the content of the manuscript?
- Do you have any other financial competing interests?
- Do you have any non-financial competing interests in relation to this paper?

If you can answer no to all of the above, write 'I declare that I have no competing interests' below. If your reply is yes to any, please give details below.

I declare that I have no competing interests

I agree to the open peer review policy of the journal. I understand that my name will be included on my report to the authors and, if the manuscript is accepted for publication, my named report including any attachments I upload will be posted on the website along with the authors' responses. I agree for my report to be made available under an Open Access Creative Commons CC-BY license (http://creativecommons.org/licenses/by/4.0/). I understand that any comments which I do not wish to be included in my named report can be included as confidential comments to the editors, which will not be published.

I agree to the open peer review policy of the journal

To further support our reviewers, we have joined with Publons, where you can gain additional credit to further highlight your hard work (see: https://publons.com/journal/530/gigascience). On publication of this paper, your review will be automatically added to Publons, you can then choose whether or not to claim your Publons credit. I understand this statement.

Yes
