# Supplementary material for: The pearl oyster Pinctada fucata martensii genome and multi-omic analyses provide insights into biomineralization
Source: Gigascience. 2017 Jul 25;6(8):1–12. doi: 10.1093/gigascience/gix059 (PMC5597905; doi:10.1093/gigascience/gix059)
Supplement: Reviewer-3-Report-(Original-Submission).pdf [file gix059_Reviewer-3-Report-(Original-Submission).pdf]

Reviewer Report

Title: The pearl oyster Pinctada fucata martensii genome and multi-omic analyses provide insights into biomineralization

Version: Original Submission **Date:** 10/13/2016

Reviewer name: Andrew Severin

Reviewer Comments to Author:

I was asked to review only the methods used for the de novo genome assembly of this oyster species.

De novo genome assembly can be challenging especially in mullusks where many of the species are known to be highly repetitive and or contain high levels of heterozygosity that can confound an assembly program. As they stated in the manuscript a straightforward assembly resulted in an inadequate assembly. Upon discovering this, the researchers adopted a BAC clone assembly method that is known to overcome some of these issues. The methods of assembling individual BACs and taking the best assembly from each BAC is reasonable. These assembled BACs were scaffold using a program I am not familiar with Rabbit, however, since it requires long overlaps for the assembly I suspect this program is more than adequate to perform the higher level assembly of joining BACs. The quality of the assembly appears to be high with scaffolds containing almost all of the CEGMA genes. The use of the genetic map to pull 86% of the assembly into chromosomes suggests this is a good assembly.

I believe this assembly can be published as is. However, I would like to make sure that all raw data is available (raw reads used to assemble BACs, assembled BACs, assembled Scaffolds, genetic map marker information (not just the centamorgan position but also the sequence for each marker).

Any further speculation of the quality of the assembled scaffolds could easily be put to rest if they used the genetic map to show there were no inconsistencies with the assembled scaffolds. I suspect they did this and broke scaffolds that were inconsistent. SSPACE is known to be a greedy scaffolder so I wouldn't be surprised if there are a couple of scaffolds that needed to be broken apart based on the genetic map. If this was done by the authors a short addition to the supplementary methods would be good to see.

Overall, I feel the authors did a great job with this assembly considering the challenges they faced and with the anchored scaffolds may represent one of the best assembly we currently have for a mullusk species.

Methods

Are the methods appropriate to the aims of the study, are they well described, and are necessary controls included? Yes

Conclusions

Are the conclusions adequately supported by the data shown? Yes

Reporting Standards

Does the manuscript adhere to the journal's guidelines on [minimum standards of reporting?]() YesChoose an item.

Statistics

Are you able to assess all statistics in the manuscript, including the appropriateness of statistical tests used? Yes, and I have assessed the statistics in my report.

Quality of Written English

Please indicate the quality of language in the manuscript: Acceptable

Declaration of Competing Interests

Please complete a declaration of competing interests, considering the following questions:

- Have you in the past five years received reimbursements, fees, funding, or salary from an organisation that may in any way gain or lose financially from the publication of this manuscript, either now or in the future?
- Do you hold any stocks or shares in an organisation that may in any way gain or lose financially from the publication of this manuscript, either now or in the future?
- Do you hold or are you currently applying for any patents relating to the content of the manuscript?
- Have you received reimbursements, fees, funding, or salary from an organization that holds or has applied for patents relating to the content of the manuscript?
- Do you have any other financial competing interests?
- Do you have any non-financial competing interests in relation to this paper?

If you can answer no to all of the above, write 'I declare that I have no competing interests' below. If your reply is yes to any, please give details below.

I declare that I have no competing interests

I agree to the open peer review policy of the journal. I understand that my name will be included on my report to the authors and, if the manuscript is accepted for publication, my named report including any attachments I upload will be posted on the website along with the authors' responses. I agree for my report to be made available under an Open Access Creative Commons CC-BY license (http://creativecommons.org/licenses/by/4.0/). I understand that any comments which I do not wish to be included in my named report can be included as confidential comments to the editors, which will not be published.

I agree to the open peer review policy of the journal

To further support our reviewers, we have joined with Publons, where you can gain additional credit to further highlight your hard work (see: https://publons.com/journal/530/gigascience). On publication of this paper, your review will be automatically added to Publons, you can then choose whether or not to claim your Publons credit. I understand this statement.

Yes
